# Conditional wealth to estimate association of wealth mobility with health and human capital in low- and middle-income country cohorts

**DOI:** 10.1186/s12874-022-01757-9

**Published:** 2022-10-27

**Authors:** Jithin Sam Varghese, Clive Osmond, Aryeh D. Stein

**Affiliations:** 1 Laney Graduate School, Nutrition and Health Sciences Program, Emory University, Atlanta, GA USA; 2 Medical Research Council Lifecourse Epidemiology Unit, University of Southampton, Southampton, UK; 3 Hubert Department of Global Health, Rollins School of Public Health, Emory University, 1518 Clifton Rd NE, Atlanta, GA #7007 USA

**Keywords:** Asset index, Conditional wealth, Life course epidemiology, Social mobility, Socioeconomic position

## Abstract

Temporally harmonized asset indices allow the study of changes in relative wealth (mean, variance, social mobility) over time and its association with adult health and human capital in cohort studies. Conditional measures are the unexplained residuals of an indicator regressed on its past values. Using such measures, previously used to study the relative importance of key life stages for anthropometric growth, we can identify specific life stages during which changes in relative wealth are important for adult health in longitudinal studies. We discuss the assumptions, strengths and limitations of this methodology as applied to relative wealth. We provide an illustrative example using a publicly-available longitudinal dataset and show how relative wealth changes at different life stages are differentially associated with body mass index in adulthood.

**Supplementary Information:**

The online version contains supplementary material available at 10.1186/s12874-022-01757-9.

## Introduction

Wealth is a robust measure of socio-economic position where individuals are vulnerable to economic shocks from unemployment and lack of social safety nets [1]. Income (usually cash income), although easy to measure, may fluctuate in LMIC settings with high rates of informal employment. Consumption expenditure, in the absence of individual (catastrophic expenditures or family events) and systemic (such as COVID-19) shocks, is relatively stable over time as a measure of standard of living since households base their spending on anticipated income [2]. This makes expenditure a useful SEP measure for programmatic targeting. However, expenditure is more difficult to measure compared to asset ownership, requiring lengthy questionnaires on durable and non-durable items, and is prone to measurement error from recall bias, prices for home produce, and seasonality [3]. Asset-based indices are widely used in national, sub-national and community surveys as proxies for household wealth and material wellbeing, due to their relative ease of data collection and computation. Typically, household asset indices are estimated as the first principal component of a dataset comprising of a household’s possession of durables (assumed public goods), housing characteristics, and public utilities [4]. Analysis of national surveys have shown how asset-based indices are associated with health and nutritional status of populations globally [5, 6]. In societies where food expenditures constitute a minority of total expenditure and where households do not experience transitory shocks to expenditure, household asset indices are correlated with non-food expenditures [7, 8]. Asset indices as measures of wealth have several limitations. First, asset indices are usually estimated from an instrument that ascertains possession of a restricted number of contextually relevant assets. Thus, asset indices may not capture non-asset wealth (such as savings or financial instruments) nor the quantity, quality, functioning, availability of substitutes and technological generation of these assets. Second, the distribution of asset indices may not reflect the true distribution of wealth due to issues of clumping (many households having the same value of the index) and truncation (failure to differentiate households at the ends of the distribution) [9]. Third, asset indices may include community infrastructure items that display an urban bias [3, 8, 10–13]. To address this issue, methodologists have suggested creating urban and rural indices separately [1]. Despite these and other criticisms, asset indices are a reliable marker of one’s societal standing in countries where many individuals do not have access to basic goods and services [1, 14].

A longitudinal measure of wealth is useful to describe both individual and population level characteristics of wealth distribution within a population over time. Although both cross-sectional asset indices for birth cohorts as well as harmonized asset indices for serial cross-sectional studies are useful in their own right, a temporally-harmonized asset index allows examination of distributional characteristics beyond these indices (Table 1). The application of the methodology of Filmer and Pritchett to create cross-sectional indices for cohort studies may provide an opportunity to examine redistribution in relative position (change in rank) but cannot capture mean changes in wealth, changes in asset inequality or magnitude of redistribution of relative position [4]. This is because such indices are standardized to zero mean and unit variance, and a unit change may have different interpretations between two cross-sectional study waves due to differences in asset loadings. However, application of methods previously used for serial cross-sectional data to longitudinal data may allow us to study other properties of wealth over time.

**Table 1:** Comparison of approaches and their relative contributions

	**Study type**	**Change in mean wealth**	**Change in individual wealth**	**Change in asset-based inequality**	**Change in individual’s relative position**	**Magnitude of change in individual’s relative position**
Cross-sectional asset index (independent construction)	Serial cross-sectional surveys	No	No	No	No	No
	Cohort	No	No	No	Yes	No
Harmonized asset index (pooled construction)	Serial cross-sectional surveys	Yes	No	Yes	No	No
	Cohort	Yes	Yes	Yes	Yes	Yes

Our objective is to describe the development of conditional wealth, an unexplained residuals modeling framework, to study the importance of changes in relative wealth (mobility) at different life stages with health outcomes in later life [15]. This methodology was previously used to identify life stages that when changes in anthropometric indicators (such as height and weight) were associated with adult health and human capital [16]. We review the methods for developing asset-based indices, define the characteristics of wealth distributions and extend the temporally harmonized index to explain relative wealth mobility over time in cohort studies using conditional measures in later sections. We use an illustrative example from Cebu Longitudinal Health and Nutrition Survey, a birth cohort from Philippines, to show how relative wealth changes at different life stages are differentially associated with body mass index in adulthood [17].

## Cross-sectional and temporally-harmonized asset indices

### Serial cross-sectional studies

Asset indices, as originally developed, can reliably measure one’s relative position at a single point in time, but by nature of their construction can capture neither a change in asset scores nor a change in asset-based inequality in populations. To address these issues, efforts have been made to harmonize asset indices to compare relative wealth between or within countries using cross-sectional data [13, 18]. The International Wealth Index (IWI) is the first component of a PCA applied to data on 12 standard items (seven consumer durables, three housing characteristics, two public utilities) in 165 cross-sectional surveys from 97 countries. The IWI demonstrates an increase in mean wealth over time, and is robust to dropping assets and surveys. The IWI was shown to account for variation in adult female BMI, child height-for-age and infant mortality in a comparative analysis of 300 surveys from 84 countries [19]. The Comparative Wealth Index (CWI) uses as an anchor a reference survey (Vietnam 2002 DHS in the original paper) to compute the relative position of households in space and time [20]. The Absolute Wealth Estimate (AWE) uses the asset index, national GDP per capita and a measure of inequality to compute a household’s income based on their relative position on the asset index [21].

Asset indices created using different dimensionality reduction techniques (PCA, exploratory factor analysis, multiple correspondence analysis, categorical PCA) may vary in their estimate of wealth even after standardization to unit variance. However, these indices are often rank correlated [13], signifying the importance of sensitivity analyses using alternate methodologies while dealing with estimates of small magnitude for association of asset indices with health.

### Longitudinal studies

Previous methodological approaches used to compare cross-sectional studies of populations over time do not permit an exploration of individual wealth trajectories, but could be extended to longitudinal studies. As an example, using five longitudinal birth cohorts, we previously constructed temporally-harmonized indices from a common set of contextually-relevant assets and polychoric PCA, separately for each cohort, using an approach similar to the International Wealth Index [22]. Similar to harmonized indices from serial cross-sectional studies, temporally-harmonized indices allow us to quantify mean gains in wealth and changes in variance of wealth distribution over time. However, temporally-harmonized indices additionally allow us to identify the changes in individual level wealth, magnitude of changes in relative position and relative importance of wealth at different life stages for later-life outcomes. Others have used similar approaches to track material standards of living, and relative distribution of wealth [23–26]. The harmonized indices for the birth cohorts were robust to dropping assets and study waves, were correlated with cross-sectional or region-stratified (by urbanicity) indices, and were rank correlated with indices constructed using alternate dimensionality reduction techniques (PCA, EFA, MCA).

## Changes in wealth, asset inequality and relative position

We define the key characteristics of the wealth distribution for a longitudinal study as follows for an individual ‘i' at two time points ‘t’. First, a positive change in wealth for the population (Eq. 1) and an individual (Eq. 2) are reflected as an increase in the factor score of the temporally-harmonized asset index. This could be either due to an increase in number of assets that load positively or accumulation of higher value assets. Second, an increase in asset-based inequality is equivalent to a positive difference in the relative variance (Eq. 3; fraction of variance attributable to each time point) between study waves of the longitudinal cohort as defined by McKenzie et.al. [27]. Third, the net relative position (Eq. 4) for individuals in the cohort is zero since the cohort is closed. We define the following quantities:

Change in mean wealth:

1 $$\mathrm{E}\left[{\mathrm{W}}_{t=2}\right]-\mathrm{E}[{W}_{t=1}]$$

Change in individual wealth:

2 $${\mathrm{w}}_{\mathrm{i},t=2}-{\mathrm{w}}_{\mathrm{i},t=1}$$

Change in asset-based inequality:

3 $$\sqrt{\frac{\mathrm{Var}[{\mathrm{W}}_{\mathrm{t}=2}]}{\sum {\mathrm{Var}[\mathrm{W}}_{\mathrm{t}=\mathrm{T}}]}}-\sqrt{\frac{\mathrm{Var}[{\mathrm{W}}_{\mathrm{t}=1}]}{\sum {\mathrm{Var}[\mathrm{W}}_{\mathrm{t}=\mathrm{T}}]}}$$

Change in relative position for an individual:

4 $$\mathrm{Rank}\left({\mathrm{w}}_{\mathrm{i},t=2}\right)-\mathrm{Rank}({\mathrm{w}}_{\mathrm{i},t=1})$$

Harmonized asset indices created from serial cross-sectional surveys have demonstrated an increase in mean wealth over time in low- and middle-income countries [9]. Ward uses a temporally harmonized index (constructed using polychoric PCA) with the China Health and Nutrition Survey to show both a consistent increase in mean wealth over time as well as an upward trend in asset-based inequality from 1989 that peaked in 2000 [28]. Inequality in assets subsequently decreased, especially among urban households. Inequality was reported using the methodology proposed by McKenzie using data from Mexico’s national household income and expenditure survey that considered the lack of scale invariance for PCA-derived indices, the China study partitioned the total temporal variance into variance by study wave [27, 28]. A comparative cross-sectional analysis of Demographic and Health Surveys estimated Gini coefficients for asset-based inequality using an index similar to the International Wealth Index [29]. A study using pooled cross-sectional surveys from South Africa identified found that rare assets may distort index estimation and negative loadings on the index (from negative correlation of assets) that may not satisfy axioms of inequality analysis [13]. The authors proposed using an uncentered PCA (UPCA) that does not produce such loadings and inspecting the joint distribution of assets before any substantive analysis.

## Conditional wealth: magnitude of change in an individual’s relative position

### Conditional measures versus adjusting for exposure

An ‘unexplained residuals’ (UR) modeling framework allows examination of the association of several measurements of an exposure, and their relative importance over time, with the outcome [30]. These measures are often operationalized as residuals from a linear regression. However, one could use non-linear approaches to estimate conditional measures while compromising interpretability as residuals absent of correlations with all previous measures of wealth. Equation 5 describes the mathematical quantity of a conditional measure (c_i,t_) for an exposure (w_i,t_) at time t as the difference beyond what is predicted by previous measures.

5 $${\mathrm{c}}_{\mathrm{i,t}}= {\mathrm{w}}_{\mathrm{i,t}}- {\widehat{\mathrm{w}}}_{\mathrm{i,t}} = {\mathrm{w}}_{\mathrm{i,t}}-\mathrm{ f}({\mathrm{w}}_{\mathrm{i,1}},{\mathrm{w}}_{\mathrm{i,2}},{\mathrm{w}}_{\mathrm{i,3}},\dots ,{\mathrm{w}}_{\mathrm{i,t}-1})$$

We demonstrate the use of conditional measures with a simple example using two time points (t = 1, 2) and exposure w_i,t_ measured at time ‘t’ for individual ‘i'. A fixed effects approach for repeated measures of the exposure is provided in Eq. 6. The conditional measures approach is provided in Eq. 7. Previous research has demonstrated how both models are equivalent for w_2_ such that a_2_ = a'_2_ [30]. However, the equality of (a_0_, a'_0_) and (a_1_, a'_1_) depends on the order of entry of variables into the statistical model [31]. This leads to debates (see Limitations of the approach) over the relevance of the anchor measure (w_1_ in our case) that is used as the predictor for other measures (such as w_2_), rendering it different from the usual approach that uses repeated measures of the exposure.

6 $$\mathrm{E}\left[\mathrm{y}\right]= a_{0}+a_{1} {\mathrm{W}}_{1} +a_{2}{\mathrm{W}}_{2}+\mathrm{Covariates}$$

7 $$\mathrm{E}\left[\mathrm{y}\right]=a_{0}^{^{\prime}}+a_{1}^{^{\prime}} {\mathrm{W}}_{1}+a_{2}^{^{\prime}}{\mathrm{C}}_{2}+\mathrm{Covariates}$$

### Conditional growth

Conditional measures have been used to study the relative importance of anthropometric growth during different life stages for adult outcomes. Positive conditional growth implies growth faster than predicted from the population experience and prior measures for that individual. In a model to predict a later life outcome, conditional growth at each life stage has a direct interpretation, since it represents growth during a specific interval [32]. For example, conditional length in first 2 years has been associated with adult height, while conditional weight between 2 and 5 years has been associated with adult BMI [16]. Other studies have shown differential associations of conditional measures of growth with IQ, blood glucose, blood pressure and offspring growth [16, 33–35].

### Conditional wealth

We extend the conditional growth model to a measure derived from asset-based indices, which we call a conditional asset index. We henceforth refer to it as conditional wealth given that asset-based indices are a proxy for wealth in LMICs. Similar to conditional growth, conditional wealth would allow us to identify stages in the life course at which changes in wealth beyond that predicted by past measures of wealth are differentially associated with health outcomes. This is especially important since LMICs are currently experiencing slowing economic growth, high or rising wealth inequality, and intergenerational social persistence. The importance of positional mobility assumes that relative position in the wealth hierarchy matters. This approach could also be used for other measures of material capital such as income and expenditure. However, full discussion of the opportunities and challenges in using these measures to estimate relative mobility is beyond the scope of this manuscript.

Conditional wealth (c_i,t_) for a life stage ‘t’ and individual ‘i’ is the difference in wealth in that life stage from that which could be predicted by all prior individual measures of wealth and the overall wealth trajectory of the population under study (Eq. 8). For our previous example with two time points, which could be extended to more than two time points, where $\mathrm{g}\left({\mathrm{w}}_{\mathrm{i},1}\right)= {\widehat{\mathrm{w}}}_{\mathrm{i},2}$, we propose:

8 $${\mathrm{w}}_{\mathrm{i},2}=g\left({\mathrm{w}}_{\mathrm{i,1}}\right)+{\mathrm{c}}_{\mathrm{i,2}}$$

Conditional wealth is the unexplained residual of the regression of wealth at time 2, as the dependent variable, on all previous measures of wealth (in this case time 1), as linearly associated independent variables (Eq. 9).

9 $${\mathrm{w}}_{\mathrm{i},2}= b_{0} +b_{1} {\mathrm{w}}_{\mathrm{i},1}+{\mathrm{c}}_{\mathrm{i},2}$$

such that

$$\mathrm{E}\left[{\mathrm{c}}_{2}\right]=0;\mathrm{Var}[{\mathrm{c}}_{2}]= {{\sigma }^{2}}_{t=2}$$

Re-writing Eq. 9, we define conditional wealth as the magnitude of change in relative position for an individual: **c**_i,2_ = **w**_i,2_ – [b_0_ + b_1_ **w**_i,1_]. The above example could be extended to more than two time points easily and we demonstrate the same (see Illustrative example).

**Fig. 1:**
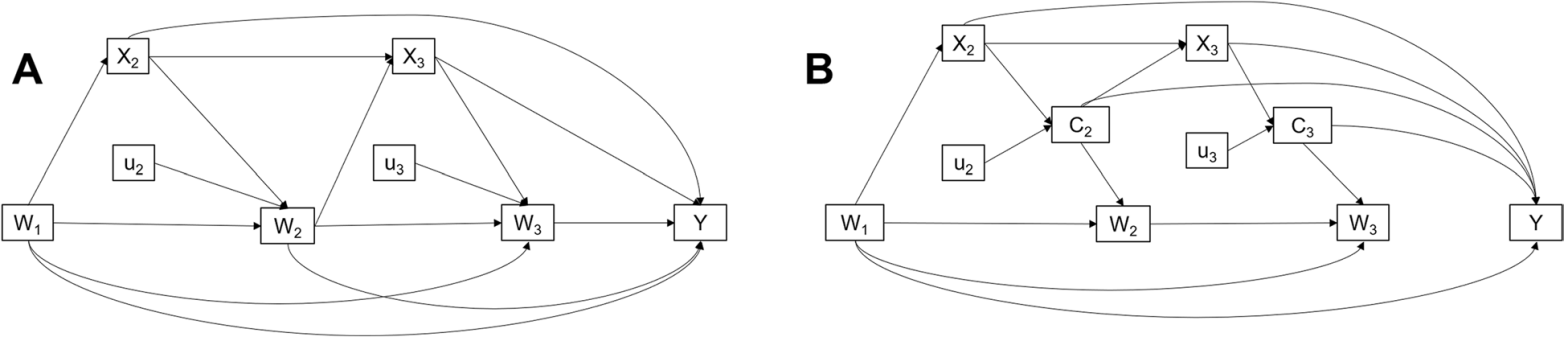
Conceptual framework for wealth and conditional wealth in longitudinal studies W_t_ are the measures of wealth, Y is the health outcome, X_t_ are the covariates associated with wealth like schooling and employment, C_t_ are conditional wealth measures or the magnitude of change in relative position. Panel (**A**) is the traditional framework for study of wealth with Y. In panel (**B**), we conceptualize conditional wealth (C_t_), an extraneous contribution to current wealth beyond past measures of wealth. W_t_ and C_t_ may also be predicted by other unmeasured variables (U_t_) that are not confounders of the wealth-outcome relationship. X_t_ may be predicted by past measures of wealth also

A previous study by Arnold et. al. has clearly demonstrated appropriate confounder adjustment mathematically and using causal diagrams for conditional measures (or unexplained residuals) [30]. Arnold et. al. recommend adjustment for confounders at both stages, i.e. during construction of conditional measures and during estimation of association with outcomes (Eq. 10). The conditional wealth derived using the Arnold et. al. approach would then be uncorrelated with previous measures of wealth and with the confounders [30]. Assume X_1_ is a predictor of wealth at time 1 (e.g. maternal schooling), and X_2_ is a predictor of conditional wealth, and is partly predicted by w_1_ (e.g. attained schooling). A directed acyclic graph (DAG) for how wealth, conditional wealth, schooling and outcome are related is provided in Fig. 1. The outcome regression as per Arnold et. al. (Eq. 11) is fit with the anchor measure, confounders, and conditional wealth.

10 $${\mathrm{w}}_{\mathrm{i},2}=b_{0}^{^{\prime}}+b_{1}^{^{\prime}} {\mathrm{w}}_{\mathrm{i},1}+b_{2}^{^{\prime}}{\mathrm{x}}_{\mathrm{i},1}+b_{3}^{^{\prime}}{\mathrm{x}}_{\mathrm{i},2}+{\mathrm{c}}_{\mathrm{i},2}^{^{\prime}}$$

11 $$\mathrm{E}\left[\mathrm{y}\right]=a_{0}^{\mathrm{^{\prime}}}+a_{1}^{\mathrm{^{\prime}}}{\mathrm{W}}_{1}+a_{2}^{\mathrm{^{\prime}}}{\mathrm{X}}_{1}+a_{3}^{\mathrm{^{\prime}}}{\mathrm{X}}_{2}+a_{3}^{\mathrm{^{\prime}}}{\mathrm{C}}_{2}^{\mathrm{^{\prime}}}$$

We deviate from the approach by Arnold et. al. during the first stage (using Eq. 9) since we are interested in understanding the ‘absorbed effect’ of omitted predictors (Eq. 12) on the conditional measures [30]. In this case, absorbed effect refers to the variability in conditional wealth explained by predictors of conditional measures, where u_i,2_ is the error term when predicting conditional wealth.

12 $${\mathrm{c}}_{\mathrm{i},2}=d_{0}+d_{1}{\mathrm{x}}_{\mathrm{i},1}+d_{2} {\mathrm{x}}_{\mathrm{i},2}+{\mathrm{u}}_{\mathrm{i},2}$$

A path analysis approach (Fig. 1A**)**, where all past measures of the exposure and other covariates are predictors of the exposure at time t, is equivalent to the conditional measures approach (Fig. 1B). Studies estimating the association of conditional wealth with health outcomes should adjust for the first (or anchor) measure of wealth, life course covariates and past measures of conditional wealth, but not for any other wealth measure. Our final outcome regression (Eq. 13) would yield equivalent regression coefficients (i.e. a_1_ = a'_1_, a_2_ = a'_2_ etc.) as Arnold et.al (Eq. 11).

13 $$\mathrm{E}\left[\mathrm{y}\right]=a_{0}+a_{1}{\mathrm{W}}_{1}+a_{2}{\mathrm{X}}_{1}+a_{3}{\mathrm{X}}_{2}+a_{3} {\mathrm{C}}_{2}$$

Given these estimates, our approach is intended to understand how one could estimate the association of conditional wealth with variables that predict it (such as X_1_ and X_2_), while also obtain unbiased estimates of relative position effect on the outcome after appropriate confounder/covariate adjustment.

We also note that studies estimating the association of early life variables on conditional wealth should not adjust for the anchor measure since it is assumed to be uncorrelated with the conditional wealth measure (Supplementary Fig. 1). There is no covariance between the anchor measure and a predictor of conditional wealth (say X_2_) that also covaries with conditional wealth. For example, only the component of attained schooling, say from an intervention such as mandatory schooling, which doesn’t depend on early life wealth predicts conditional wealth. Conditional wealth is, in effect, a decomposition of current wealth into explained and unexplained components that are uncorrelated with each other. Conditional wealth is therefore the magnitude of change in relative position for an individual.

### Conditional wealth versus adjusting for wealth

An advantage of adjusting for conditional wealth over adjusting for wealth at different time points is in ease of interpretability. The coefficient for conditional wealth could be interpreted as the independent contributions of extraneous variations in wealth, resulting in positional mobility, to health disparities. While similar in magnitude, the coefficient for the concurrent wealth variable may alternately be interpreted as the contribution of wealth after adjusting for previous measures of wealth and other covariates.

While reporting the association of conditional wealth and other variables, one should report both (a) the predictors of conditional wealth at a life stage, and (b) the associations of conditional wealth with the health outcome after adjusting for the predictors of conditional wealth that are confounders of conditional wealth and health association. Moreover, one should check if the harmonized wealth measures used to create conditional wealth are suitably distributed continuous variables such that linear regression is an appropriate model formulation.

#### Assumptions for temporally harmonized wealth

We state the assumptions for the temporally harmonized index for individuals or households.

1. Household assets, housing characteristics and public infrastructure items included are public goods, i.e. access by one family member does not prevent the access or availability of others.
2. Household wealth reflects an individual’s standard of living. The harmonized index increases with increase in real and asset-based wealth.
3. Asset loadings on the harmonized index are similar over time, i.e., the relative importance of assets as indicators of household wealth are similar over time.
4. Rankings of households is similar between harmonized index and cross-sectional index for any study wave
5. Criterion validity: Positively associated with other measures of socio-economic position such as schooling, subjective social status and income
6. The harmonized index can distinguish household wealth over the range of the index
7. The harmonized index can distinguish household wealth within the extremes of the distribution (between poor and very poor, between rich and very rich)

The latter two assumptions (clumping and truncation respectively) could be observed from exploratory plots, but are not verifiable due to the absence of a gold-standard marker of wealth.

#### Assumptions for conditional wealth

We state the following assumptions for conditional wealth to be a valid measure of magnitude of change in relative position beyond that predicted by past measures of wealth.

1. *Temporal consistency*: One unit change in conditional wealth should be interpretable in the same scale at different study waves
2. *Appropriate model specification*: The model for creating conditional wealth (as unexplained residuals) is specified correctly, such that residuals are independently and identically distributed, and free of heteroskedasticity.
3. *Appropriate interval selection*: Conditional wealth should have variance as a predictor. Canalization of wealth, i.e., high rank correlation between successive time points, would lead to low variance in conditional wealth.

### Conditional wealth and changes in wealth, asset inequality and relative position

**Fig. 2:**
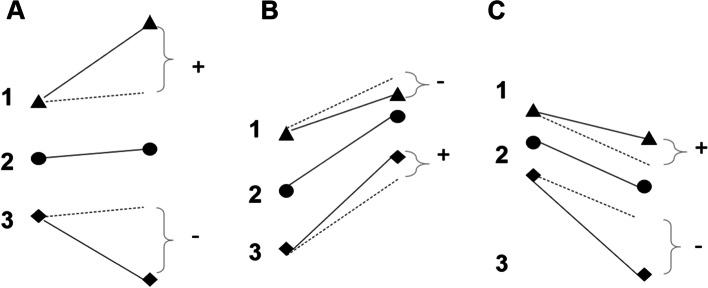
Examples of changes in wealth at two time points for scenarios of mean, variance and relative position The different shapes represent wealth of individuals at two different time points. Additional examples are available in Supplementary File 2

Negative conditional wealth does not necessarily imply that an individual’s wealth decreases. Conversely, positive conditional wealth does not necessarily imply that an individual’s wealth increases. As shown in Fig. 2 (additional examples in Supplementary File 2) for three example individuals at two time points, mean wealth increases in panels A and B, and decreases in panel C. Conditional wealth at time 2 is the vertical distance between the individual trajectory (solid line) and predicted trajectory of each individual based on the cohort (dashed line). For example, in panel A, conditional wealth at time 2 is positive for individual 1, zero for individual 2 and negative for individual 3. In panel B, although the conditional wealth for individual 1 is negative, their overall wealth change is positive due to the large mean change in wealth.

Typically, an individual with positive conditional wealth was more likely than someone with zero or negative conditional wealth to move up the ranks of wealth (see Supplementary File 2, Panels B). However, an important takeaway is that the proportion of variance in wealth at time 2 that is conditional wealth variance explains the extent of positional mobility. Since (w_i,2_ = b_0_ + b_1_ w_i,1_ + c_i,2_), if b_1_ is non-zero, then conditional wealth variance is less than variance of wealth at time 2. If past wealth is unable to explain future wealth (i.e. b_1_ ≈ 0), then positional mobility is high. In a context of rising inequality and no positional mobility, the share of variance unexplained by past wealth may be zero. In such a case, conditional wealth is also zero by virtue of how it is constructed. In such a context, relative social mobility (positional mobility) is non-existent. Associations with health, under a framework where only relative position matters for health, would therefore be entirely explained by past wealth.

In summary, associations of conditional wealth at a time point with an outcome are interpreted as the change in the outcome per a unit of relative wealth mobility, after adjusting for past wealth and other covariates.

## Illustrative example

### Study Population

We used publicly available data from the Cebu Longitudinal Health and Nutrition Survey (CLHNS) – a cohort of women and children conducted in the Philippines [17]. The data were downloaded from the Carolina Population Center Dataverse (https://dataverse.unc.edu/dataverse/cebu). The CLHNS cohort was established with the identification and recruitment of all pregnant women from a single-stage cluster-sample of 17 urban and 16 rural barangays in Metro Cebu in 1983. Among the 3327 women interviewed at baseline, there were 3080 singleton and 26 multiple births, which were followed up during subsequent waves. The harmonized index for CLHNS that we developed included seven publicly available study waves (1983, 1991, 1994, 1998, 2002, 2005, 2009) and data on the 2017–18 wave shared privately with the authors, to be consistent with what was previously reported [22]. However, a sensitivity analysis that did not include the 2017–18 wave showed rank correlation (*r* = 1.00) with the original reported index. We restricted our analysis to singleton births who participated in 2009 (*n* = 1709; age 26–27). The proportion of missingness for wealth was less than 4% in any study wave. Hence, for ease of analysis and interpretation, we restricted our analysis to a complete-case scenario (*n* = 1581). We compared the early life and adult characteristics of those were included and excluded based on complete-case analysis, and found no systematic differences (Supplementary Table 1).

### Variable specification

*Exposure:* Wealth was measured by using questionnaires on assets and housing characteristics (such as building material, toilet, source of water etc.) over the life course. We pooled data on 30 assets (e.g. car, television, house ownership), housing characteristics (e.g. housing material, rooms per resident) and public utilities (e.g. garbage collection) collected during study waves from 1983 to 2018 and created a temporally harmonized asset index, as reported previously [22]. We used a polychoric principal components analysis, extracted the first component and standardized it to unit variance.

We compute the conditional wealth as the residual from a linear regression which predicts wealth at one time point using all previous measures of wealth. We did not adjust for confounders of wealth and outcome association while creating conditional wealth, consistent with the methodology for conditional growth [16].

*Outcome:* Height and weight were measured in 2005 and 2009 respectively. Since adult height stabilizes after age 20, we used height from 2005 to compute body mass index in 2009 as weight (in kg) per square meters.

*Covariates:* Maternal schooling, maternal age, birth order, sex and residence (rural or urban) were collected upon enrollment in 1983. Additionally, every survey collected information on current residence that was classified as urban or rural based on administrative databases. Attained schooling and status of formal employment were collected in adulthood.

### Statistical analysis

We estimated the mean and variance for wealth and conditional wealth at each study wave. We computed change inequality as per equation [3]. We then computed the proportion of variance in wealth that was unexplained, and is an indicator of positional mobility. We first identified the predictors of conditional wealth using multivariable linear regression. Next, we estimate the association of wealth in childhood and conditional wealth at different life stages with body mass index to identify stages of the life course at which changes in relative position were associated with BMI at 26y. We repeated the analysis after stratifying by sex, since patterns of weight status are known to differ by sex in LMICs [36].

All analysis was carried out using R 3.6.1. The code for the analysis is available on https://github.com/jvargh7/conditional-wealth.

### Results

#### Longitudinal trends in wealth

**Fig. 3:**
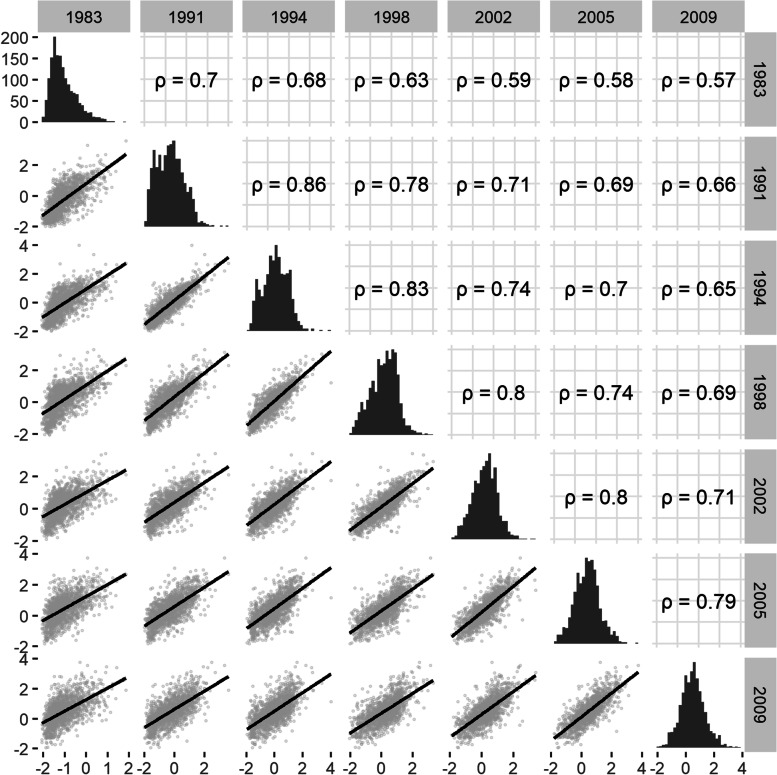
Joint distribution of temporally harmonized wealth at different study waves (*n* = 1581). All correlations reported are Pearson correlation coefficients. Figure created using GGally v2.0.0

Our results suggest that mean wealth increased over time (Table 2). We display the univariate and bivariate distribution of wealth at different study waves in Fig. 3. As expected, correlations between wealth measures decrease as they are further apart in time.

**Table 2:** Summary of harmonized wealth and conditional wealth for Cebu Longitudinal Health and Nutrition Survey 1983–2009 (*n* = 1581)

Year	Wealth	Change in mean wealth	Change in wealth inequality from preceding wave	Conditional wealth	Proportion of variance unexplained
**1983**	-1.04 ± 0.62				
**1991**	-0.24 ± 0.91	0.80	0.13	0 ± 0.65	0.51
**1994**	-0.05 ± 0.89	0.19	-0.01	0 ± 0.44	0.25
**1998**	0.16 ± 0.88	0.22	0.00	0 ± 0.47	0.29
**2002**	0.25 ± 0.79	0.08	-0.04	0 ± 0.45	0.33
**2005**	0.44 ± 0.83	0.20	0.02	0 ± 0.47	0.32
**2009**	0.49 ± 0.85	0.04	0.00	0 ± 0.49	0.34

Values are in mean ± standard deviation for harmonized wealth and conditional wealth (units same as harmonized wealth)

#### Conditional wealth

Conditional wealth measures at the different study waves were normally distributed, consistent with the assumptions of polychoric Principal Component Analysis (Supplementary Fig. 2). Inequality increased from 1983 to 1991 but then remained steady. The share of variance explained by conditional wealth (or unexplained variance in wealth) was 51% in 1991 and decreased to nearly 30% afterwards.

#### Association of conditional wealth measures with body mass index

We noted previously that adjusting for past measures of wealth, the coefficient for absolute wealth and conditional wealth for a single time point are similar in magnitude. As an example, we demonstrate the implications of varying adjustment for wealth and conditional wealth on regression coefficients in Supplementary Table 2.

We present the results for association of early life and life course conditional wealth with body mass index at age 29 y in Supplementary Table 3. One unit increase in wealth in 1983 (age 0 y) was associated with an increase of 0.40 kg/m^2^ (95%CI: -0.02, 0.82) in adult BMI. A unit of relative wealth mobility in 1991 (age 8 y) was associated with 0.36 kg/m^2^ (0.04, 0.68) increase in BMI. A unit of relative wealth mobility in 2009 (age 26) was associated with 0.43 kg/m^2^ (0.04, 0.82) higher BMI. Relative wealth mobility at other ages (age 11 (1994), age 15 (1998) and age 18 (2002)) were not associated with BMI. Maternal schooling was positively associated with conditional wealth in early life, while residing in a rural area in the corresponding study wave was negatively associated (Table 3). Attained schooling (measured in 2002) and formal employment were positively associated with conditional wealth in adulthood.

**Table 3:** Predictors of conditional wealth for Cebu Longitudinal Health and Nutrition Survey 1983–2009 (*n* = 1581)

Year	1991	1994	1998	2002	2005	2009
**Maternal schooling (y)**	0.04 (0.03, 0.05)	0.01 (0, 0.01)	0.01 (0, 0.02)	0 (-0.01, 0.01)	0.01 (0, 0.02)	0 (-0.01, 0.01)
**Maternal age (y)**	0 (-0.01, 0.01)	-0.01 (-0.01, 0)	0 (-0.01, 0)	0 (-0.01, 0)	0 (-0.01, 0)	0 (0, 0.01)
**Birth order**	0.02 (-0.02, 0.05)	0.03 (0.01, 0.06)	0.02 (-0.01, 0.04)	0.01 (-0.01, 0.04)	0.01 (-0.02, 0.04)	-0.02 (-0.04, 0.01)
**Male**	0.02 (-0.04, 0.08)	-0.01 (-0.05, 0.03)	0.09 (0.04, 0.13)	-0.07 (-0.11, -0.02)	0 (-0.04, 0.05)	-0.16 (-0.21, -0.11)
**Rural in 1983**	0 (-0.19, 0.19)	-0.13 (-0.26, 0.01)	0.08 (-0.07, 0.22)	0.17 (0.03, 0.3)	0.02 (-0.12, 0.17)	0.02 (-0.13, 0.17)
**Rural in 1991**	-0.20 (-0.39, -0.01)	0.2 (-0.02, 0.43)	-0.24 (-0.48, 0)	-0.26 (-0.49, -0.03)	-0.15 (-0.4, 0.09)	0.15 (-0.09, 0.4)
**Rural in 1994**		-0.19 (-0.38, 0)	0.17 (-0.06, 0.4)	0.24 (0.02, 0.46)	0.08 (-0.15, 0.31)	0.01 (-0.23, 0.24)
**Rural in 1998**			0.03 (-0.14, 0.2)	0.01 (-0.18, 0.21)	0.21 (0, 0.42)	-0.1 (-0.32, 0.11)
**Rural in 2002**				-0.08 (-0.23, 0.08)	0.23 (0.04, 0.42)	0.05 (-0.15, 0.24)
**Attained schooling (y)**				0.02 (0.02, 0.03)	0.02 (0.01, 0.03)	0.02 (0.01, 0.03)
**Rural in 2005**					-0.35 (-0.49, -0.21)	0.14 (-0.02, 0.29)
**Rural in 2009**						-0.20 (-0.31, -0.10)
**Formal employment in 2009**						0.05 (0.01, 0.10)

Values are displayed estimate and 95% confidence interval from multivariable linear regressions with varying precision reported (all independent variables were entered into the regression in their original units)

**Fig. 4:**
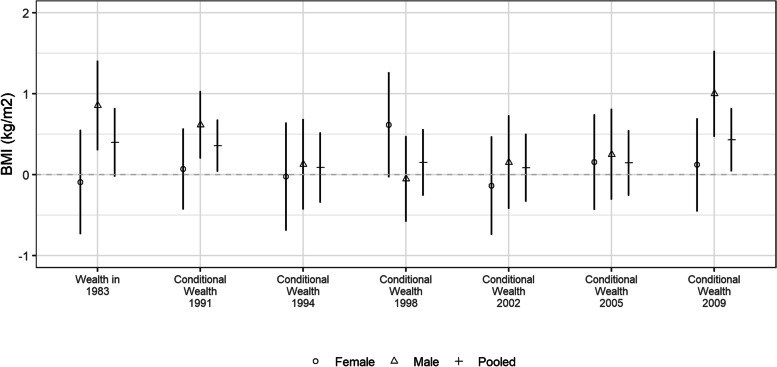
Pooled and sex-stratified association of conditional wealth with body mass index (kg/m^2^) in 2009 for Cebu Longitudinal Health and Nutrition Survey 1983–2009 (*n* = 1503) Values are estimate and 95% confidence interval from linear regression. All measures (wealth in 1983 and conditional wealth) were in the same units as harmonized wealth. We adjusted for maternal schooling, maternal age, birth order, rural residence (1983 to 2009), attained schooling, and formal employment (in 2009). Pregnant women (*n* = 77) were excluded from the analysis. BMI missing for one individual (*n* = 1). Coefficients for all variables are reported in Supplementary Table 3

Our stratified analysis by sex suggest differences in association of body mass index with wealth and conditional wealth at different life stages suggest in 1983, 1991, 1998 and 2009, although we did not formally explore this (Fig. 4, Supplementary Table 3). For example, wealth in 1983 (age 0) and relative wealth mobility in 1991 (age 8) was associated with adult BMI for males but not females. However, relative wealth mobility in 1998 (age 15) was associated with adult BMI for females but not males. We did not conduct hypothesis tests to assess if these sex differences were statistically significant.

### Discussion

Low- and middle-income countries are undergoing a phase of rapid nutrition transition as a result of economic growth and globalization of unhealthy diets. This nutrition transition is accompanied by an obesity transition, where individuals in highest socio-economic position are increasingly overweight while those in lower socioeconomic position are at increased risk of underweight. As countries grow economically, individuals in lower socioeconomic position tend to experience a rise in body mass index. The association of relative wealth mobility and high BMI suggests that individuals who previously were unlikely to consume unhealthy high calorie diets, relative to their peers, may now have access and consume such foods. Other mechanisms include changes in employment type (e.g. manual to non-manual or sedentary occupations) that may increase both relative wealth as well as BMI.

## Limitations of the conditional wealth approach

Firstly, asset-based measures of relative wealth are prone to issues of interference. By virtue of construction of asset-based indices in a study population, an individual’s membership in a high wealth stratum leads to another individual’s membership in a low wealth stratum and consequently the others’ health outcomes [37, 38].

Second, studying SEP (as a function of material, human and social capital) or wealth (as a latent construct measured through the asset index) leads to issues of ‘compound treatments with multiple versions’, i.e. an exposure that comprises of more than one exposure (in this case, combinations of assets and housing characteristics). The asset index comprises of a data-derived weighted (linear) composite of items such as durable goods, housing characteristics and infrastructure. When calculating the asset index, an individual could arrive at the same score through different combinations of items (perfect substitution) [39]. This is a possible violation of the consistency assumption in causal inference, such that individuals who receive an exposure in the real world display the same outcome as they would have if they receive the exposure in the counterfactual world [40, 41]. Furthermore, the items comprising the index could be associated with the health outcome through mechanisms other than the latent construct signified by the index. As an example, possessing a television could be associated with social capital (such as during group viewing of sports or peer-interaction during play hours) or information (versus other media such as radio or internet). Another example is flooring or water source, which determine exposure to infections. In our analysis, we assume treatment-variation irrelevance wherein the same associations are observed independent of which set of items contribute to a participant’s asset index score. Given the possible alternate mechanisms through which the assets might operate jointly (or independently) and knowing that they are not well characterized, it might not be possible to develop interventions from our analysis in practice.

Third, similar to the temporally harmonized index, we do not account for individuals selecting themselves into higher wealth because of increased availability of infrastructure items through migration (from rural to urban areas for employment). We also do not account for changes in household composition (through marriage or separation) that may result in higher (or lower) scores than that of their original birth household [22].

Fourth, a limitation of conditional wealth that is not present in conditional growth is the possibility of treatment-confounder feedback [41]. Early life wealth and conditional wealth may predict confounders of the association (such as attained schooling) between later life conditional wealth and health outcomes. This may bias the association of early life measures with health outcomes.

Fifth, as opposed to an estimation of the association between life course wealth measures and health using outcome regression, we adopt a two-step approach that first estimates conditional wealth, and then estimates the association of the anchor measure and conditional wealth with health, after adjusting for confounders. Further research into the possibility for incorrect standard error estimation, as is the case with manual two-stage estimation for instrumental variable analysis, ought to be considered.

Finally, coefficients for conditional measures cannot be interpreted independent of the anchor (usually first) measure that was used. Our model assumes that one’s absolute wealth at different time points is not independently associated with health, and all such associations are explained by one’s starting position and changes in relative position. Conditional measures estimate the role of relative wealth mobility conditional on the anchor measure. The coefficient for the anchor measure would be higher if positively correlated with the outcome or lower if negatively correlated with the outcome compared to a model where wealth measures at different time points are entered into the model. This is due to the anchor measure capturing the association of changes in mean wealth with the outcome, while conditional measures capture association of changes in relative wealth with the outcome. Consequently, it may falsely suggest designing only interventions that target only the anchor measure as opposed to measures at other time points. For example, in a context of rising inequality and low positional mobility (or high social persistence) irrespective of changes in mean wealth, the anchor measure entirely explains the association of relative position with health. We could also conceptualize an alternate formulation of conditional wealth with the anchor measure as the last measurement. This may answer a different set of questions such as the relative importance of prior relative wealth mobility, beyond current wealth, for a health outcome [32].

### Conclusion

Conditional measures are a useful statistical decomposition of exposures measured longitudinally. Beyond previous established applications such as identifying periods of growth that are associated with health and human capital, applying this technique to harmonized wealth measures may help identify life stages where changes in relative wealth are associated with health. The nature of conditional wealth implies that longitudinal data at an individual or household level are required to construct such measures. Studies such as the Young Lives cohorts, Millennium Villages Project, WHO SAGE surveys, international partner studies of Health and Retirement Surveys and other cohorts or panel studies from LMICs that have collected data on asset-based wealth are sources of such data. Although results with and without decomposing wealth into conditional measures are the same, provided appropriate statistical adjustment, the additional utility of identifying predictors of relative wealth changes at different life stages is important from a public health perspective.

## Supplementary Information

**Additional file 1.** Conditional wealth to estimate association of wealth mobility with health and human capital in low- and middle-income country cohorts

**Additional file 2.**

## Data Availability

The raw datasets are available at the Carolina Population Center Dataverse: https://dataverse.unc.edu/dataverse/cebu. The code and data for the analysis is available at https://github.com/jvargh7/conditional-wealth.

## Abbreviations

CLHNS: Cebu Longitudinal Health and Nutrition Survey
EFA: Exploratory Factor Analysis
LMIC: Low- and middle-income countries
MCA: Multiple Correspondence Analysis
PCA: Principal Component Analysis
SD: Standard deviation
SEP: Socio-economic position

## References

[1] Poirier MJP, Grépin KA, Grignon M (2019). Approaches and Alternatives to the Wealth Index to Measure Socioeconomic Status Using Survey Data: A Critical Interpretive Synthesis. Soc Indic Res.

[2] International Labour Organization. Report II: Household income and expenditure statistics. Geneva: International Labour Organization; 2003. Contract No.: ICLS/17/2003/2.

[3] Howe LD, Galobardes B, Matijasevich A, Gordon D, Johnston D, Onwujekwe O (2012). Measuring socio-economic position for epidemiological studies in low- and middle-income countries: a methods of measurement in epidemiology paper. Int J Epidemiol.

[4] Filmer D, Pritchett LH (2001). Estimating wealth effects without expenditure data–or tears: an application to educational enrollments in states of India. Demography.

[5] Vollmer S, Harttgen K, Subramanyam MA, Finlay J, Klasen S, Subramanian SV (2014). Association between economic growth and early childhood undernutrition: evidence from 121 Demographic and Health Surveys from 36 low-income and middle-income countries. Lancet Glob Health.

[6] Kim R, Kawachi I, Coull BA, Subramanian SV (2018). Contribution of socioeconomic factors to the variation in body-mass index in 58 low-income and middle-income countries: an econometric analysis of multilevel data. Lancet Glob Health.

[7] Howe LD, Hargreaves JR, Gabrysch S, Huttly SR (2009). Is the wealth index a proxy for consumption expenditure? A systematic review. J Epidemiol Community Health.

[8] Filmer D, Scott K (2012). Assessing asset indices. Demography.

[9] Smits J, Steendijk R (2014). The International Wealth Index (IWI). Soc Indic Res.

[10] Hackman J, Hruschka D, Vizireanu M (2020). An Agricultural Wealth Index for Multidimensional Wealth Assessments. Popul Dev Rev.

[11] Bingenheimer JB (2007). Wealth, wealth indices and HIV risk in East Africa. Int Fam Plan Perspect.

[12] Rutstein SO. The DHS Wealth Index: Approaches for Rural and Urban Areas. Calverton, Maryland, USA: Macro International; 2008.

[13] Wittenberg M, Leibbrandt M (2017). Measuring Inequality by Asset Indices: A General Approach with Application to South Africa. The Review of Income and Wealth.

[14] Traynor A, Raykov T (2013). Household Possessions Indices as Wealth Measures: A Validity Evaluation. Comp Educ Rev.

[15] Varghese JS, Patel SA, Martorell R, Ramirez-Zea M, Stein AD (2021). Relative and absolute wealth mobility since birth in relation to health and human capital in middle adulthood: An analysis of a Guatemalan birth cohort. SSM Popul Health.

[16] Adair LS, Fall CH, Osmond C, Stein AD, Martorell R, Ramirez-Zea M (2013). Associations of linear growth and relative weight gain during early life with adult health and human capital in countries of low and middle income: findings from five birth cohort studies. Lancet.

[17] Adair LS, Popkin BM, Akin JS, Guilkey DK, Gultiano S, Borja J (2011). Cohort profile: the Cebu longitudinal health and nutrition survey. Int J Epidemiol.

[18] Córdova A. Methodological Note: Measuring Relative Wealth using Household Asset Indicators. AmericasBarometer Insights. 2008;6.

[19] Mayfour KW, Hruschka D (2022). Assessing comparative asset-based measures of material wealth as predictors of physical growth and mortality. SSM Popul Health.

[20] Rutstein SO, Staveteig S. Making the Demographic and Health Surveys Wealth Index Comparable. Rockville, Maryland, USA: ICF International; 2013.

[21] Hruschka DJ, Gerkey D, Hadley C (2015). Estimating the absolute wealth of households. Bull World Health Organ.

[22] Varghese JS, Adair LS, Patel SA, Bechayda SA, Bhargava SK, Carba DB (2021). Changes in asset-based wealth across the life course in birth cohorts from five low- and middle-income countries. SSM Popul Health.

[23] Östberg W, Howland O, Mduma J, Brockington D. Tracing Improving Livelihoods in Rural Africa Using Local Measures of Wealth: A Case Study from Central Tanzania, 1991–2016. Land. 2018;7(2).

[24] Kabudula CW, Houle B, Collinson MA, Kahn K, Tollman S, Clark S (2017). Assessing Changes in Household Socioeconomic Status in Rural South Africa, 2001–2013: A Distributional Analysis Using Household Asset Indicators. Soc Indic Res.

[25] Michelson H, Muñiz M, DeRosa K (2013). Measuring Socio-economic Status in the Millennium Villages: The Role of Asset Index Choice. J Dev Stud.

[26] Varghese JS, Maluccio JA, Cunningham SA, Ramirez-Zea M, Stein AD (2021). Development of a temporally harmonized asset index: evidence from across 50 years of follow up of a birth cohort in Guatemala. BMC Med Res Methodol.

[27] McKenzie DJ (2005). Measuring inequality with asset indicators. J Popul Econ.

[28] Ward P (2014). Measuring the Level and Inequality of Wealth: An Application to China. Rev Income Wealth.

[29] van Deurzen I, van Oorschot W, van Ingen E (2014). The link between inequality and population health in low and middle income countries: policy myth or social reality?. PLoS ONE.

[30] Arnold KF, Ellison G, Gadd SC, Textor J, Tennant P, Heppenstall A (2019). Adjustment for time-invariant and time-varying confounders in 'unexplained residuals' models for longitudinal data within a causal framework and associated challenges. Stat Methods Med Res.

[31] Hastie T, Tibshirani R, Friedman J. Linear Methods for Regression. The Elements of Statistical Learning. Springer Series in Statistics2009. p. 43–99.

[32] Osmond C, Fall CHD. Conditional Growth Models: An Exposition and Some Extensions. Disease Modelling and Public Health, Part B. Handbook of Statistics2017. p. 275–300.

[33] Poveda NE, Hartwig FP, Victora CG, Adair LS, Barros FC, Bhargava SK (2021). Patterns of Growth in Childhood in Relation to Adult Schooling Attainment and Intelligence Quotient in 6 Birth Cohorts in Low- and Middle-Income Countries: Evidence from the Consortium of Health-Oriented Research in Transitioning Societies (COHORTS). J Nutr.

[34] Norris SA, Osmond C, Gigante D, Kuzawa CW, Ramakrishnan L, Lee NR (2012). Size at birth, weight gain in infancy and childhood, and adult diabetes risk in five low- or middle-income country birth cohorts. Diabetes Care.

[35] Addo OY, Stein AD, Fall CH, Gigante DP, Guntupalli AM, Horta BL (2013). Maternal height and child growth patterns. J Pediatr.

[36] Jaacks LM, Vandevijvere S, Pan A, McGowan CJ, Wallace C, Imamura F (2019). The obesity transition: stages of the global epidemic. Lancet Diabetes Endocrinol.

[37] Tchetgen Tchetgen EJ, VanderWeele TJ (2012). On causal inference in the presence of interference. Stat Methods Med Res.

[38] Wilkinson RG, Pickett KE (2007). The problems of relative deprivation: why some societies do better than others. Soc Sci Med.

[39] Shimeles A, Ncube M (2015). The Making of the Middle-Class in Africa: Evidence from DHS Data. The Journal of Development Studies.

[40] Hernan MA, VanderWeele TJ (2011). Compound treatments and transportability of causal inference. Epidemiology.

[41] Hernán M, Robins J. Causal Inference: What If: Boca Raton: Chapman & Hall/CRC; 2020.

