# Supplementary material for: Conditional wealth to estimate association of wealth mobility with health and human capital in low- and middle-income country cohorts
Source: BMC Med Res Methodol. 2022 Oct 27;22:279. doi: 10.1186/s12874-022-01757-9 (PMC9607821; doi:10.1186/s12874-022-01757-9)
Supplement: Supplementary file 1 — Additional file 1. Conditional wealth to estimate association of wealth mobility with health and human capital in low- and middle-income country cohorts [file 12874_2022_1757_MOESM1_ESM.docx]

**Supplementary File 1**

Conditional wealth to estimate association of wealth mobility with health and human capital in low- and middle-income country cohorts

**Contents**

[Supplementary Table 1. Comparison of early life characteristics and adult characteristics between those included in analytic sample and those excluded 2](#_Toc93919297)

[Supplementary Table 2. Coefficients with varying adjustment for wealth and conditional wealth (n = 1503) 3](#_Toc93919298)

[Supplementary Table 3. Pooled and sex-stratified association of early life and adult characteristics with body mass index 4](#_Toc93919299)

[Supplementary Fig 1 Example of collider bias from adjusting for current measures of wealth while predicting conditional wealth 6](#_Toc93919300)

[Supplementary Fig 2 Distribution of conditional wealth at different study waves (n = 1581) 7](#_Toc93919301)

# Supplementary Table 1. Comparison of early life characteristics and adult characteristics between those included in analytic sample and those excluded

| **Variable** | **N** | **Original sample (n = 3080)** | **Analytic sample**  **(n = 1581)** | **Did not participate in 2009 (n = 1371)** | **Excluded from complete-case analysis (n = 128)** |
| --- | --- | --- | --- | --- | --- |
| **Maternal schooling (y)** | 3080 | 6 (5,9) | 6 (5,9) | 6 (5,10) | 6 (5,9) |
| **Maternal age (y)** | 3080 | 25 (22,30) | 26 (22,30) | 25 (21,30) | 26 (22,30) |
| **Birth order** | 3080 | 3 (2,4) | 3 (2,4) | 3 (2,4) | 3 (2,4) |
| **Male** | 3080 | 53.0% | 52.0% | 53.9% | 55.5% |
| **Rural in 1983** | 3061 | 23.6% | 27.1% | 19.1% | 27.3% |
| **Rural in 1991** | 2264 | 25.7% | 27.0% | 21.2% | 29.1% |
| **Rural in 1994** | 2186 | 26.9% | 27.6% | 23.8% | 29.6% |
| **Rural in 1998** | 2089 | 28.3% | 28.3% | 26.2% | 36.4% |
| **Rural in 2002** | 2023 | 27.8% | 28.5% | 25.1% | 25.2% |
| **Attained schooling (y)** | 1786 | 11 (10,13) | 11 (10,13) | 11 (9,13) | 10 (7,12) |
| **Rural in 2005** | 1888 | 29.7% | 29.9% | 27.6% | 31.1% |
| **Pregnant in 2009** |  |  | N = 77 | - | N = 3 |
| **Rural in 2009** | 1708 | 30.6% | 30.4% | - | 33.6% |
| **Formal employment in 2009** | 1903 | 44.6% | 46.7% | - | 44.5% |
| **Body mass index in 2009 (kg/m^2^)** | 1885 | 22.2±3.8 | 22.4±3.7 | 21.0±3.6 | 22.2±3.4 |
| **Wealth in 1983** | 3080 | -1.0±0.6 | -1.0±0.6 | -0.9±0.7 | -1.0±0.6 |
| **Wealth in 1991** | 2264 | -0.2±0.9 | -0.2±0.9 | -0.1±0.9 | -0.4±0.9 |
| **Wealth in 1994** | 2186 | <0.1±0.9 | -0.1±0.9 | 0.0±1.0 | -0.2±0.9 |
| **Wealth in 1998** | 2082 | 0.2±0.9 | 0.2±0.9 | 0.3±1.0 | 0.1±0.8 |
| **Wealth in 2002** | 2015 | 0.3±0.8 | 0.2±0.8 | 0.3±0.8 | 0.1±0.8 |
| **Wealth in 2005** | 1886 | 0.5±0.8 | 0.4±0.8 | 0.6±0.9 | 0.3±0.8 |
| **Wealth in 2009** | 1709 | 0.5±0.8 | 0.5±0.8 | - | 0.4±0.8 |

All values are in mean +- standard deviation or median (25^th^ percentile, 75^th^ percentile) or percentage (%)

# Supplementary Table 2. Coefficients with varying adjustment for wealth and conditional wealth (n = 1503)

|  | **Intercept** | **Coefficient for 1983** | **Coefficient for 1991** | **Coefficient for 1994** | **Adjusted R^2^** |
| --- | --- | --- | --- | --- | --- |
| BMI in 2009 ~ Early life covariates up to 1983 | 20.89  (19.88, 21.9) | - | - | - | 0.051 |
| BMI in 2009 ~ Wealth 1983 | 21.35  (20.12, 22.57) | 0.25  (-0.13, 0.64) | - | - | 0.051 |
| BMI in 2009 ~ Wealth 1991 | 21.31  (20.25, 22.37) | - | 0.32  (0.05, 0.58) | - | 0.055 |
| BMI in 2009 ~ Wealth 1994 | 21.16  (20.12, 22.19) | - | - | 0.28  (0.01, 0.55) | 0.053 |
| BMI in 2009 ~ Wealth 1983 + Wealth 1991 | 21.32  (20.09, 22.55) | 0.01  (-0.44, 0.45) | 0.32  (0.01, 0.62) | - | 0.054 |
| BMI in 2009 ~ Wealth 1983 + Conditional 1991 | 21.58  (20.33, 22.83) | 0.33  (-0.07, 0.73) | 0.32  (0.01, 0.62) | - | 0.054 |
| BMI in 2009 ~ Wealth 1983 + Wealth 1991 + Wealth 1994 | 21.3  (20.07, 22.53) | 0  (-0.45, 0.45) | 0.26  (-0.17, 0.69) | 0.08  (-0.35, 0.51) | 0.053 |
| BMI in 2009 ~ Wealth 1983 + Conditional 1991 + Conditional 1994 | 21.58  (20.33, 22.84) | 0.34  (-0.07, 0.74) | 0.32  (0.01, 0.62) | 0.08  (-0.35, 0.51) | 0.053 |
| BMI in 2009 ~ Wealth 1983 + Wealth 1991 + Conditional 1994 | 21.33  (20.1, 22.55) | 0.01  (-0.43, 0.46) | 0.32  (0.01, 0.62) | 0.08  (-0.35, 0.51) | 0.053 |

Values are estimate and 95% confidence interval from linear regression. All measures (wealth in 1983 and conditional wealth) were in the same units as harmonized wealth. We adjusted for maternal schooling, maternal age, birth order, rural residence (up to year in regression). Pregnant women (n = 77) were excluded from the analysis. BMI missing for one individual (n = 1).

# Supplementary Table 3. Pooled and sex-stratified association of early life and adult characteristics with body mass index

| **Variable** | **Pooled (n = 1503)** | **Female (n = 682)** | **Male (n = 821)** |
| --- | --- | --- | --- |
| Male | 1.01  (0.61, 1.4) | - | - |
| Maternal schooling (y) | 0.09  (0.02, 0.17) | 0.08  (-0.03, 0.2) | 0.10  (0, 0.21) |
| Maternal age (y) | 0.04  (0, 0.08) | 0.04  (-0.02, 0.11) | 0.04  (-0.02, 0.09) |
| Birth order | -0.38  (-0.6, -0.16) | -0.44  (-0.76, -0.13) | -0.34  (-0.63, -0.05) |
| Rural in 1983 | 0.4  (-0.8, 1.6) | -0.77  (-2.58, 1.04) | 1.44  (-0.14, 3.02) |
| Rural in 1991 | -0.53  (-2.5, 1.45) | 0.23  (-2.93, 3.4) | -0.67  (-3.16, 1.81) |
| Rural in 1994 | -0.41  (-2.29, 1.47) | -0.12  (-3.14, 2.91) | -0.77  (-3.18, 1.64) |
| Rural in 1998 | -0.62  (-2.34, 1.1) | 0.41  (-2.31, 3.14) | -1.81  (-4.11, 0.49) |
| Rural in 2002 | 0.41  (-1.17, 1.99) | -1.27  (-3.39, 0.86) | 1.94  (-0.45, 4.32) |
| Attained schooling (y) | -0.03  (-0.1, 0.05) | -0.18  (-0.31, -0.05) | 0.05  (-0.05, 0.14) |
| Rural in 2005 | 0.1  (-1.13, 1.32) | -0.27  (-1.99, 1.45) | 0.16  (-1.59, 1.9) |
| Rural in 2009 | 0.4  (-0.47, 1.28) | 1.13  (-0.15, 2.41) | -0.19  (-1.39, 1) |
| Formal employment in 2009 | -0.23  (-0.61, 0.15) | -0.64  (-1.23, -0.06) | 0.4  (-0.09, 0.89) |
| Wealth in 1983 | 0.4  (-0.02, 0.82) | -0.09  (-0.73, 0.55) | 0.86  (0.3, 1.41) |
| Conditional wealth in 1991 | 0.36  (0.04, 0.68) | 0.07  (-0.43, 0.57) | 0.62  (0.2, 1.03) |
| Conditional wealth in 1994 | 0.09  (-0.34, 0.52) | -0.02  (-0.69, 0.65) | 0.13  (-0.43, 0.69) |
| Conditional wealth in 1998 | 0.15  (-0.26, 0.56) | 0.62  (-0.03, 1.27) | -0.05  (-0.58, 0.48) |
| Conditional wealth in 2002 | 0.09  (-0.33, 0.51) | -0.14  (-0.74, 0.47) | 0.16  (-0.42, 0.73) |
| Conditional wealth in 2005 | 0.15  (-0.26, 0.55) | 0.16  (-0.43, 0.74) | 0.25  (-0.31, 0.81) |
| Conditional wealth in 2009 | 0.43  (0.04, 0.82) | 0.12  (-0.45, 0.7) | 1.00  (0.47, 1.53) |

Values are estimate and 95% confidence interval from linear regression. All measures (wealth in 1983 and conditional wealth) were in the same units as harmonized wealth. We adjusted for maternal schooling, maternal age, birth order, rural residence (1983 to 2009), attained schooling, and formal employment (in 2009). Pregnant women (n = 77) were excluded from the analysis. BMI missing for one individual (n = 1).

# Supplementary Fig 1 Example of bias from adjusting for current measures of wealth while predicting conditional wealth

**
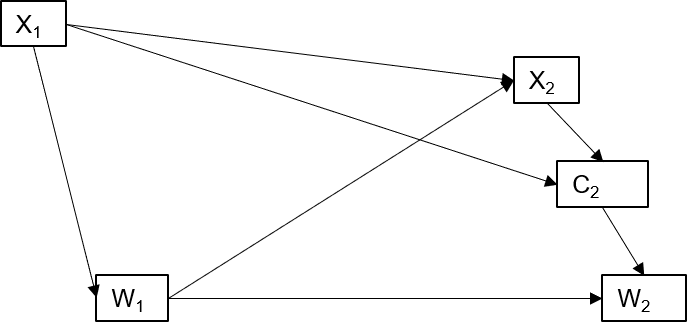
**

X_1_ and X_2_ are covariates associated with wealth (W_1_, W_2_). C_2_ is conditional wealth. Our conditioning set while predicting C_2_ consists of X_1_ and X_2_ only. We define early life wealth (W_1_), maternal schooling (X_1_) and attained schooling (X_2_): X_1_ 🡪 W_1_ 🡪 X_2_. We do not include W_2_ in the conditioning set since W_2_ is a linear combination of W_1_ and C_2_.

# Supplementary Fig 2 Distribution of conditional wealth at different study waves (n = 1581)


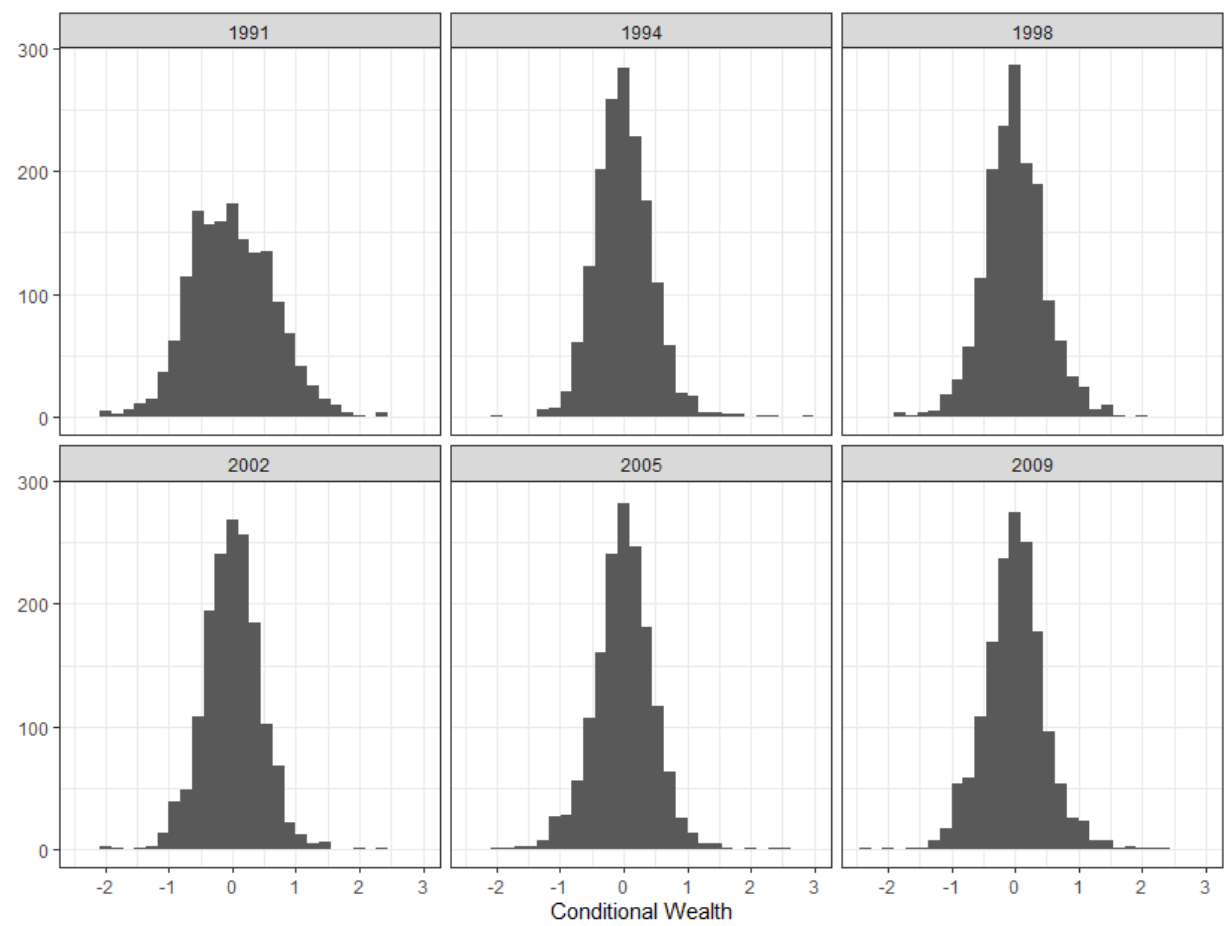


All values are conditional wealth measures derived from complete case analysis of temporally harmonized index.
