# Supplementary material for: Conditional wealth to estimate association of wealth mobility with health and human capital in low- and middle-income country cohorts
Source: BMC Med Res Methodol. 2022 Oct 27;22:279. doi: 10.1186/s12874-022-01757-9 (PMC9607821; doi:10.1186/s12874-022-01757-9)

**S2Fig 1:**
**Example with w_1 = N(0,1)**
**w_2 = −1 + −1*w_1 + N(0,0.2)**
**Ratio of variance at time 2: time 1 = 1.1**

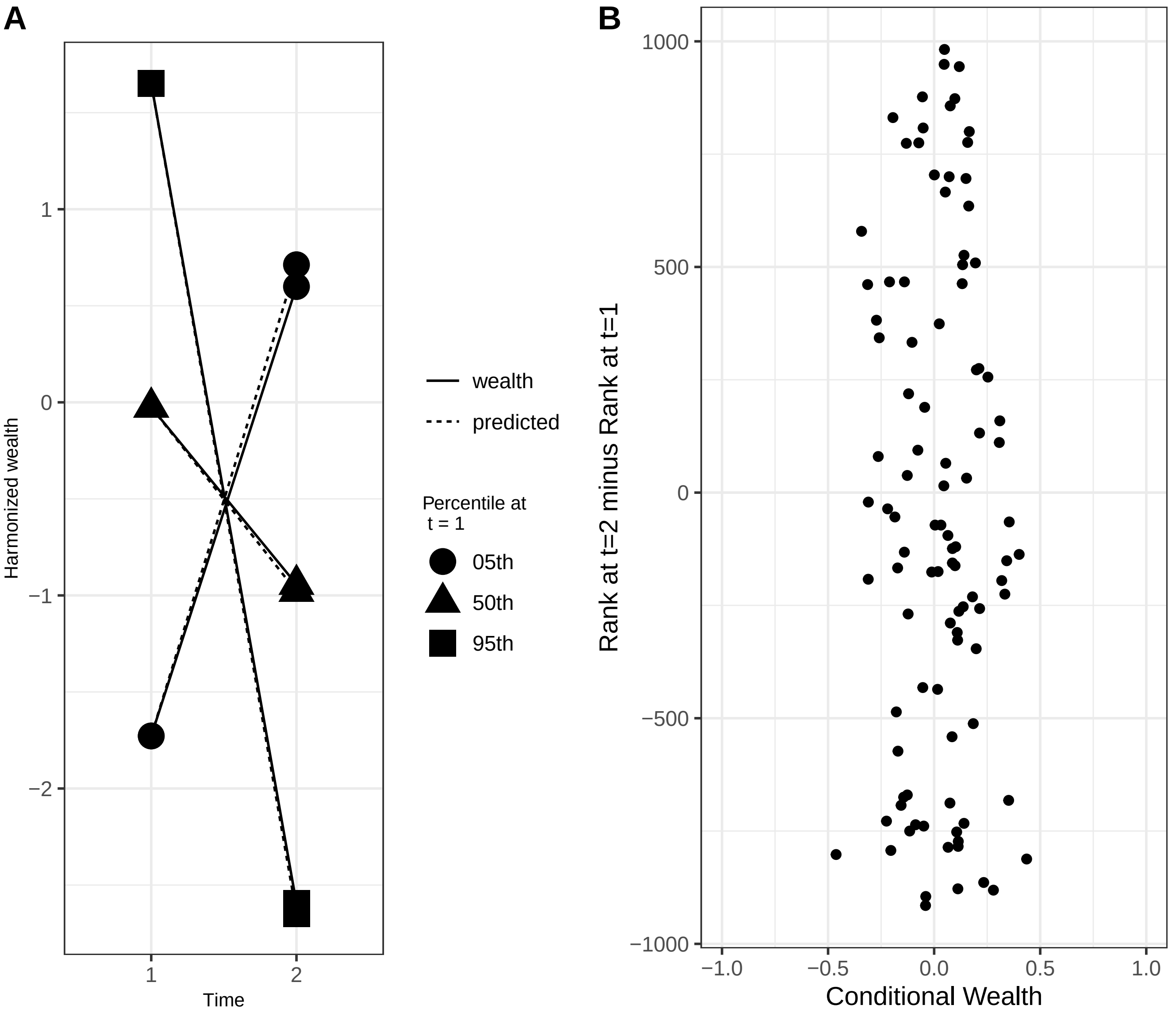

**S2Fig 2:**
**Example with w_1 = N(0,1)**
**w_2 = −0.5 + −1*w_1 + N(0,0.2)**
**Ratio of variance at time 2: time 1 = 1.1**

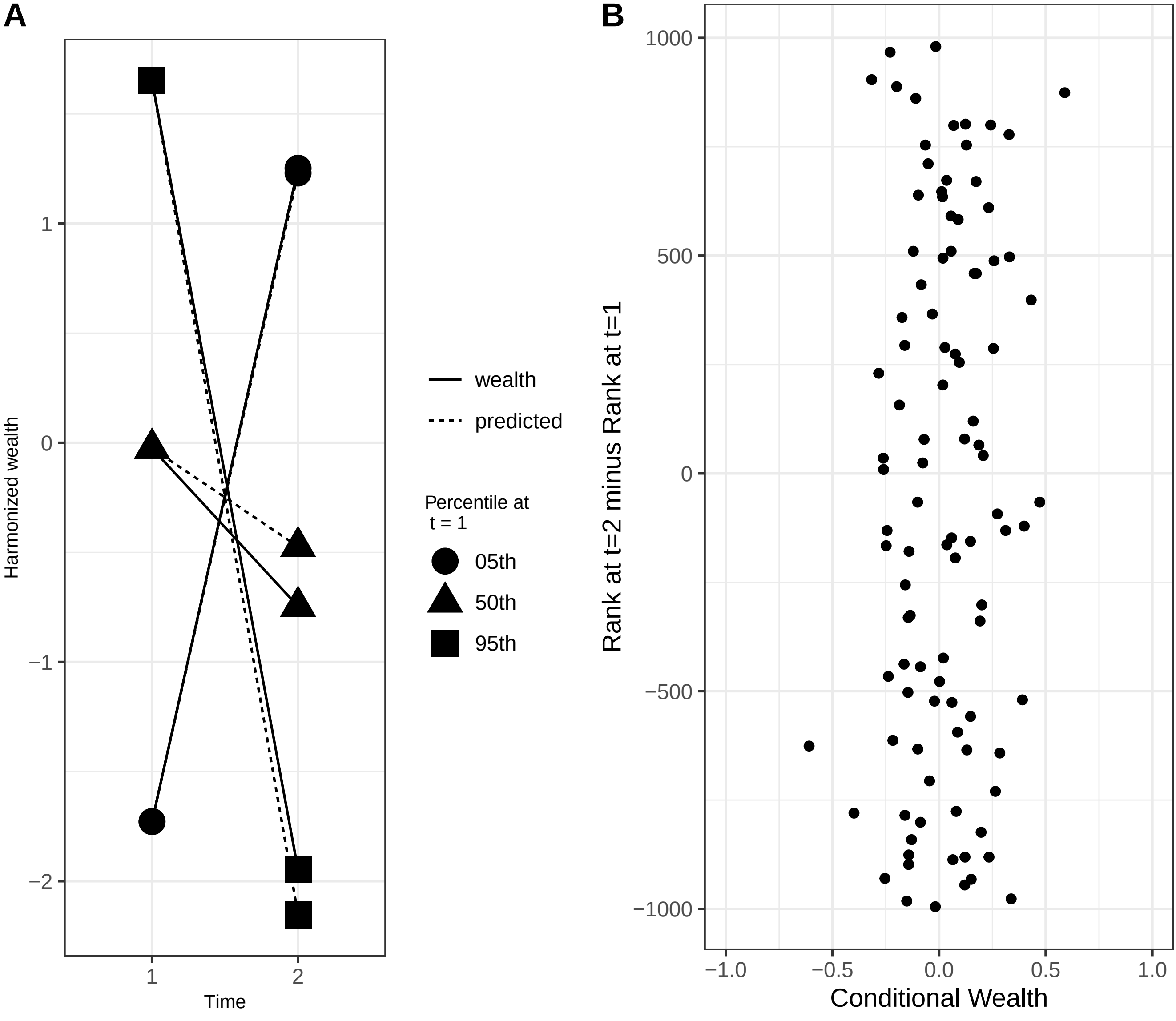

**S2Fig 3:**
**Example with w_1 = N(0,1)**
**w_2 = −0.3 + −1*w_1 + N(0,0.2)**
**Ratio of variance at time 2: time 1 = 1.1**

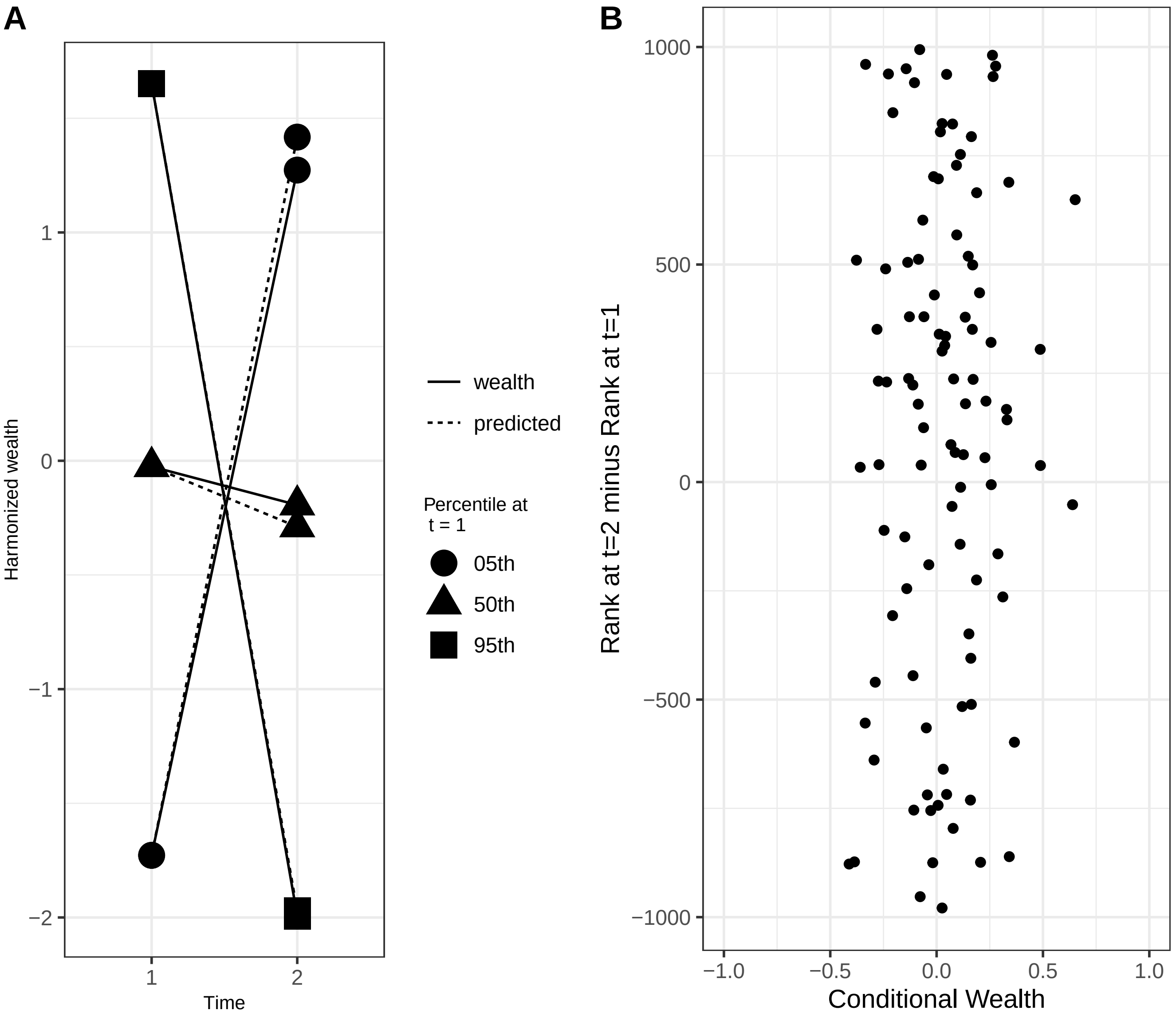

**S2Fig 4:**
**Example with w_1 = N(0,1)**
**w_2 = 0 + −1*w_1 + N(0,0.2)**
**Ratio of variance at time 2: time 1 = 1.1**

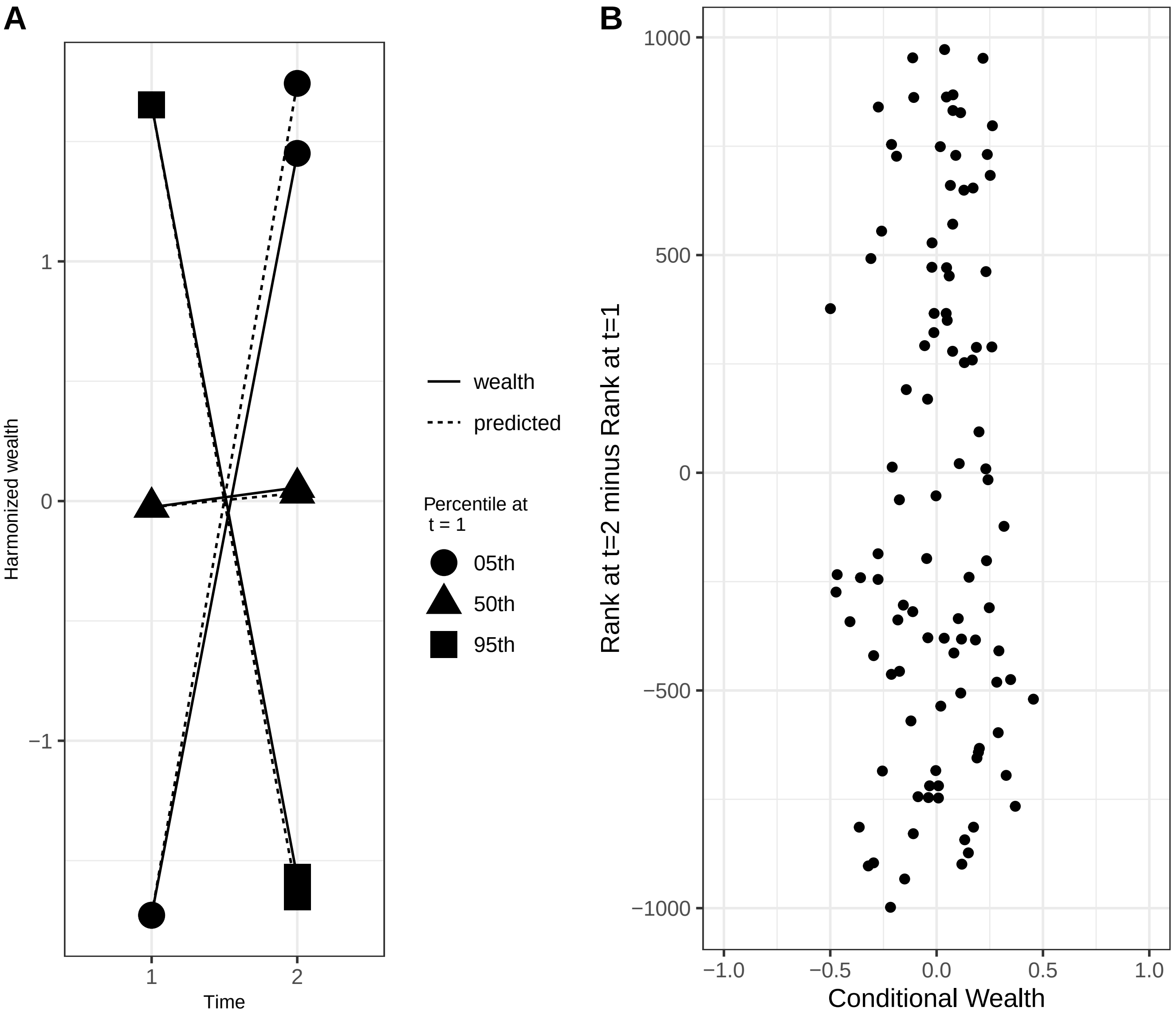

**S2Fig 5:**
**Example with w_1 = N(0,1)**
**w_2 = 0.3 + −1*w_1 + N(0,0.2)**
**Ratio of variance at time 2: time 1 = 1.1**

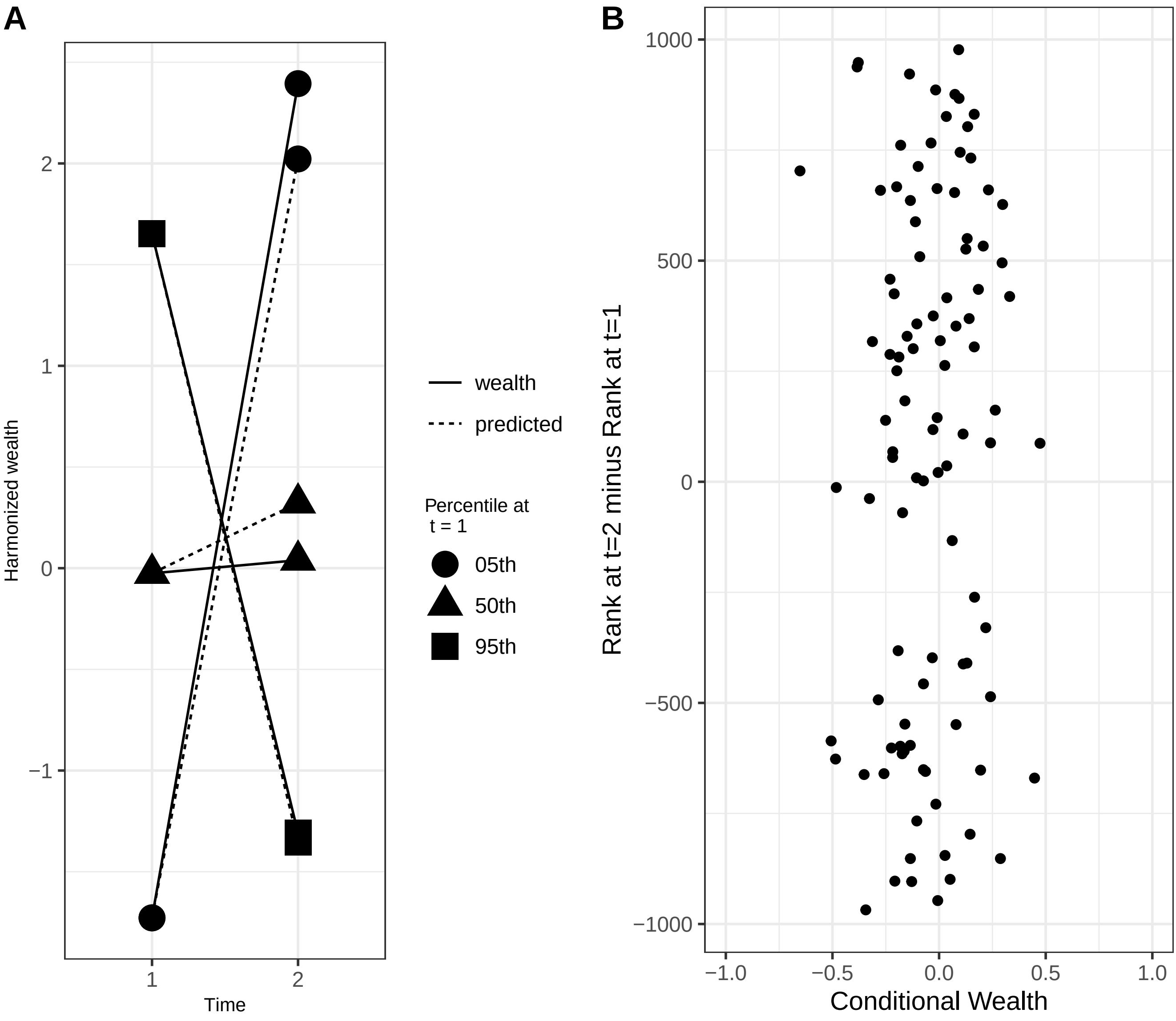

**S2Fig 6:**
**Example with w_1 = N(0,1)**
**w_2 = 0.5 + −1*w_1 + N(0,0.2)**
**Ratio of variance at time 2: time 1 = 1.1**

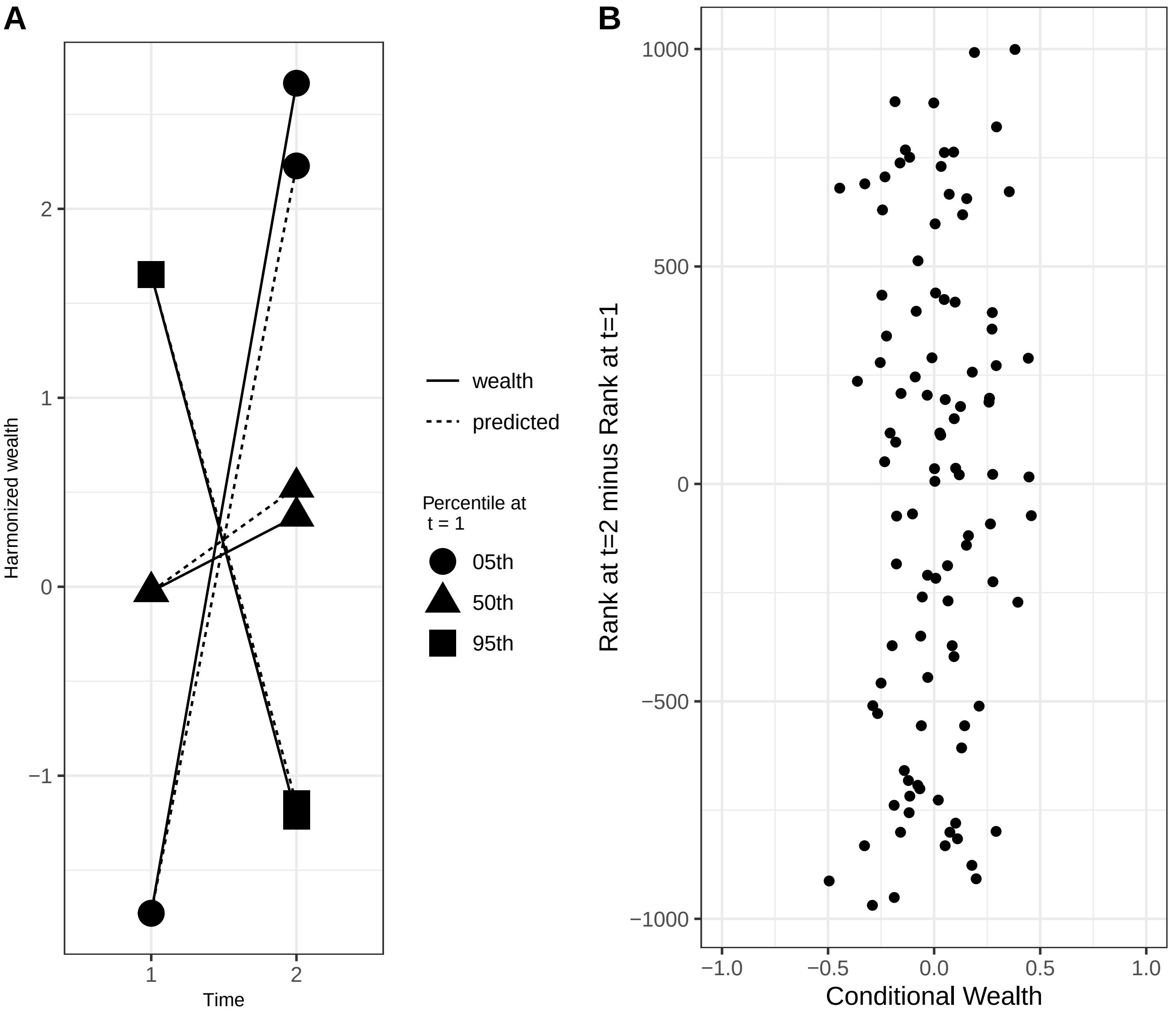

**S2Fig 7:**
**Example with w_1 = N(0,1)**
**w_2 = 1 + −1*w_1 + N(0,0.2)**
**Ratio of variance at time 2: time 1 = 1.1**

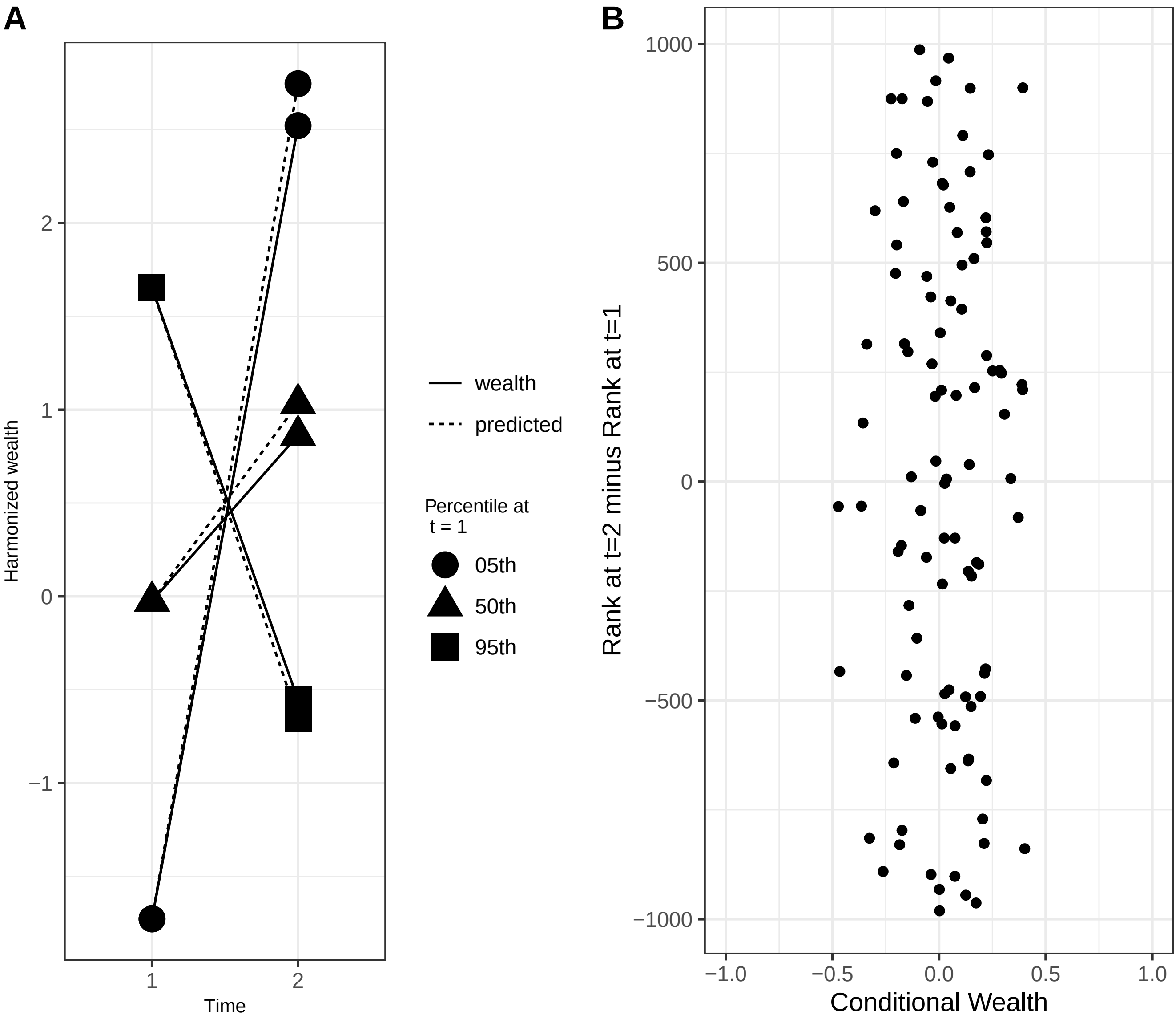

**S2Fig 8:**
**Example with w_1 = N(0,1)**
**w_2 = −1 + −0.5*w_1 + N(0,0.2)**
**Ratio of variance at time 2: time 1 = 0.3**

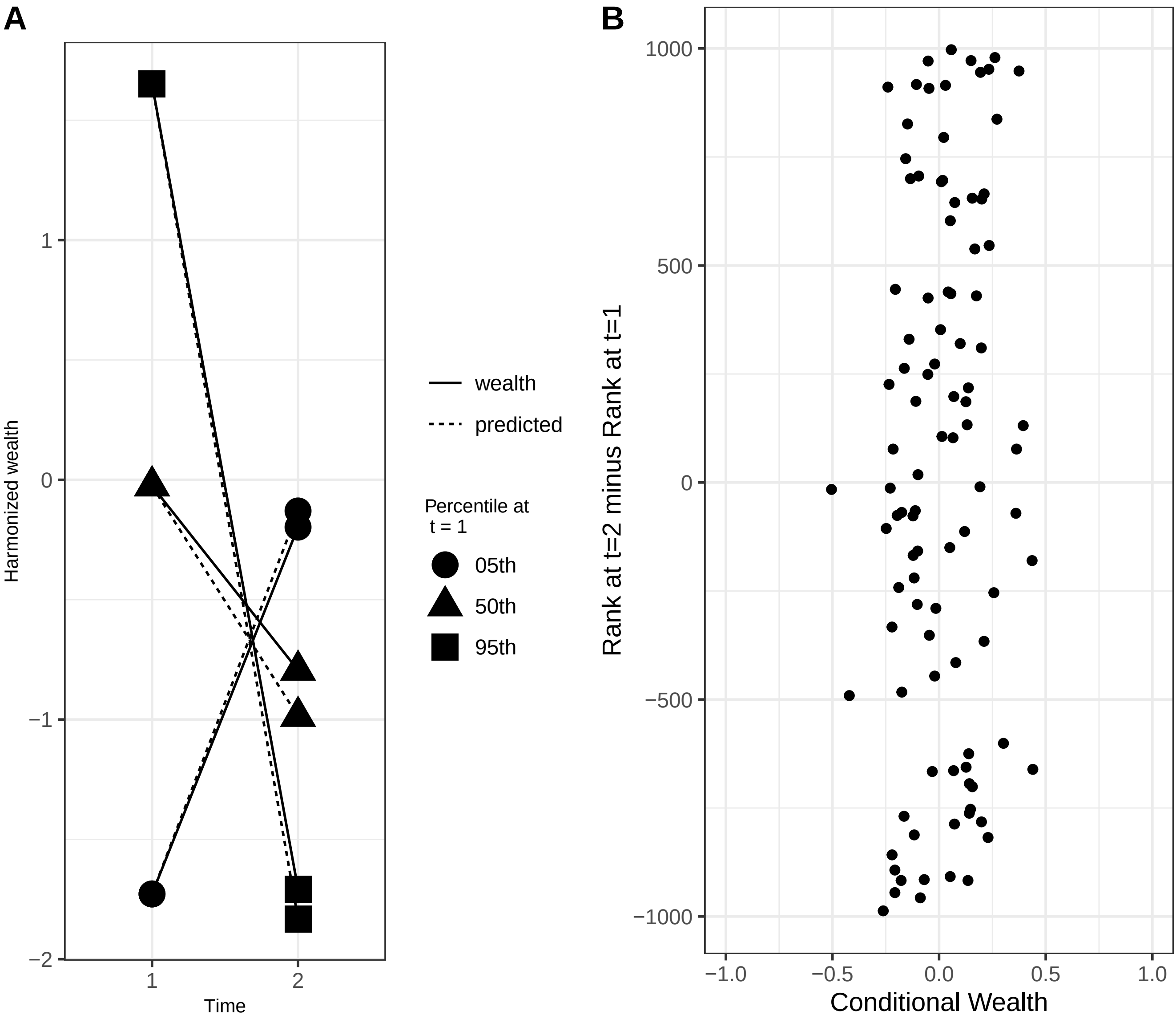

**S2Fig 9:**
**Example with w_1 = N(0,1)**
**w_2 = −0.5 + −0.5*w_1 + N(0,0.2)**
**Ratio of variance at time 2: time 1 = 0.3**

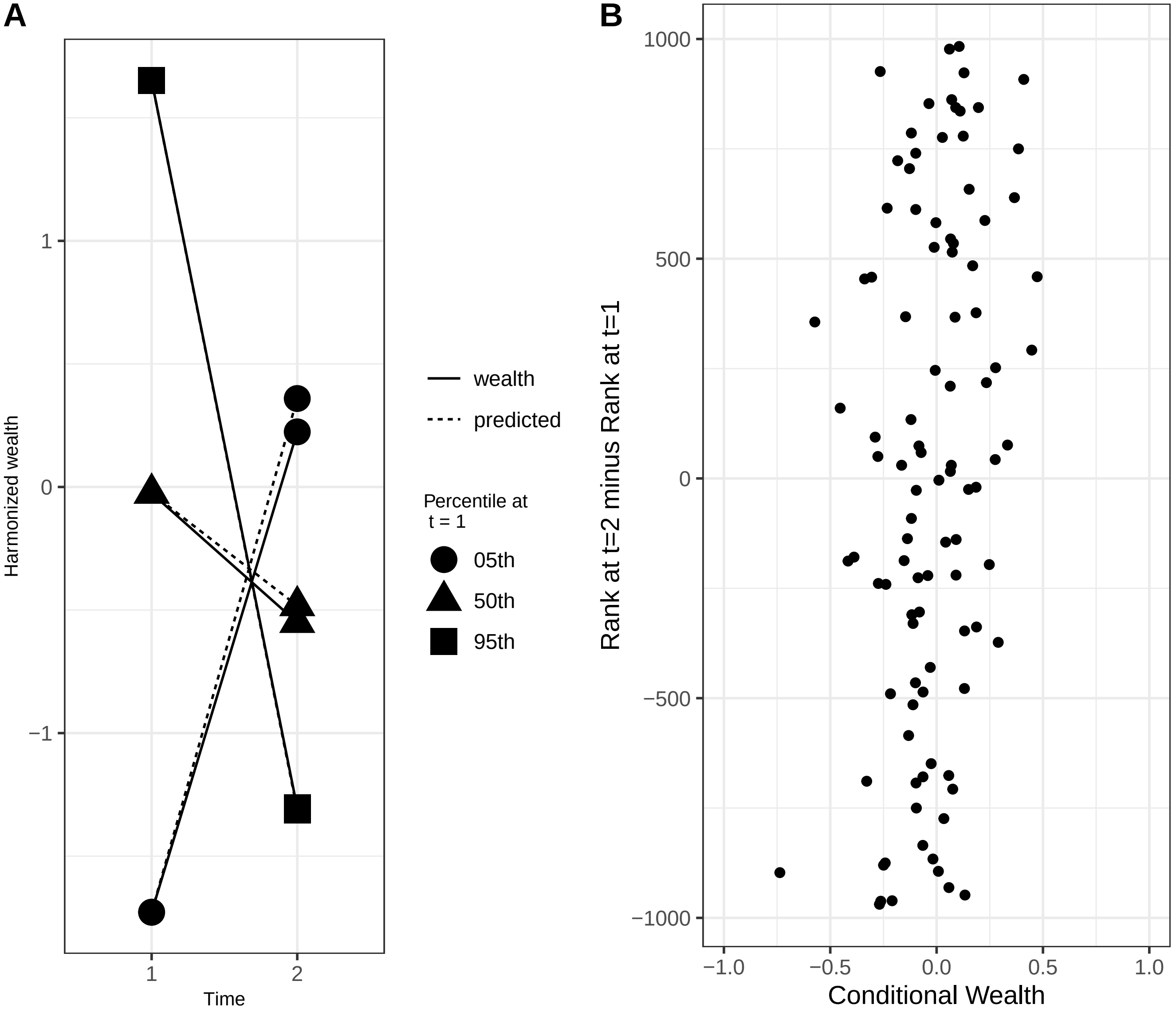

**S2Fig 10:**
**Example with w_1 = N(0,1)**
**w_2 = −0.3 + −0.5*w_1 + N(0,0.2)**
**Ratio of variance at time 2: time 1 = 0.3**

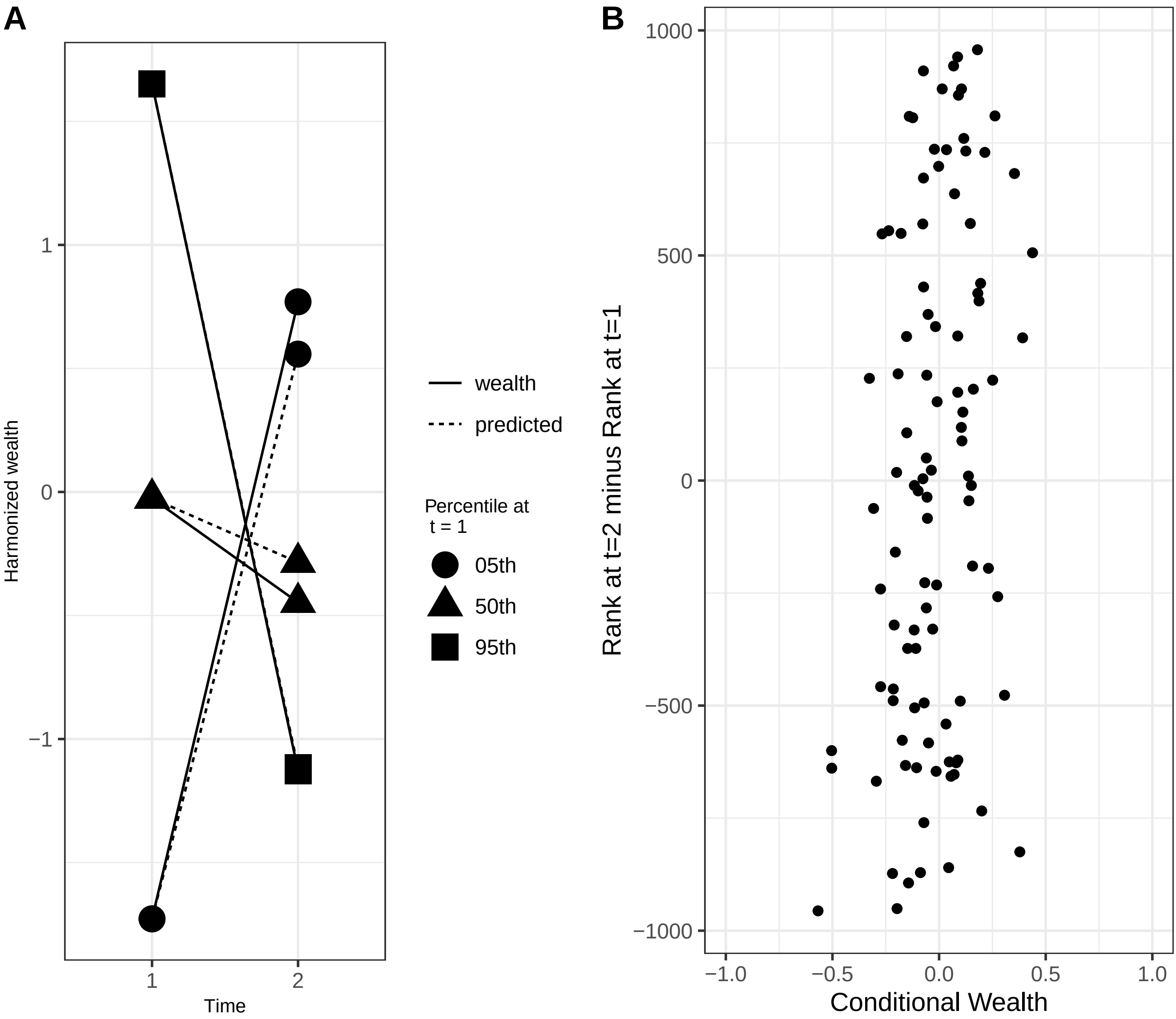

**S2Fig 11:**
**Example with w_1 = N(0,1)**
**w_2 = 0 + −0.5*w_1 + N(0,0.2)**
**Ratio of variance at time 2: time 1 = 0.3**

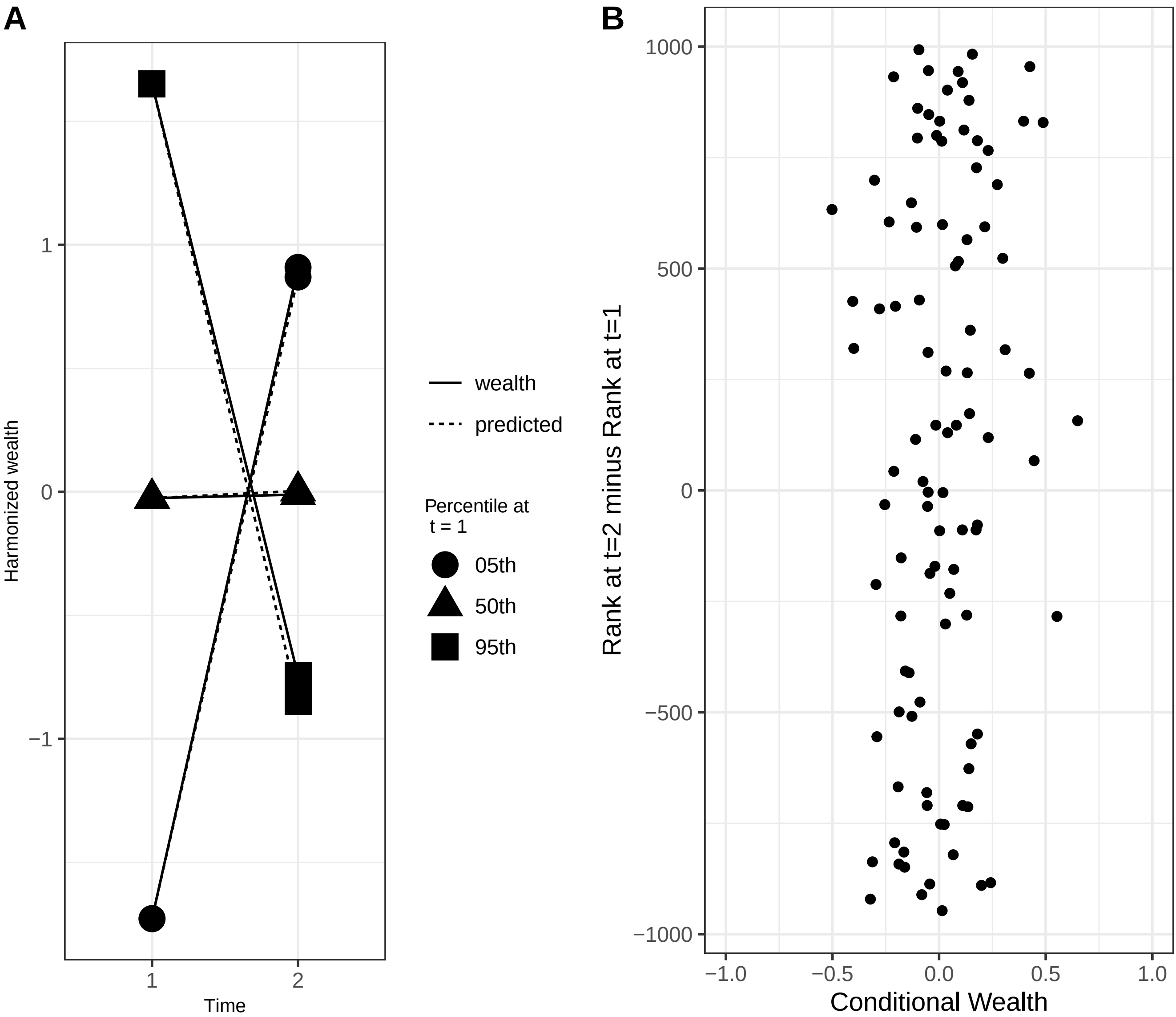

**S2Fig 12:**
**Example with w_1 = N(0,1)**
**w_2 = 0.3 + −0.5*w_1 + N(0,0.2)**
**Ratio of variance at time 2: time 1 = 0.3**

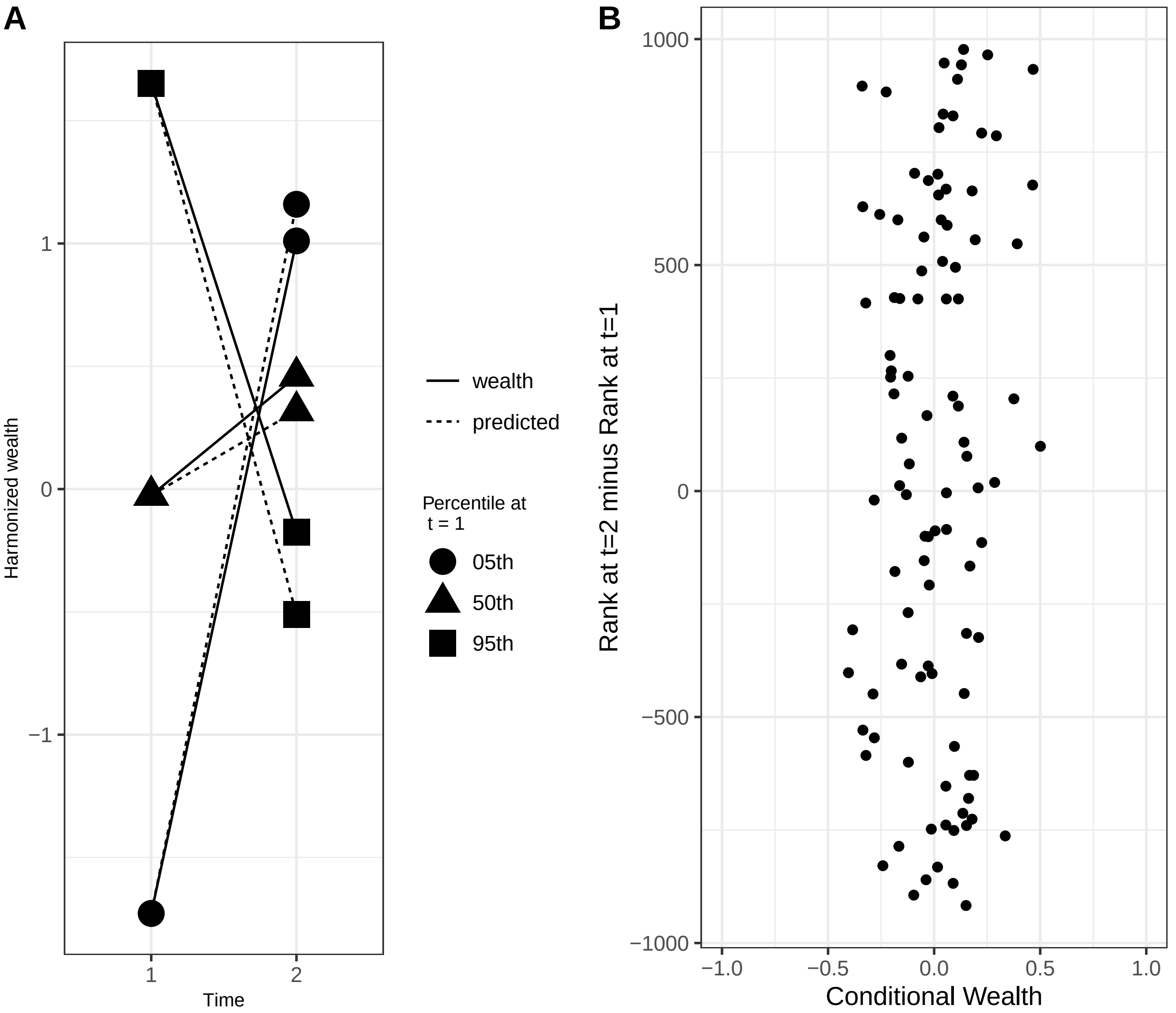

**S2Fig 13:**
**Example with w_1 = N(0,1)**
**w_2 = 0.5 + −0.5*w_1 + N(0,0.2)**
**Ratio of variance at time 2: time 1 = 0.3**

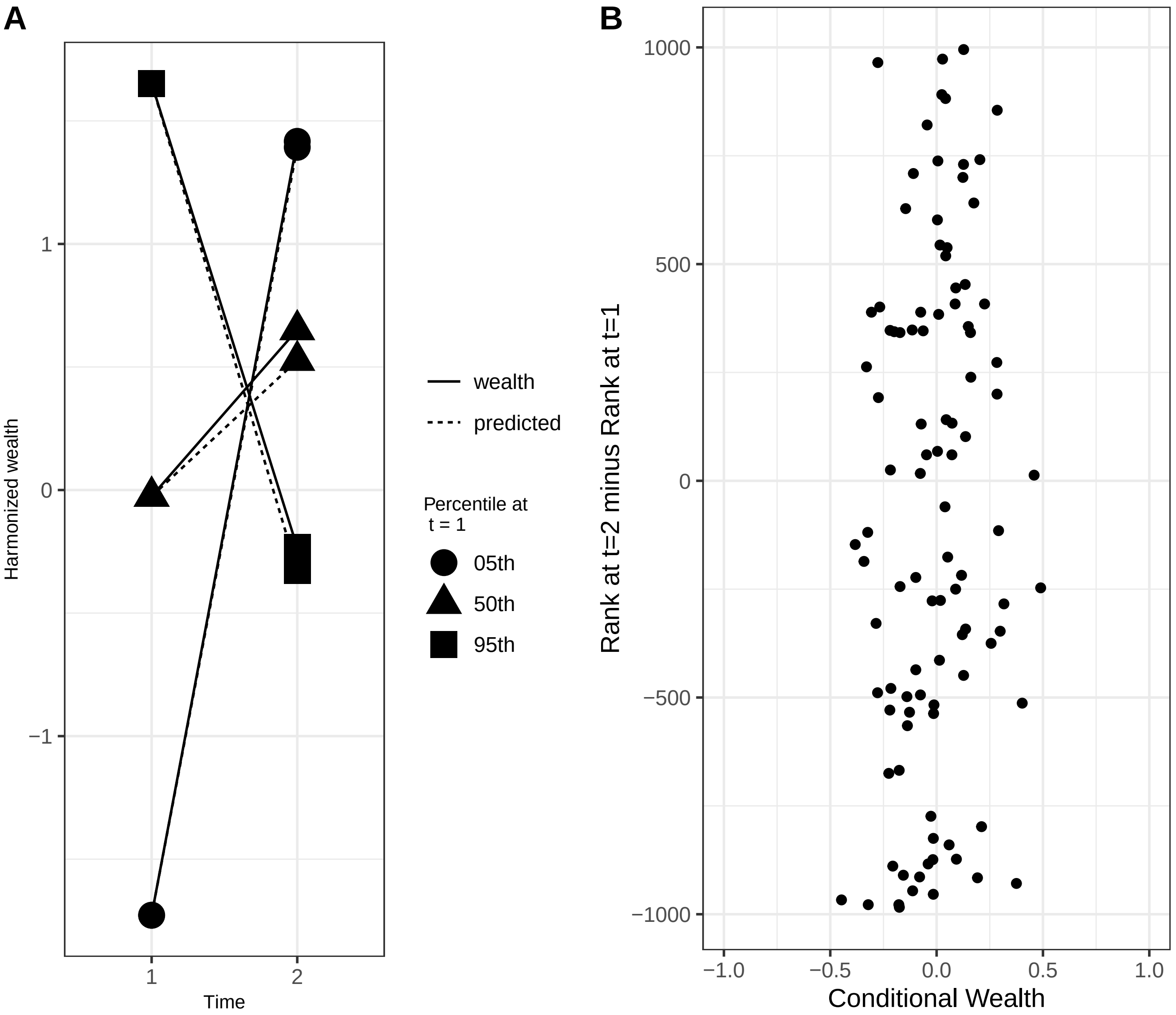

**S2Fig 14:**
**Example with w_1 = N(0,1)**
**w_2 = 1 + −0.5*w_1 + N(0,0.2)**
**Ratio of variance at time 2: time 1 = 0.3**

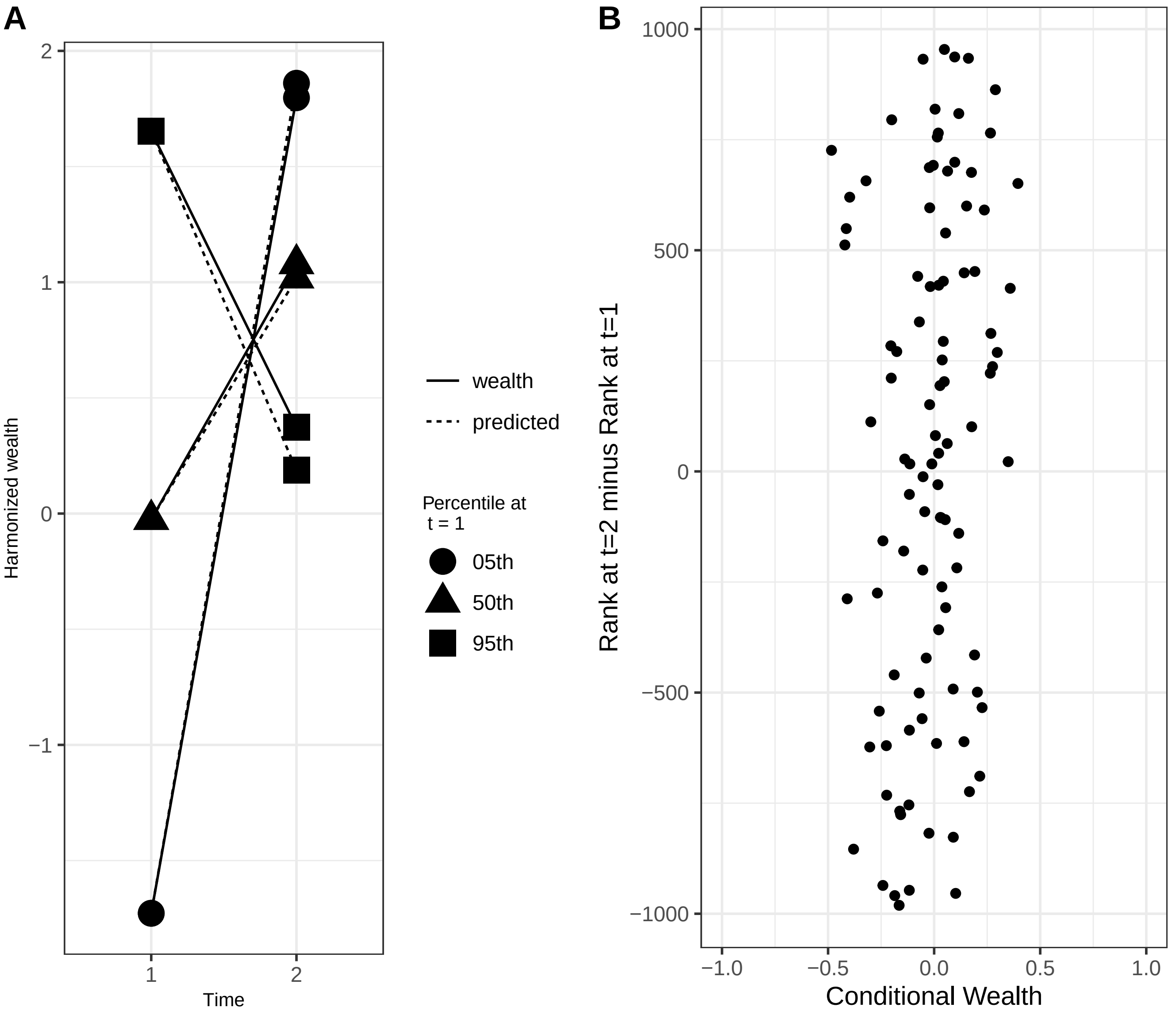

**S2Fig 15:**
**Example with w_1 = N(0,1)**
**w_2 = −1 + 0*w_1 + N(0,0.2)**
**Ratio of variance at time 2: time 1 = 0**

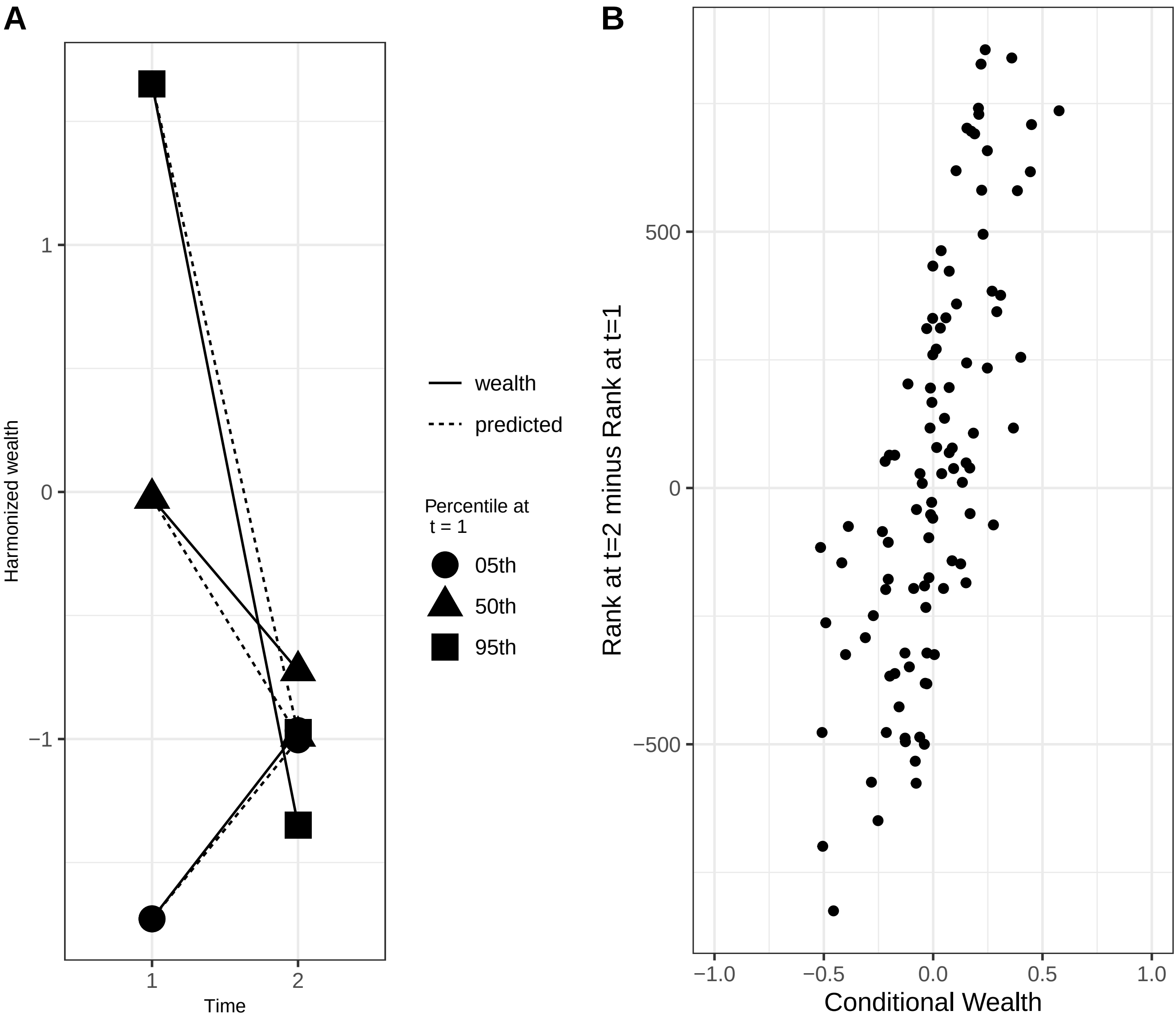

**S2Fig 16:**
**Example with w_1 = N(0,1)**
**w_2 = −0.5 + 0*w_1 + N(0,0.2)**
**Ratio of variance at time 2: time 1 = 0**

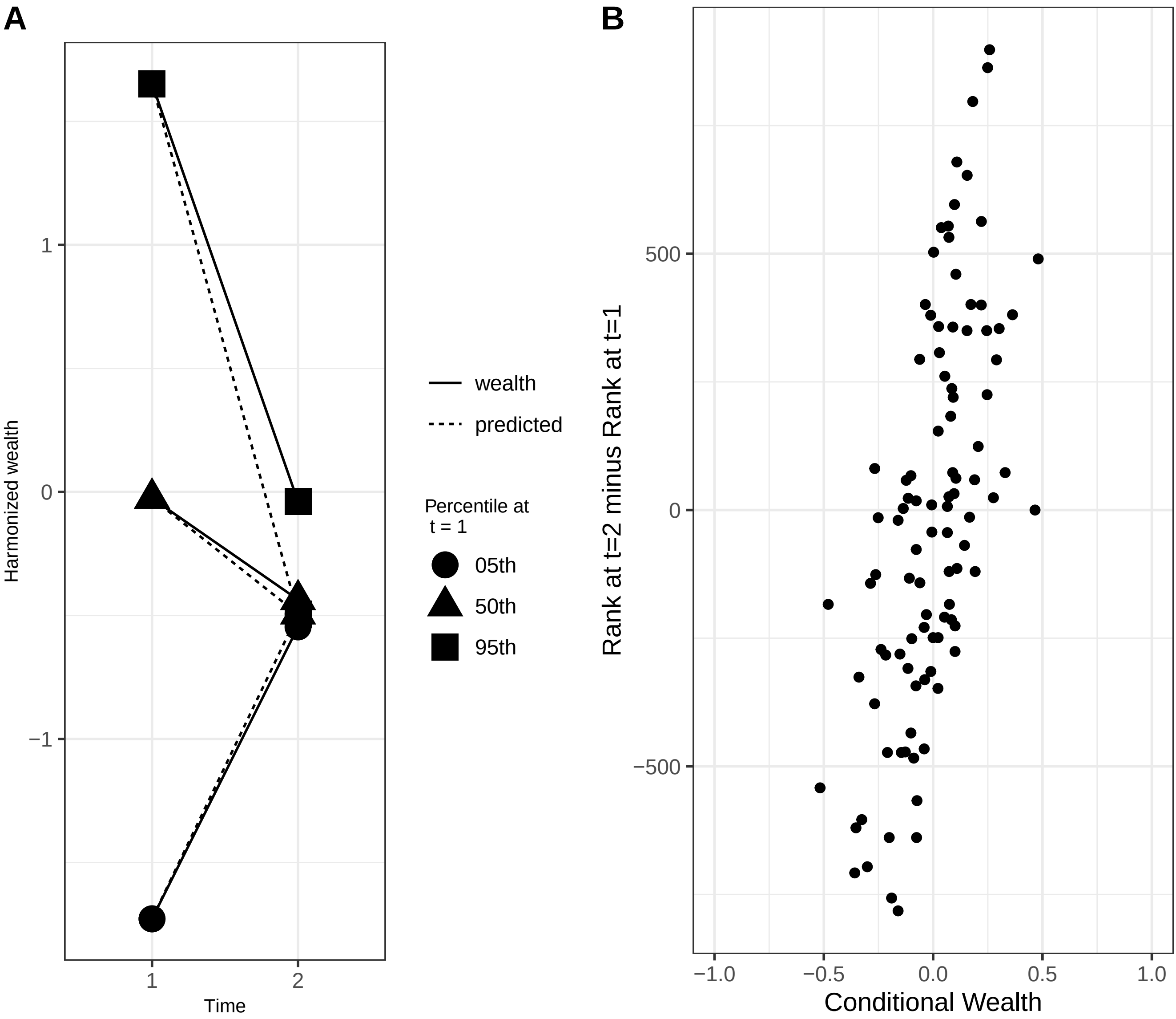

**S2Fig 17:**
**Example with w_1 = N(0,1)**
**w_2 = −0.3 + 0*w_1 + N(0,0.2)**
**Ratio of variance at time 2: time 1 = 0**

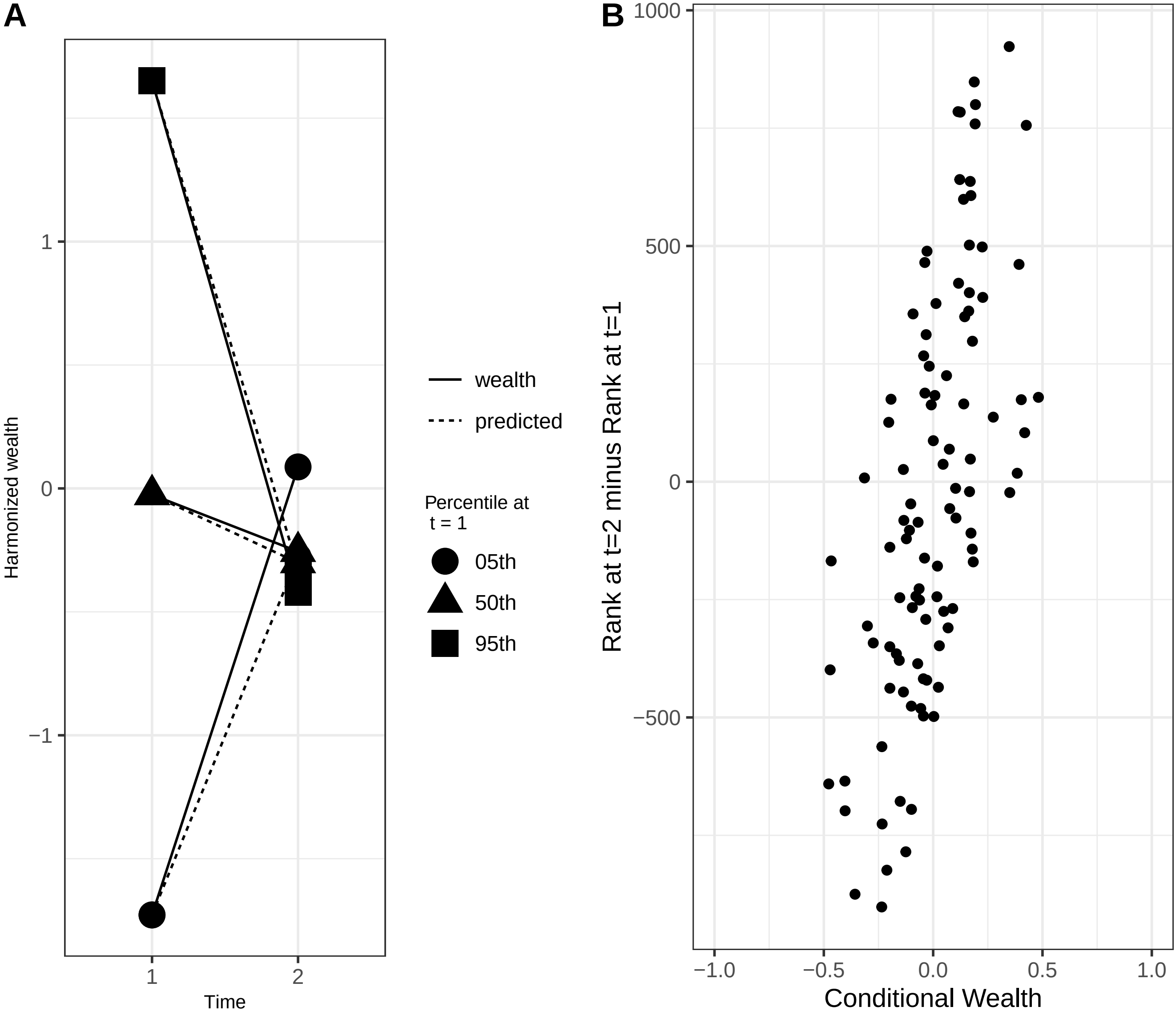

**S2Fig 18:**
**Example with w_1 = N(0,1)**
**w_2 = 0 + 0*w_1 + N(0,0.2)**
**Ratio of variance at time 2: time 1 = 0**

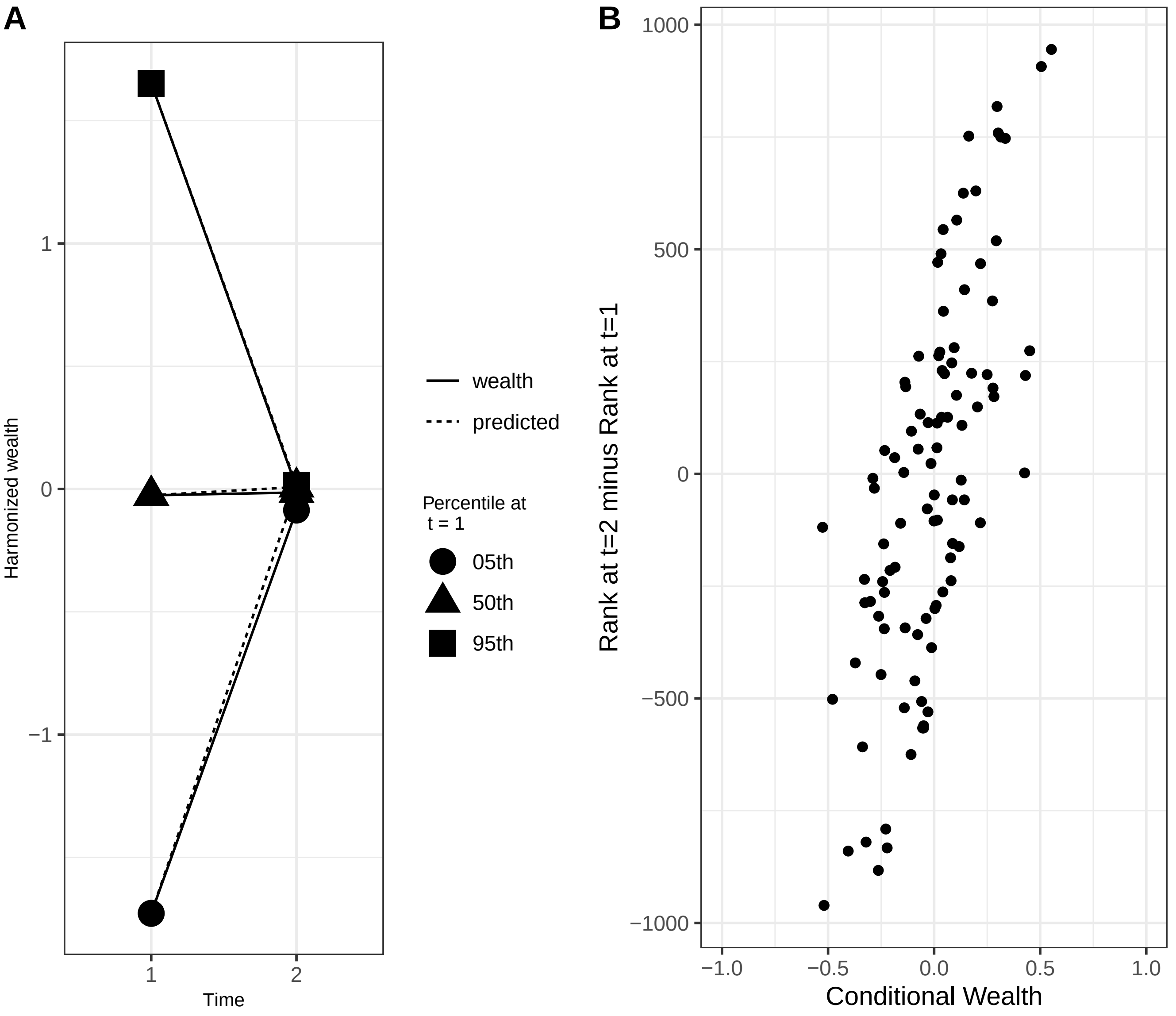

**S2Fig 19:**
**Example with w_1 = N(0,1)**
**w_2 = 0.3 + 0*w_1 + N(0,0.2)**
**Ratio of variance at time 2: time 1 = 0**

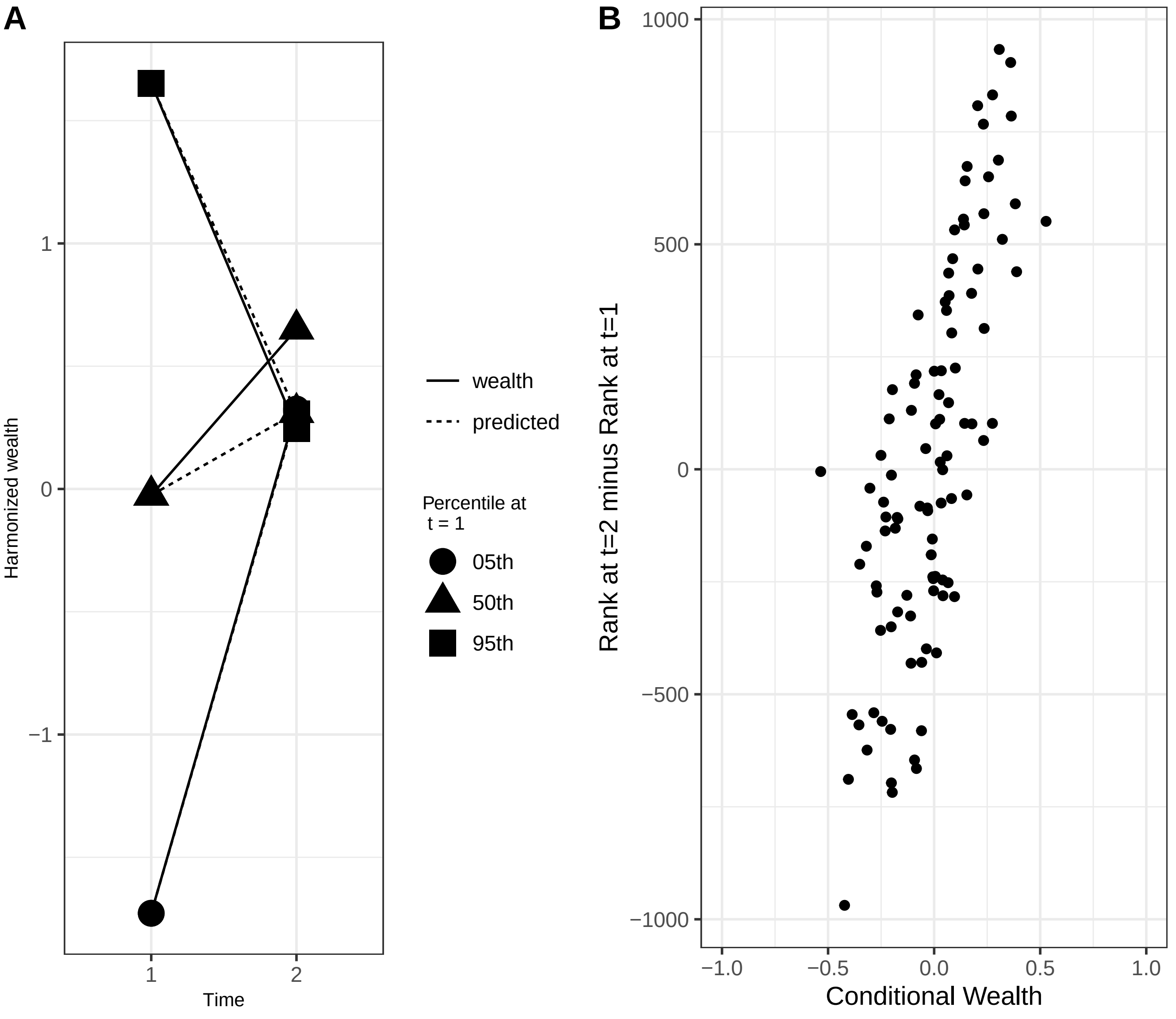

**S2Fig 20:**
**Example with w_1 = N(0,1)**
**w_2 = 0.5 + 0*w_1 + N(0,0.2)**
**Ratio of variance at time 2: time 1 = 0**

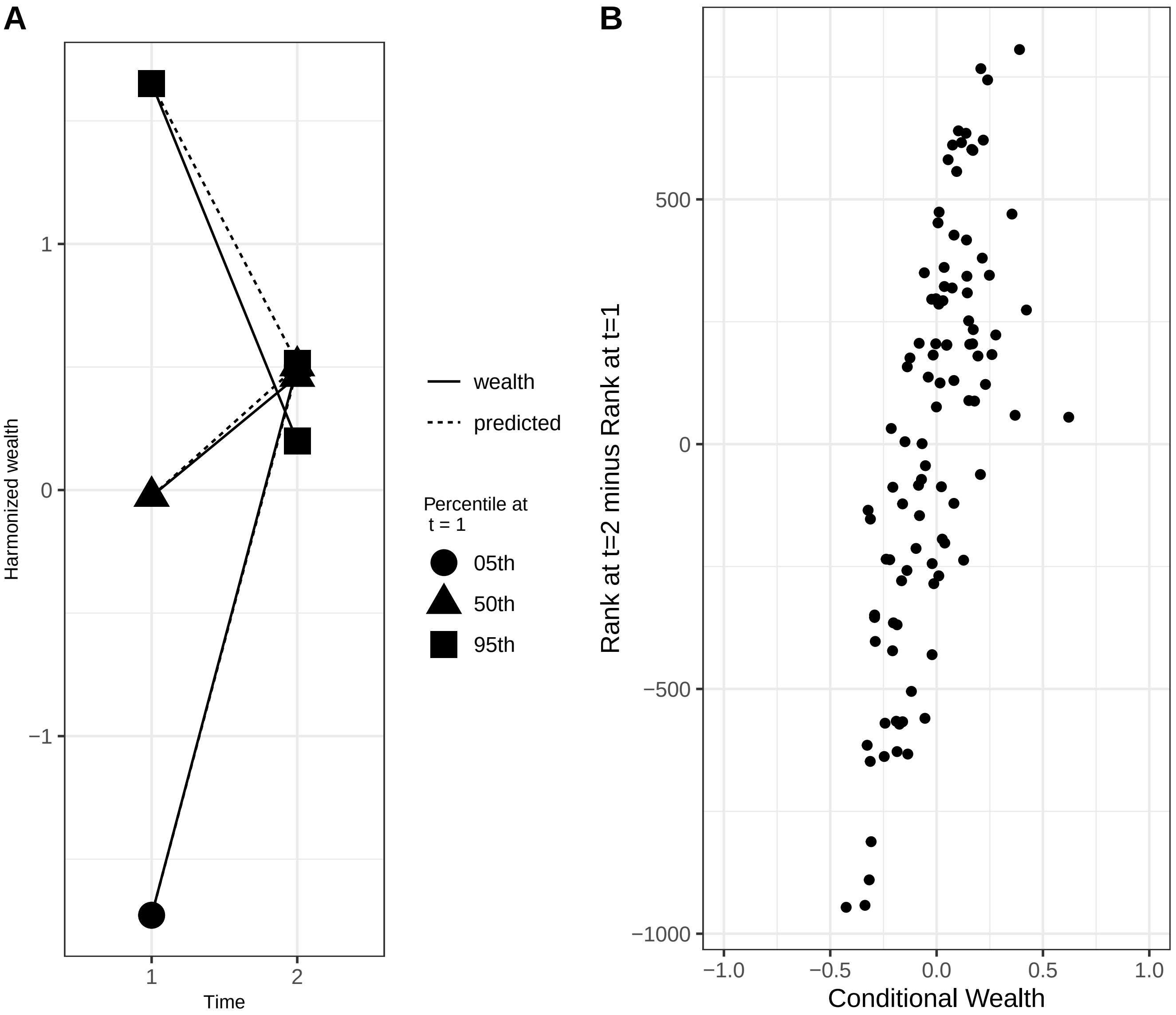

**S2Fig 21:**
**Example with w_1 = N(0,1)**
**w_2 = 1 + 0*w_1 + N(0,0.2)**
**Ratio of variance at time 2: time 1 = 0**

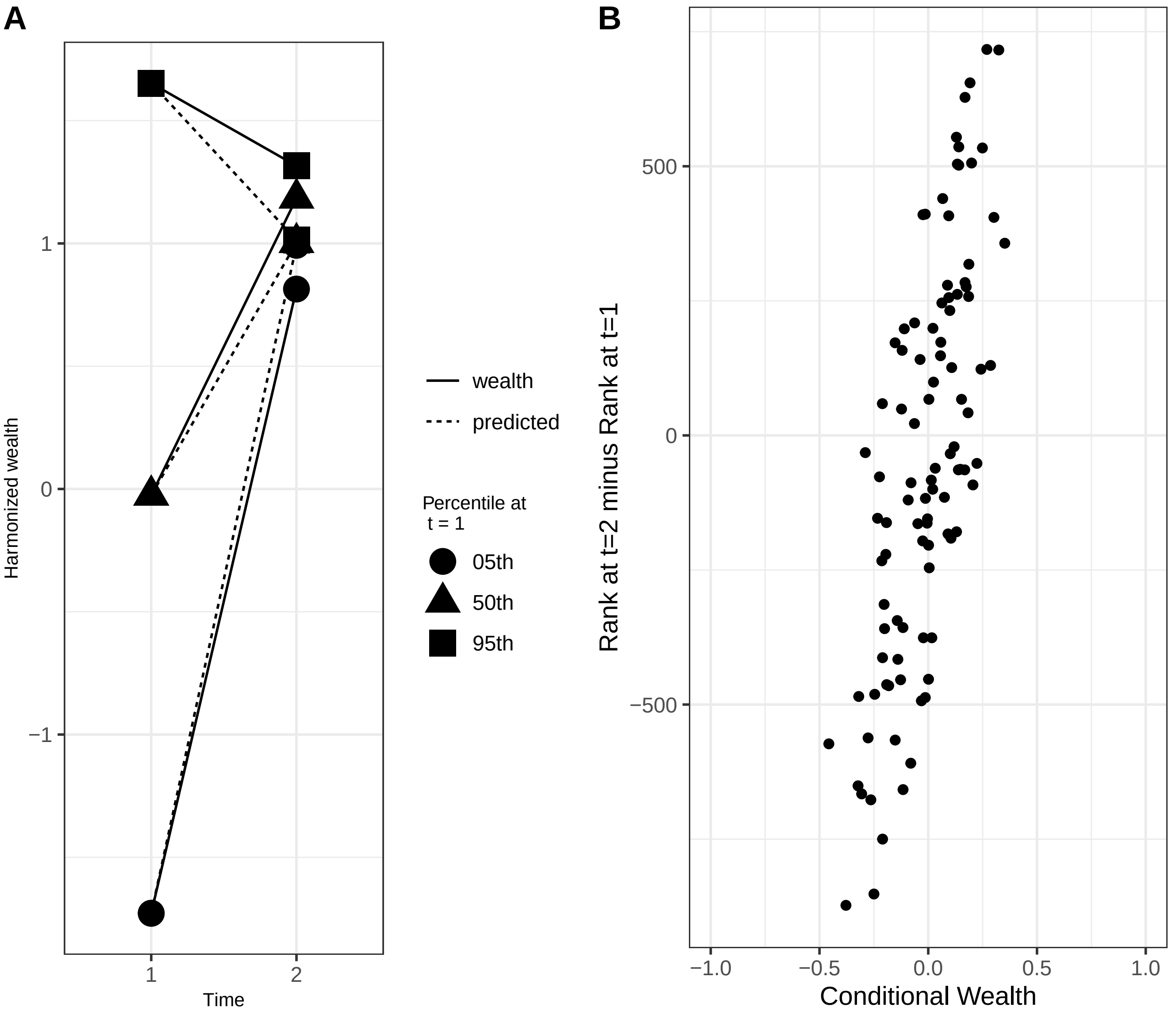

**S2Fig 22:**
**Example with w_1 = N(0,1)**
**w_2 = −1 + 0.5*w_1 + N(0,0.2)**
**Ratio of variance at time 2: time 1 = 0.3**

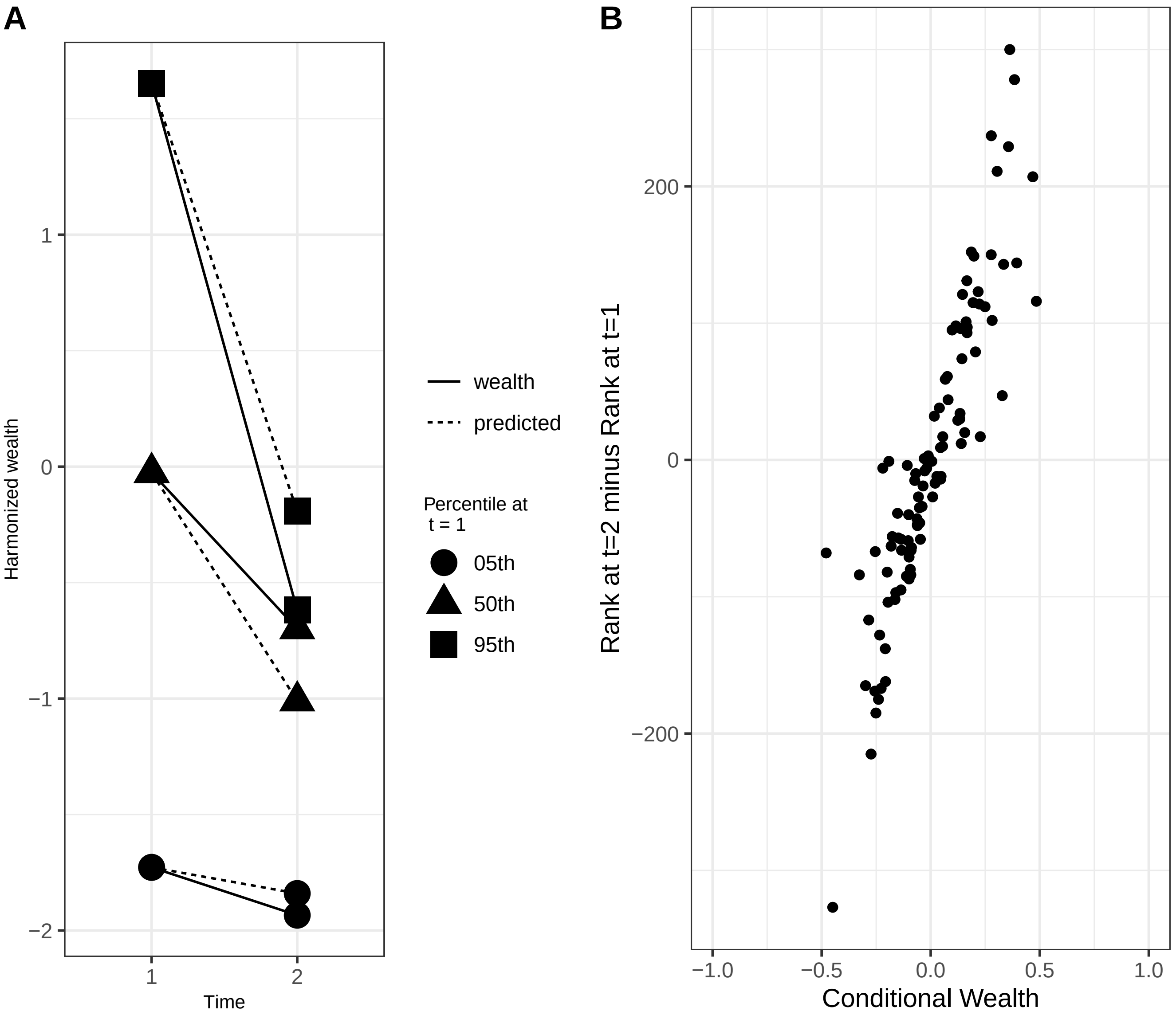

**S2Fig 23:**
**Example with w_1 = N(0,1)**
**w_2 = −0.5 + 0.5*w_1 + N(0,0.2)**
**Ratio of variance at time 2: time 1 = 0.3**

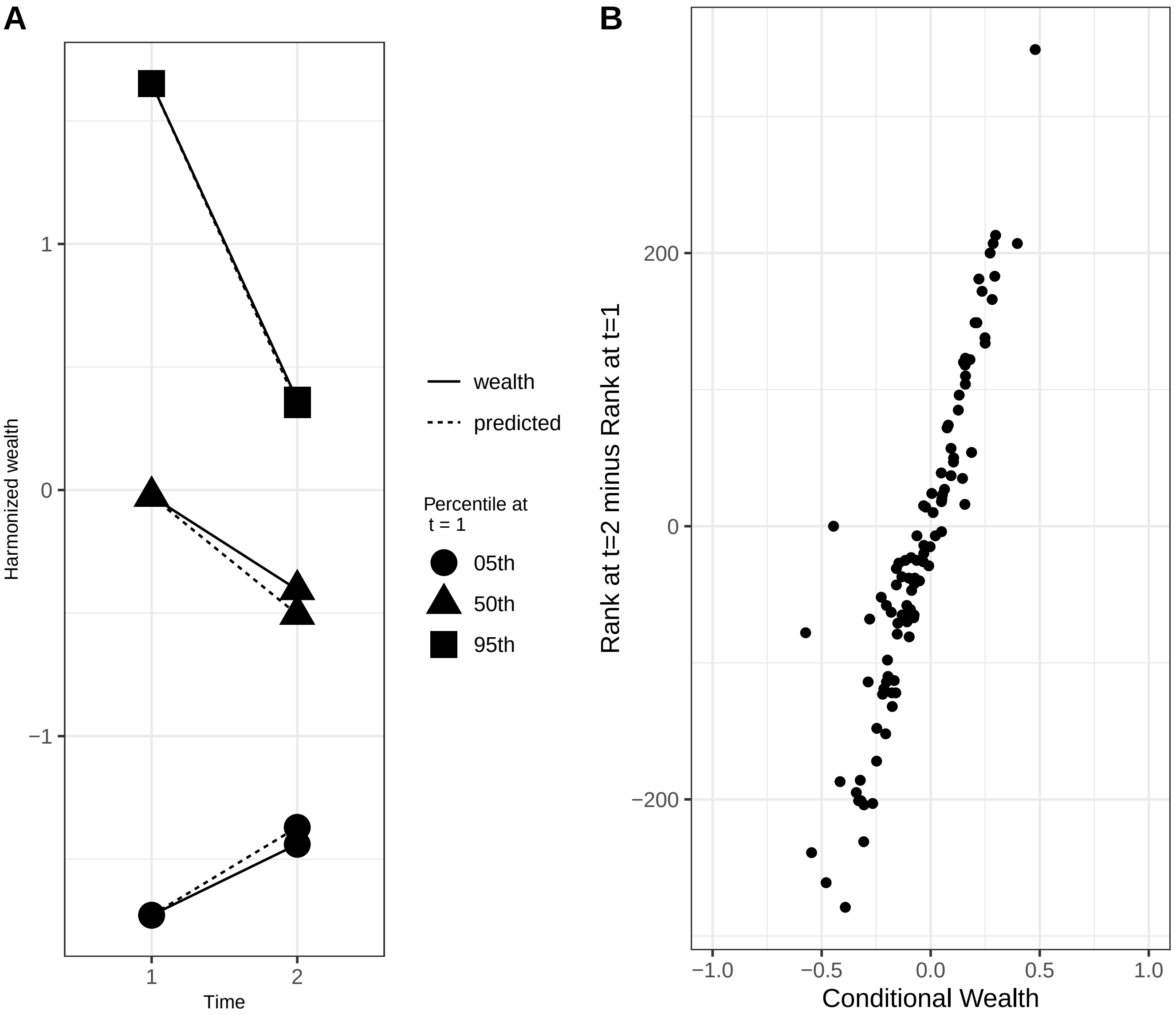

**S2Fig 24:**
**Example with w_1 = N(0,1)**
**w_2 = −0.3 + 0.5*w_1 + N(0,0.2)**
**Ratio of variance at time 2: time 1 = 0.3**

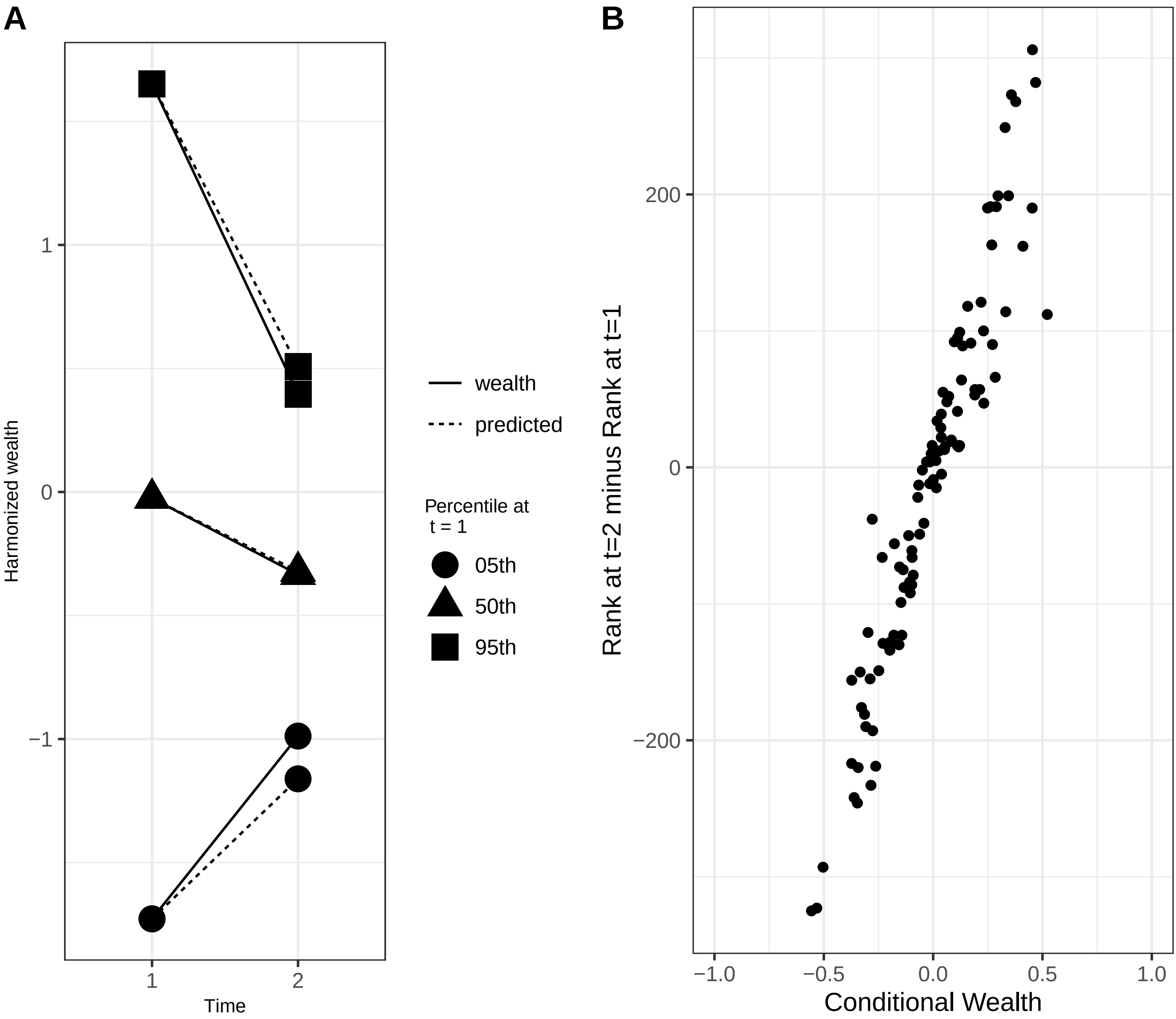

**S2Fig 25:**
**Example with w_1 = N(0,1)**
**w_2 = 0 + 0.5*w_1 + N(0,0.2)**
**Ratio of variance at time 2: time 1 = 0.3**

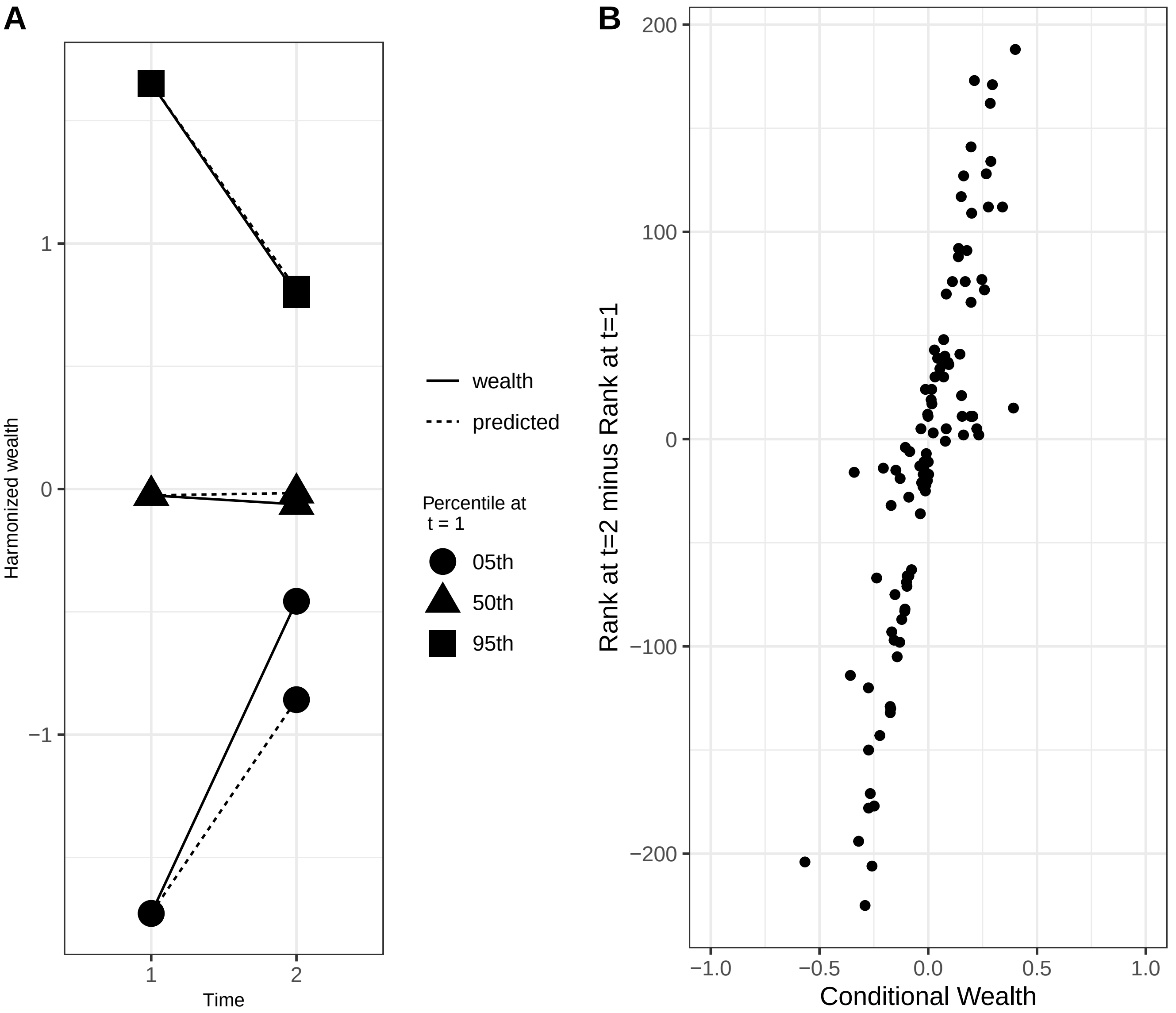

**S2Fig 26:**
**Example with w_1 = N(0,1)**
**w_2 = 0.3 + 0.5*w_1 + N(0,0.2)**
**Ratio of variance at time 2: time 1 = 0.3**

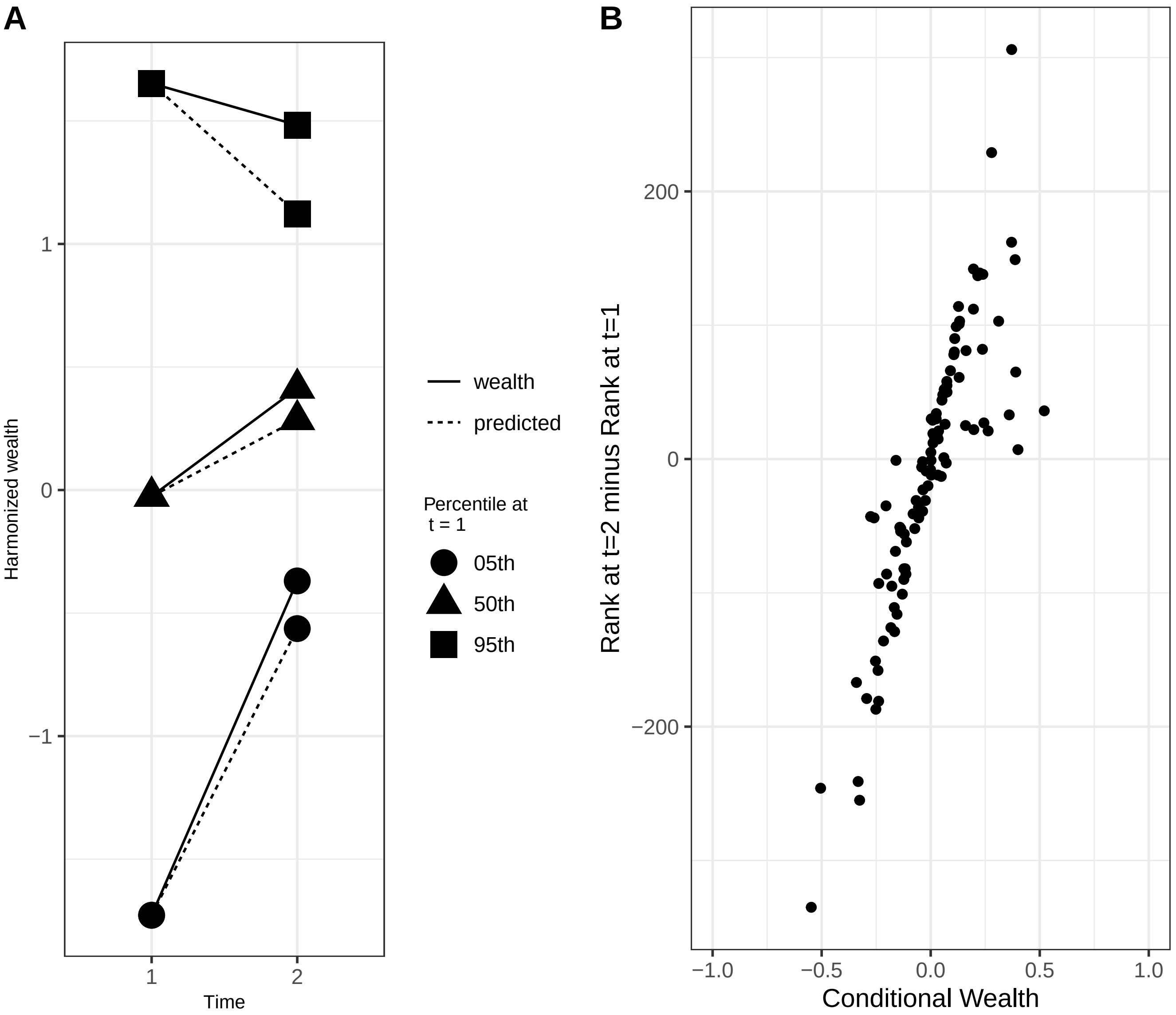

**S2Fig 27:**
**Example with w_1 = N(0,1)**
**w_2 = 0.5 + 0.5*w_1 + N(0,0.2)**
**Ratio of variance at time 2: time 1 = 0.3**

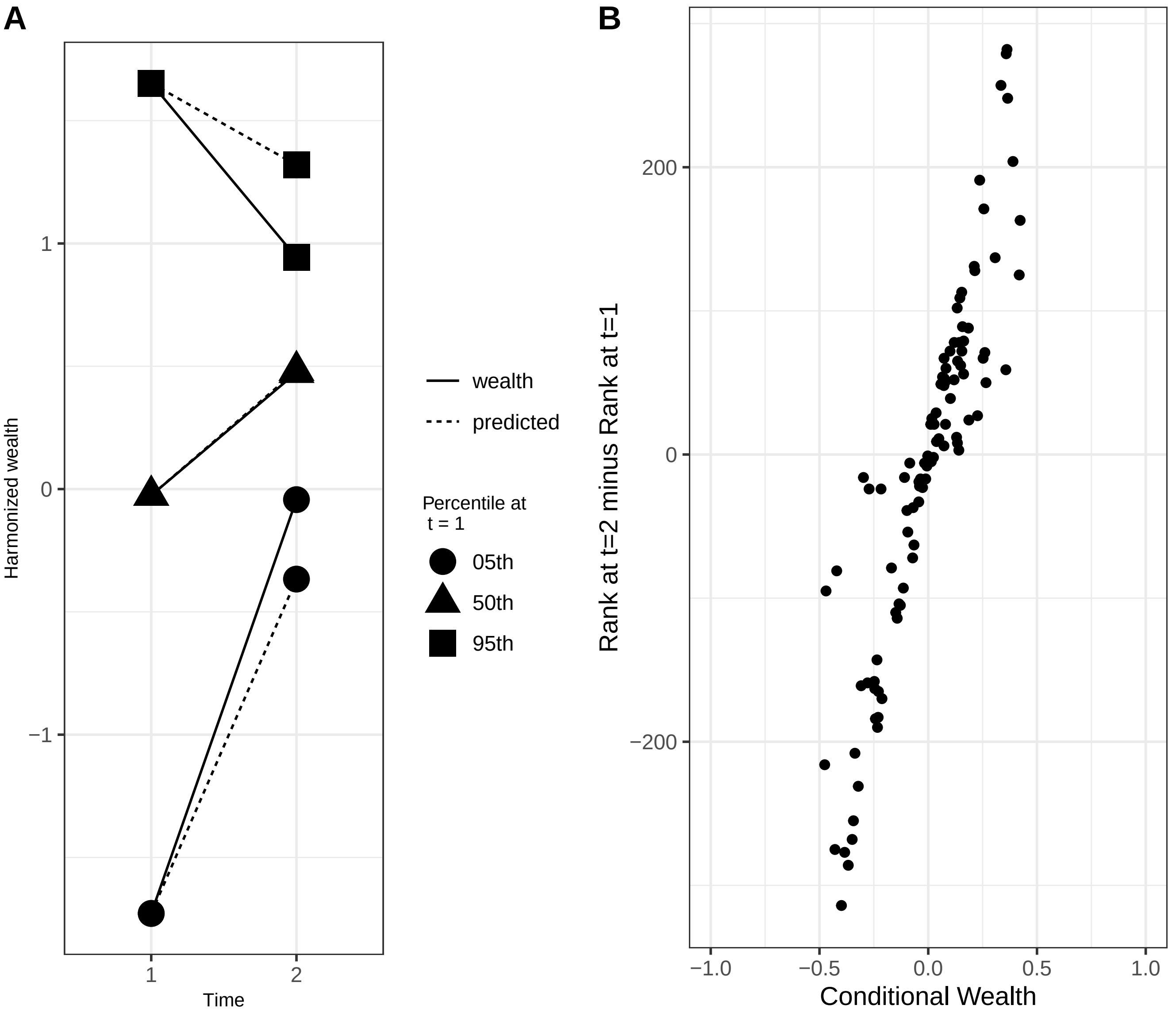

**S2Fig 28:**
**Example with w_1 = N(0,1)**
**w_2 = 1 + 0.5*w_1 + N(0,0.2)**
**Ratio of variance at time 2: time 1 = 0.3**

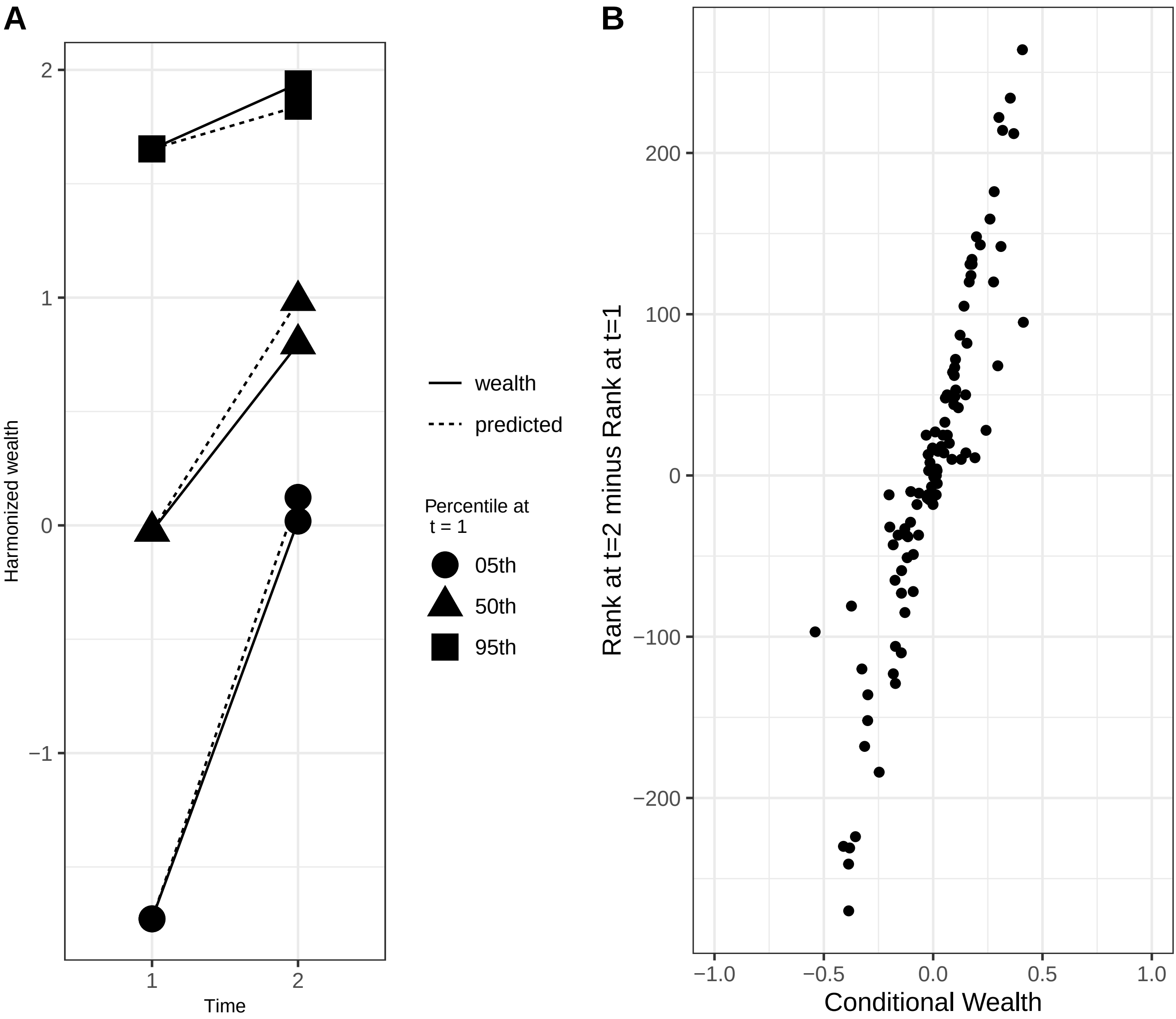

**S2Fig 29:**
**Example with w_1 = N(0,1)**
**w_2 = −1 + 1*w_1 + N(0,0.2)**
**Ratio of variance at time 2: time 1 = 1.1**

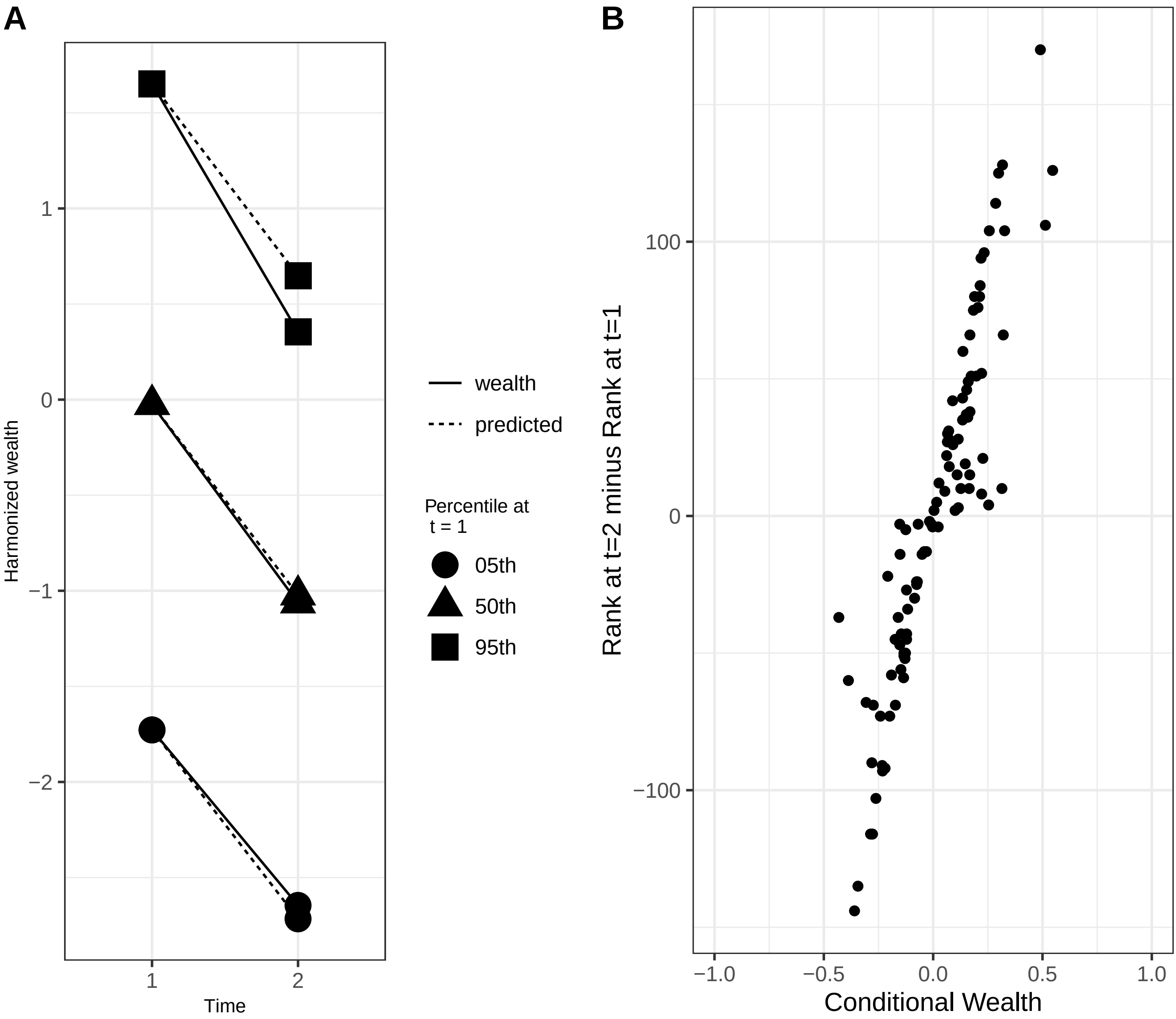

**S2Fig 30:**
**Example with w_1 = N(0,1)**
**w_2 = −0.5 + 1*w_1 + N(0,0.2)**
**Ratio of variance at time 2: time 1 = 1.1**

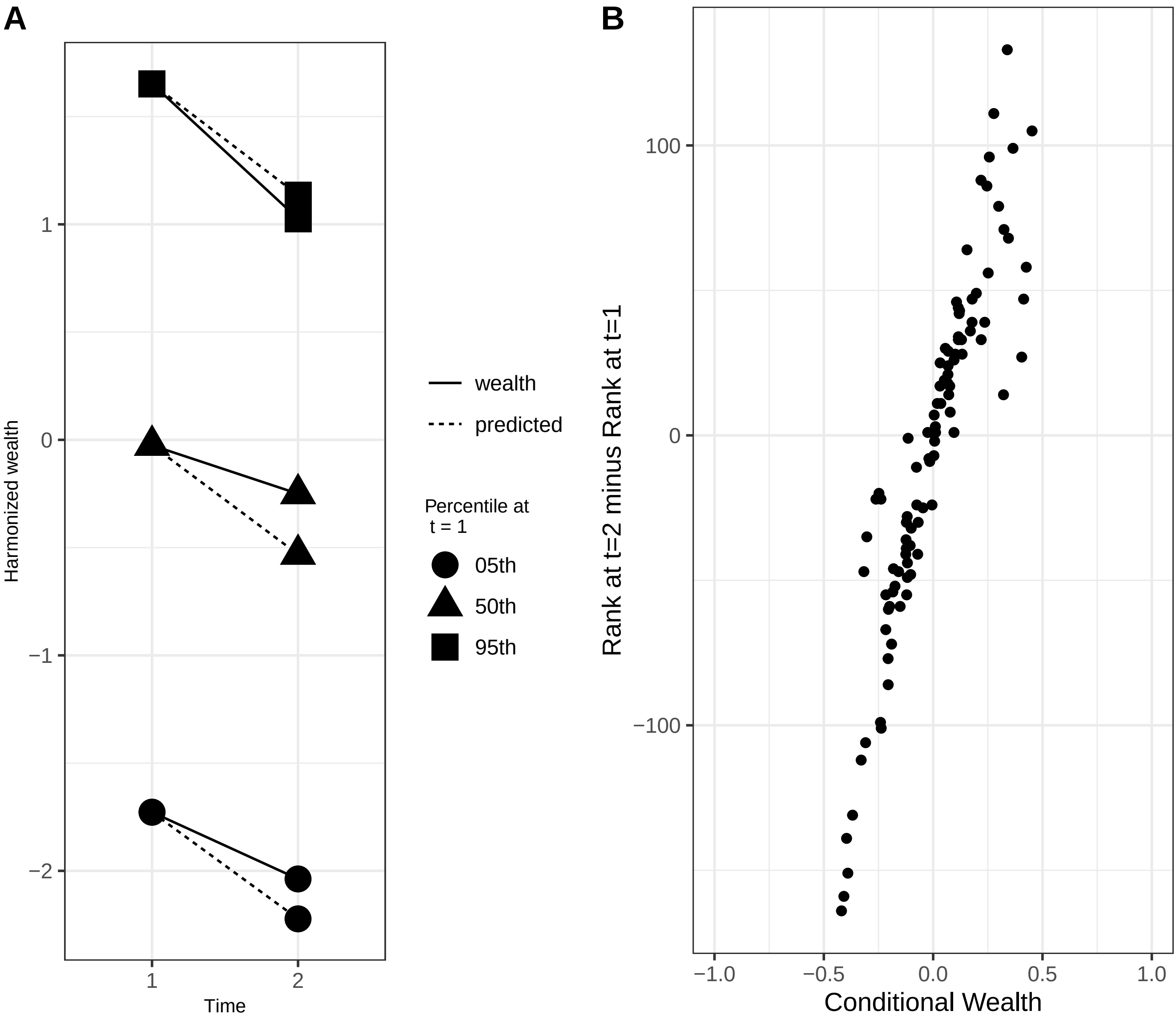

**S2Fig 31:**
**Example with w_1 = N(0,1)**
**w_2 = −0.3 + 1*w_1 + N(0,0.2)**
**Ratio of variance at time 2: time 1 = 1.1**

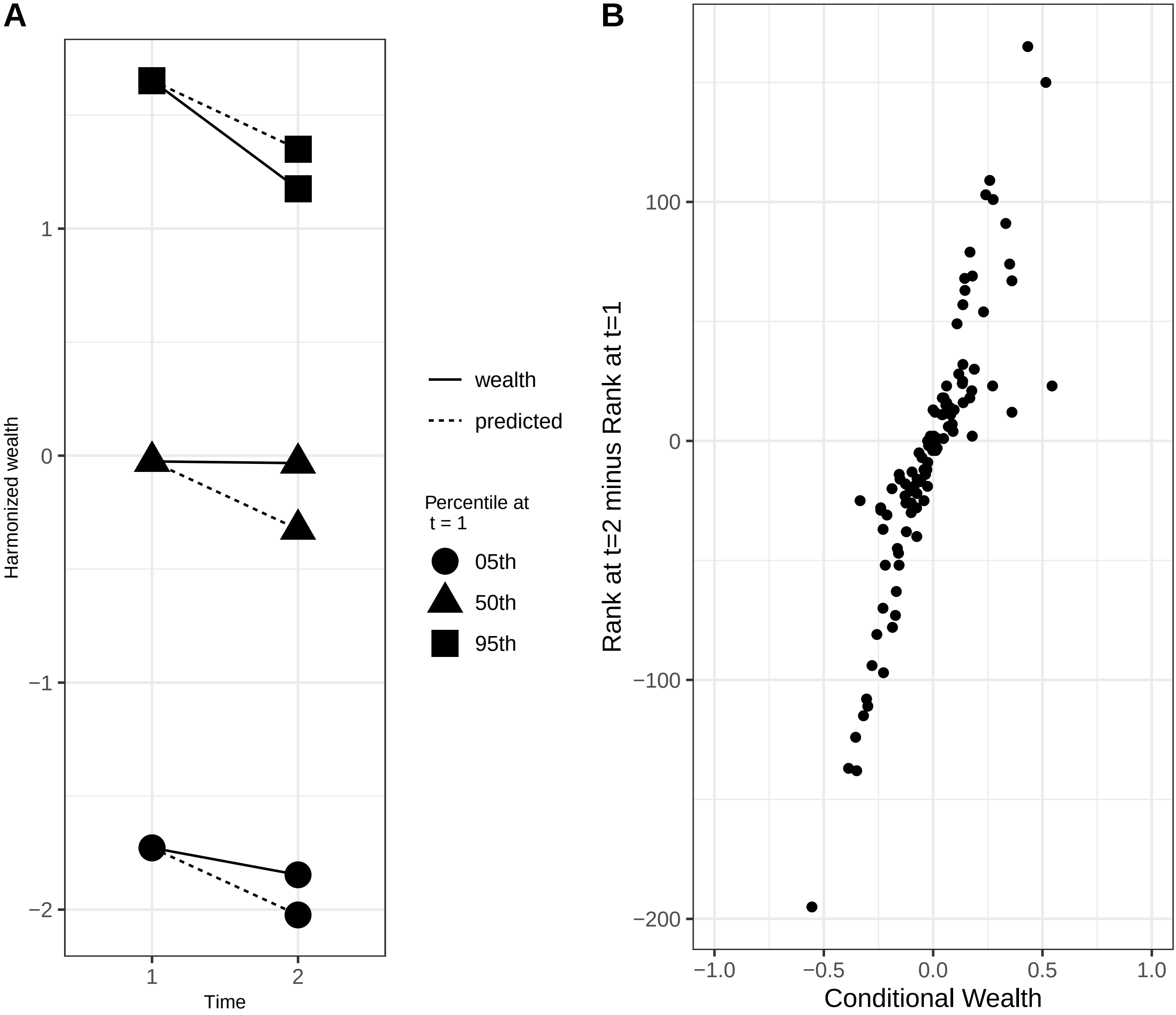

**S2Fig 32:**
**Example with w_1 = N(0,1)**
**w_2 = 0 + 1*w_1 + N(0,0.2)**
**Ratio of variance at time 2: time 1 = 1.1**

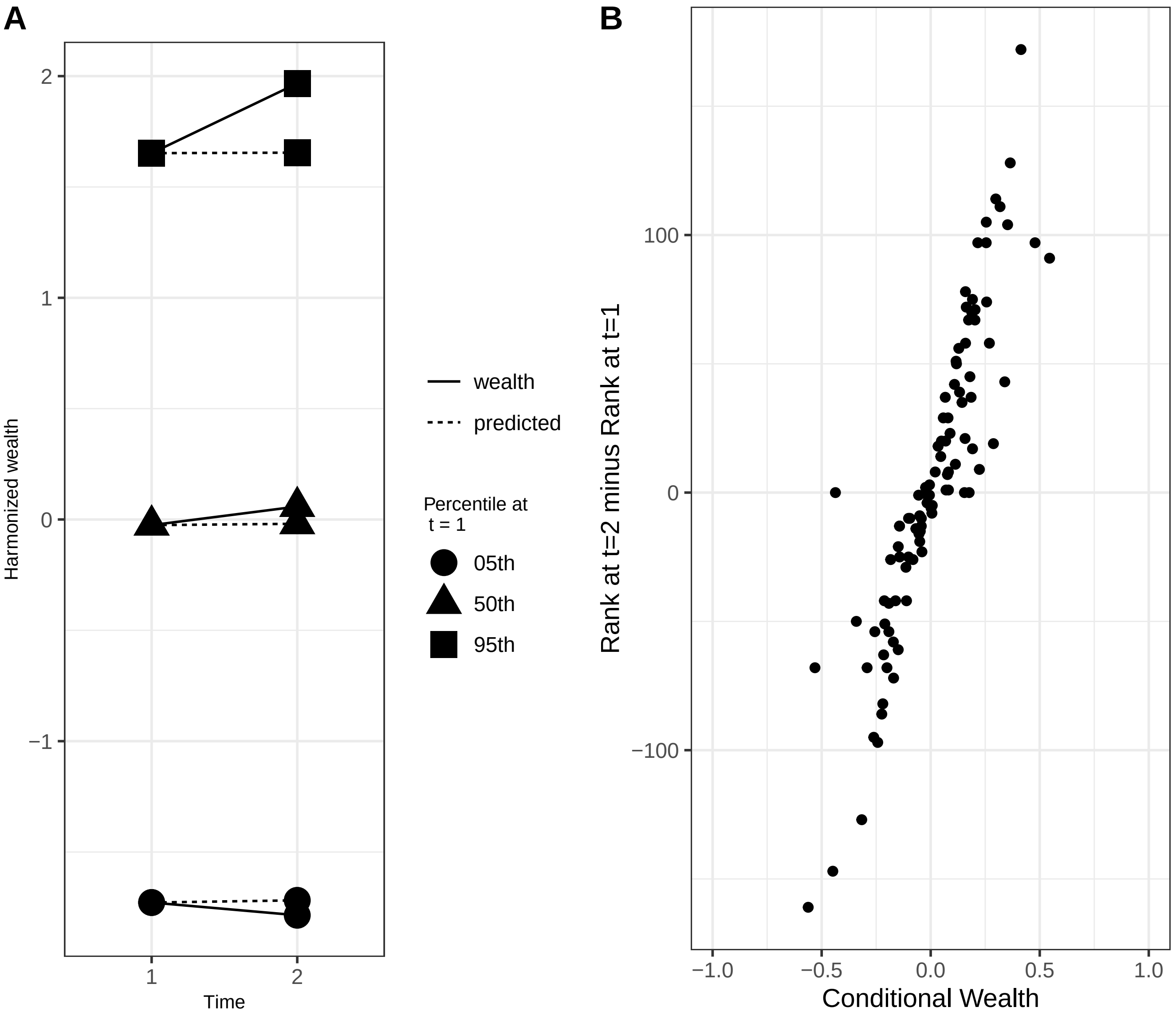

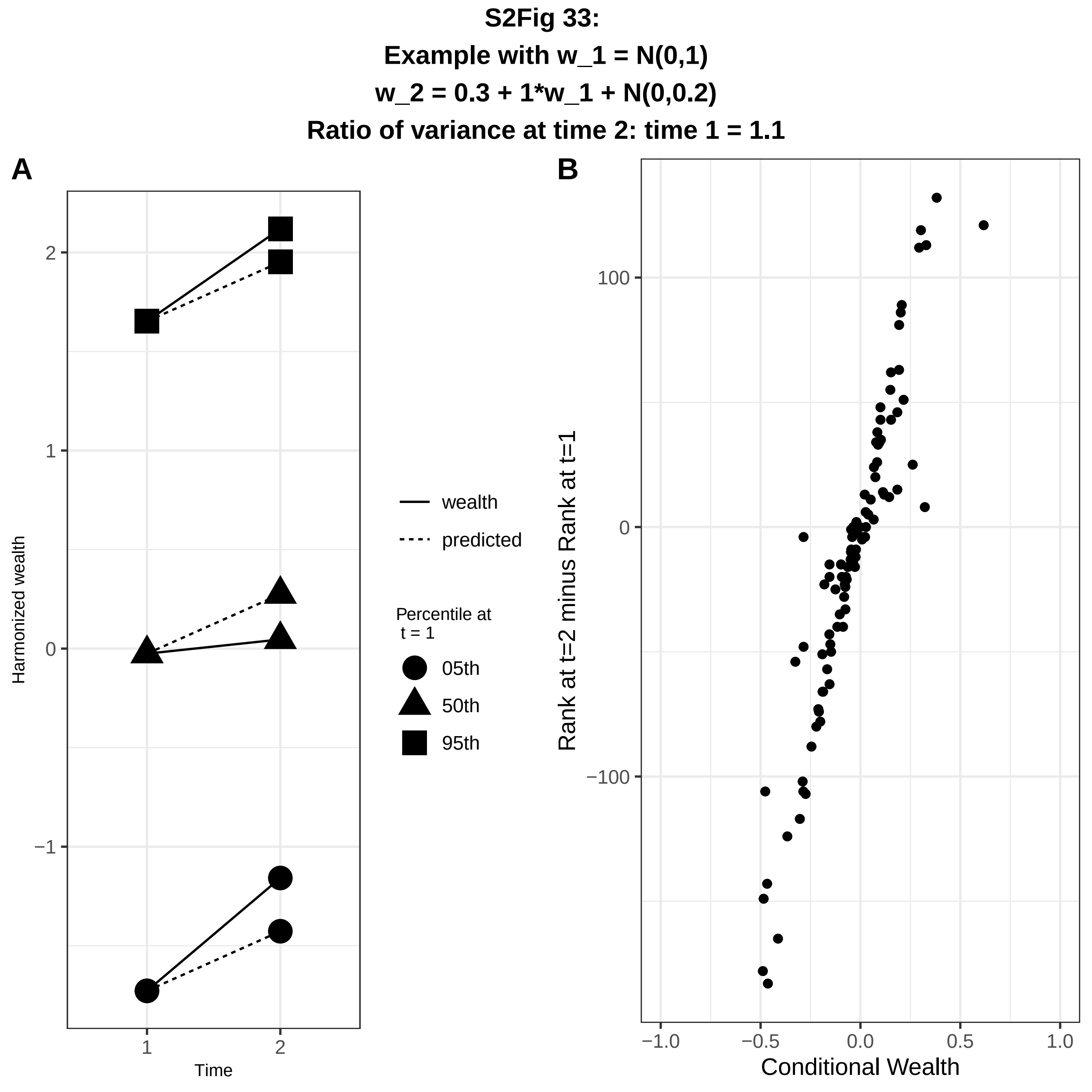
S2Fig 33:
Example with w_1 = N(0,1)
w_2 = 0.3 + 1*w_1 + N(0,0.2)
Ratio of variance at time 2: time 1 = 1.1
A
Harmonized wealth
2
1
0
−1
Time
1
2
wealth
predicted
Percentile at t = 1
05th
50th
95th
B
Rank at t=2 minus Rank at t=1
100
0
−100
Conditional Wealth
−1.0
−0.5
0.0
0.5
1.0

**S2Fig 34:**
**Example with w_1 = N(0,1)**
**w_2 = 0.5 + 1*w_1 + N(0,0.2)**
**Ratio of variance at time 2: time 1 = 1.1**

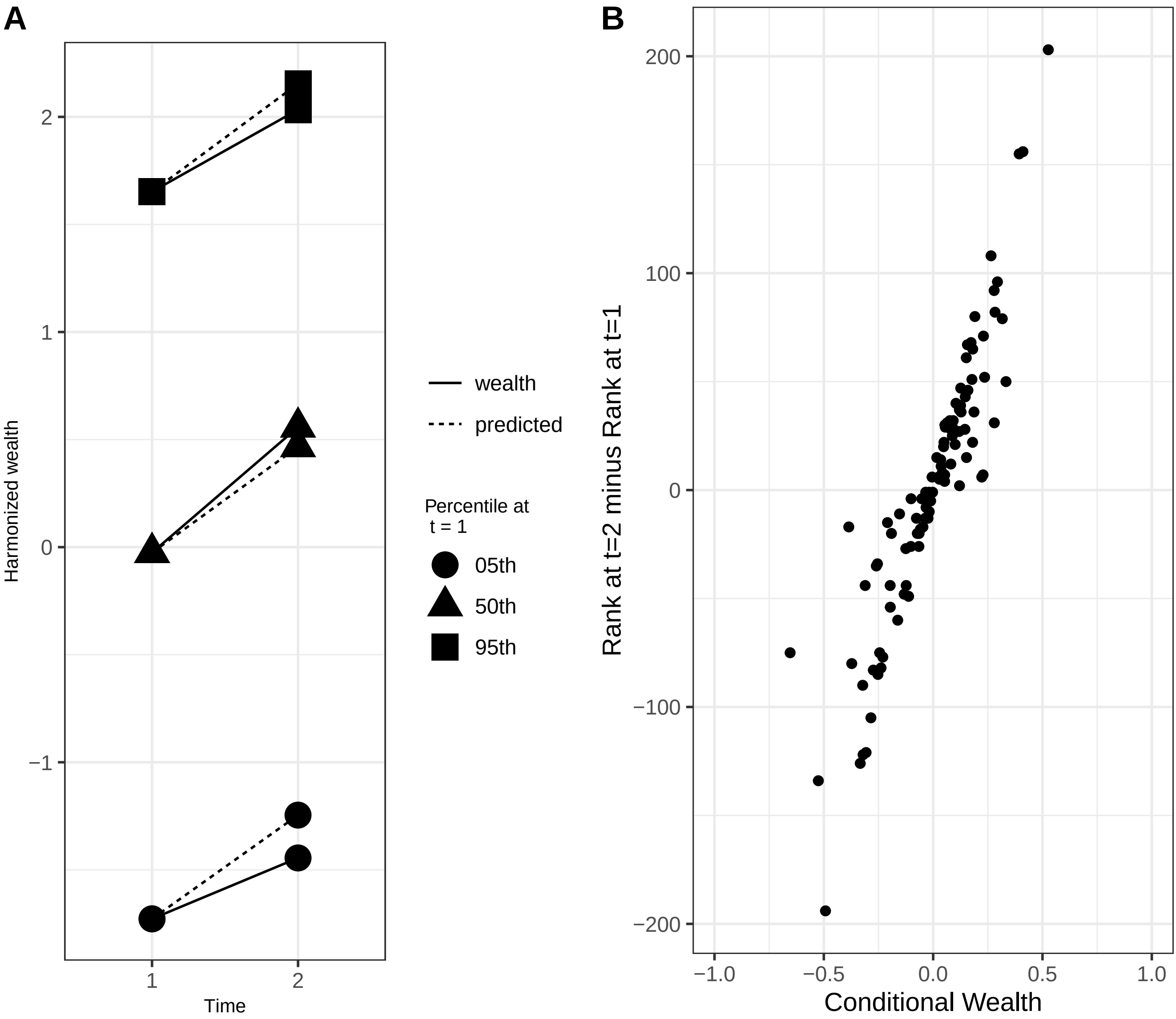

**S2Fig 35:**
**Example with w_1 = N(0,1)**
**w_2 = 1 + 1*w_1 + N(0,0.2)**
**Ratio of variance at time 2: time 1 = 1.1**

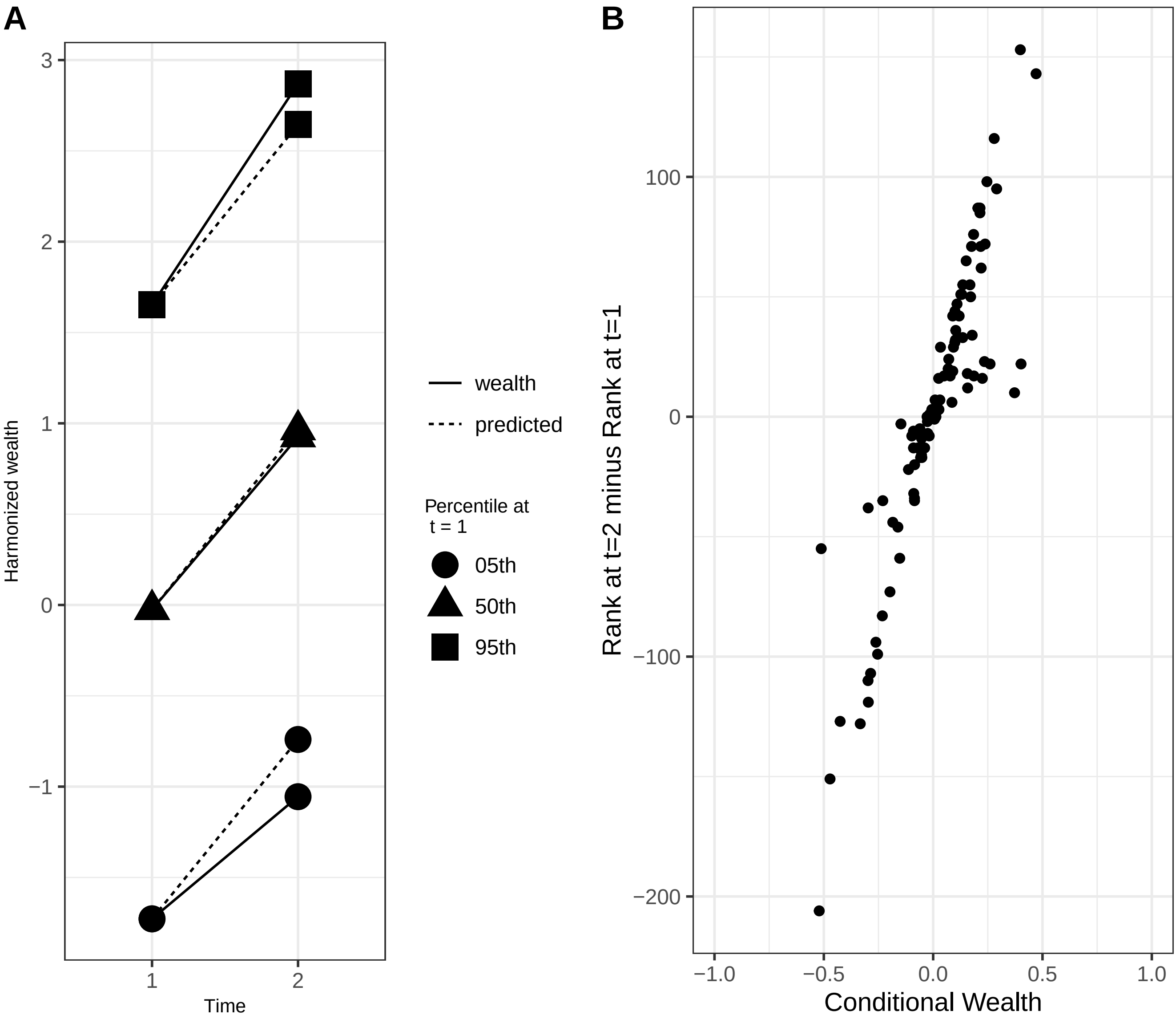

**S2Fig 36:**
**Example with w_1 = N(0,1)**
**w_2 = −1 + −1*w_1 + N(0,0.5)**
**Ratio of variance at time 2: time 1 = 1.3**

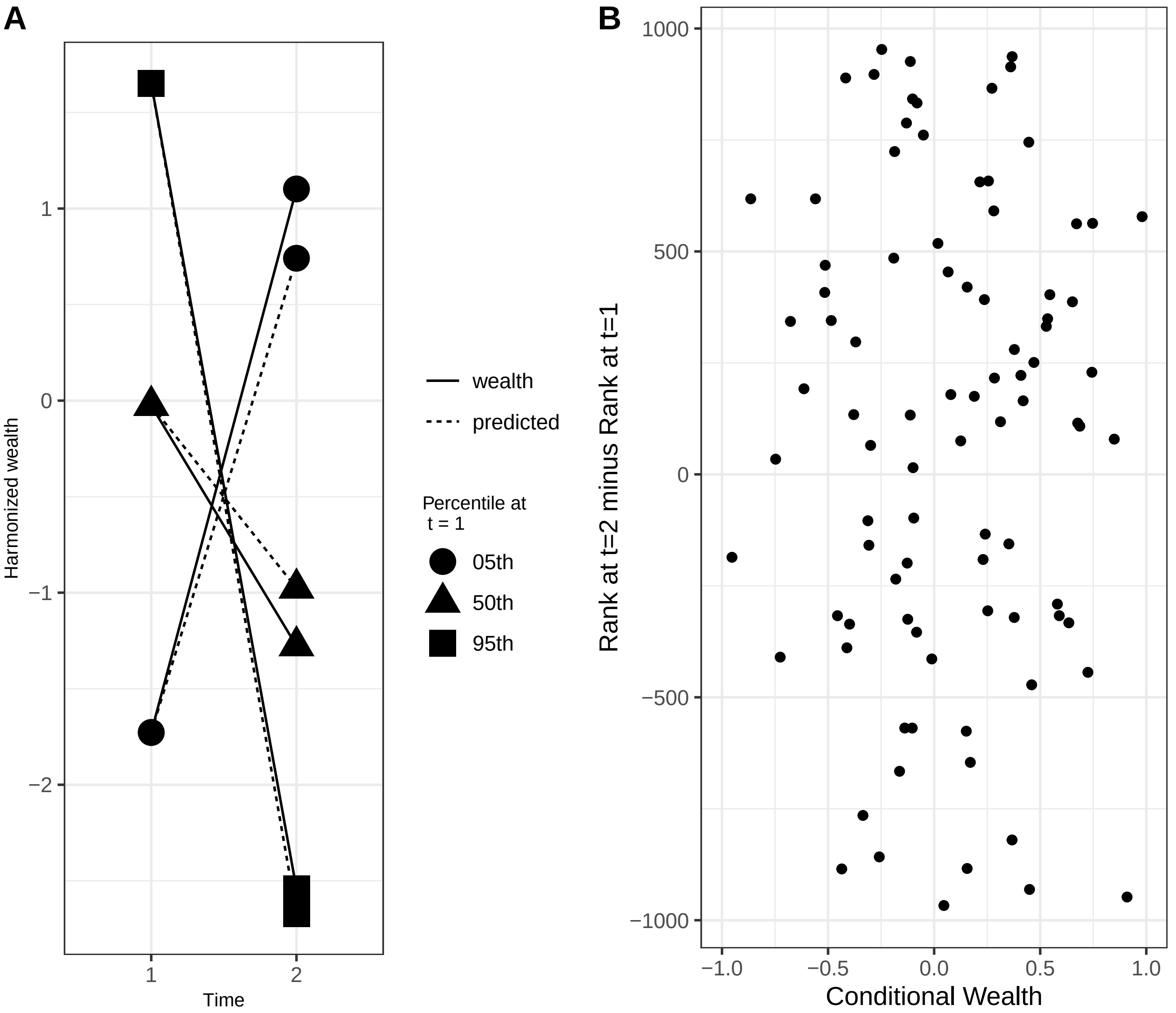

**S2Fig 37:**
**Example with w_1 = N(0,1)**
**w_2 = −0.5 + −1*w_1 + N(0,0.5)**
**Ratio of variance at time 2: time 1 = 1.3**

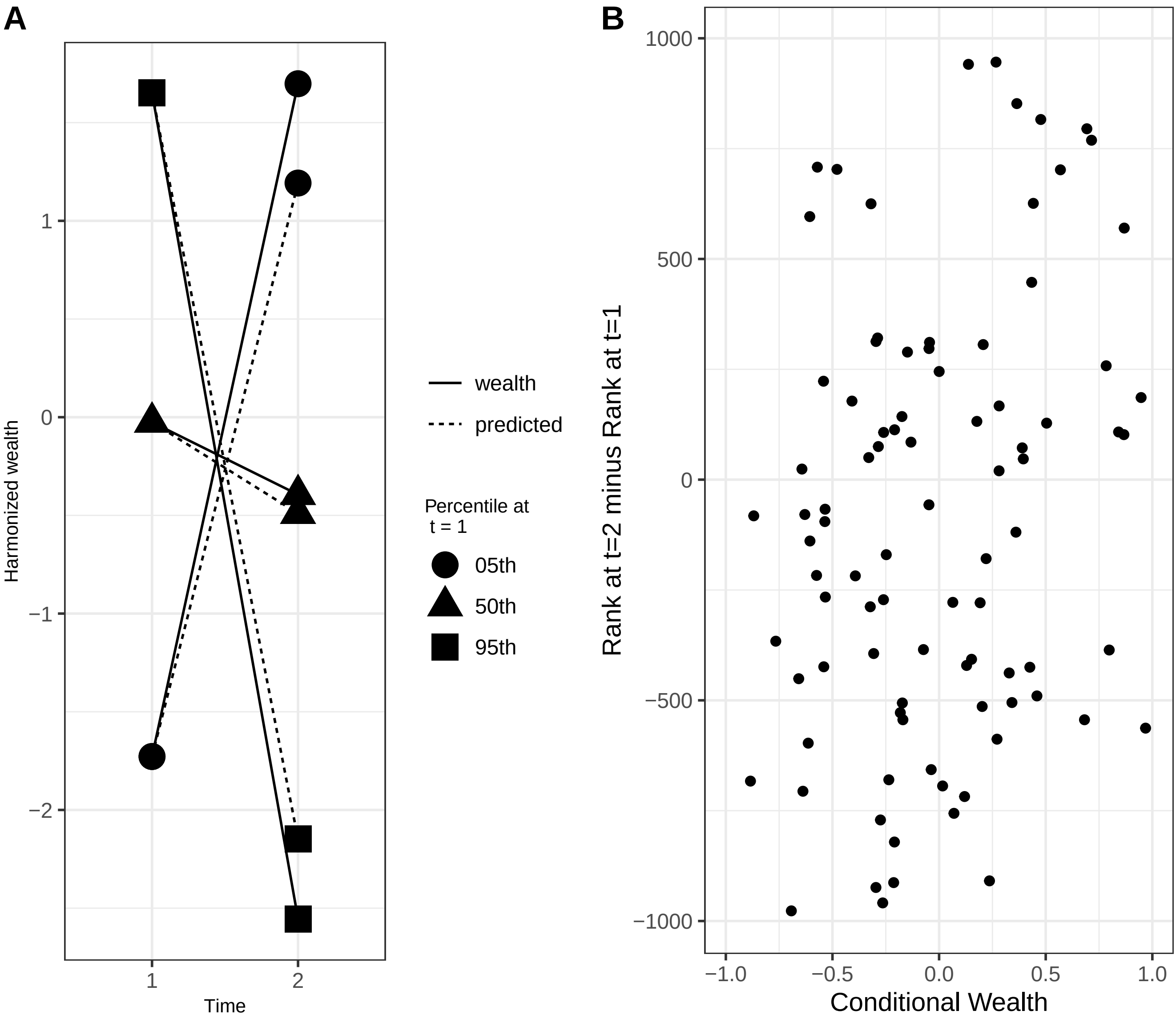

## S2Fig 38:
## Example with w_1 = N(0,1)
## w_2 = −0.3 + −1*w_1 + N(0,0.5)
## Ratio of variance at time 2: time 1 = 1.3

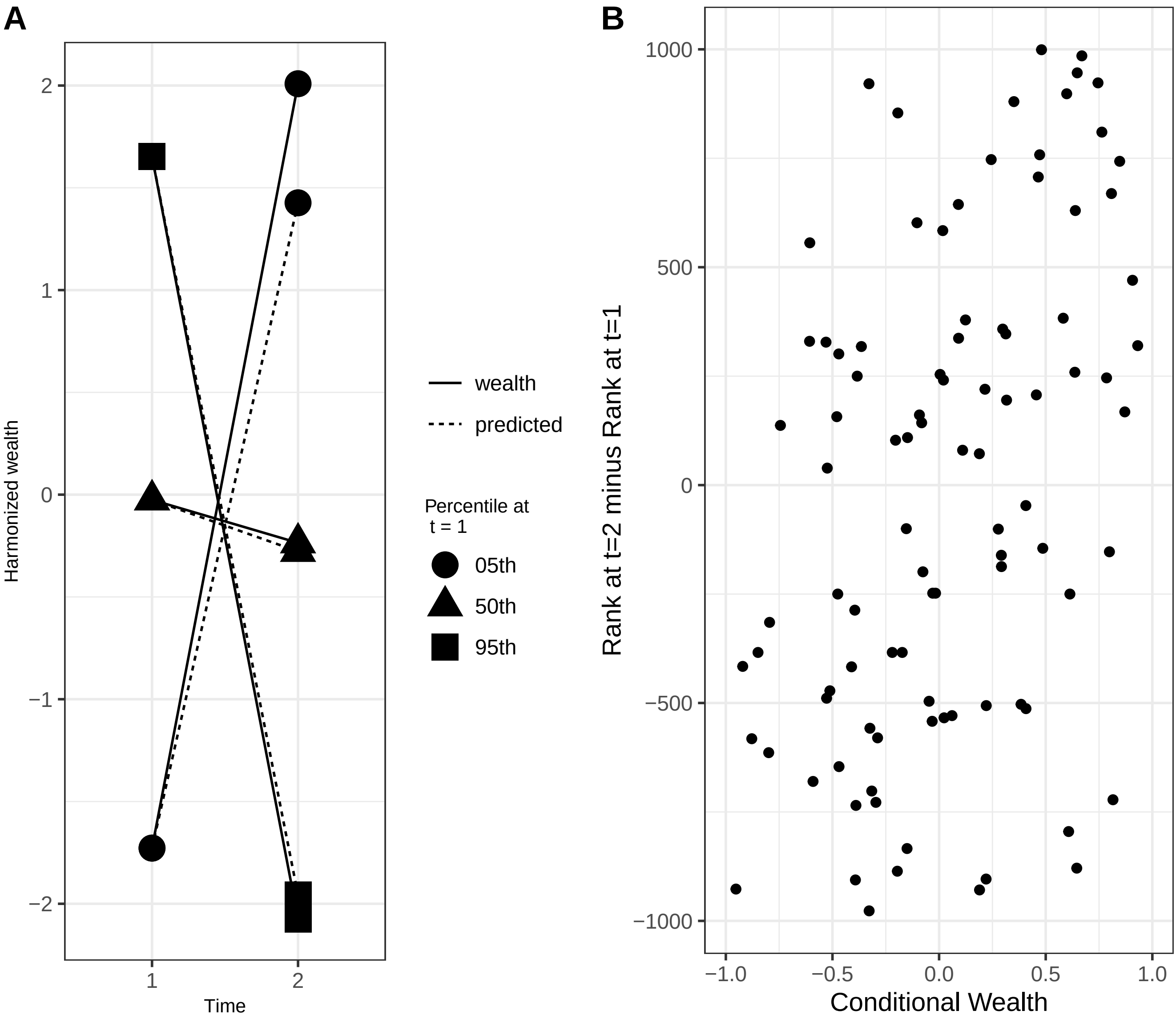

**S2Fig 39:**
**Example with w_1 = N(0,1)**
**w_2 = 0 + −1*w_1 + N(0,0.5)**
**Ratio of variance at time 2: time 1 = 1.3**

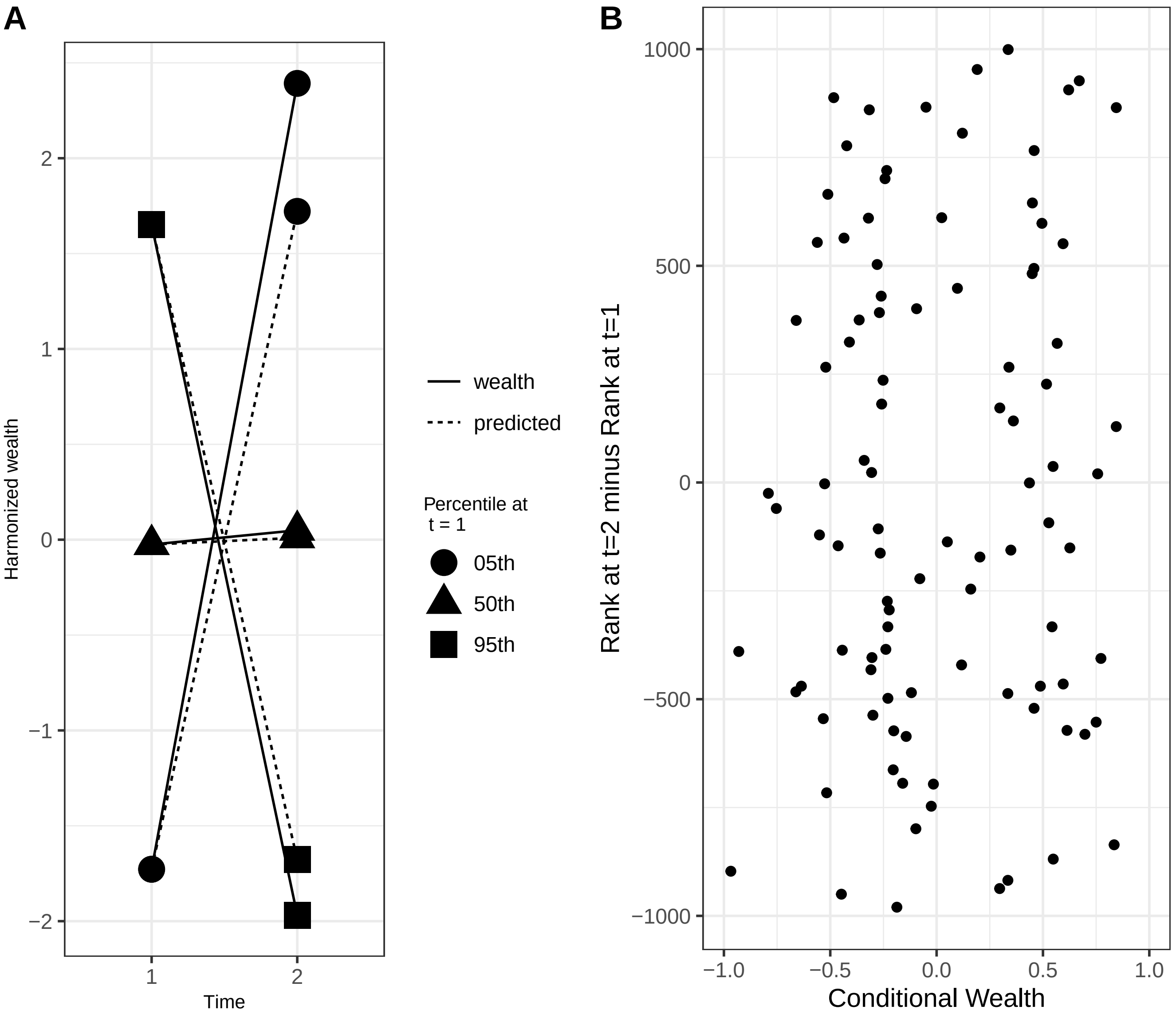

**S2Fig 40:**
**Example with w_1 = N(0,1)**
**w_2 = 0.3 + −1*w_1 + N(0,0.5)**
**Ratio of variance at time 2: time 1 = 1.3**

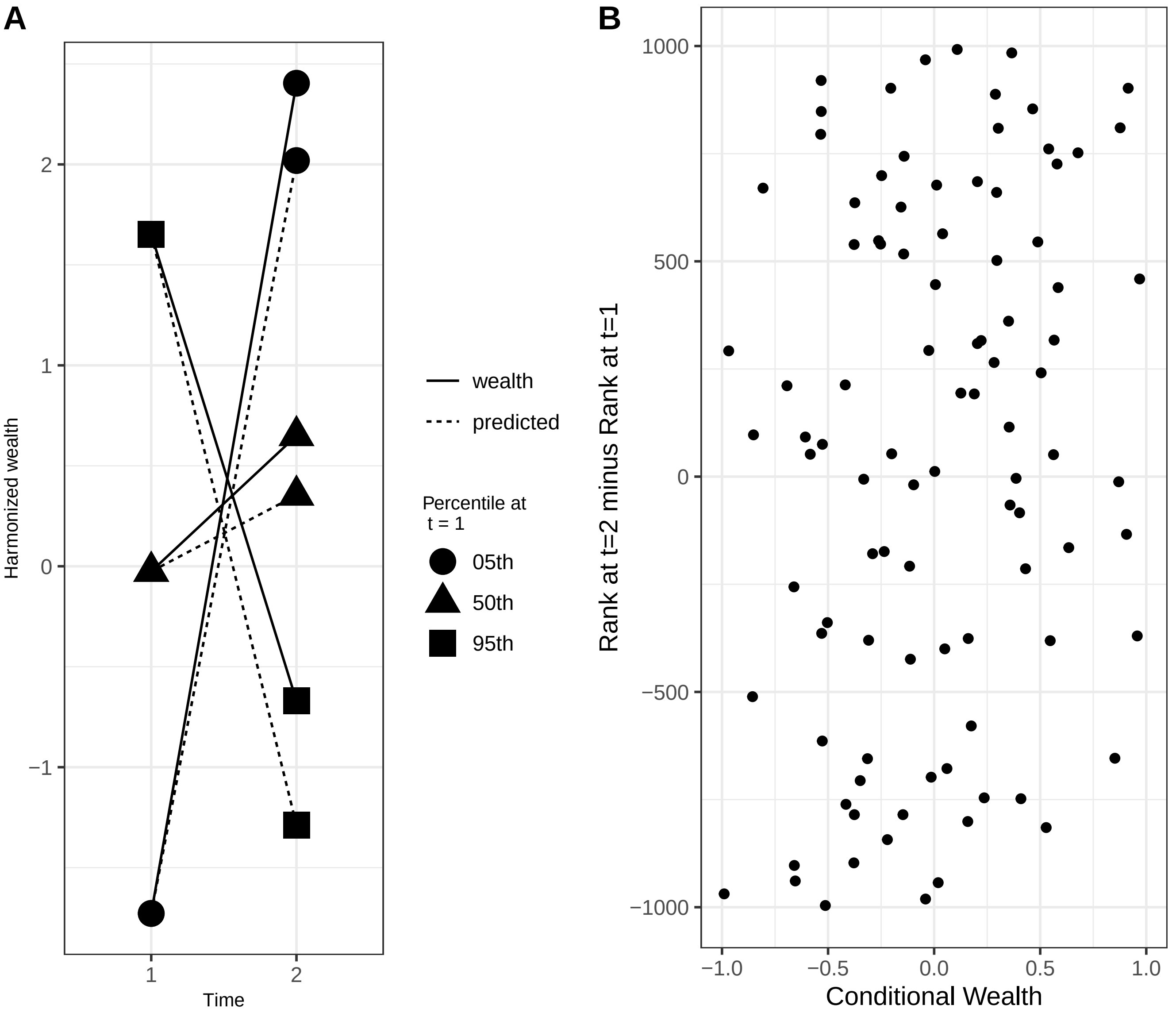

**S2Fig 41:**
**Example with w_1 = N(0,1)**
**w_2 = 0.5 + −1*w_1 + N(0,0.5)**
**Ratio of variance at time 2: time 1 = 1.2**

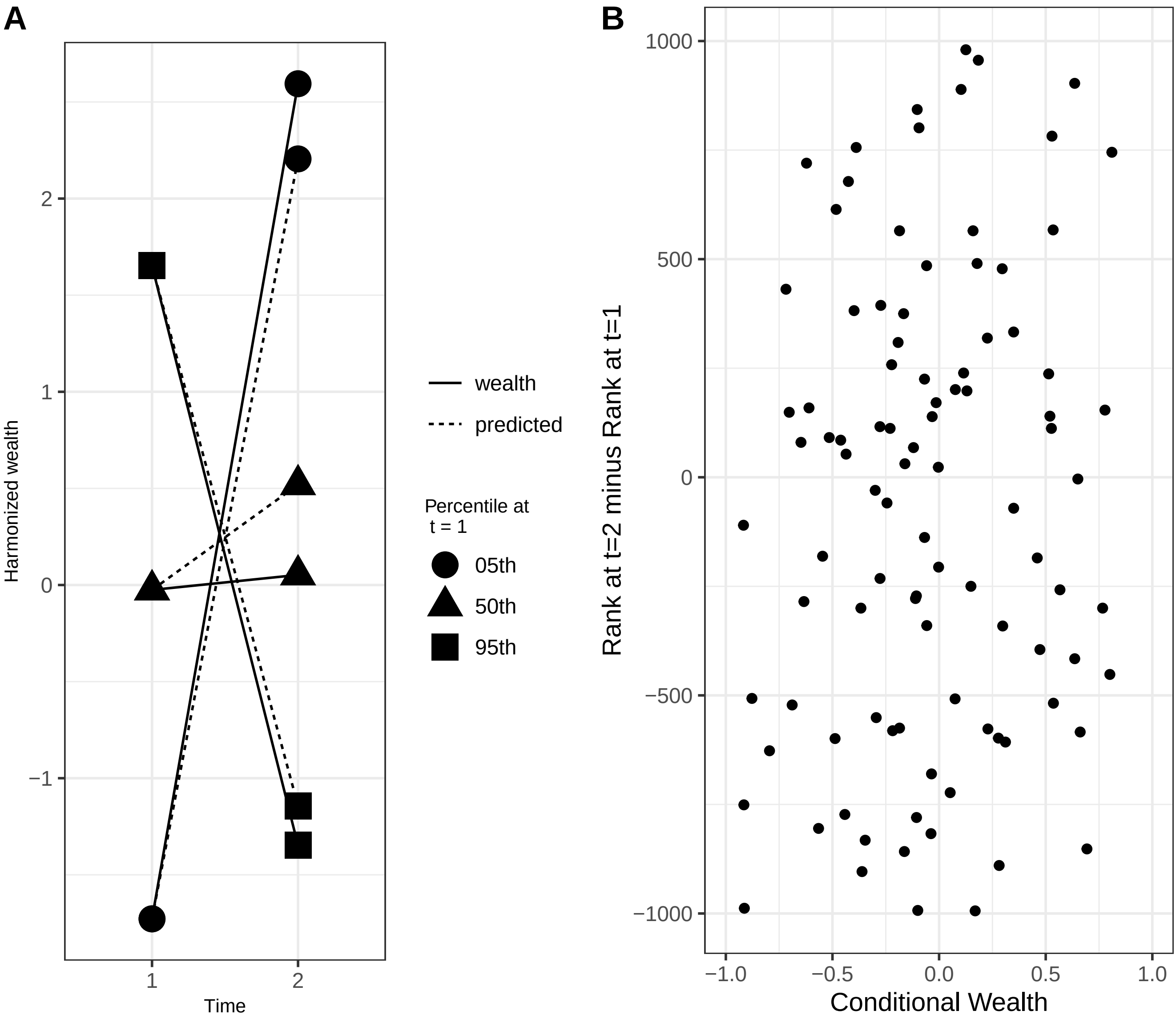

**S2Fig 42:**
**Example with w_1 = N(0,1)**
**w_2 = 1 + −1*w_1 + N(0,0.5)**
**Ratio of variance at time 2: time 1 = 1.3**

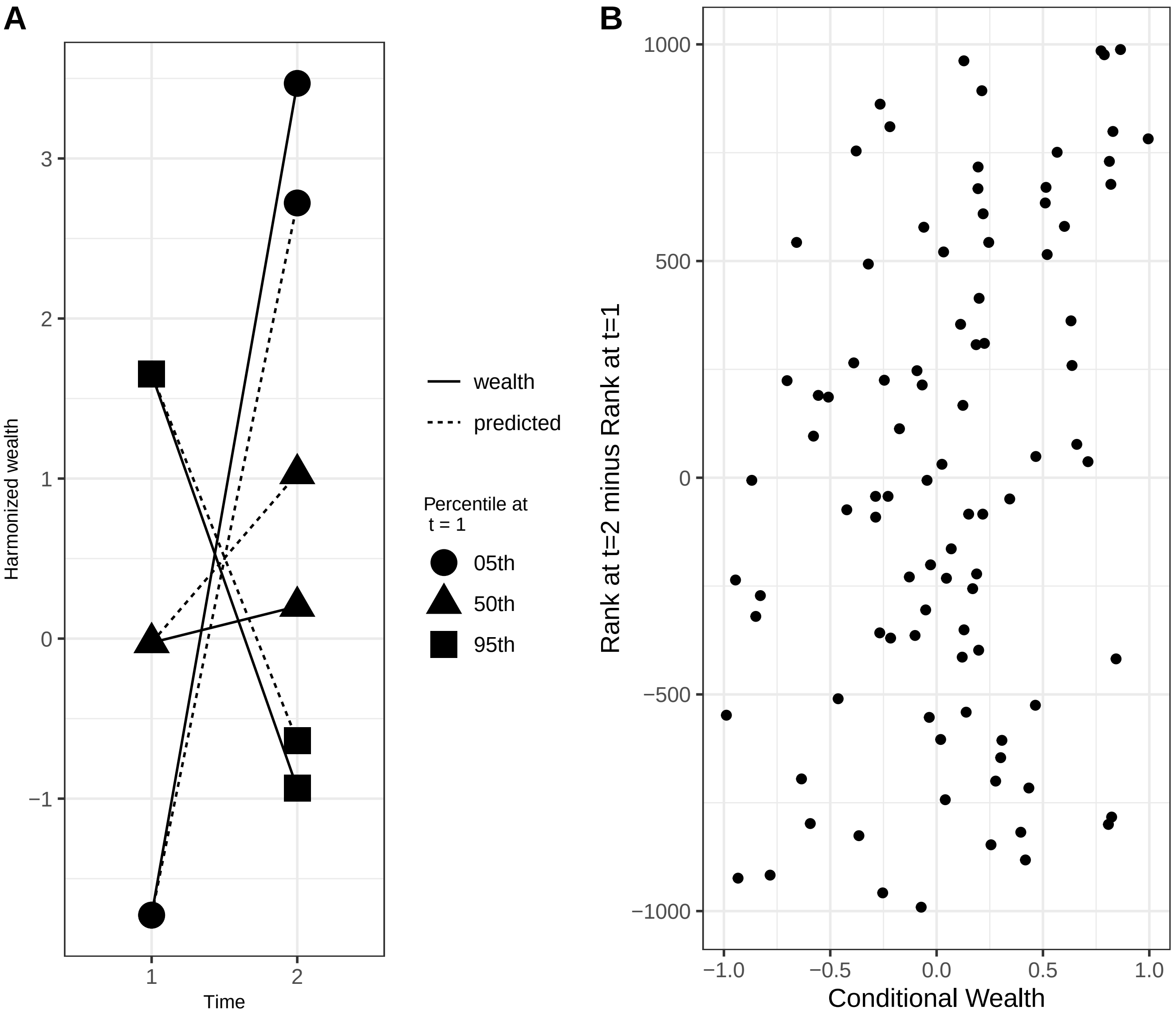

**S2Fig 43:**
**Example with w_1 = N(0,1)**
**w_2 = −1 + −0.5*w_1 + N(0,0.5)**
**Ratio of variance at time 2: time 1 = 0.5**

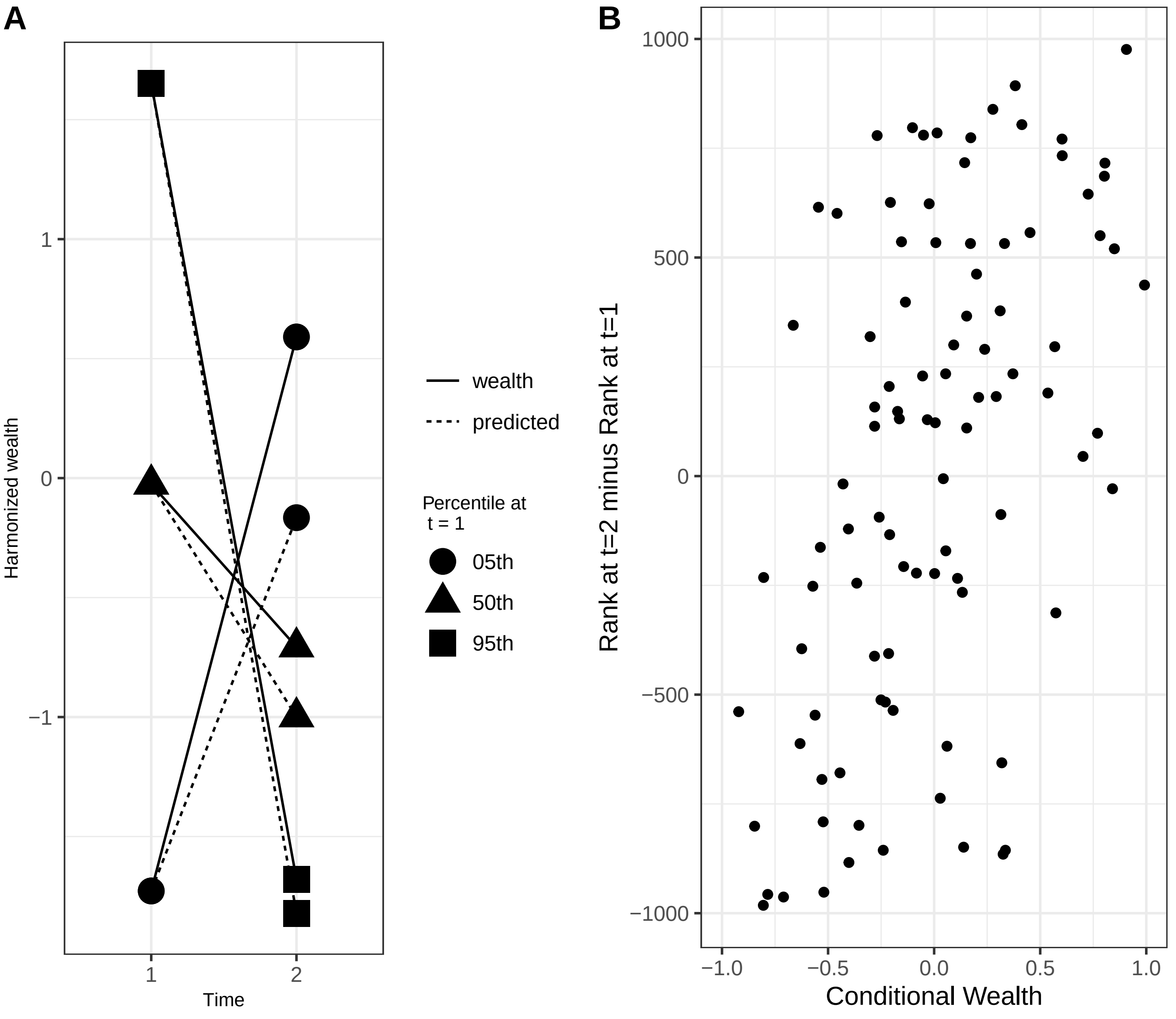

**S2Fig 44:**
**Example with w_1 = N(0,1)**
**w_2 = −0.5 + −0.5*w_1 + N(0,0.5)**
**Ratio of variance at time 2: time 1 = 0.5**

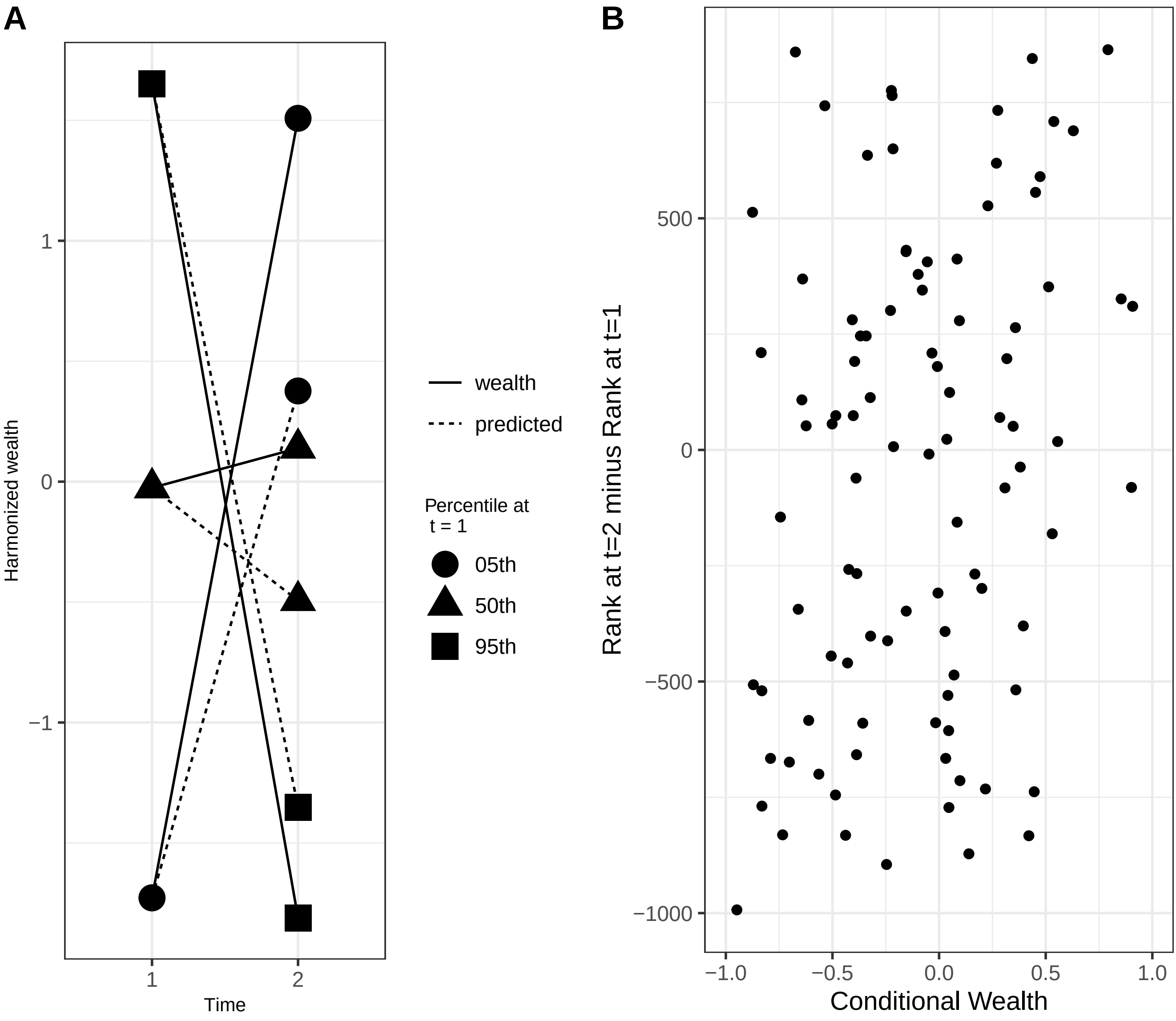

**S2Fig 45:**
**Example with w_1 = N(0,1)**
**w_2 = −0.3 + −0.5*w_1 + N(0,0.5)**
**Ratio of variance at time 2: time 1 = 0.5**

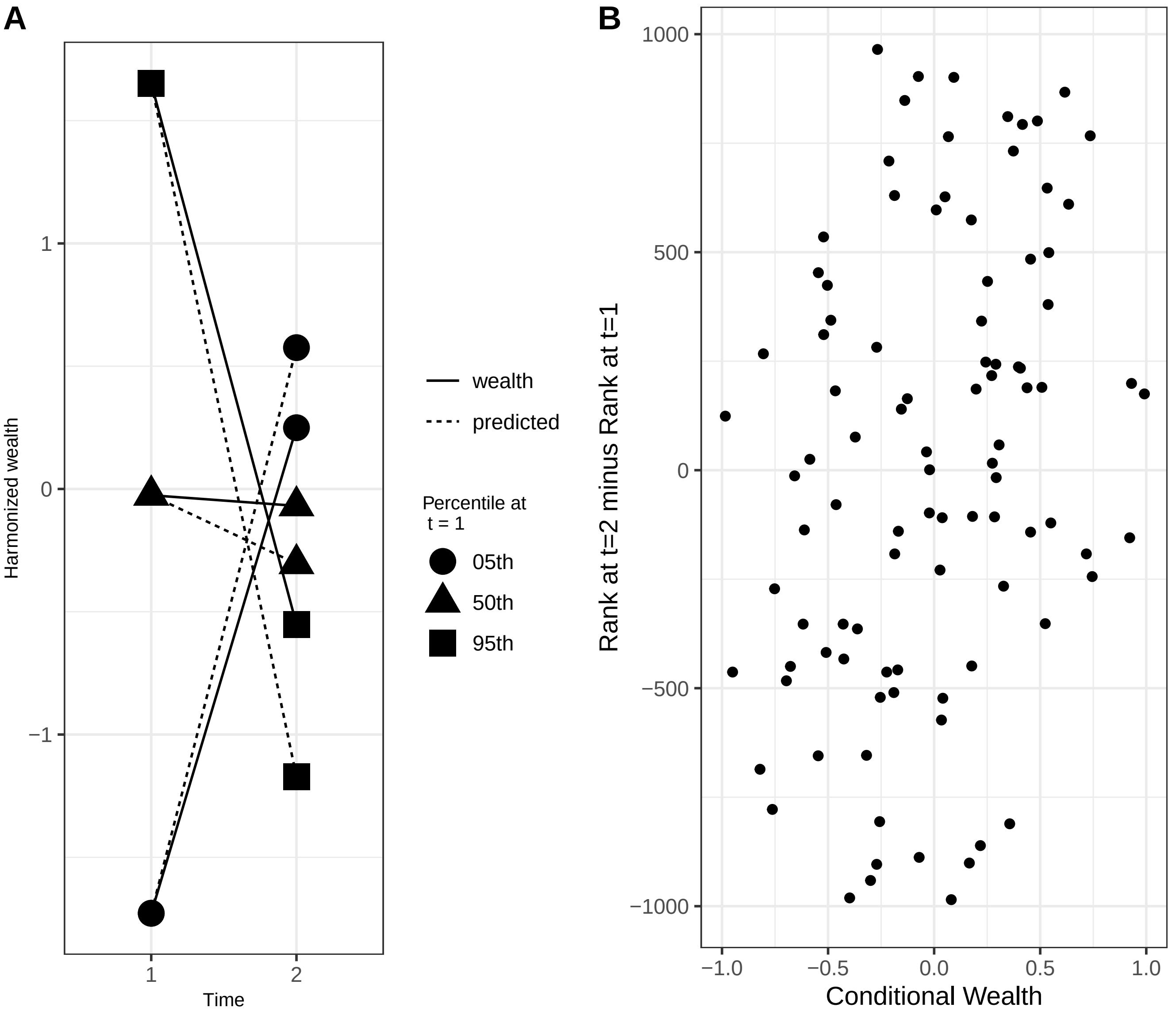

**S2Fig 46:**
**Example with w_1 = N(0,1)**
**w_2 = 0 + −0.5*w_1 + N(0,0.5)**
**Ratio of variance at time 2: time 1 = 0.5**

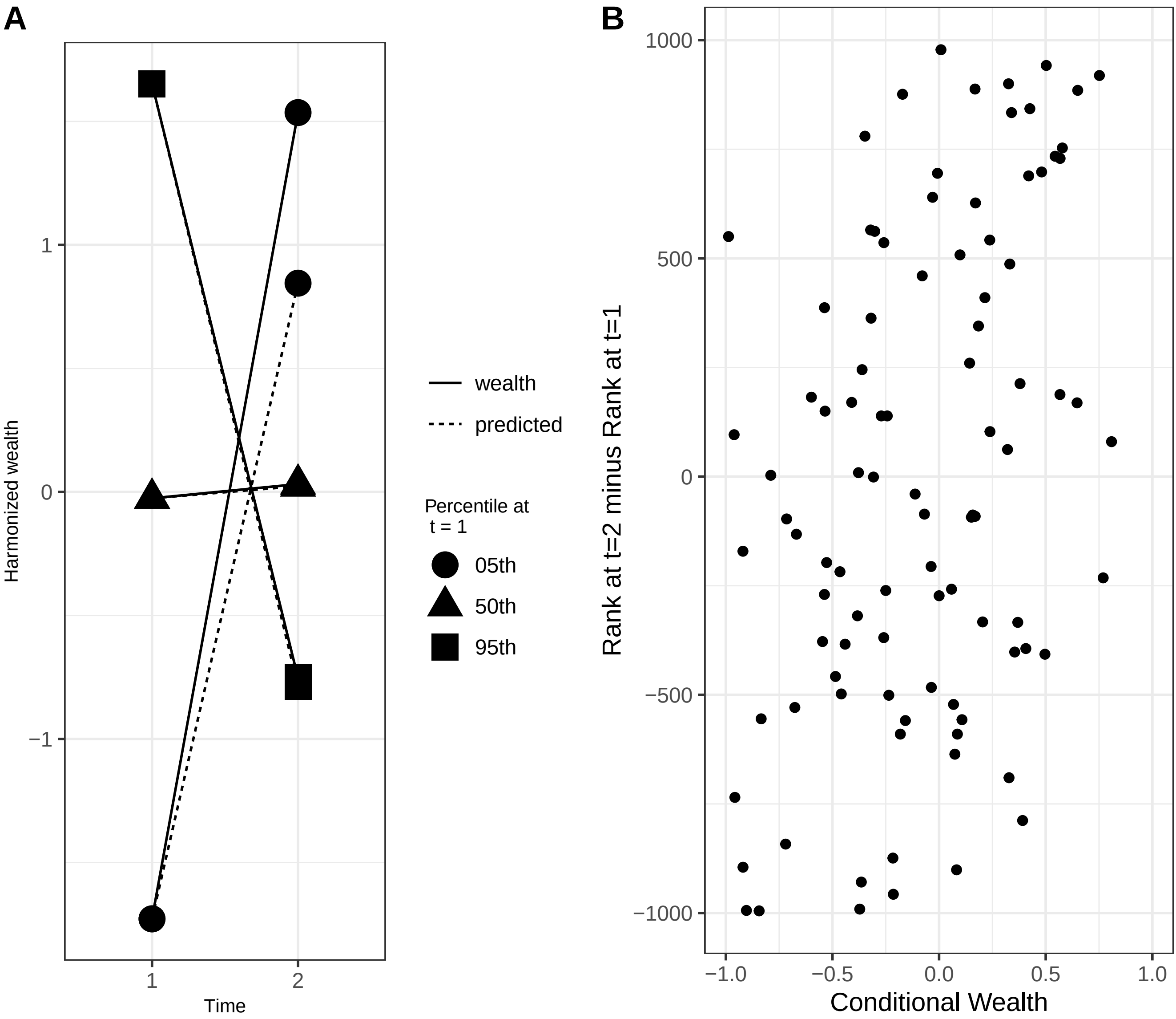

**S2Fig 47:**
**Example with w_1 = N(0,1)**
**w_2 = 0.3 + −0.5*w_1 + N(0,0.5)**
**Ratio of variance at time 2: time 1 = 0.5**

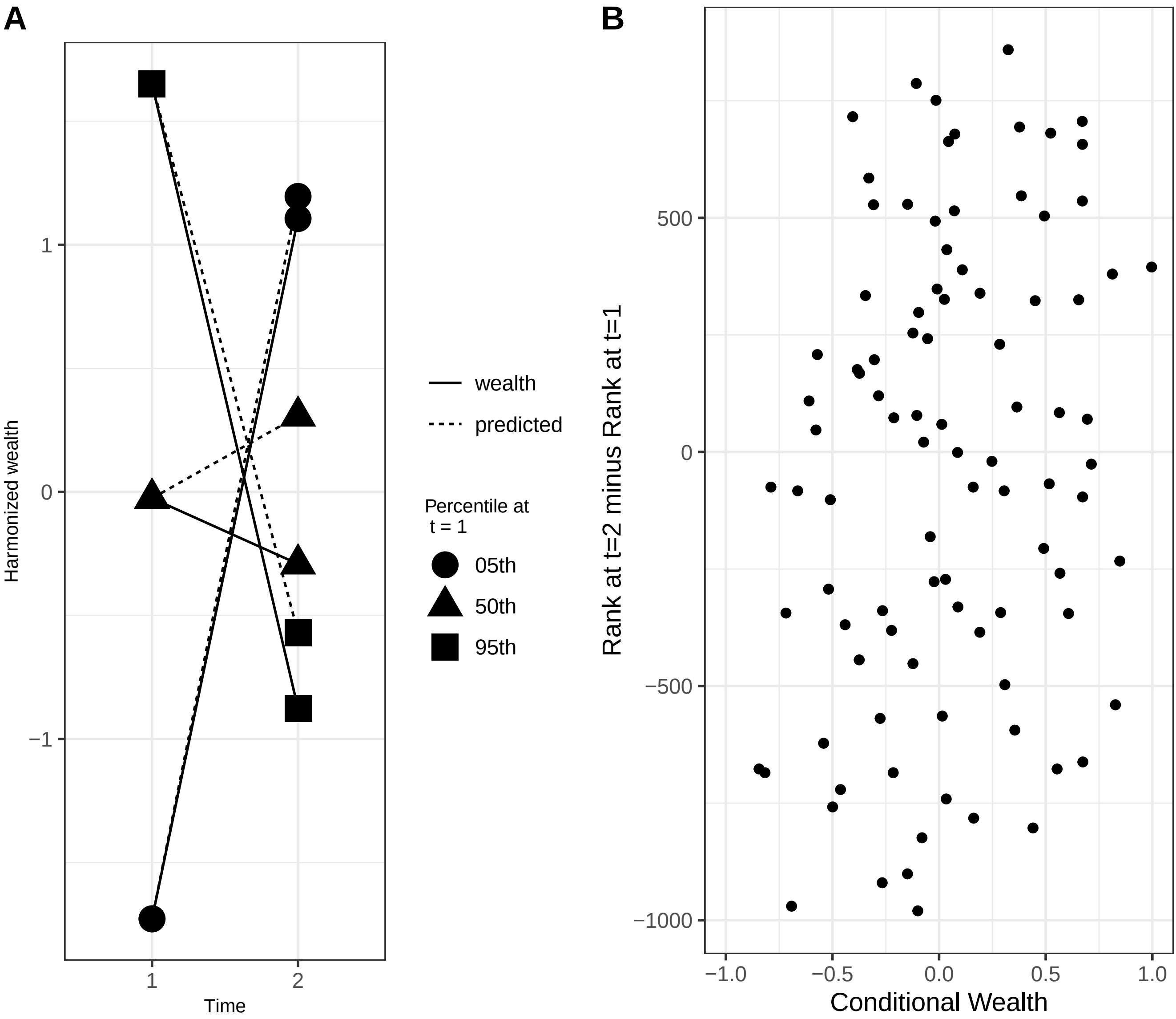

**S2Fig 48:**
**Example with w_1 = N(0,1)**
**w_2 = 0.5 + −0.5*w_1 + N(0,0.5)**
**Ratio of variance at time 2: time 1 = 0.5**

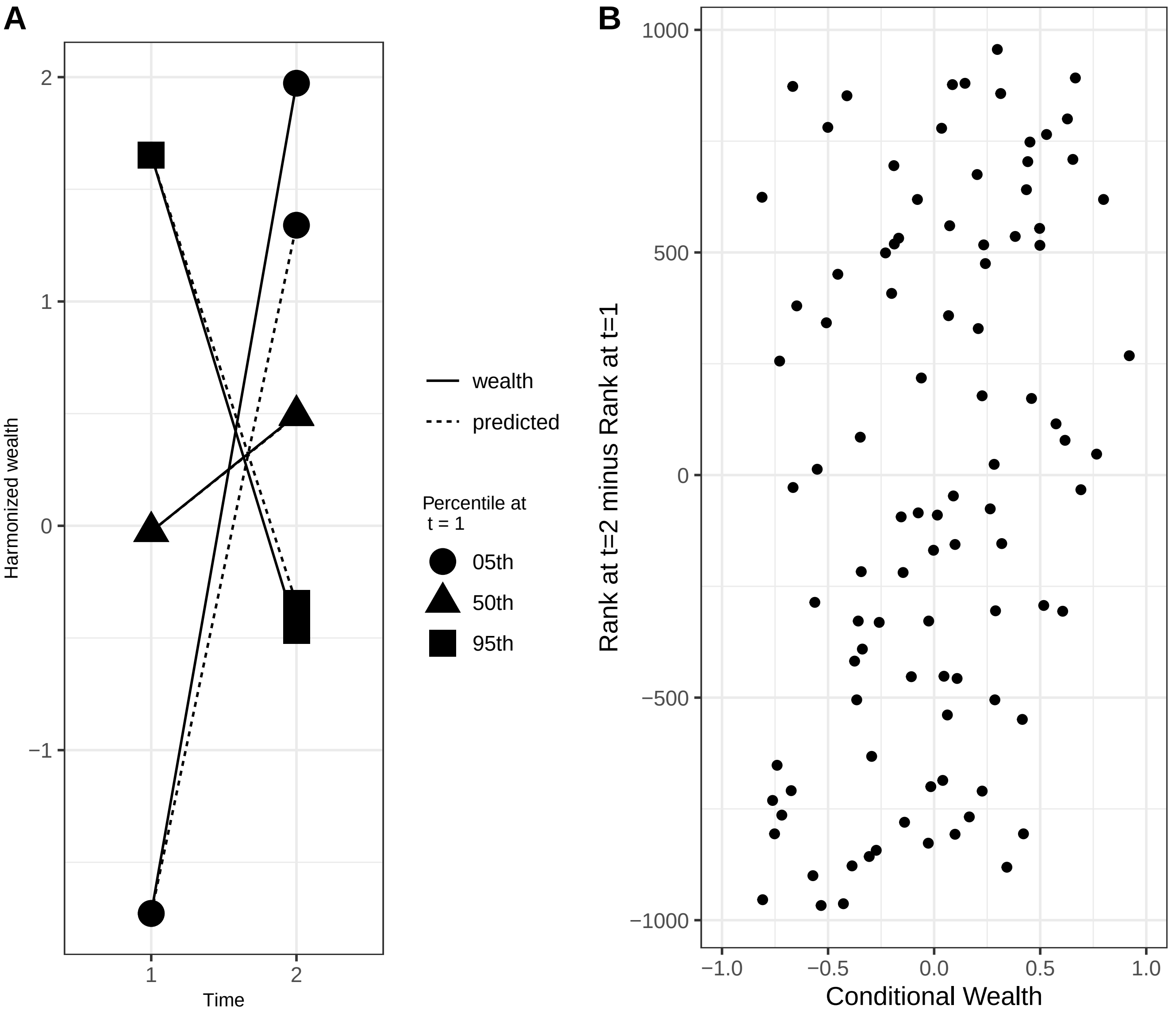

**S2Fig 49:**
**Example with w_1 = N(0,1)**
**w_2 = 1 + −0.5*w_1 + N(0,0.5)**
**Ratio of variance at time 2: time 1 = 0.5**

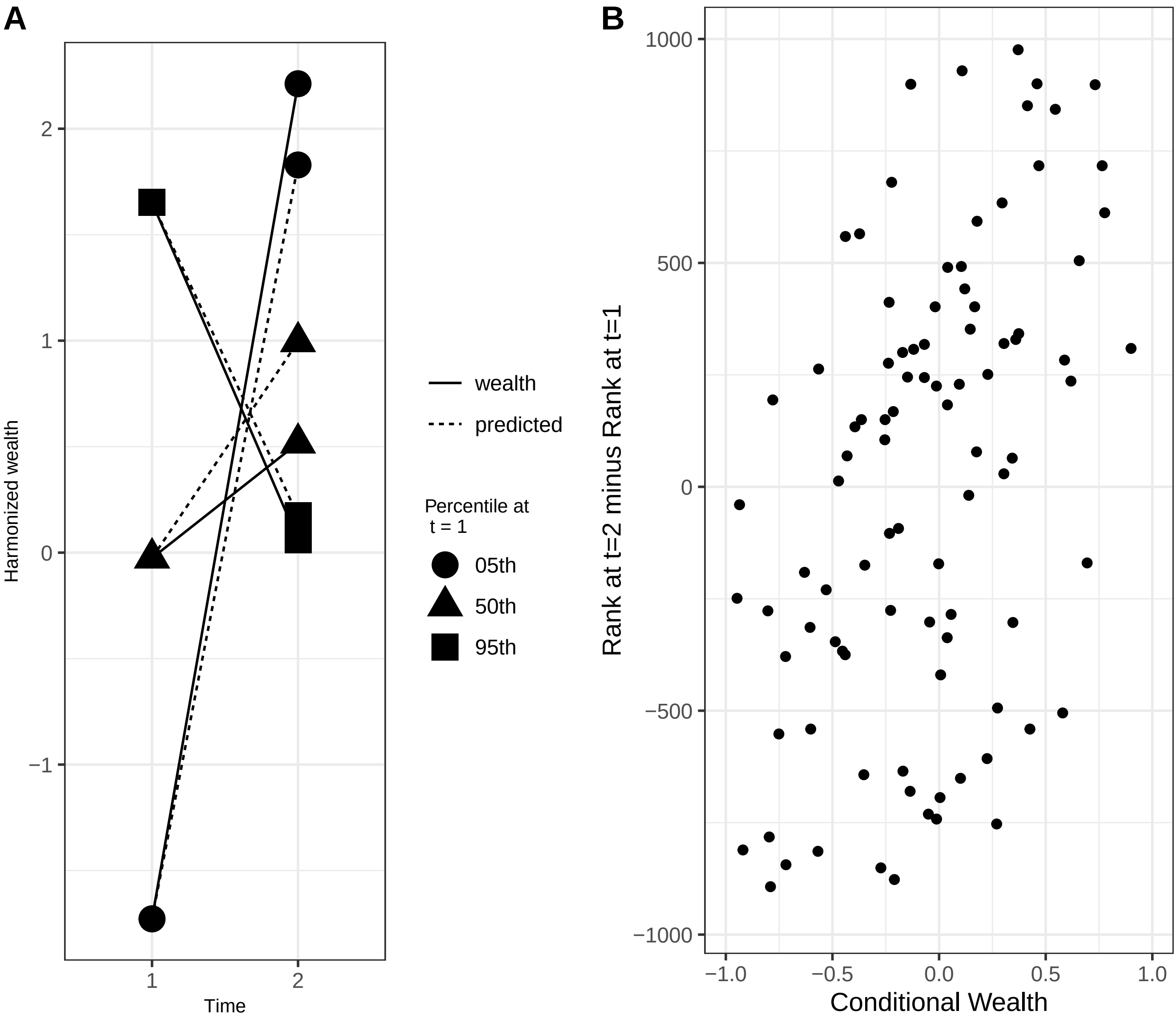

**S2Fig 50:**
**Example with w_1 = N(0,1)**
**w_2 = −1 + 0*w_1 + N(0,0.5)**
**Ratio of variance at time 2: time 1 = 0.2**

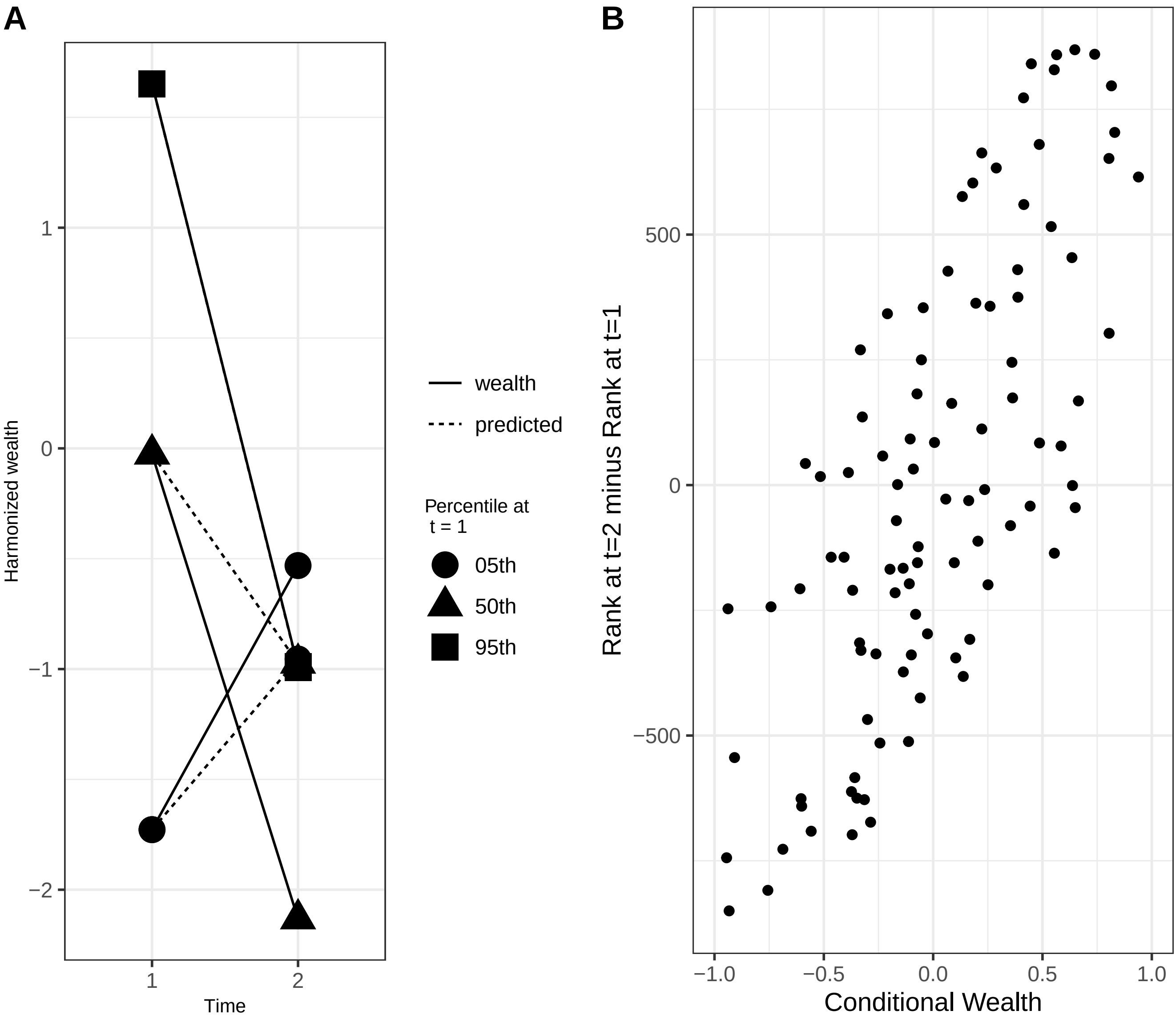

**S2Fig 51:**
**Example with w_1 = N(0,1)**
**w_2 = −0.5 + 0*w_1 + N(0,0.5)**
**Ratio of variance at time 2: time 1 = 0.3**

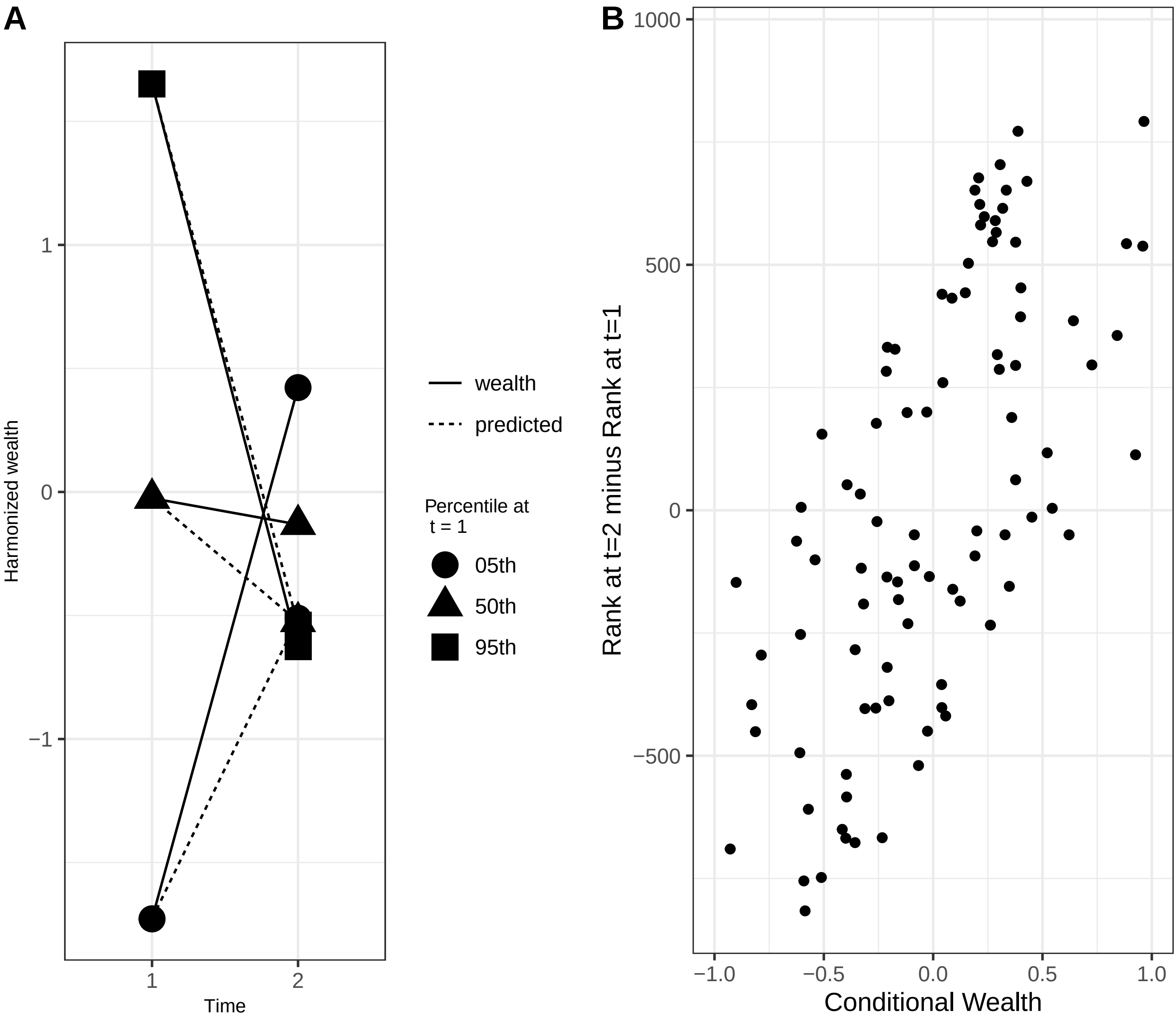

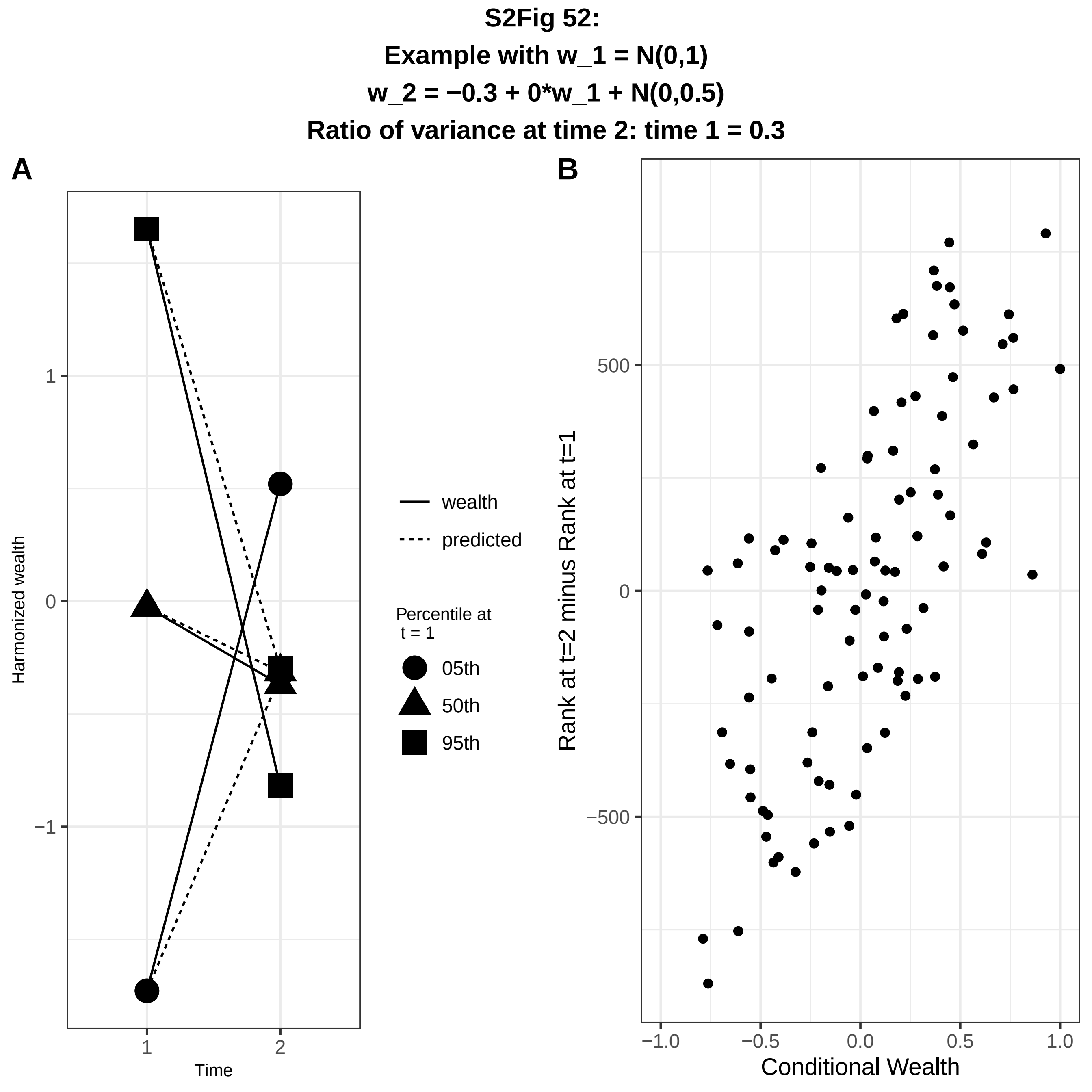
S2Fig 52:
Example with w_1 = N(0,1)
w_2 = −0.3 + 0*w_1 + N(0,0.5)
Ratio of variance at time 2: time 1 = 0.3
A
Harmonized wealth
Time
1
0
−1
1
2
wealth
predicted
Percentile at
t = 1
05th
50th
95th
B
Rank at t=2 minus Rank at t=1
Conditional Wealth
500
0
−500
−1.0
−0.5
0.0
0.5
1.0

**S2Fig 53:**
**Example with w_1 = N(0,1)**
**w_2 = 0 + 0*w_1 + N(0,0.5)**
**Ratio of variance at time 2: time 1 = 0.2**

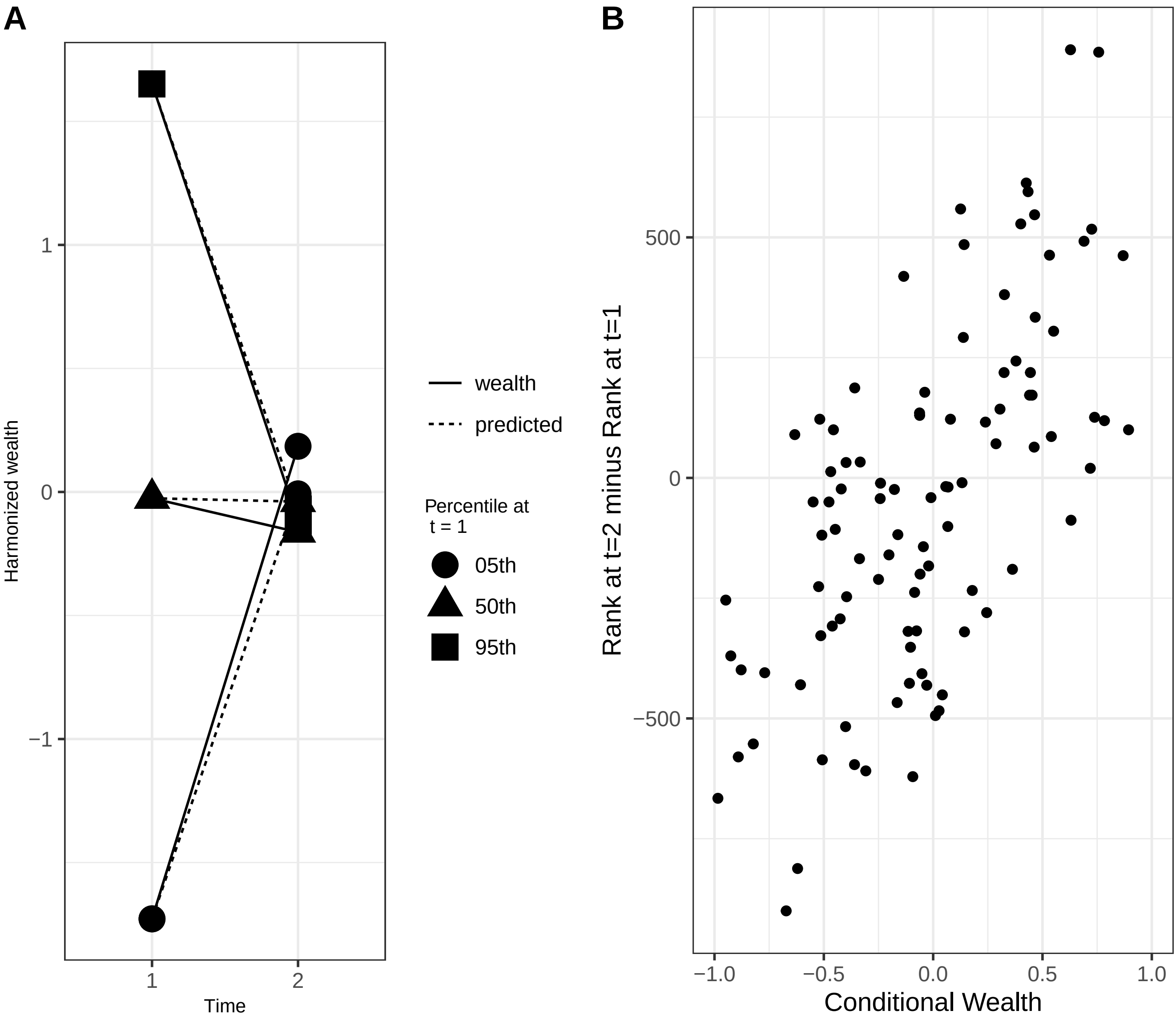

**S2Fig 54:**
**Example with w_1 = N(0,1)**
**w_2 = 0.3 + 0*w_1 + N(0,0.5)**
**Ratio of variance at time 2: time 1 = 0.2**

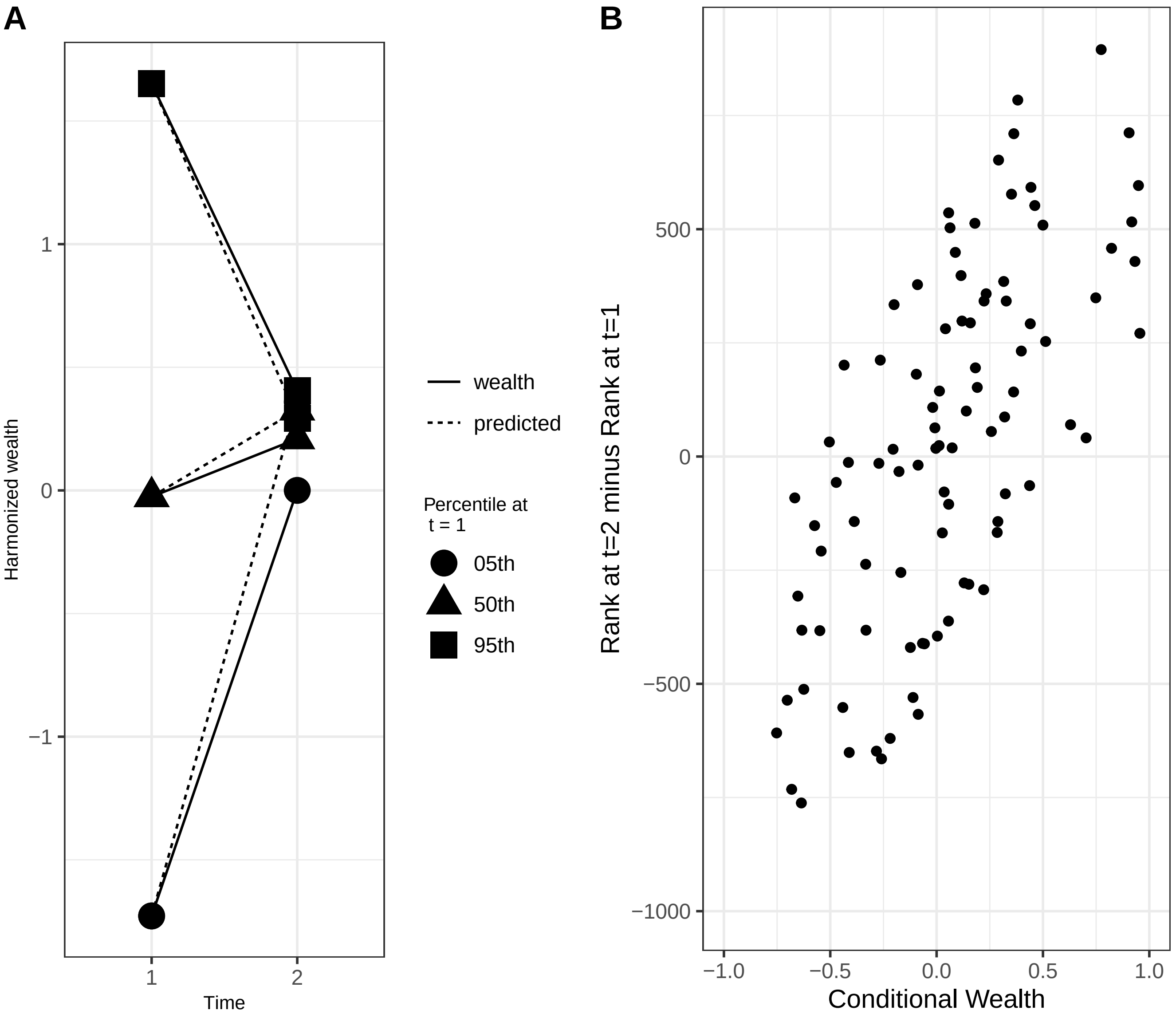

**S2Fig 55:**
**Example with w_1 = N(0,1)**
**w_2 = 0.5 + 0*w_1 + N(0,0.5)**
**Ratio of variance at time 2: time 1 = 0.3**

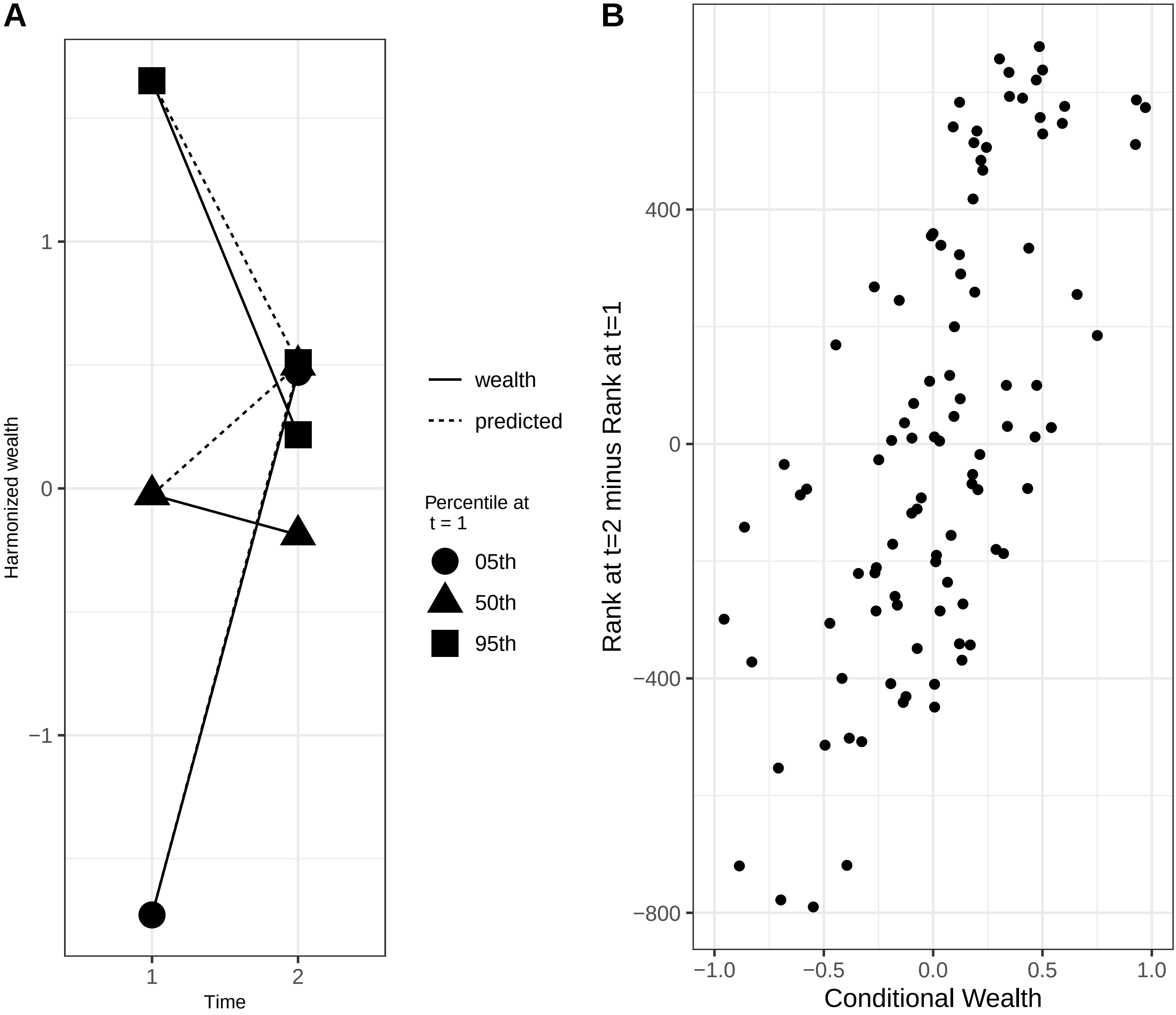

**S2Fig 56:**
**Example with w_1 = N(0,1)**
**w_2 = 1 + 0*w_1 + N(0,0.5)**
**Ratio of variance at time 2: time 1 = 0.3**

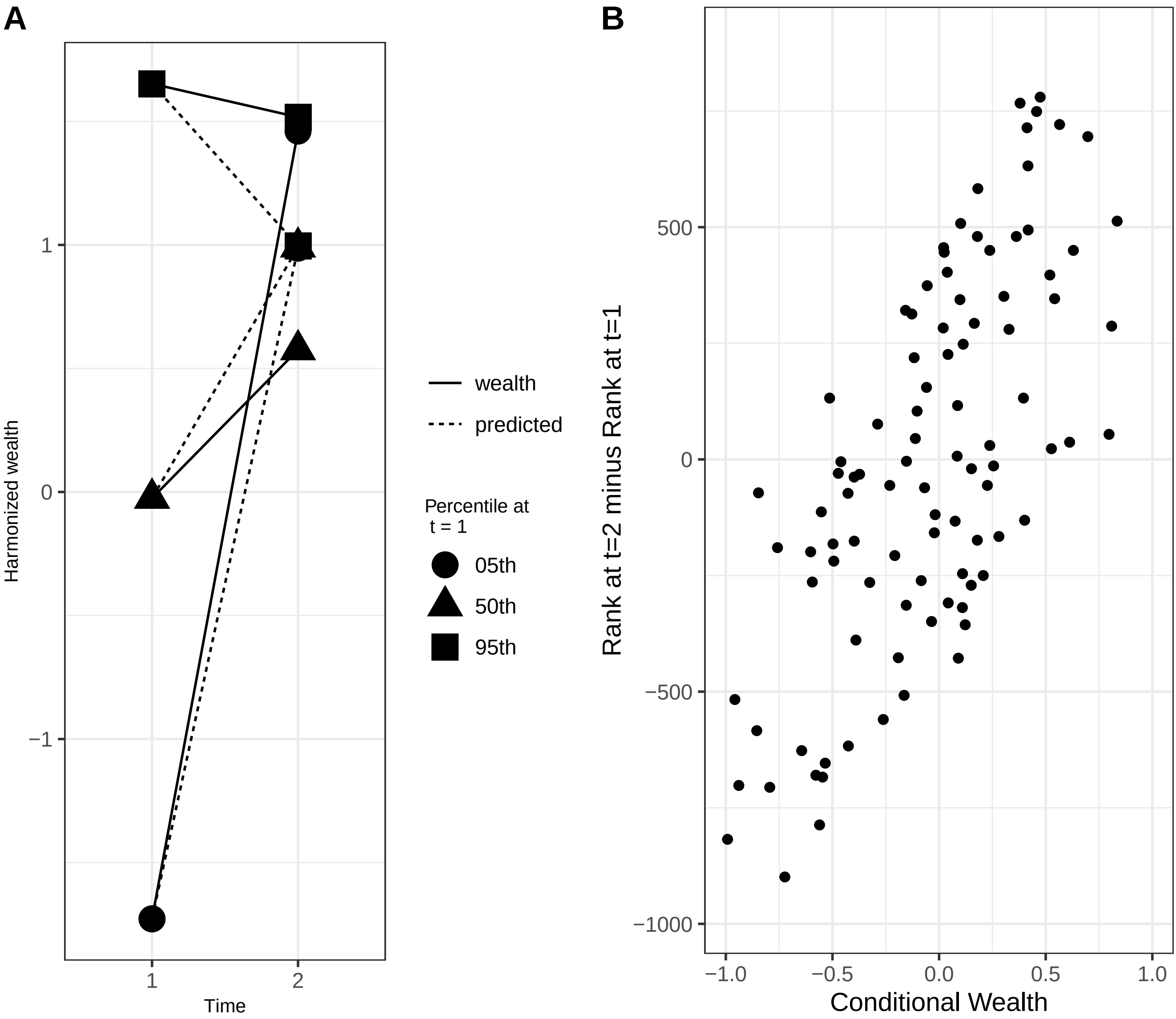

**S2Fig 57:**
**Example with w_1 = N(0,1)**
**w_2 = −1 + 0.5*w_1 + N(0,0.5)**
**Ratio of variance at time 2: time 1 = 0.5**

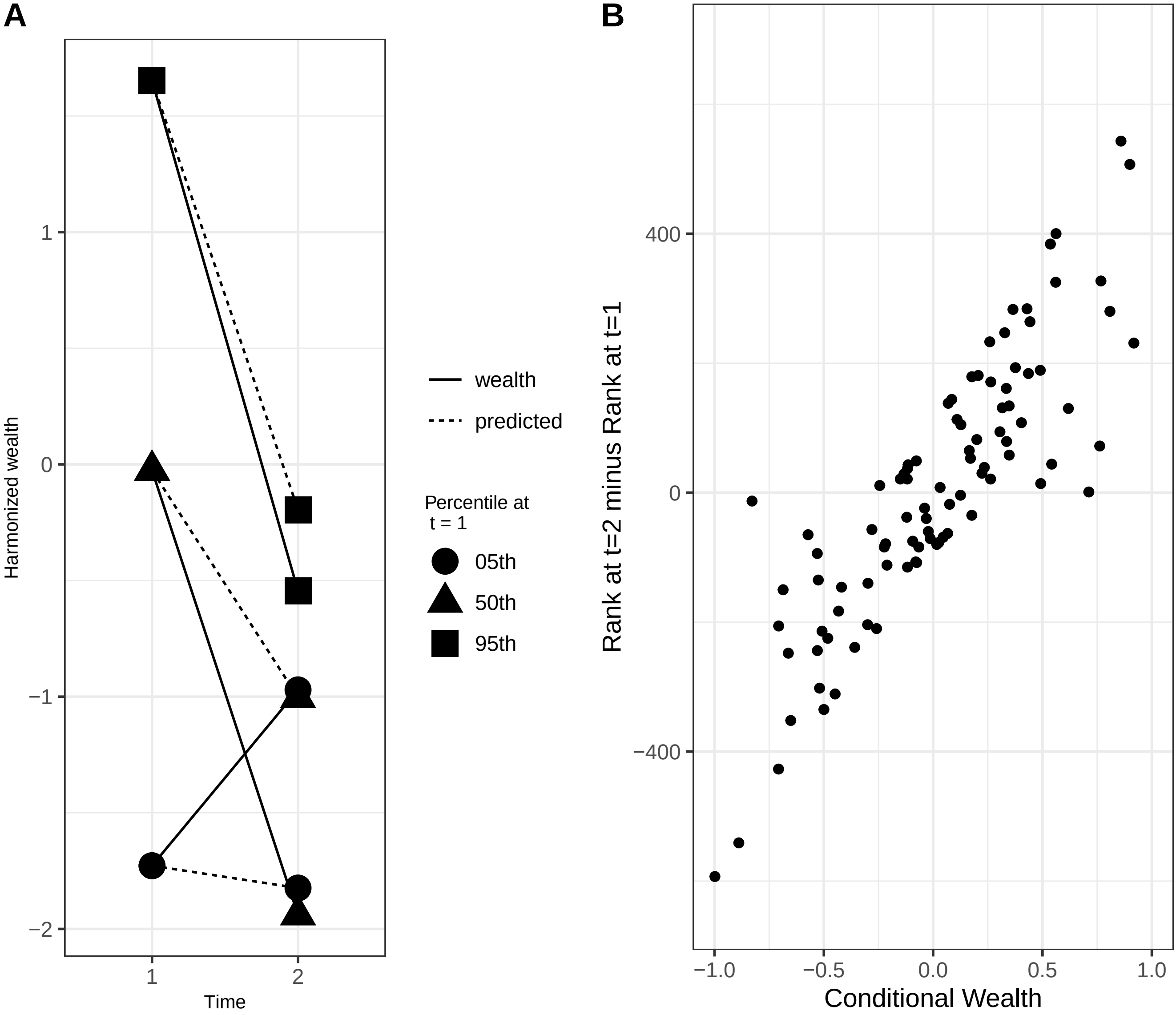

**S2Fig 58:**
**Example with w_1 = N(0,1)**
**w_2 = −0.5 + 0.5*w_1 + N(0,0.5)**
**Ratio of variance at time 2: time 1 = 0.5**

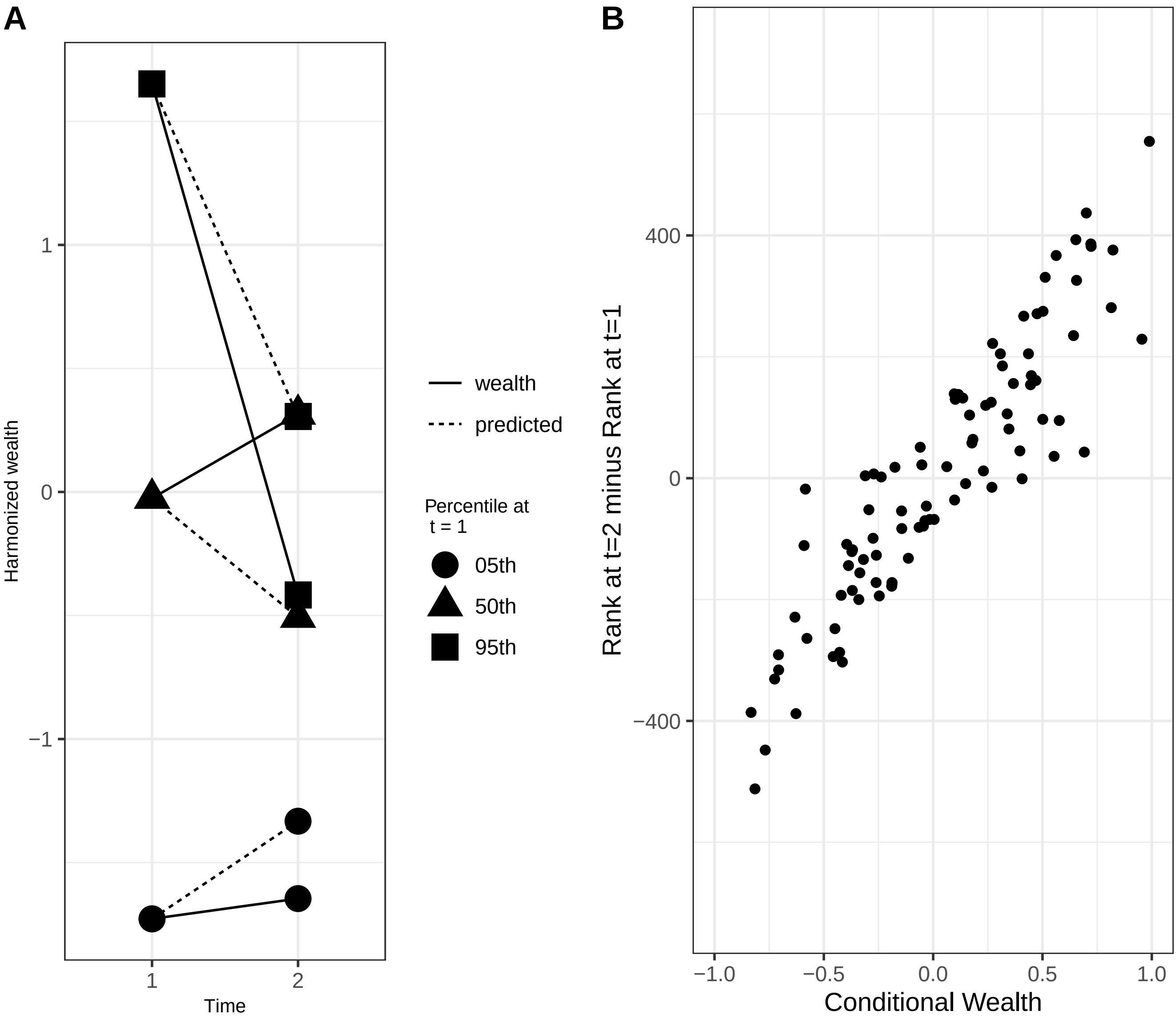

**S2Fig 59:**
**Example with w_1 = N(0,1)**
**w_2 = −0.3 + 0.5*w_1 + N(0,0.5)**
**Ratio of variance at time 2: time 1 = 0.5**

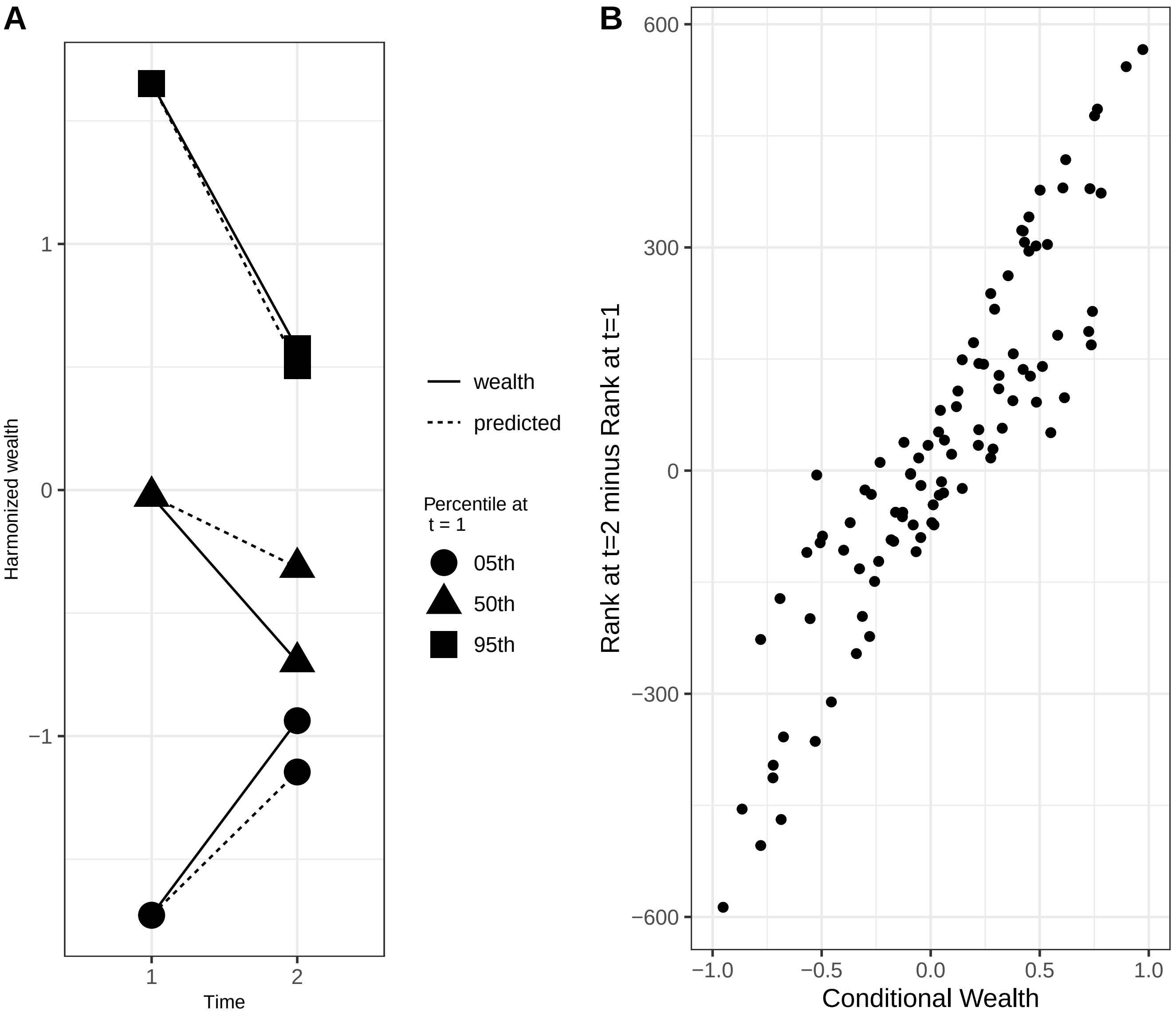

**S2Fig 60:**
**Example with w_1 = N(0,1)**
**w_2 = 0 + 0.5*w_1 + N(0,0.5)**
**Ratio of variance at time 2: time 1 = 0.5**

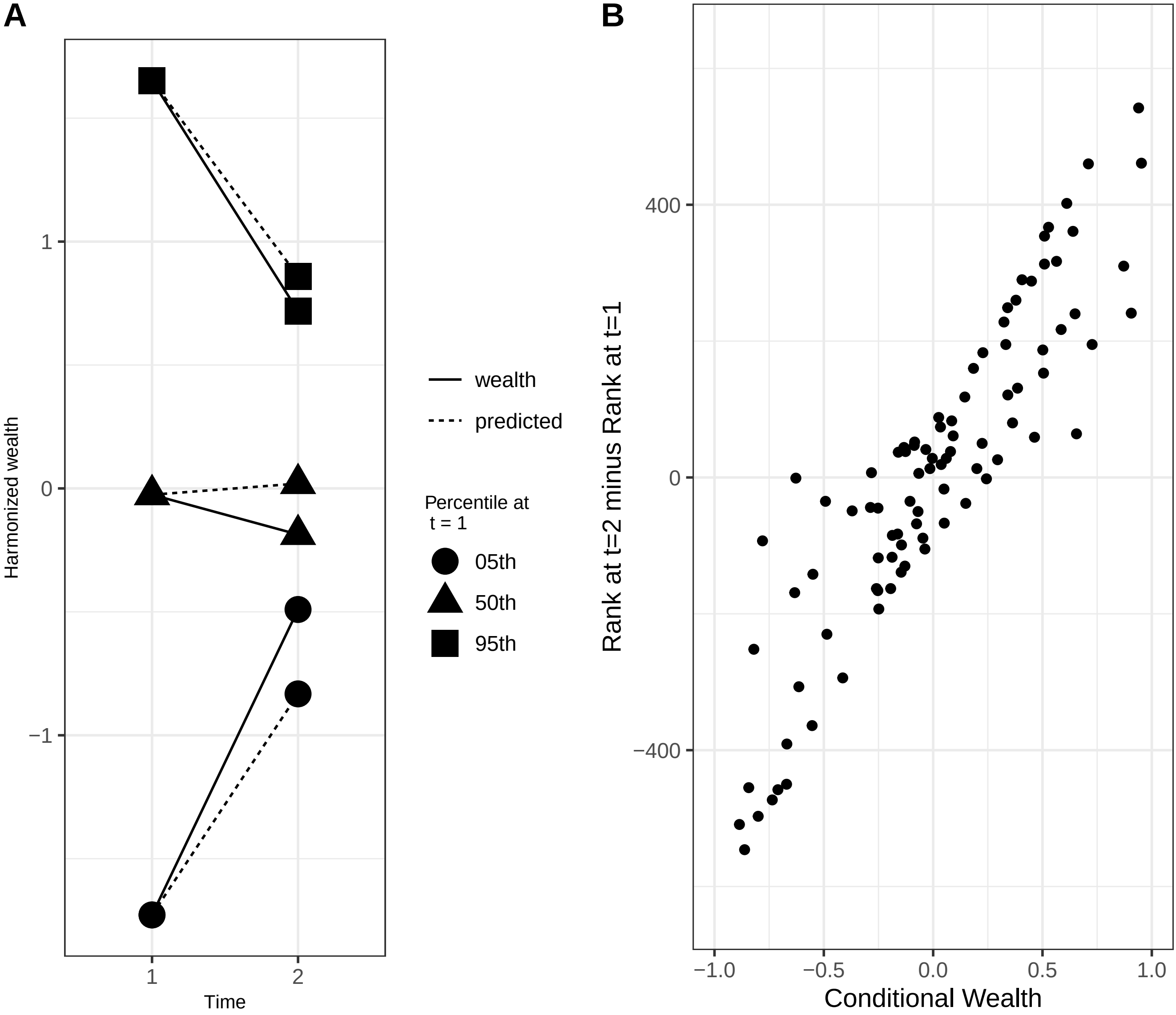

**S2Fig 61:**
**Example with w_1 = N(0,1)**
**w_2 = 0.3 + 0.5*w_1 + N(0,0.5)**
**Ratio of variance at time 2: time 1 = 0.5**

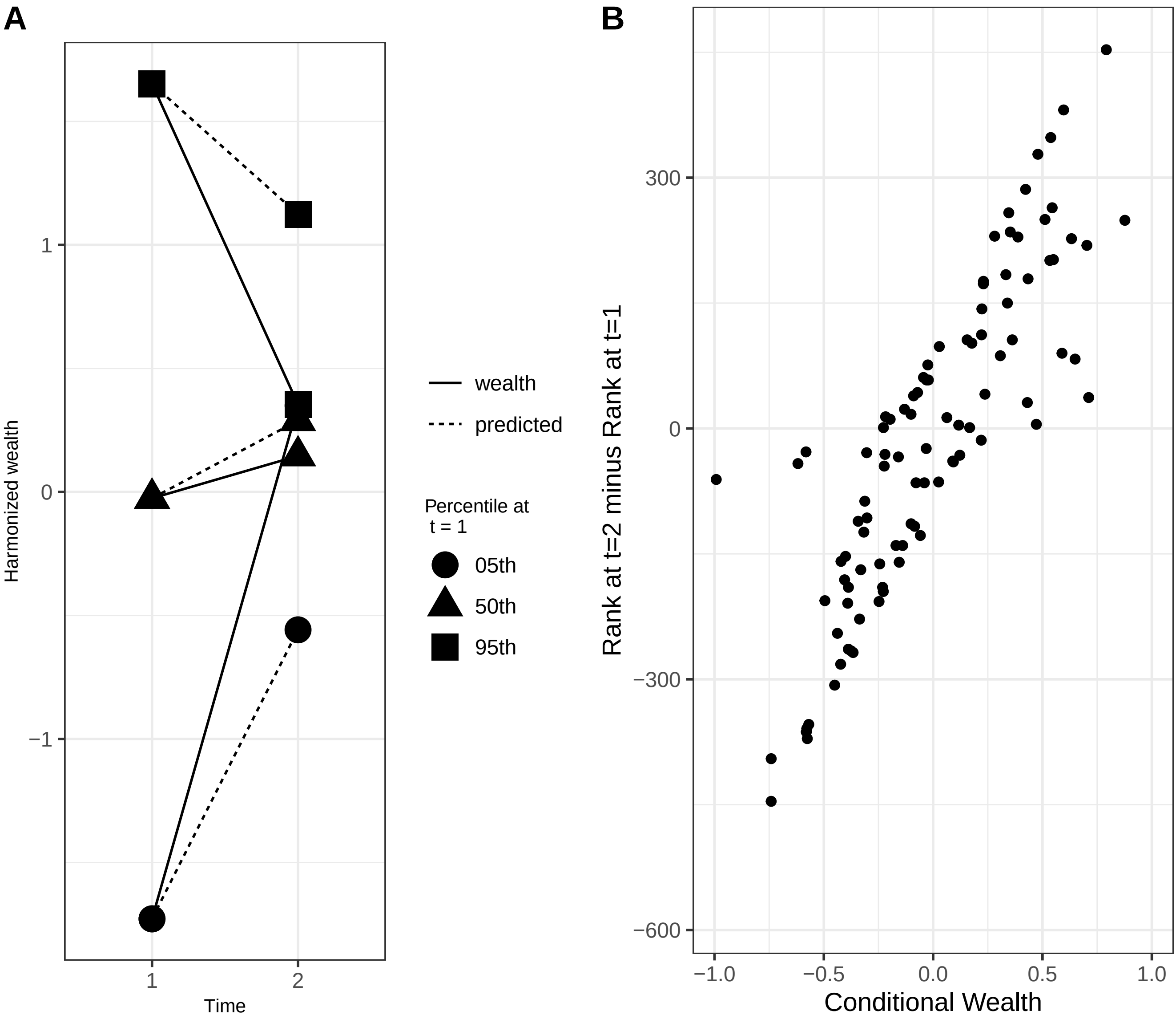

**S2Fig 62:**
**Example with w_1 = N(0,1)**
**w_2 = 0.5 + 0.5*w_1 + N(0,0.5)**
**Ratio of variance at time 2: time 1 = 0.5**

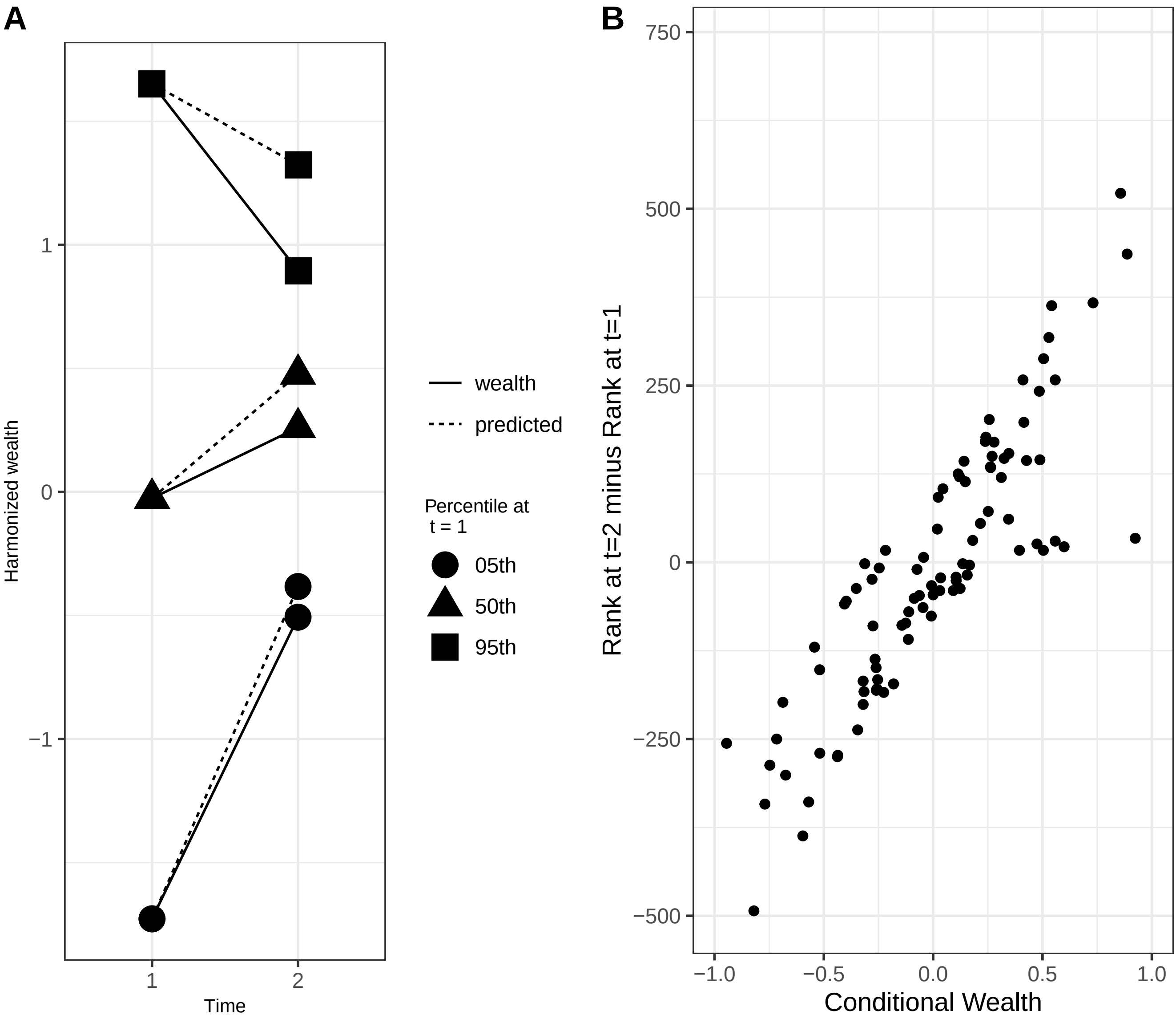

**S2Fig 63:**
**Example with w_1 = N(0,1)**
**w_2 = 1 + 0.5*w_1 + N(0,0.5)**
**Ratio of variance at time 2: time 1 = 0.6**

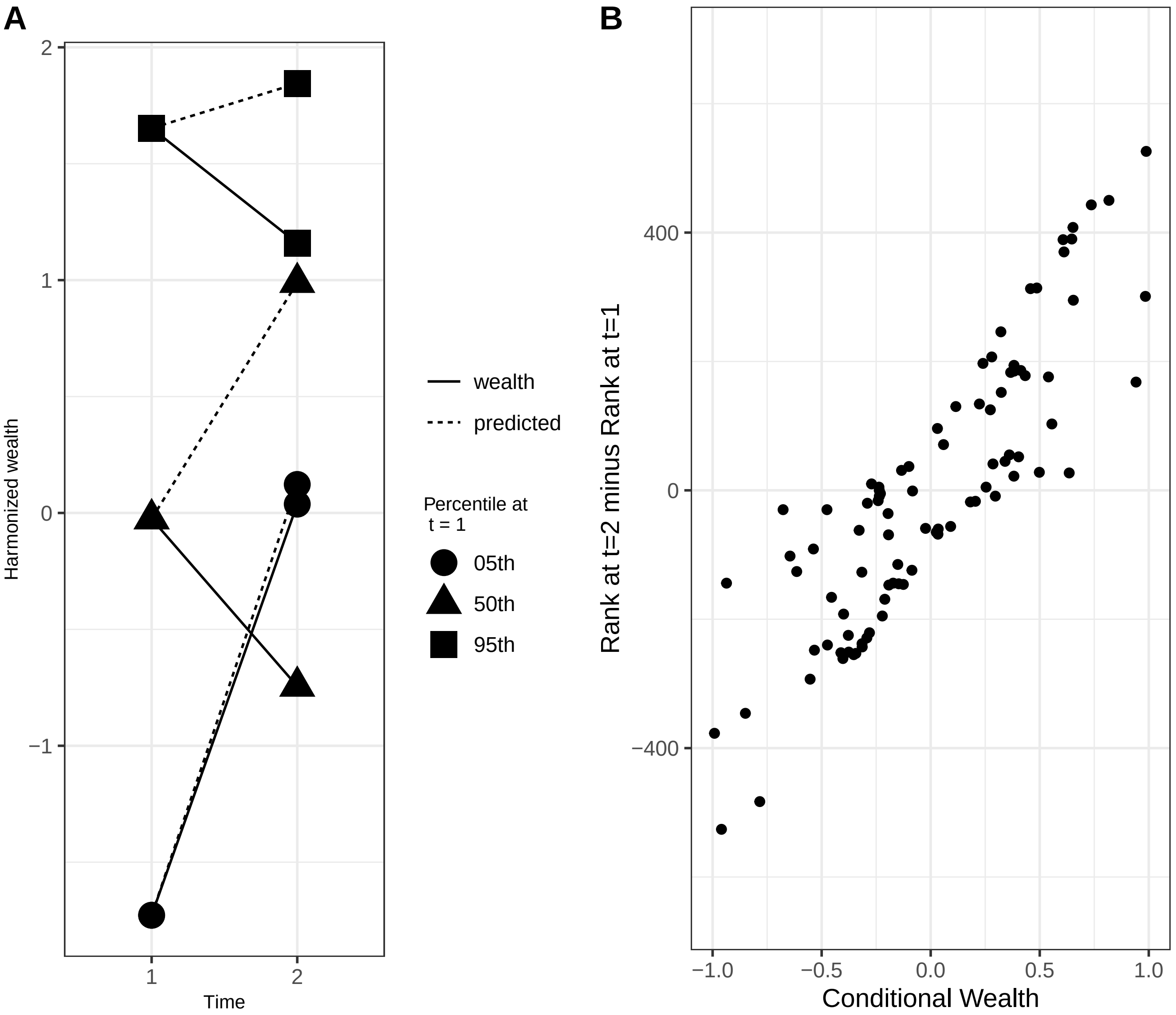

**S2Fig 64:**
**Example with w_1 = N(0,1)**
**w_2 = −1 + 1*w_1 + N(0,0.5)**
**Ratio of variance at time 2: time 1 = 1.3**

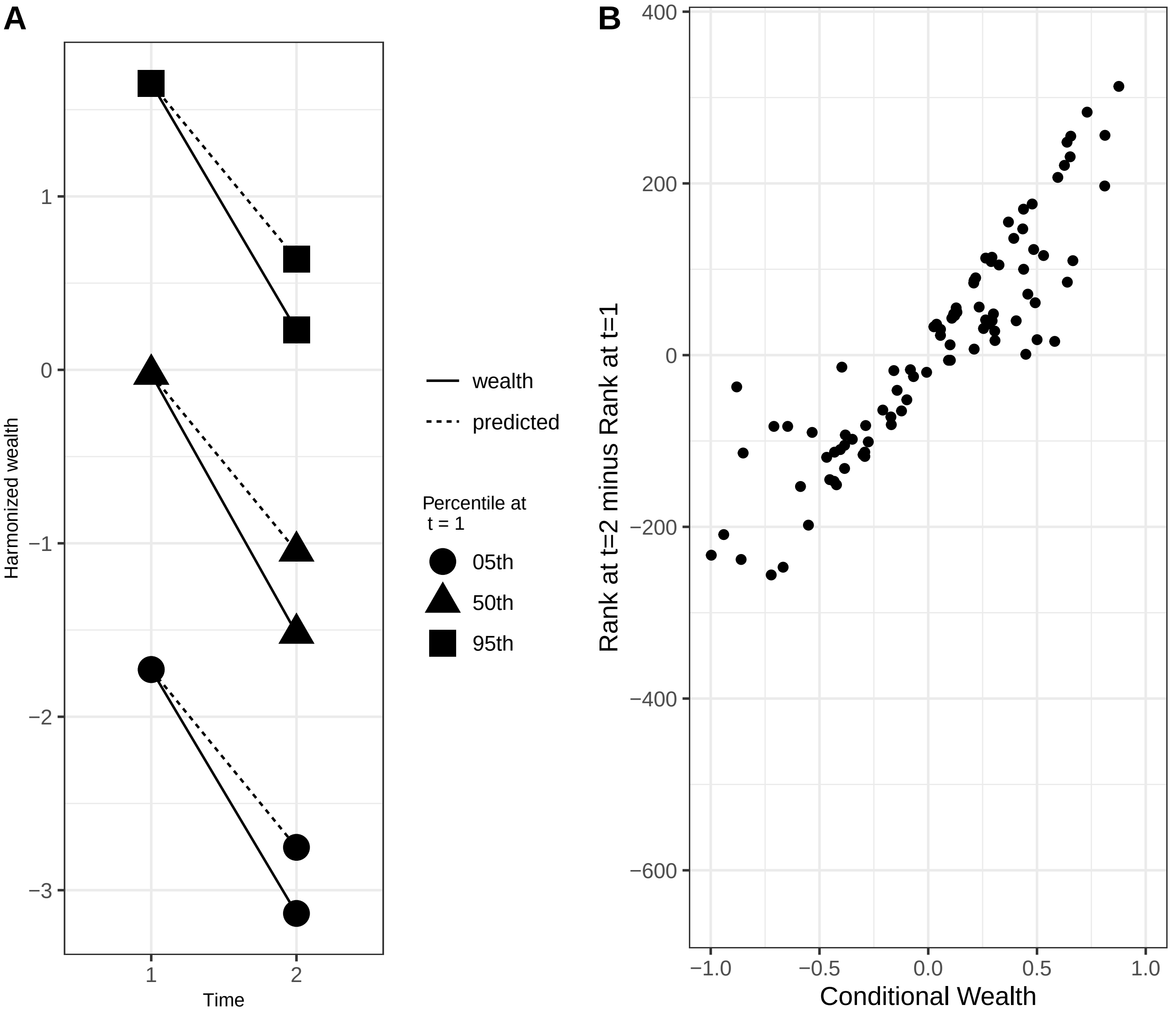

**S2Fig 65:**
**Example with w_1 = N(0,1)**
**w_2 = −0.5 + 1*w_1 + N(0,0.5)**
**Ratio of variance at time 2: time 1 = 1.3**

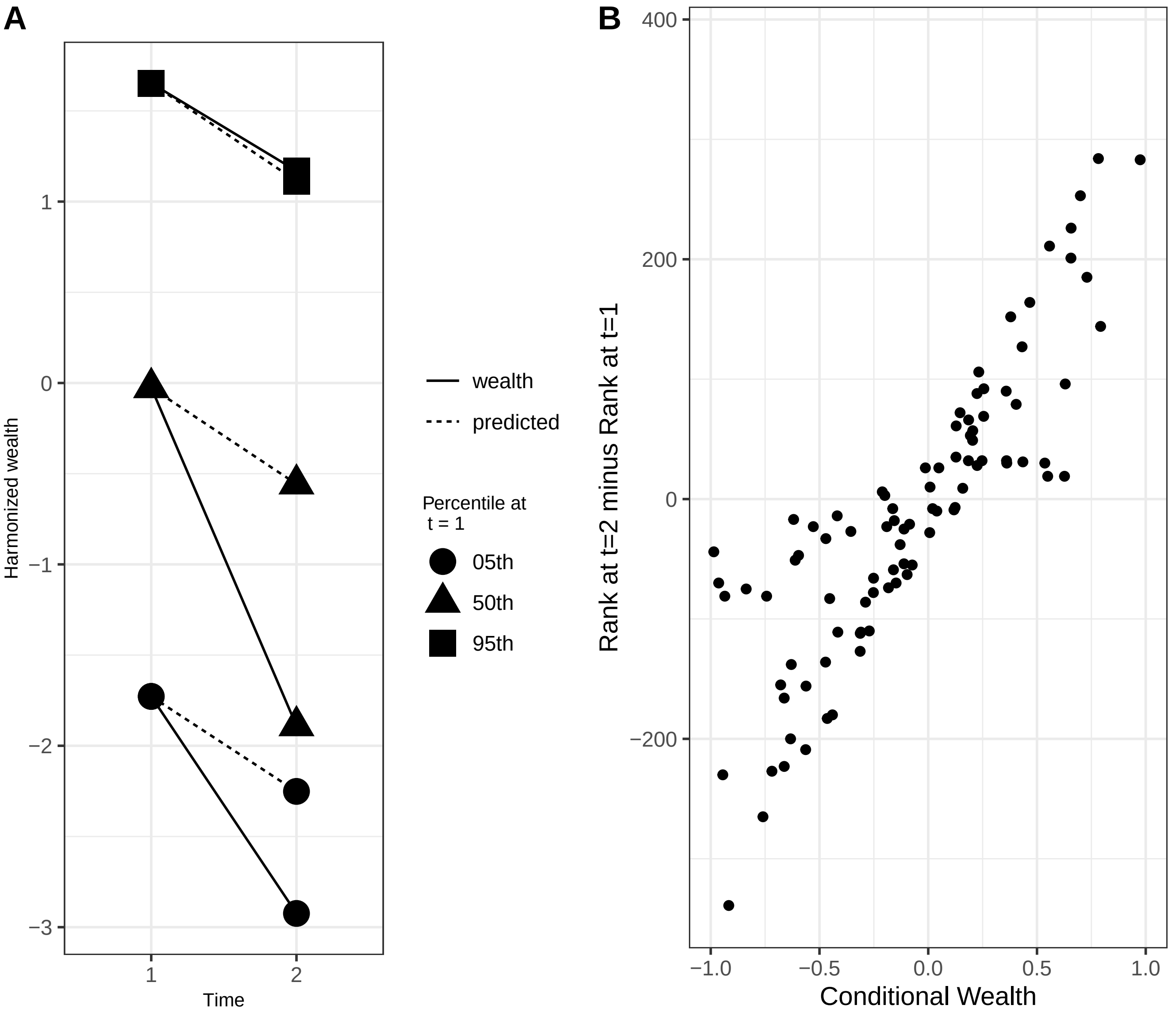

**S2Fig 66:**
**Example with w_1 = N(0,1)**
**w_2 = −0.3 + 1*w_1 + N(0,0.5)**
**Ratio of variance at time 2: time 1 = 1.2**

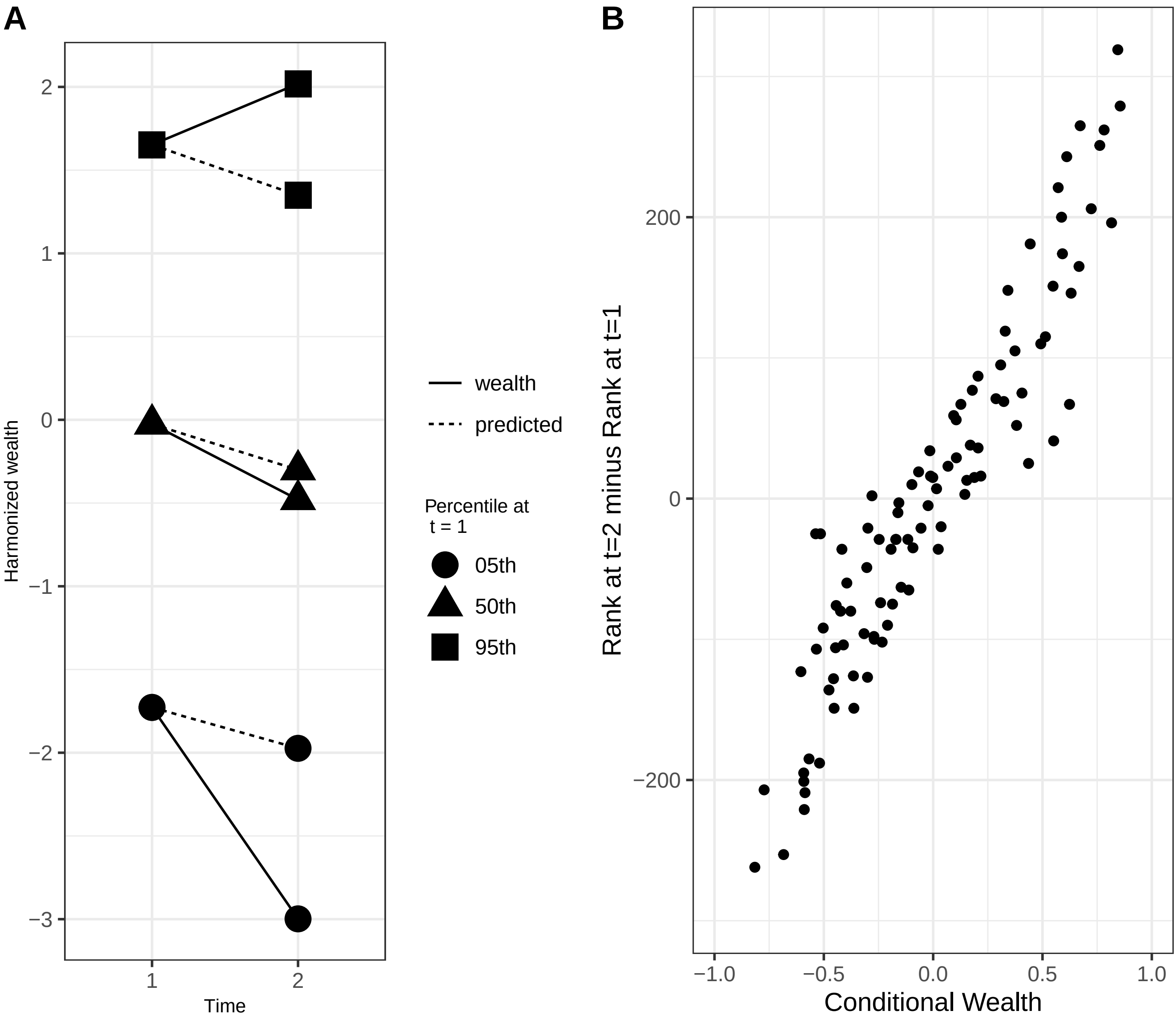

**S2Fig 67:**
**Example with w_1 = N(0,1)**
**w_2 = 0 + 1*w_1 + N(0,0.5)**
**Ratio of variance at time 2: time 1 = 1.3**

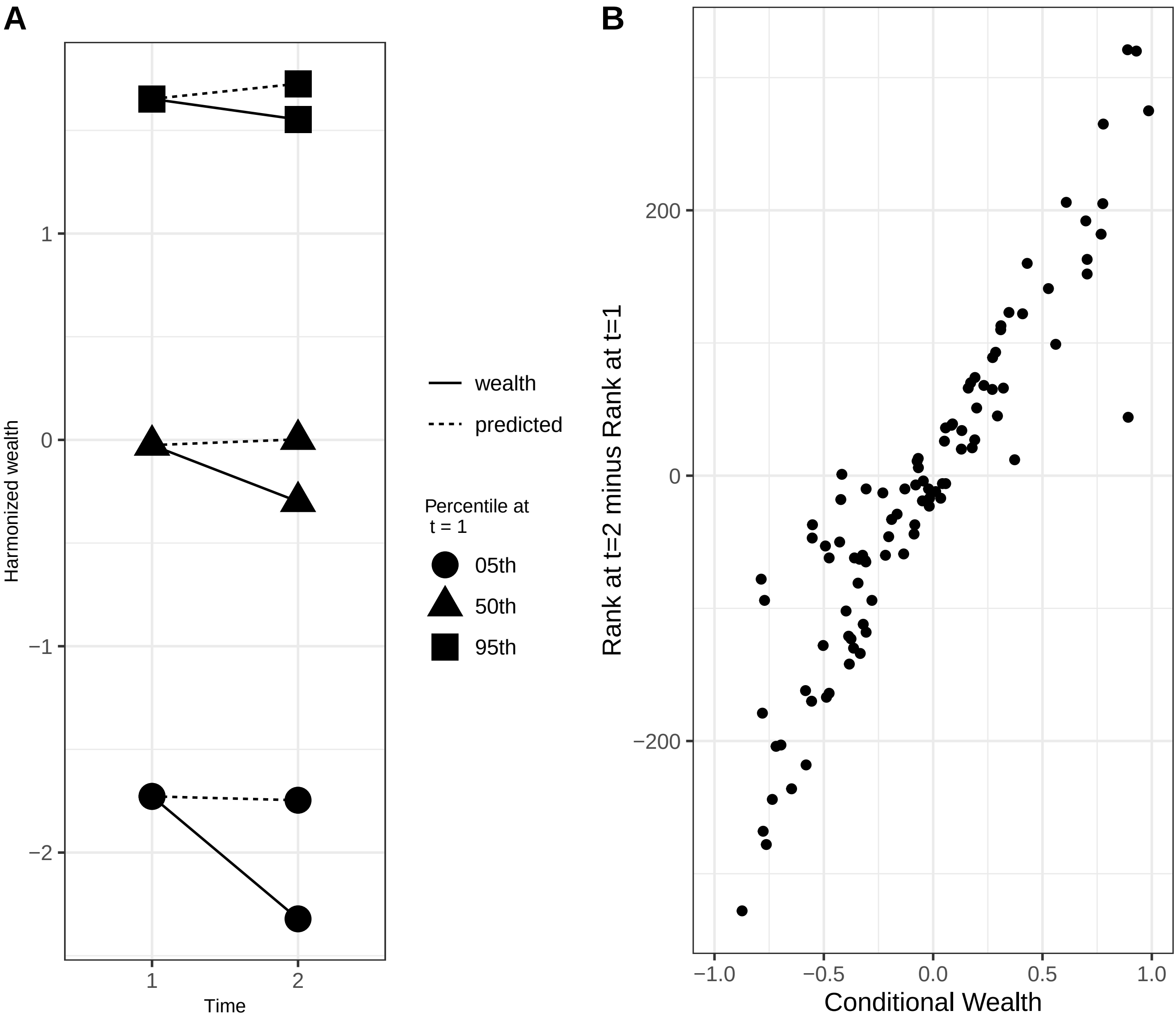

**S2Fig 68:**
**Example with w_1 = N(0,1)**
**w_2 = 0.3 + 1*w_1 + N(0,0.5)**
**Ratio of variance at time 2: time 1 = 1.3**

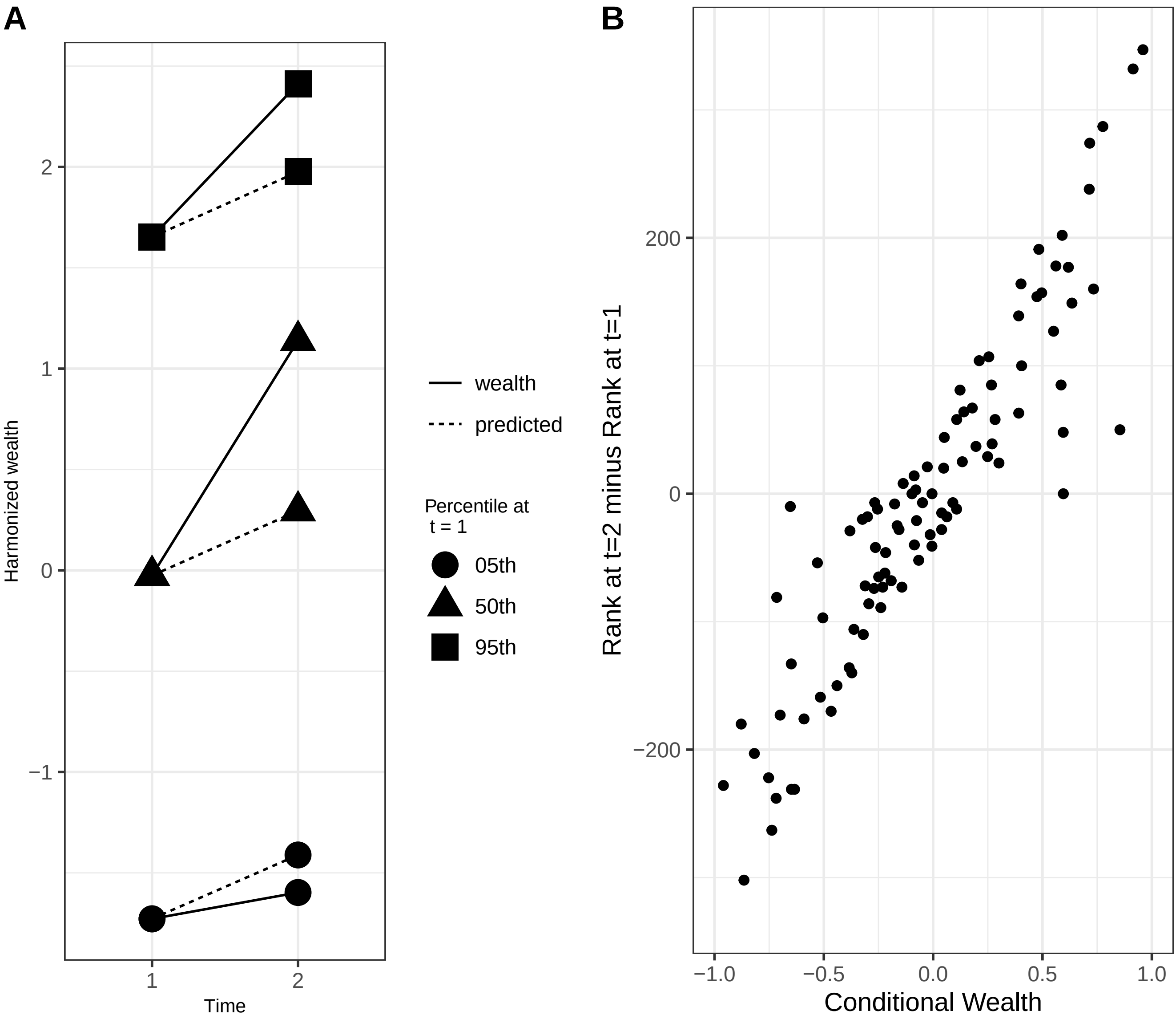

**S2Fig 69:**
**Example with w_1 = N(0,1)**
**w_2 = 0.5 + 1*w_1 + N(0,0.5)**
**Ratio of variance at time 2: time 1 = 1.3**

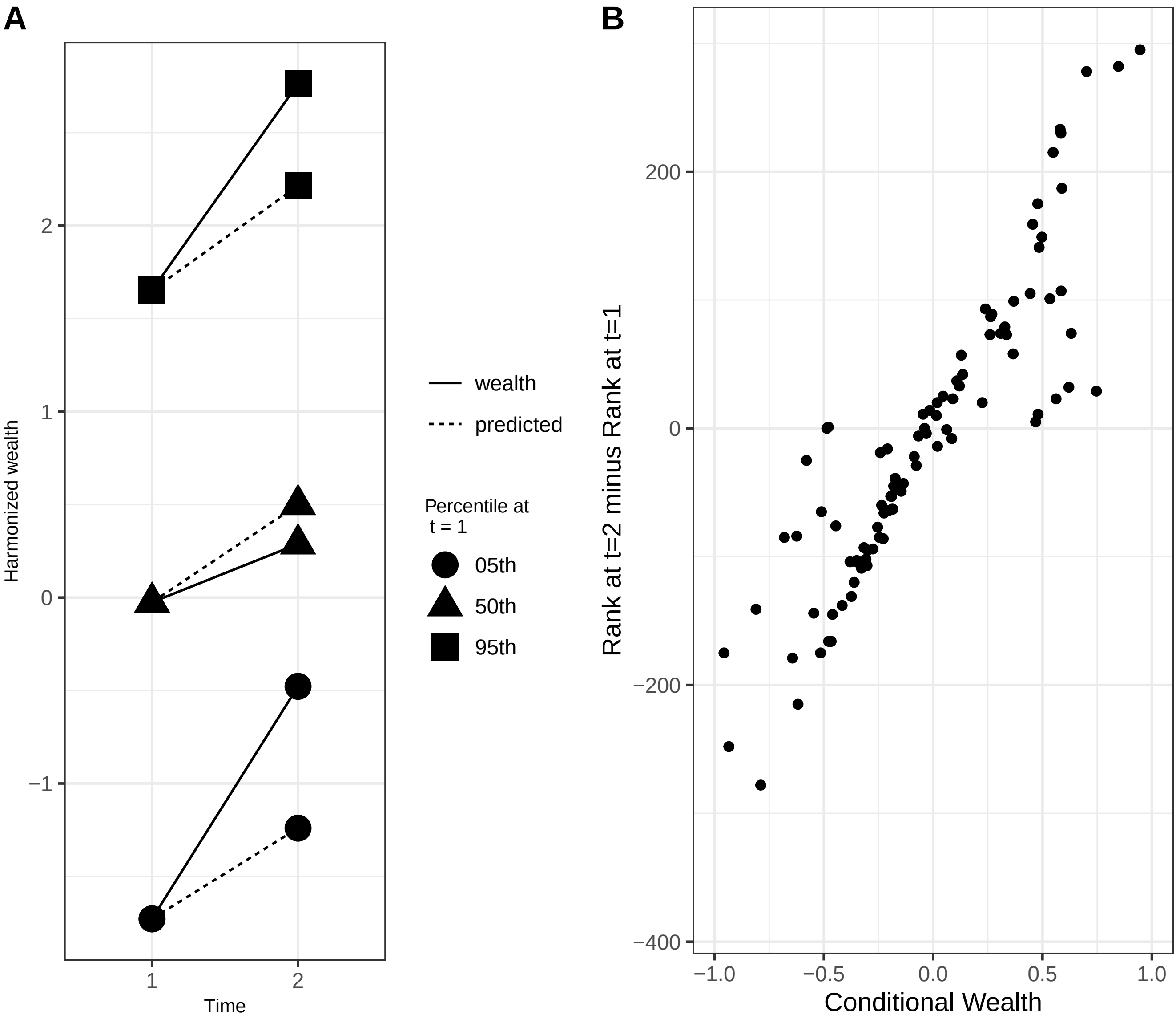

**S2Fig 70:**
**Example with w_1 = N(0,1)**
**w_2 = 1 + 1*w_1 + N(0,0.5)**
**Ratio of variance at time 2: time 1 = 1.3**

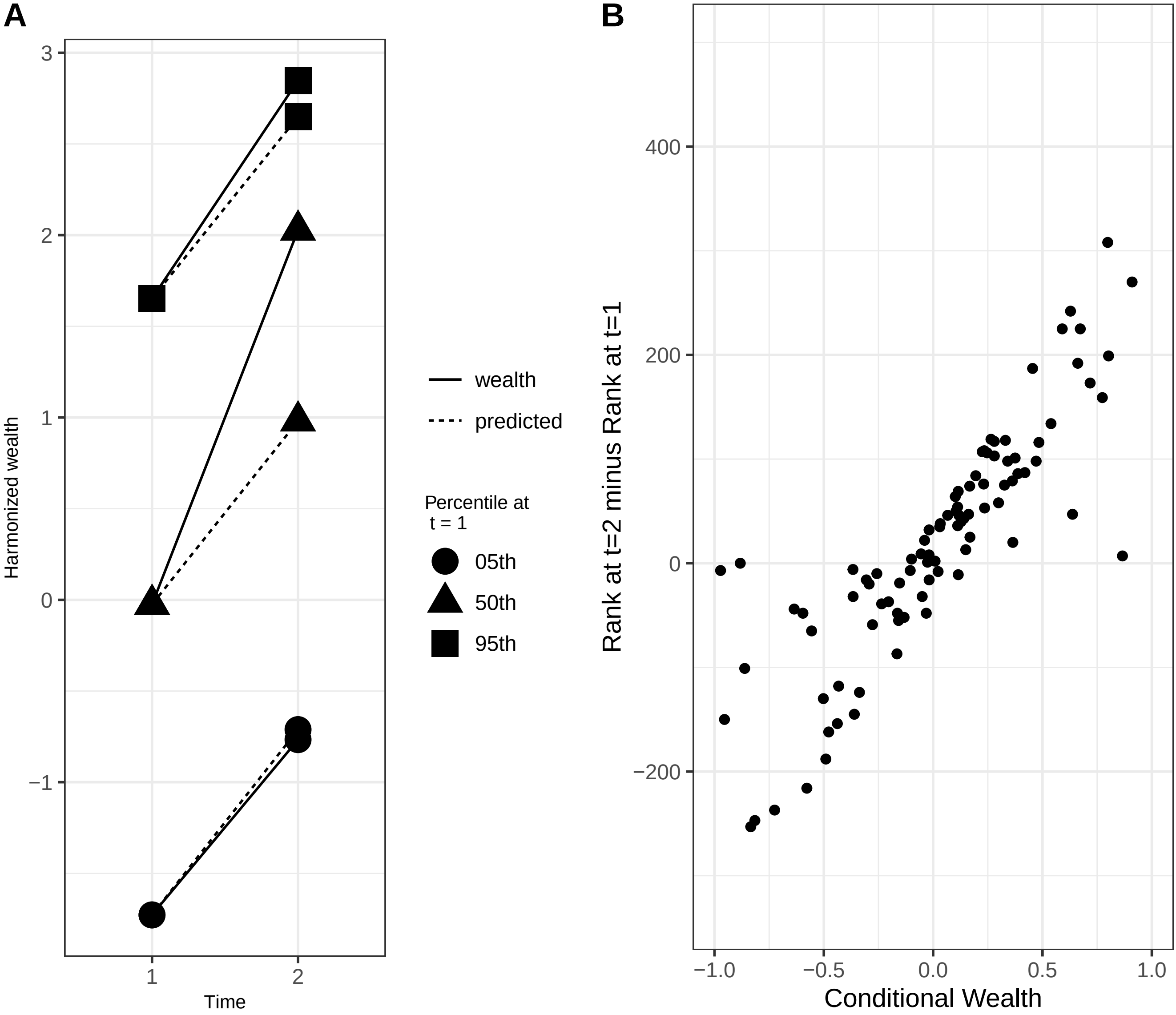

**S2Fig 71:**
**Example with w_1 = N(0,1)**
**w_2 = −1 + −1*w_1 + N(0,1)**
**Ratio of variance at time 2: time 1 = 2.1**

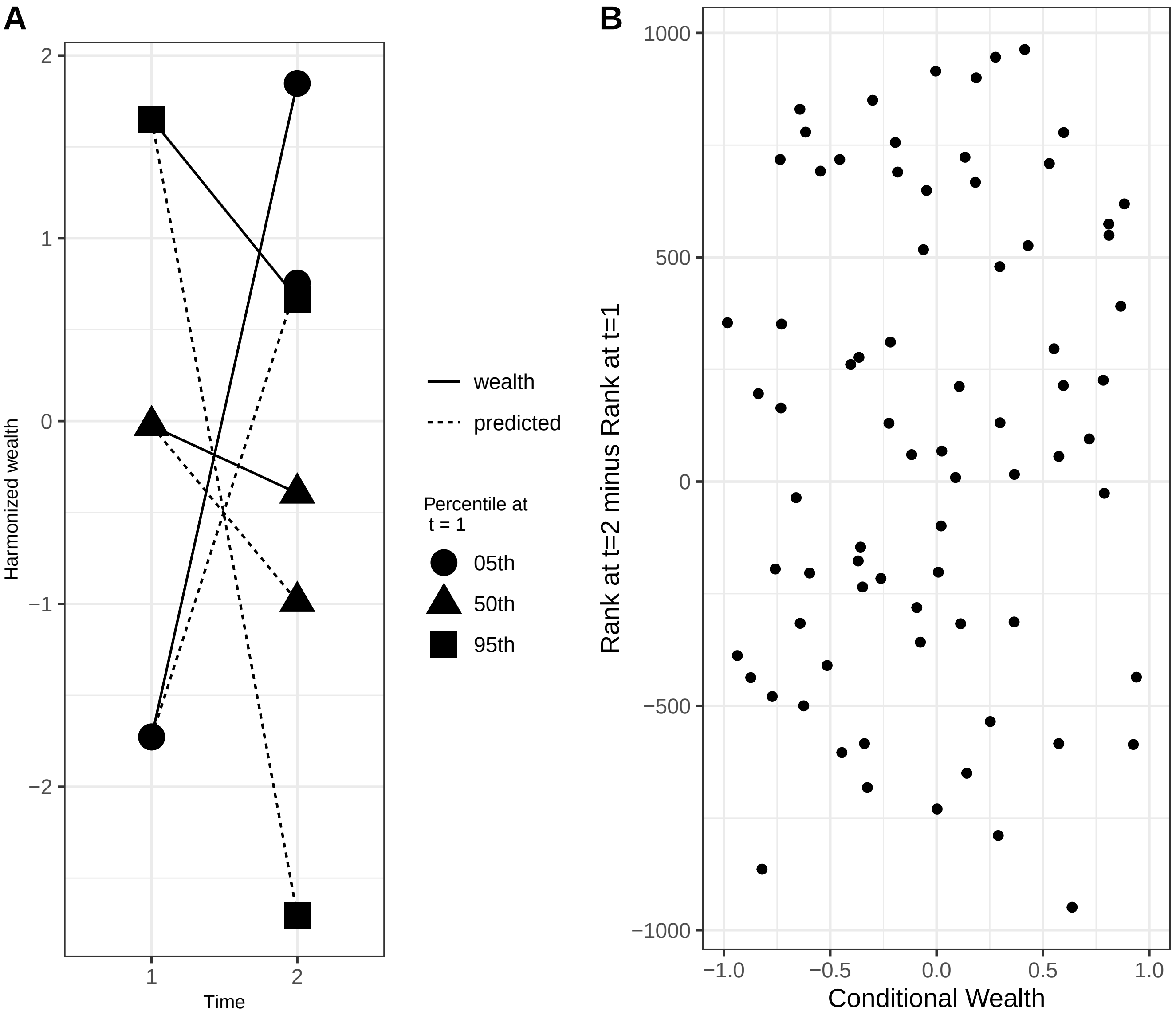

**S2Fig 72:**
**Example with w_1 = N(0,1)**
**w_2 = −0.5 + −1*w_1 + N(0,1)**
**Ratio of variance at time 2: time 1 = 2.3**

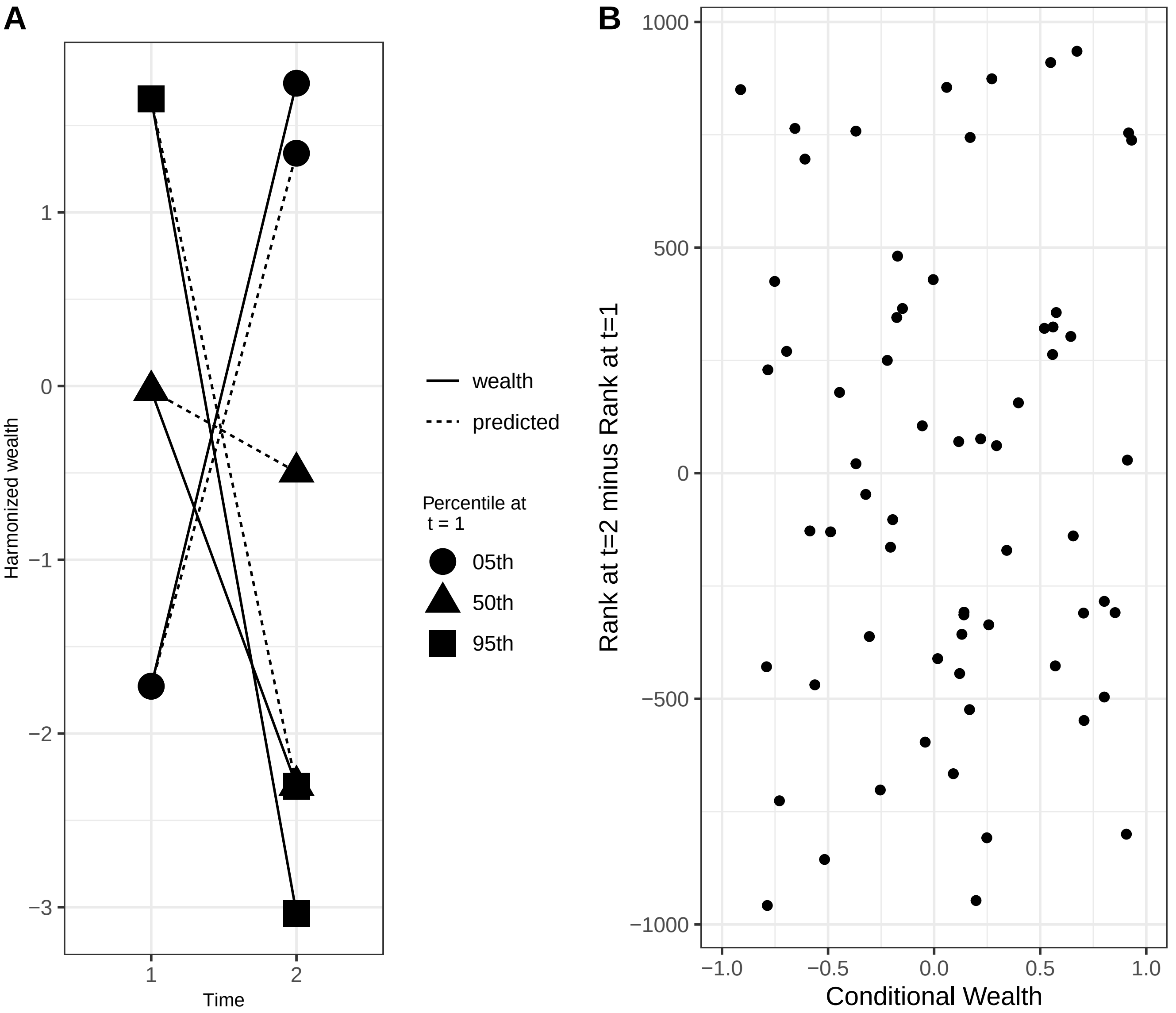

## S2Fig 73:
## Example with w_1 = N(0,1)
## w_2 = −0.3 + −1*w_1 + N(0,1)
## Ratio of variance at time 2: time 1 = 2

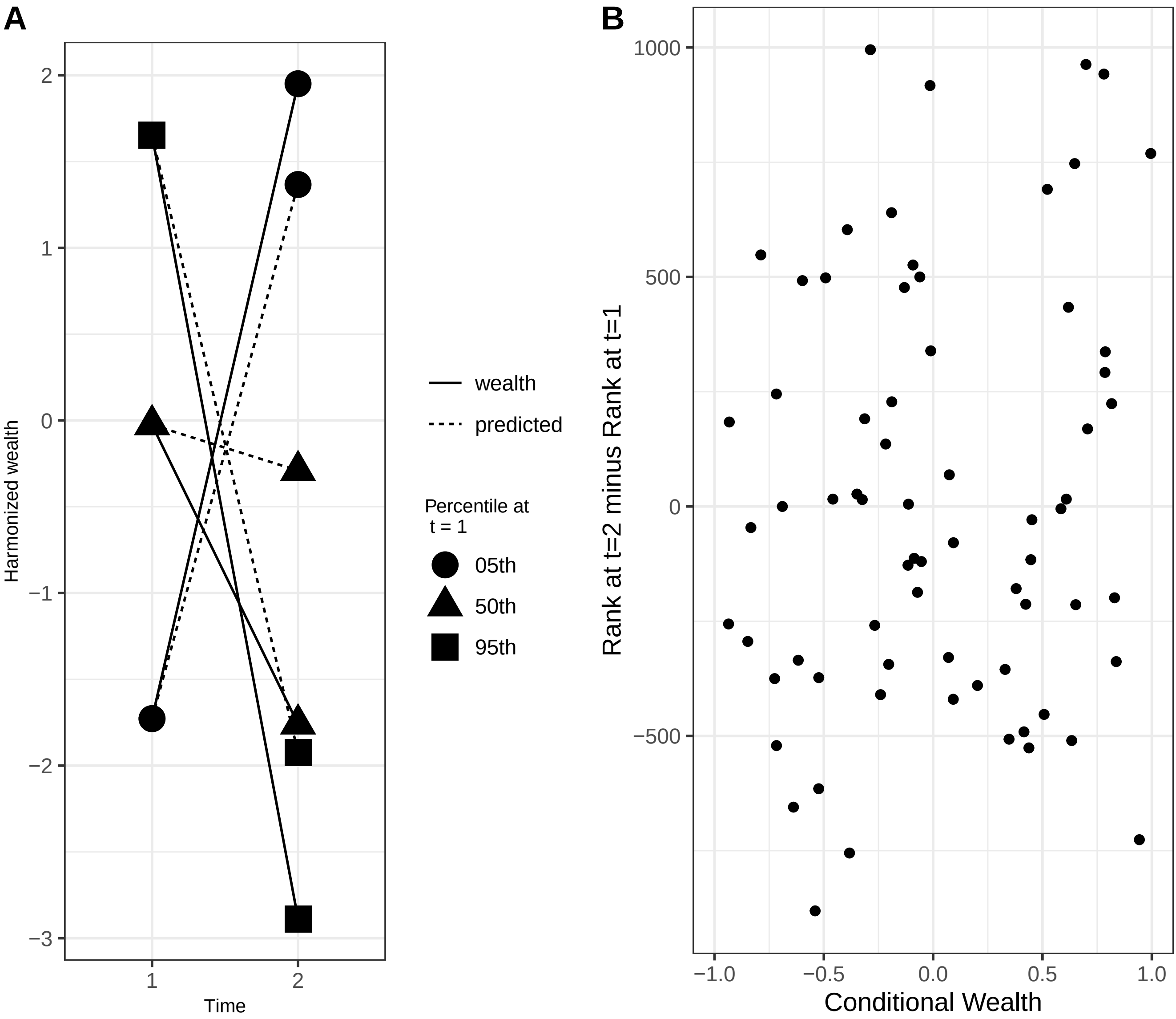

**S2Fig 74:**
**Example with w_1 = N(0,1)**
**w_2 = 0 + −1*w_1 + N(0,1)**
**Ratio of variance at time 2: time 1 = 2**

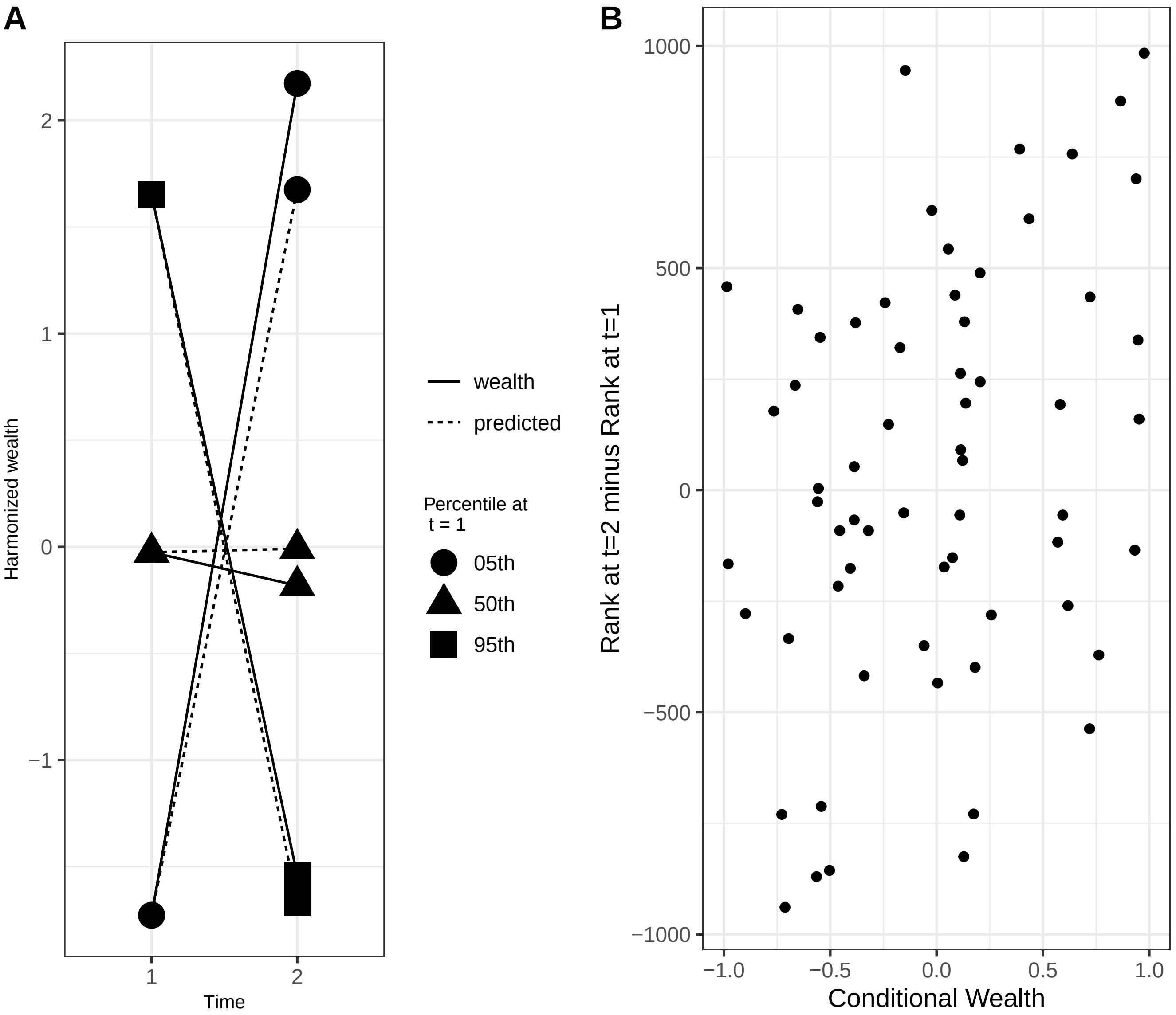

**S2Fig 75:**
**Example with w_1 = N(0,1)**
**w_2 = 0.3 + −1*w_1 + N(0,1)**
**Ratio of variance at time 2: time 1 = 1.9**

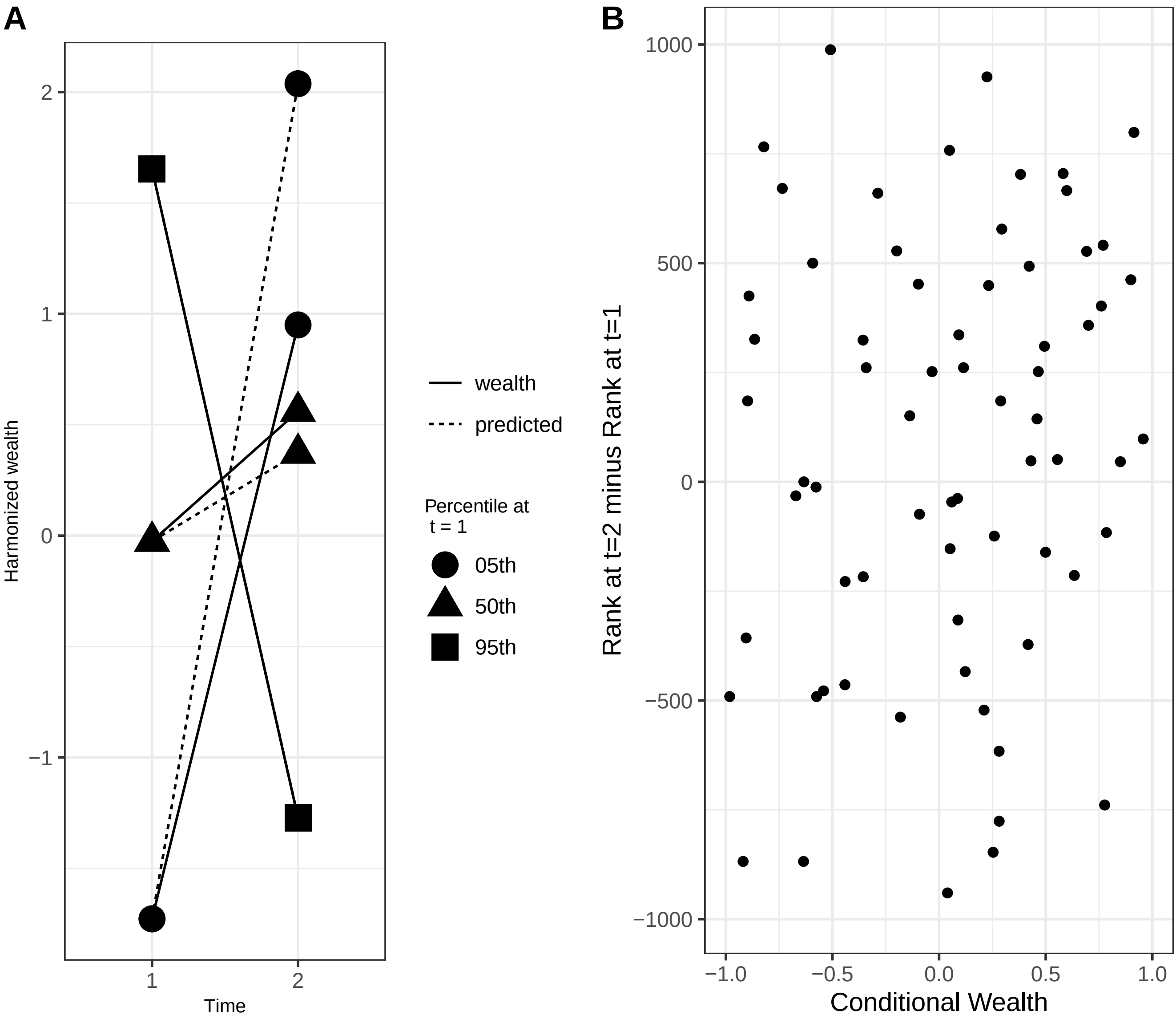

**S2Fig 76:**
**Example with w_1 = N(0,1)**
**w_2 = 0.5 + −1*w_1 + N(0,1)**
**Ratio of variance at time 2: time 1 = 1.9**

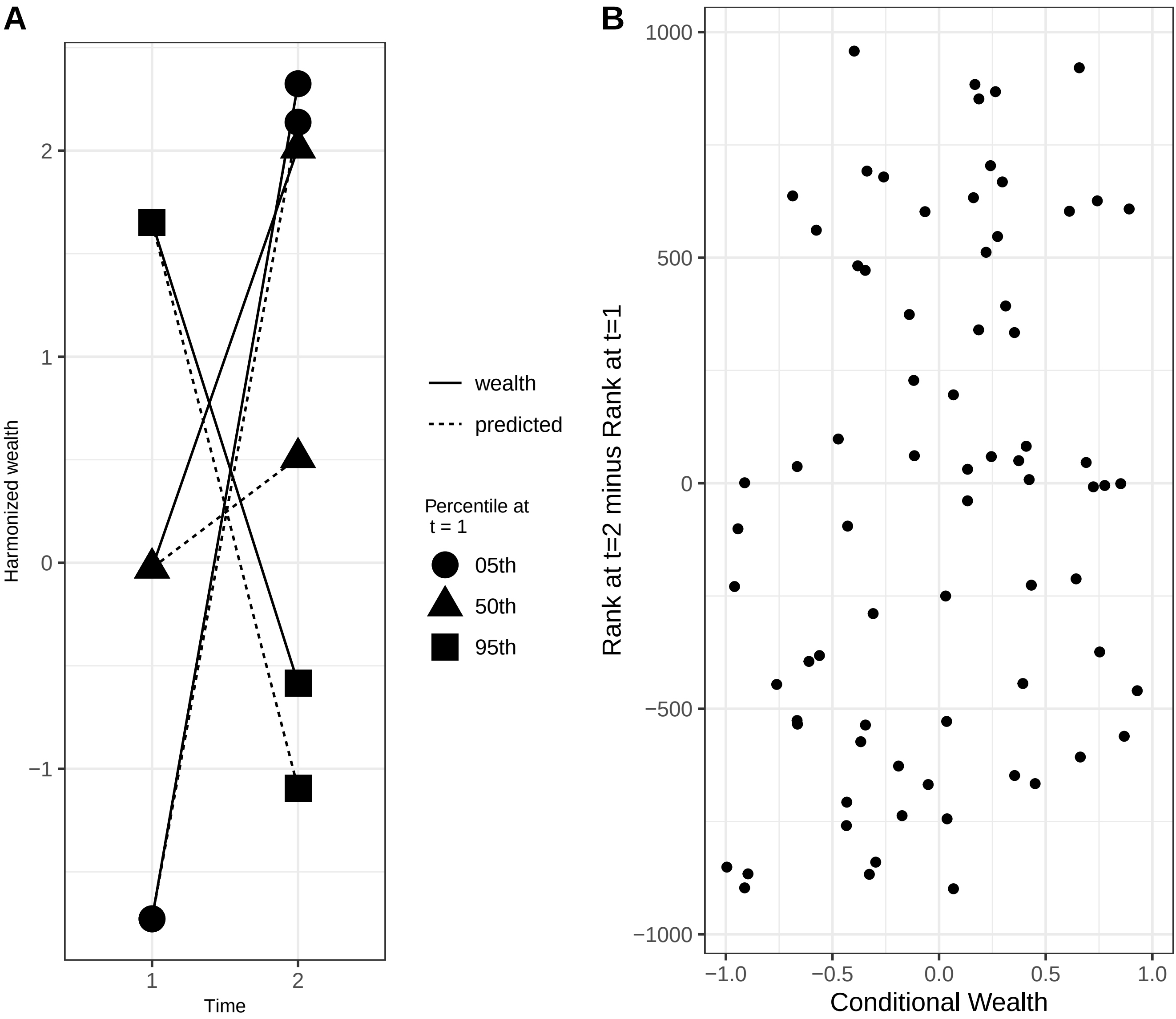

**S2Fig 77:**
**Example with w_1 = N(0,1)**
**w_2 = 1 + −1*w_1 + N(0,1)**
**Ratio of variance at time 2: time 1 = 2.1**

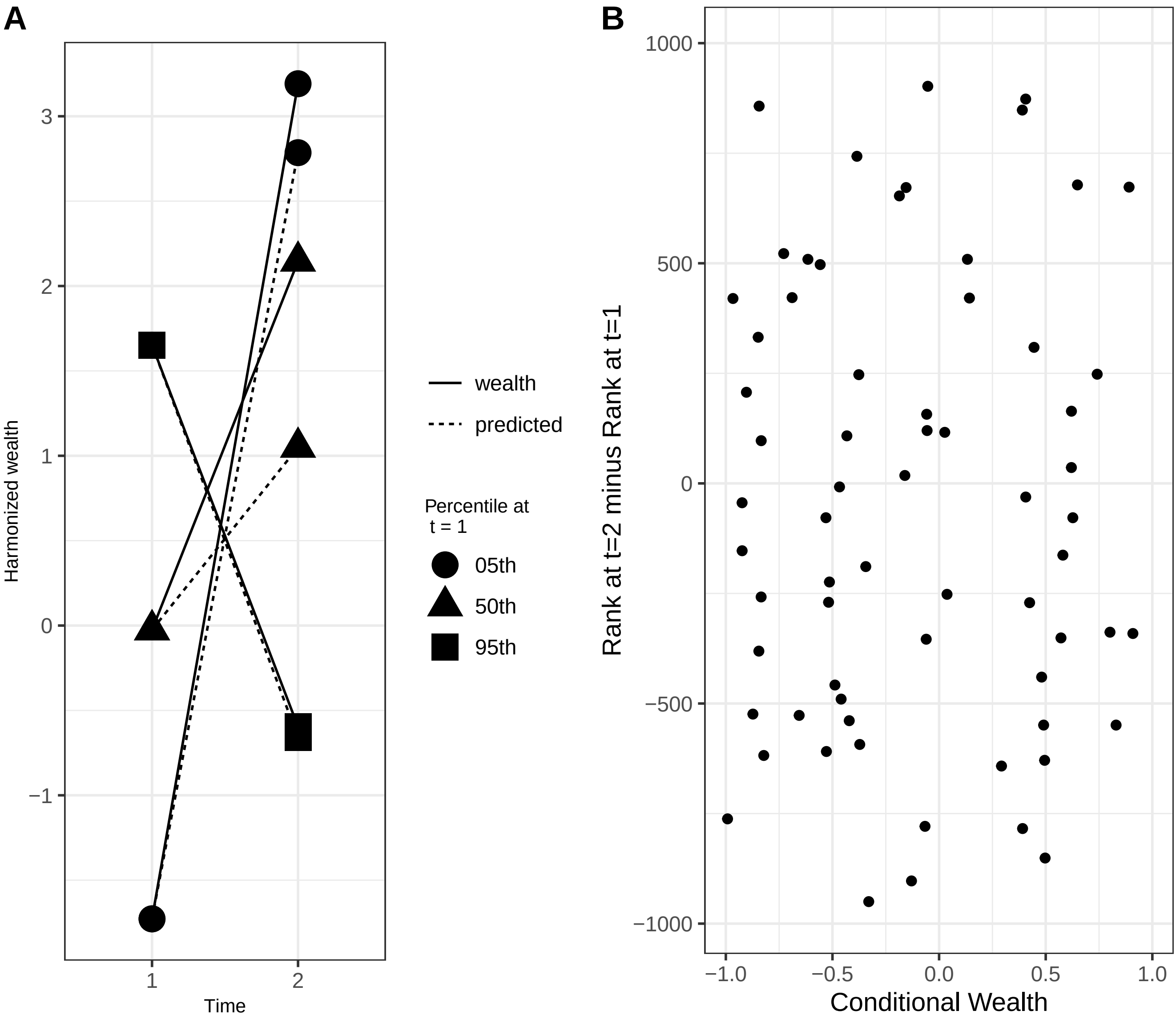

**S2Fig 78:**
**Example with w_1 = N(0,1)**
**w_2 = −1 + −0.5*w_1 + N(0,1)**
**Ratio of variance at time 2: time 1 = 1.2**

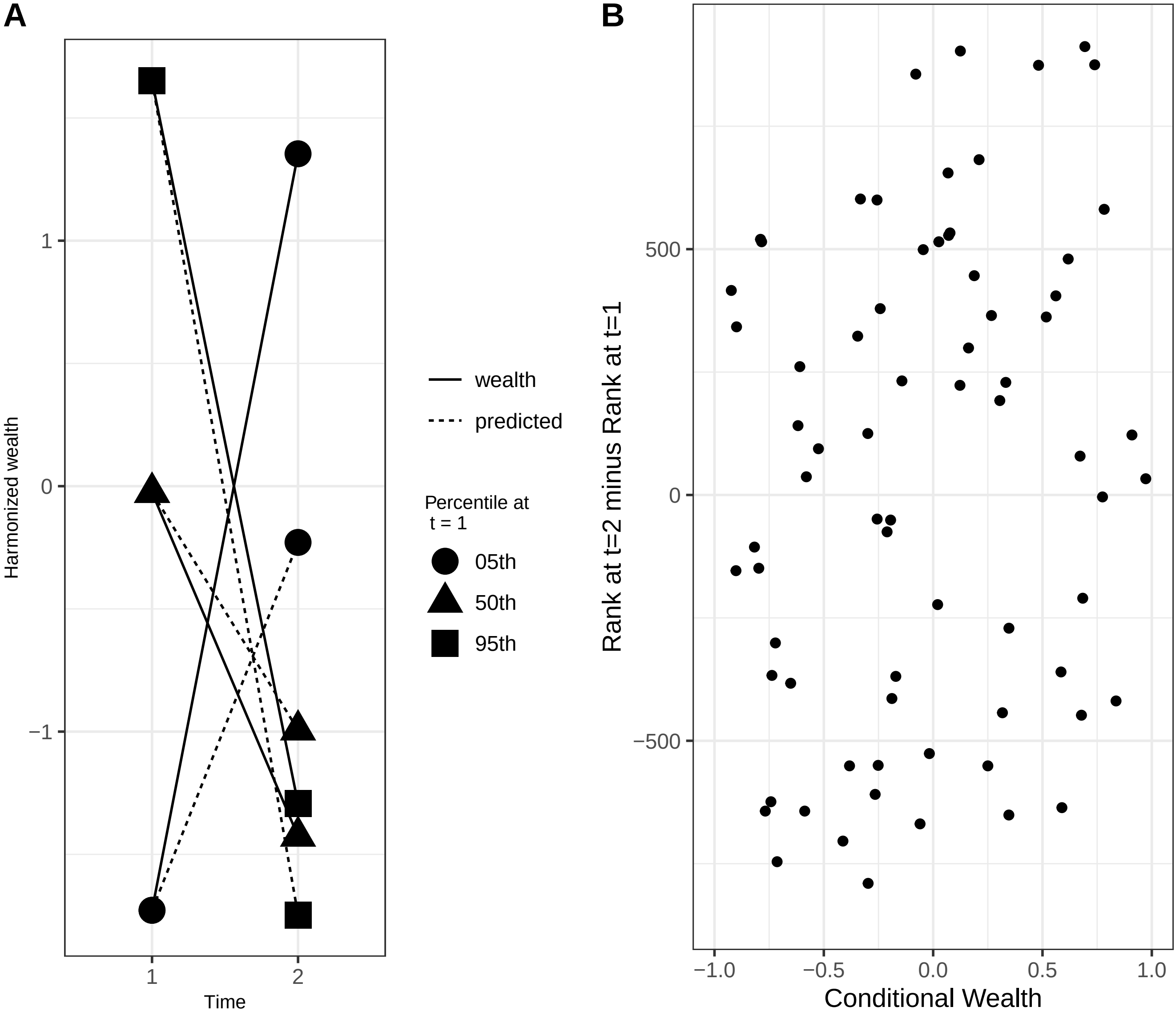

S2Fig 79:
Example with w_1 = N(0,1)
w_2 = −0.5 + −0.5*w_1 + N(0,1)
Ratio of variance at time 2: time 1 = 1.2

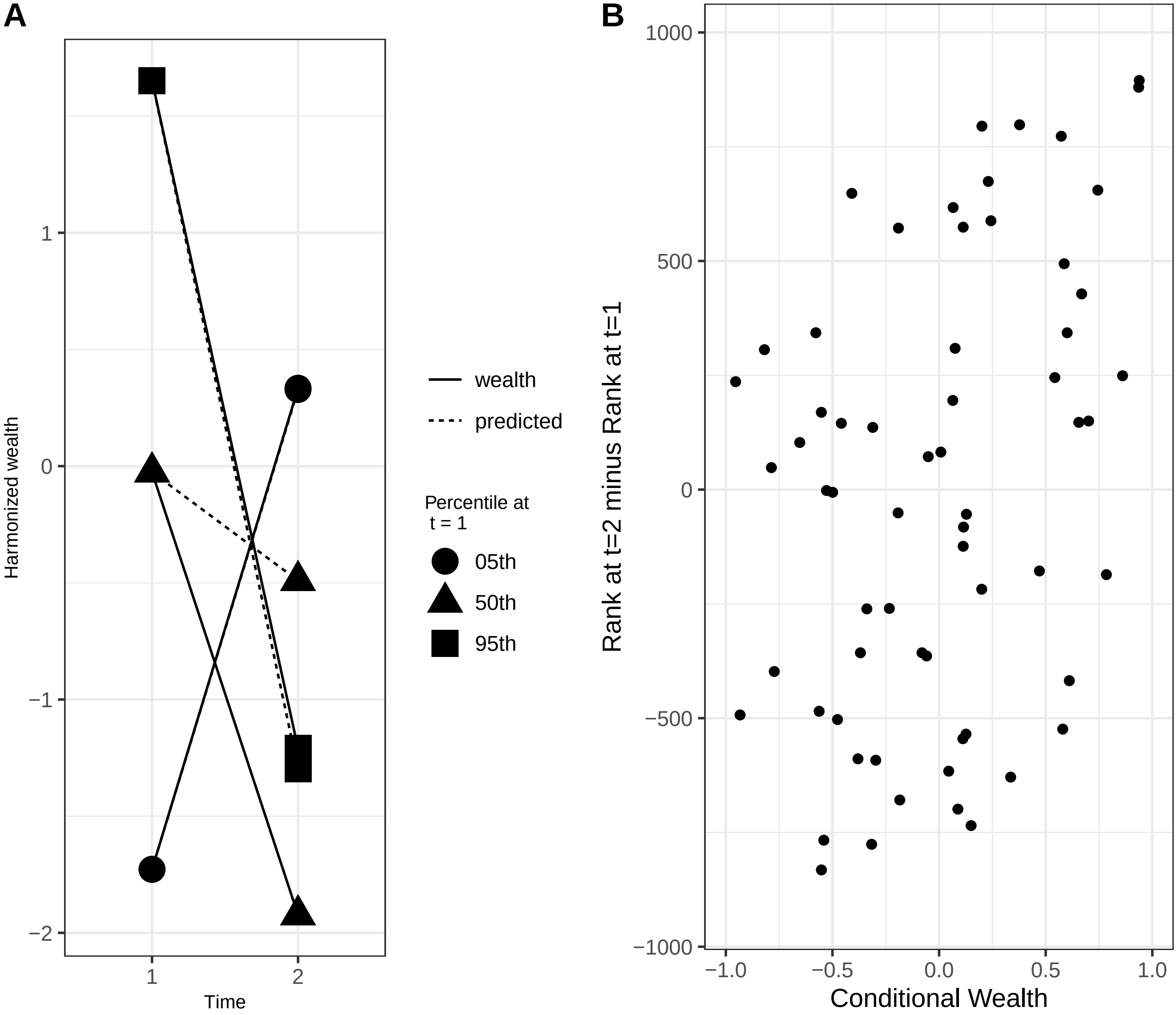

**S2Fig 80:**
**Example with w_1 = N(0,1)**
**w_2 = −0.3 + −0.5*w_1 + N(0,1)**
**Ratio of variance at time 2: time 1 = 1.3**

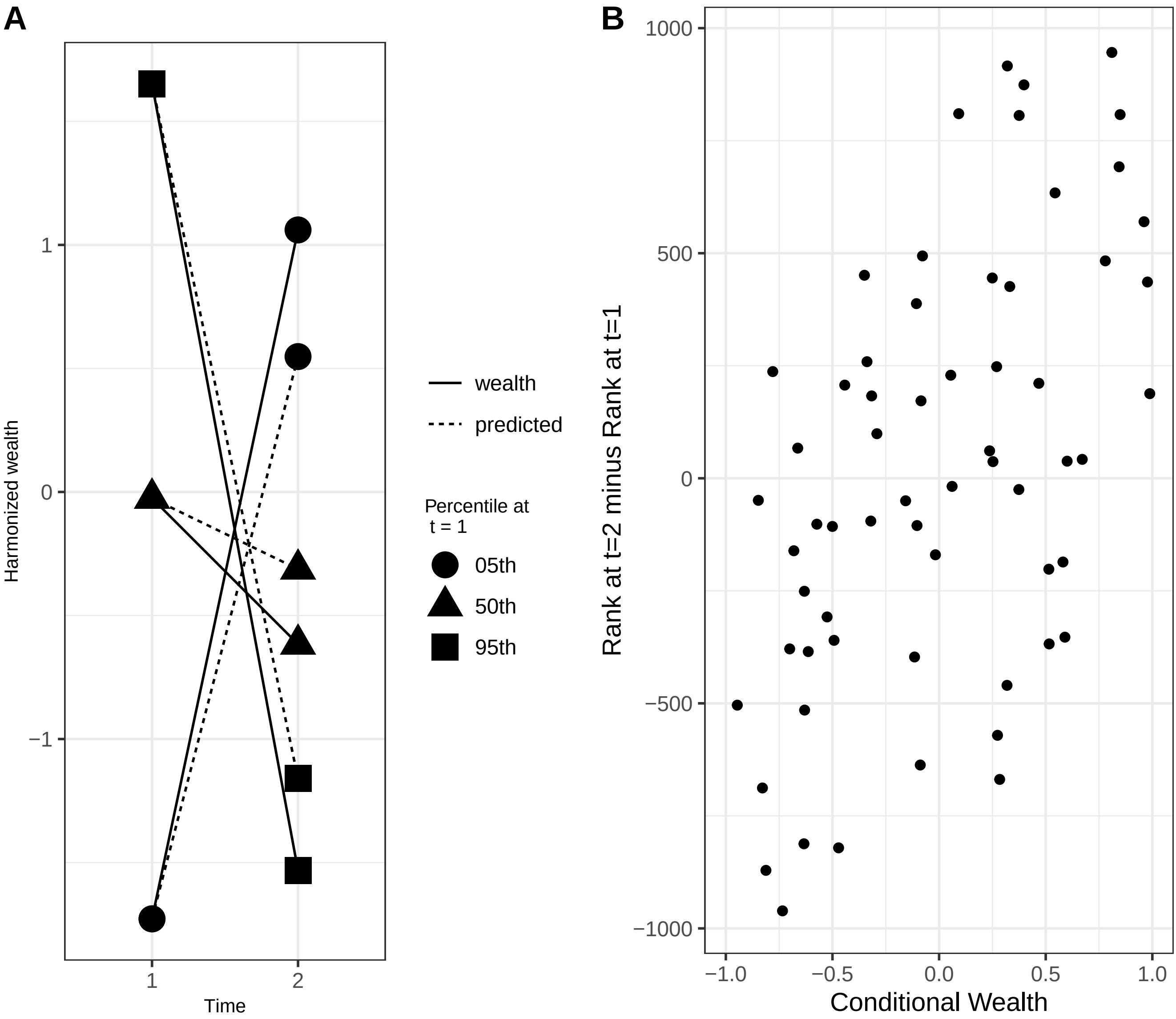

**S2Fig 81:**
**Example with w_1 = N(0,1)**
**w_2 = 0 + −0.5*w_1 + N(0,1)**
**Ratio of variance at time 2: time 1 = 1.3**

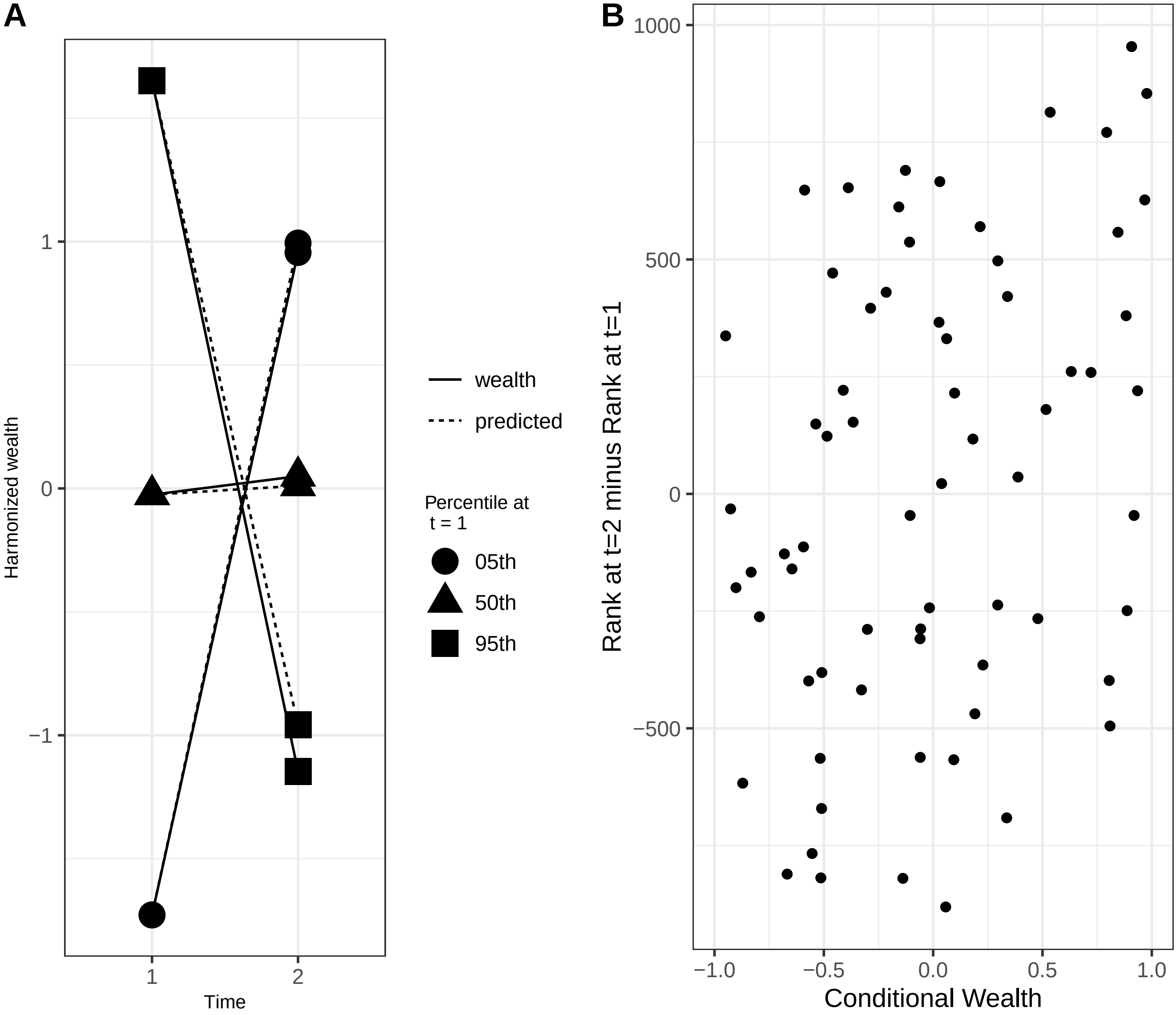

**S2Fig 82:**
**Example with w_1 = N(0,1)**
**w_2 = 0.3 + −0.5*w_1 + N(0,1)**
**Ratio of variance at time 2: time 1 = 1.2**

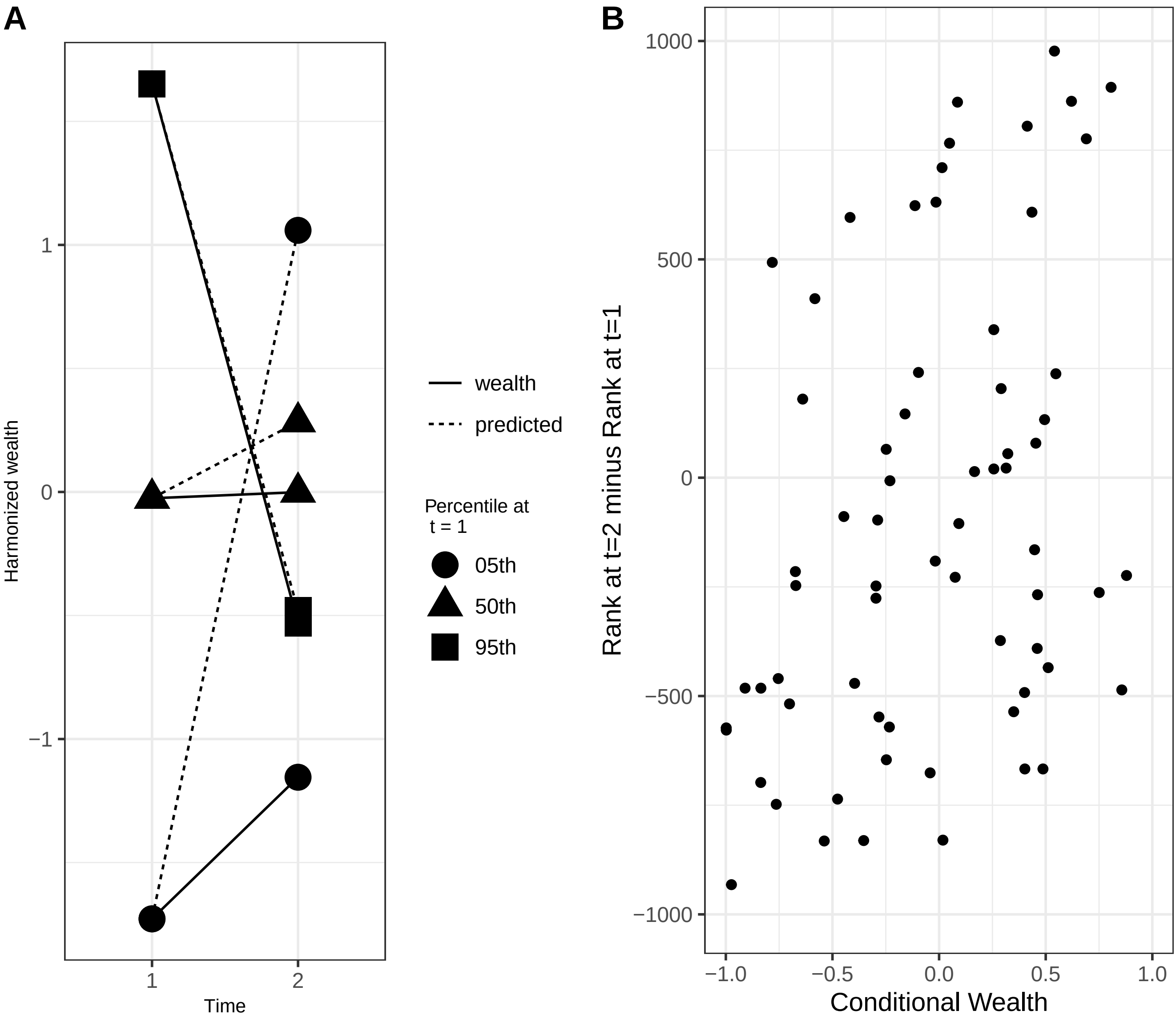

**S2Fig 83:**
**Example with w_1 = N(0,1)**
**w_2 = 0.5 + −0.5*w_1 + N(0,1)**
**Ratio of variance at time 2: time 1 = 1.2**

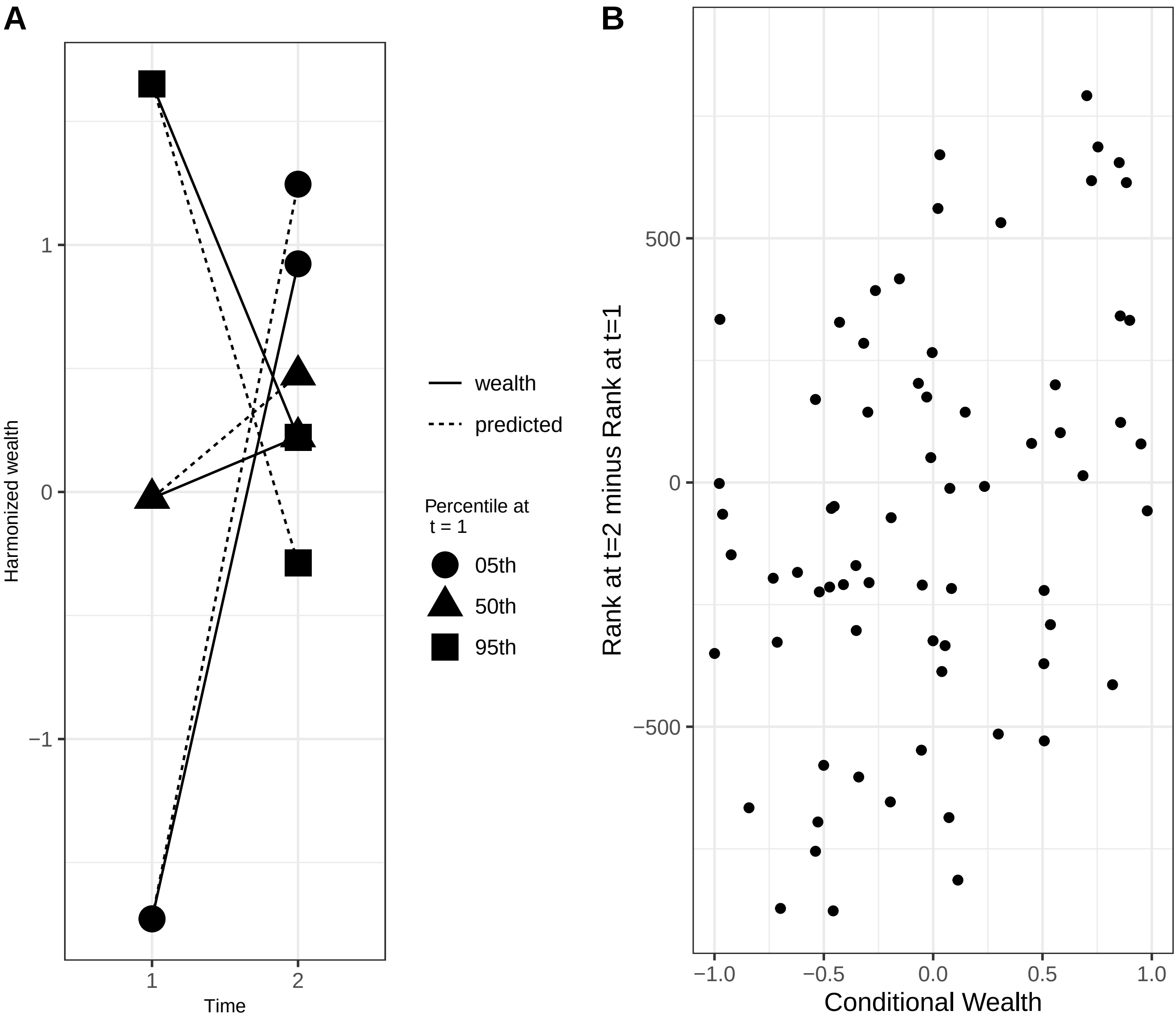

**S2Fig 84:**
**Example with w_1 = N(0,1)**
**w_2 = 1 + −0.5*w_1 + N(0,1)**
**Ratio of variance at time 2: time 1 = 1.3**

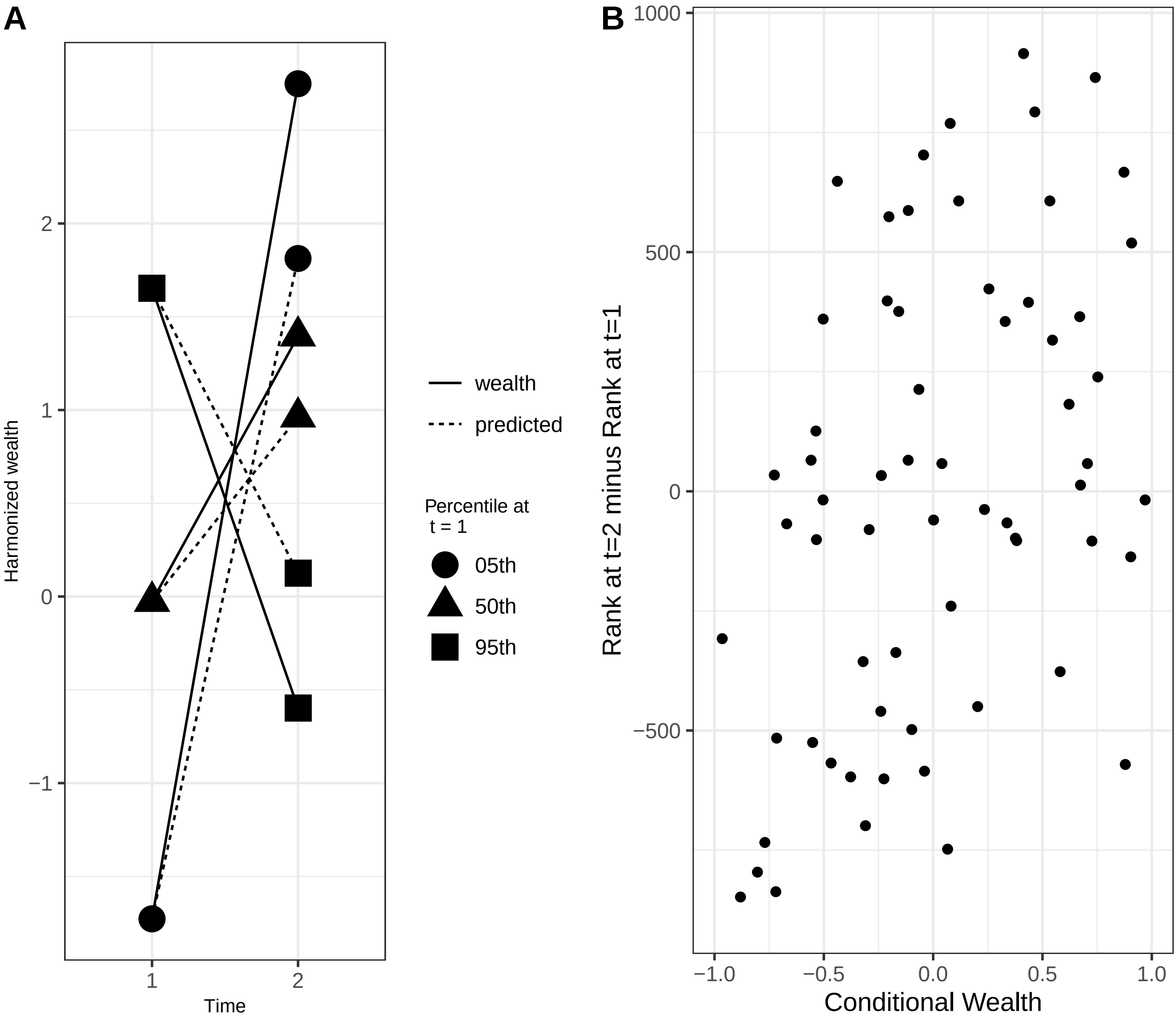

**S2Fig 85:**
**Example with w_1 = N(0,1)**
**w_2 = −1 + 0*w_1 + N(0,1)**
**Ratio of variance at time 2: time 1 = 1.1**

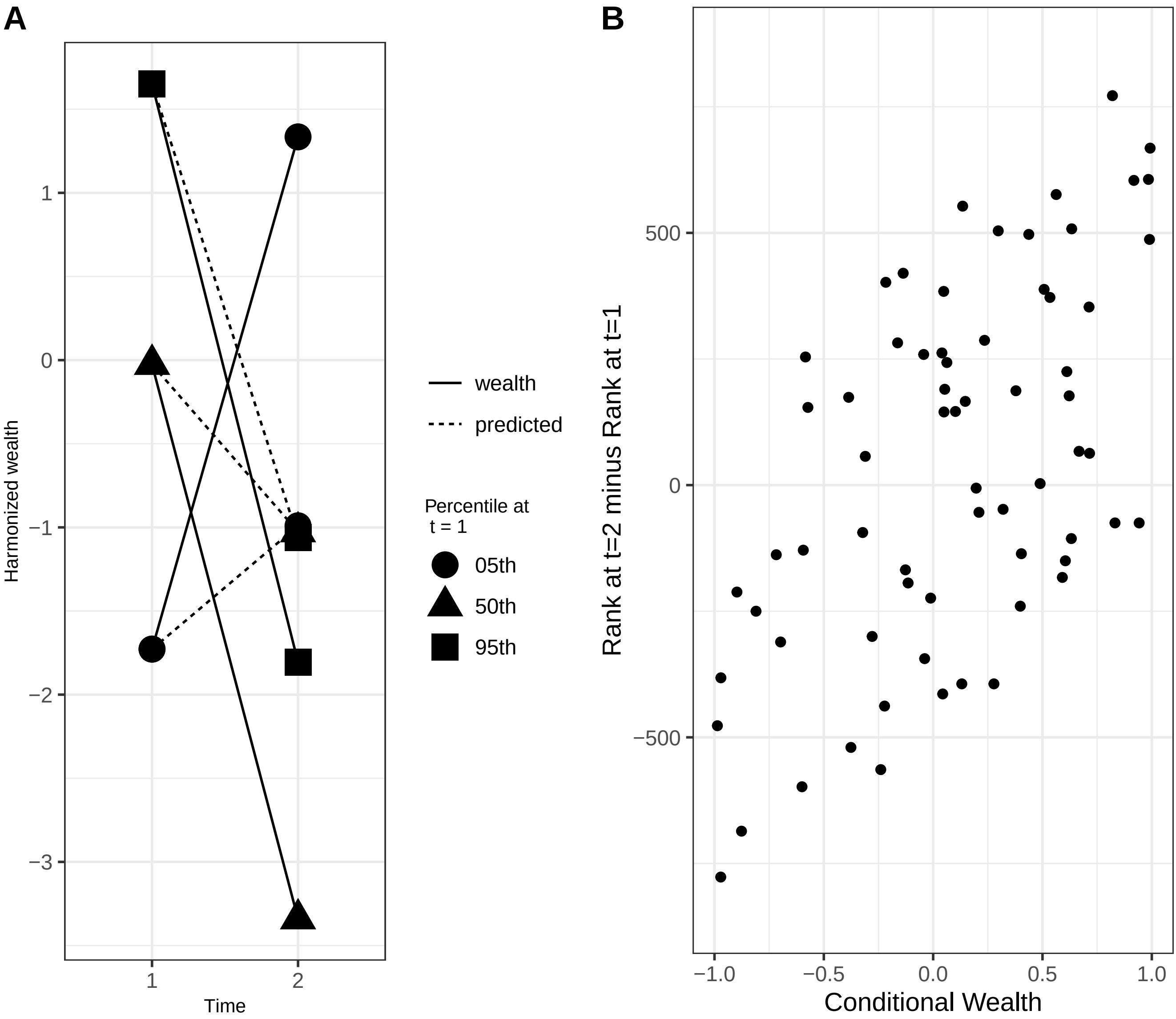

**S2Fig 86:**
**Example with w_1 = N(0,1)**
**w_2 = −0.5 + 0*w_1 + N(0,1)**
**Ratio of variance at time 2: time 1 = 1**

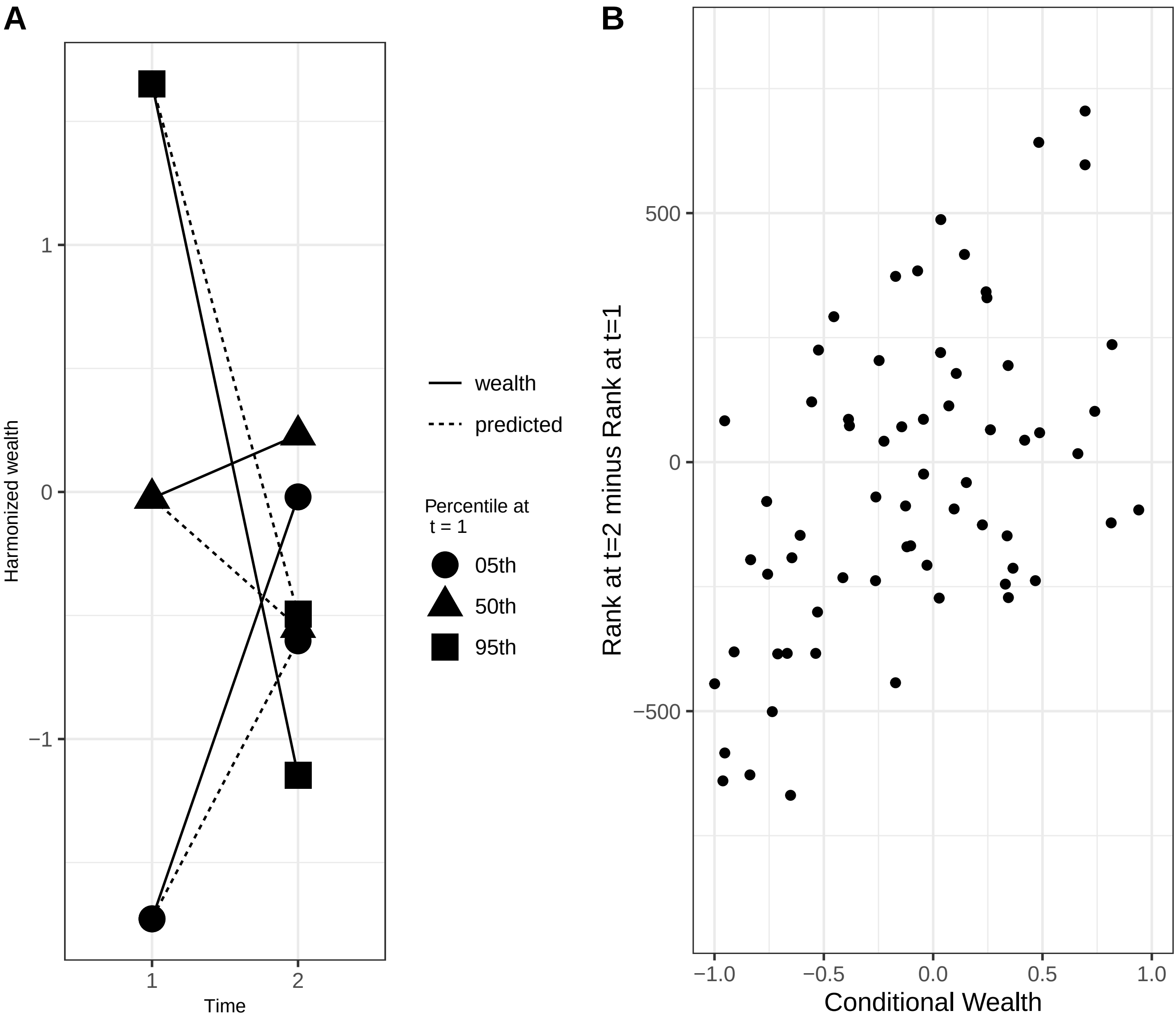

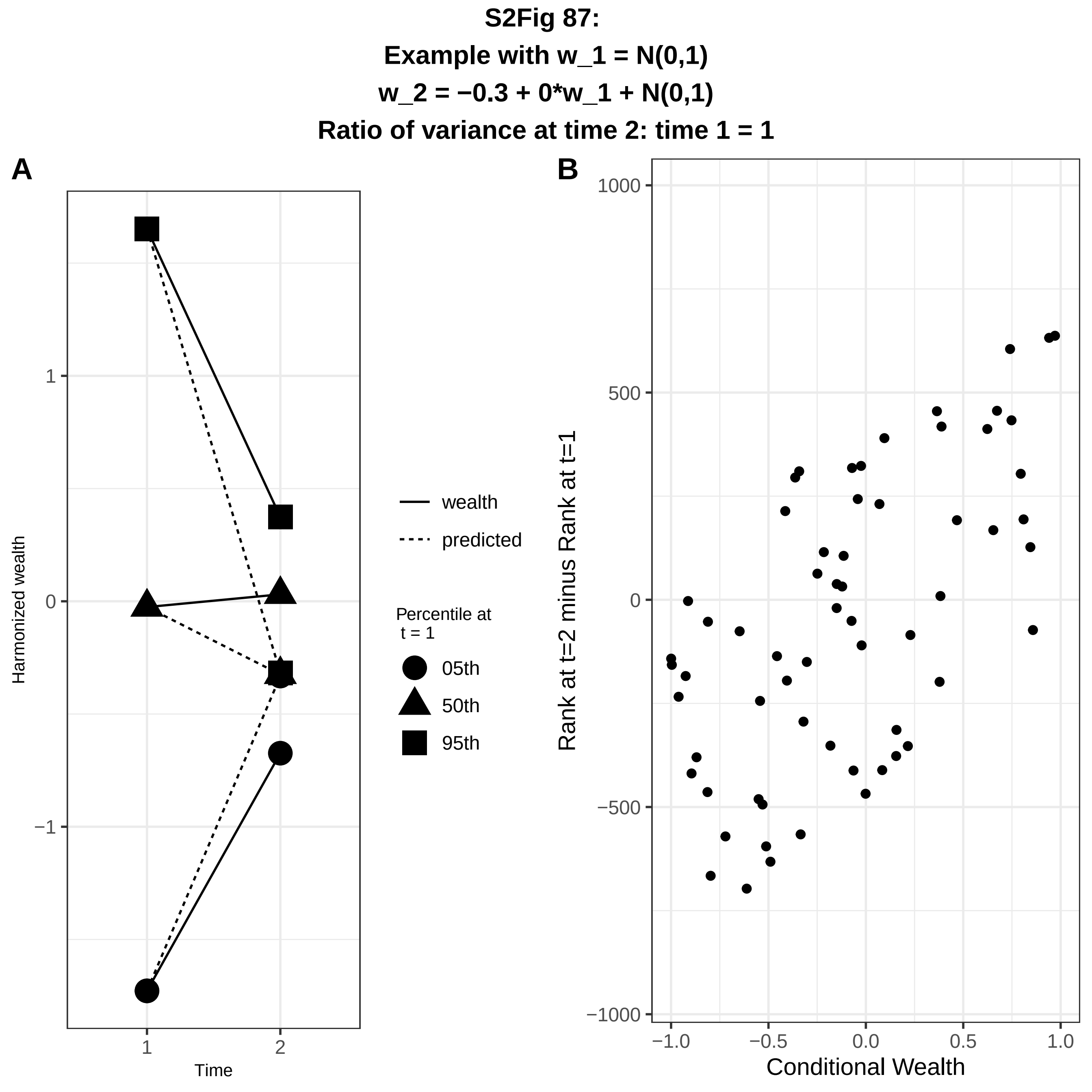
S2Fig 87:
Example with w_1 = N(0,1)
w_2 = −0.3 + 0*w_1 + N(0,1)
Ratio of variance at time 2: time 1 = 1
A
Harmonized wealth
Time
wealth
predicted
Percentile at t = 1
05th
50th
95th
B
Rank at t=2 minus Rank at t=1
Conditional Wealth

**S2Fig 88:**
**Example with w_1 = N(0,1)**
**w_2 = 0 + 0*w_1 + N(0,1)**
**Ratio of variance at time 2: time 1 = 1**

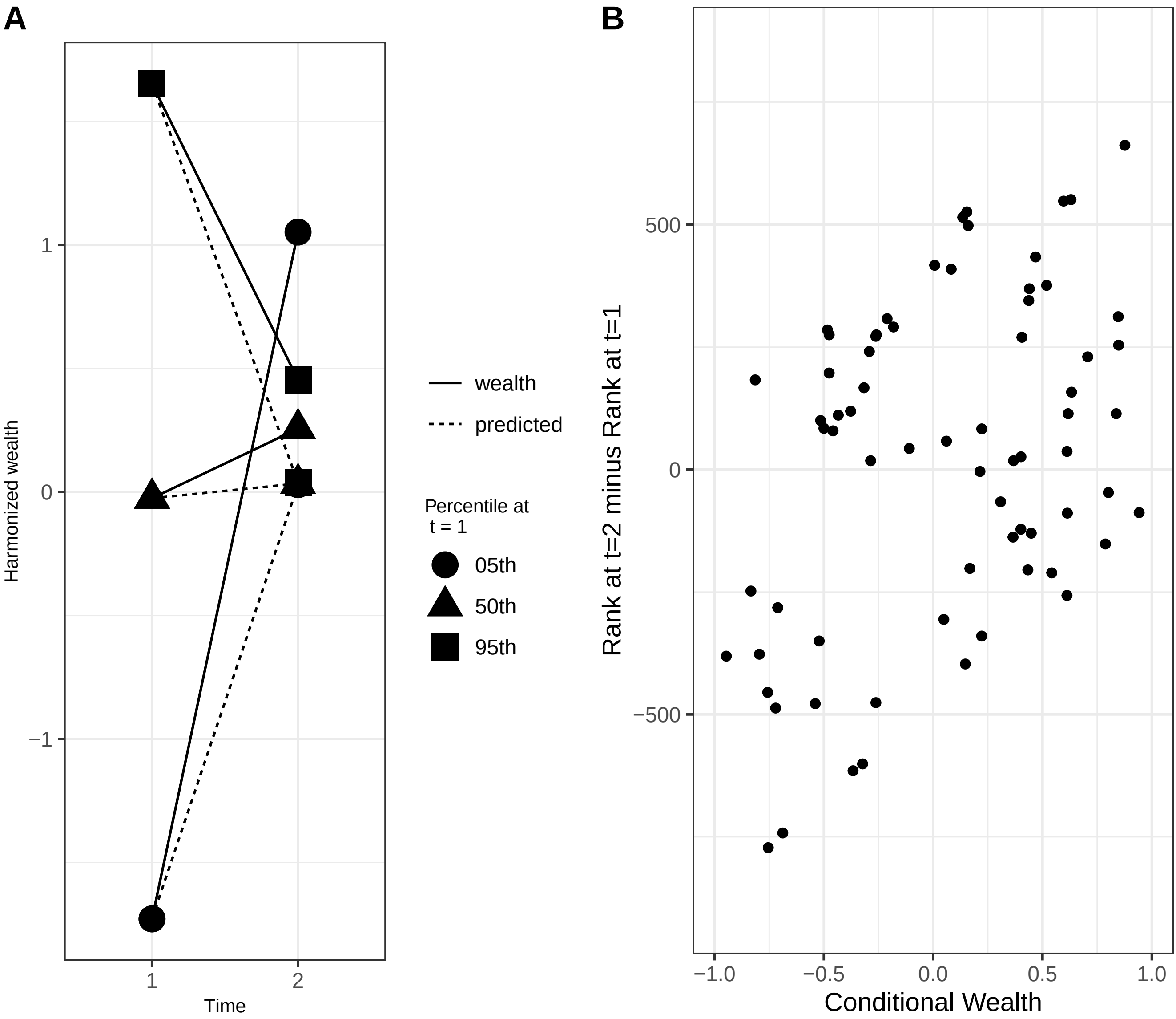

**S2Fig 89:**
**Example with w_1 = N(0,1)**
**w_2 = 0.3 + 0*w_1 + N(0,1)**
**Ratio of variance at time 2: time 1 = 0.9**

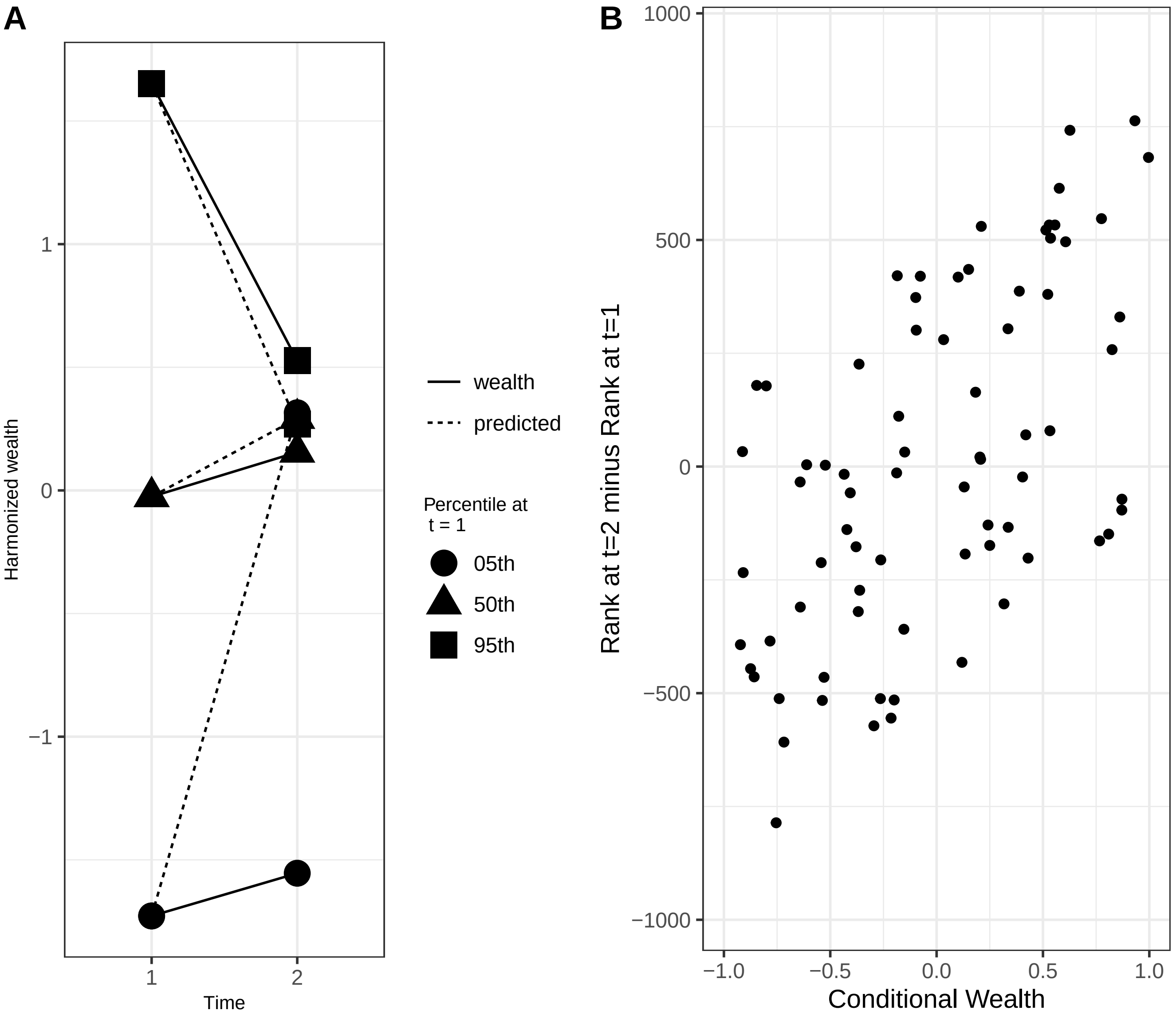

**S2Fig 90:**
**Example with w_1 = N(0,1)**
**w_2 = 0.5 + 0*w_1 + N(0,1)**
**Ratio of variance at time 2: time 1 = 0.9**

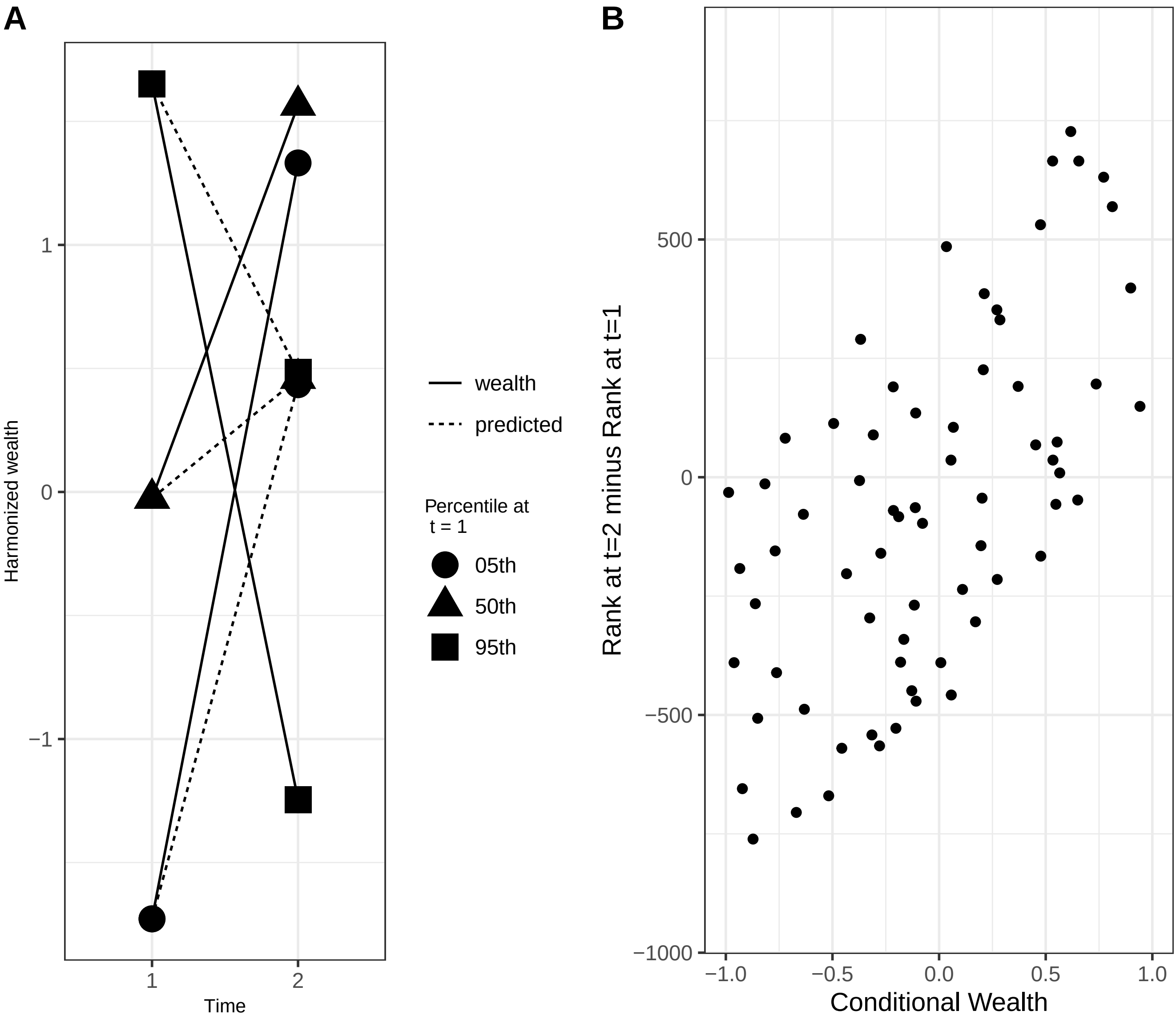

**S2Fig 91:**
**Example with w_1 = N(0,1)**
**w_2 = 1 + 0*w_1 + N(0,1)**
**Ratio of variance at time 2: time 1 = 1**

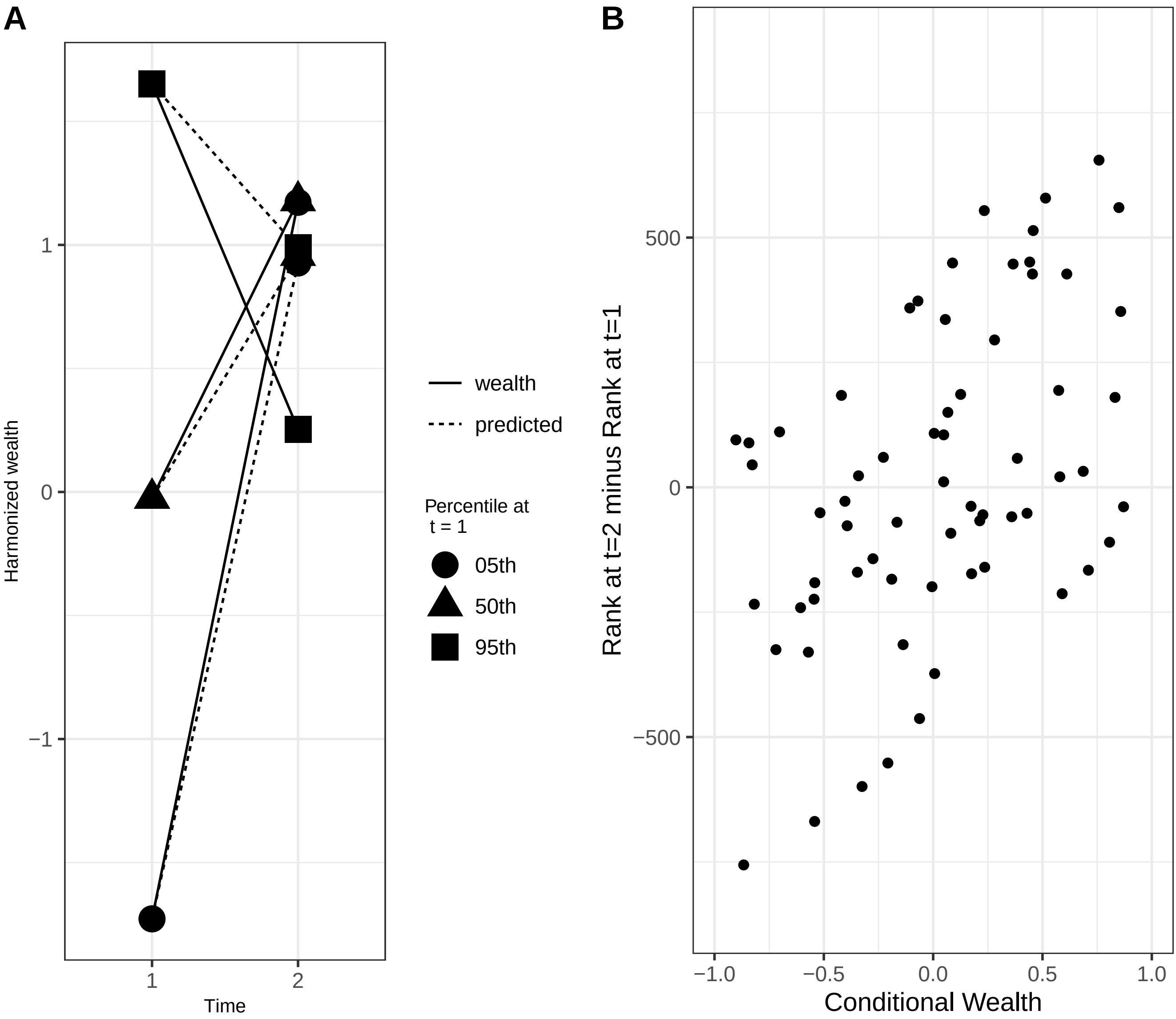

## S2Fig 92:
## Example with w_1 = N(0,1)
## w_2 = −1 + 0.5*w_1 + N(0,1)
## Ratio of variance at time 2: time 1 = 1.2

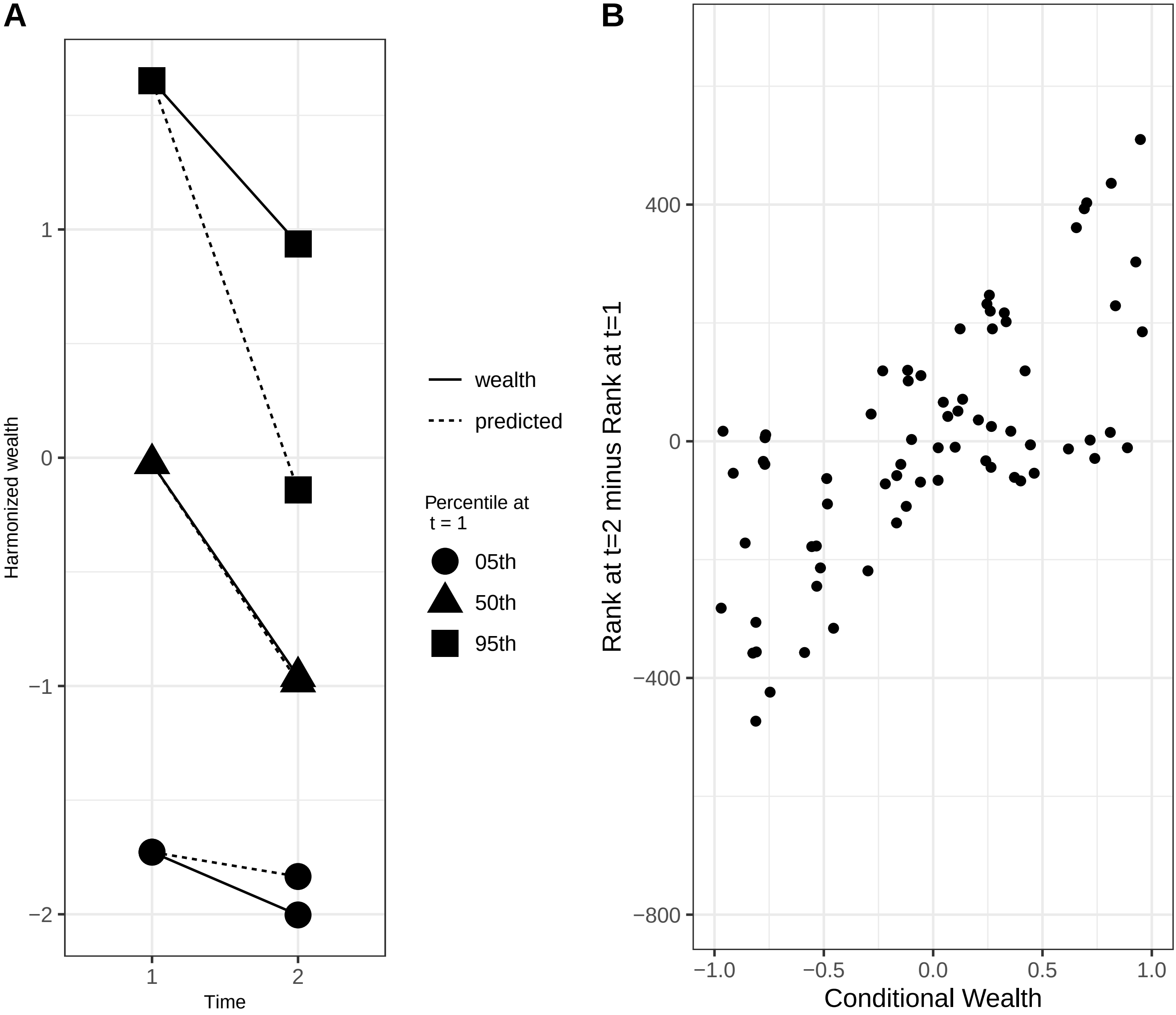

**S2Fig 93:**
**Example with w_1 = N(0,1)**
**w_2 = −0.5 + 0.5*w_1 + N(0,1)**
**Ratio of variance at time 2: time 1 = 1.2**

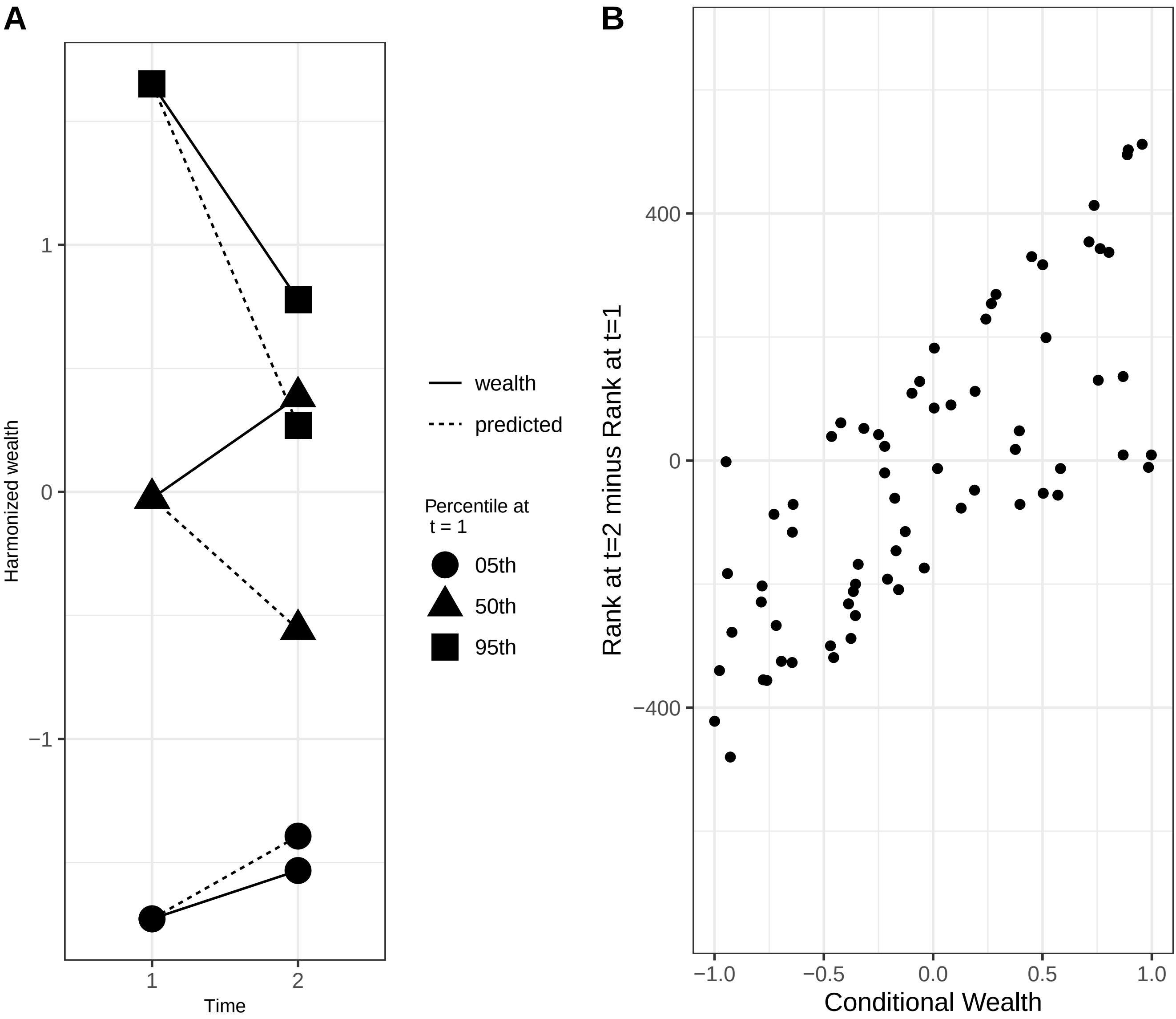

**S2Fig 94:**
**Example with w_1 = N(0,1)**
**w_2 = −0.3 + 0.5*w_1 + N(0,1)**
**Ratio of variance at time 2: time 1 = 1.3**

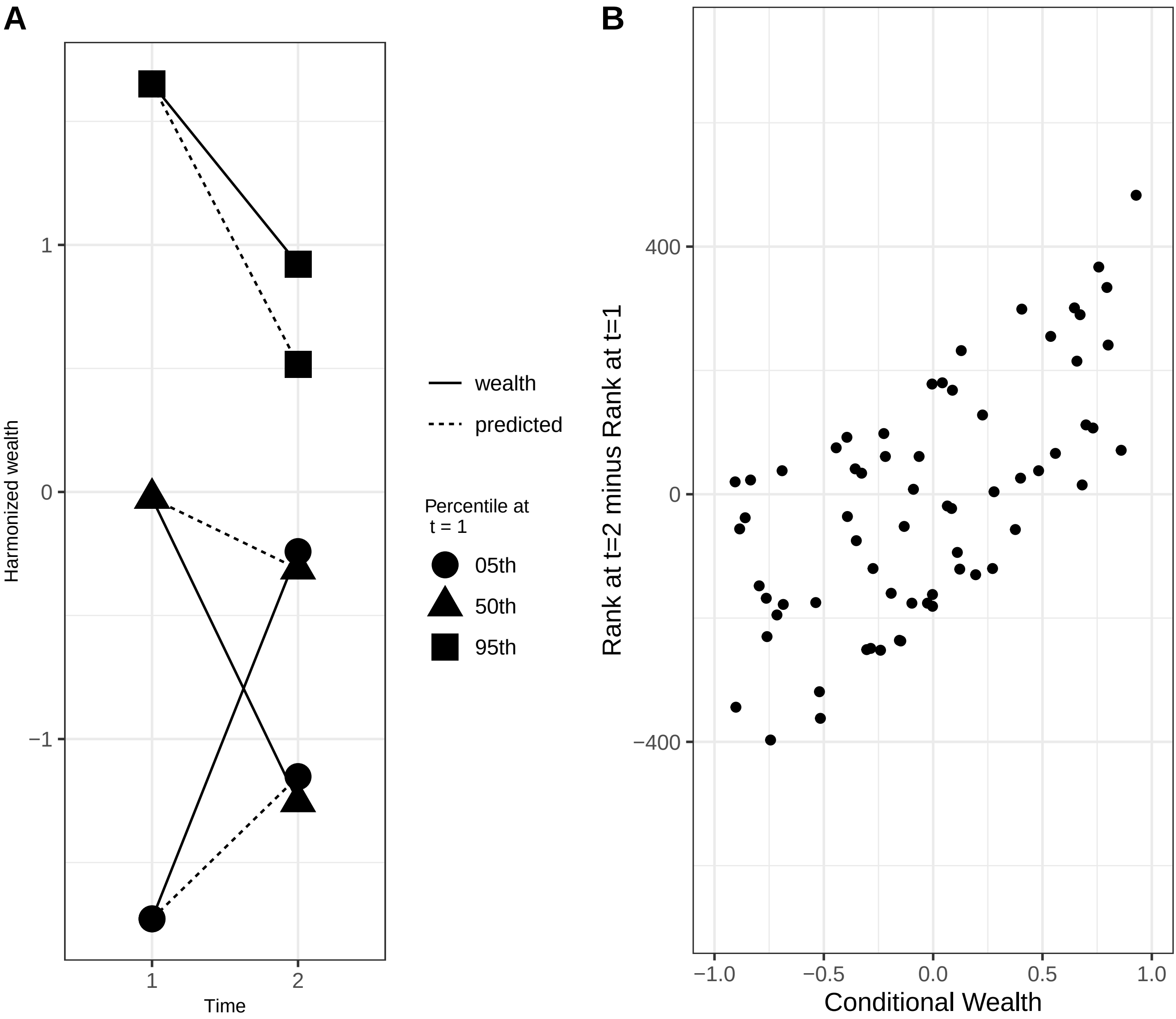

**S2Fig 95:**
**Example with w_1 = N(0,1)**
**w_2 = 0 + 0.5*w_1 + N(0,1)**
**Ratio of variance at time 2: time 1 = 1.2**

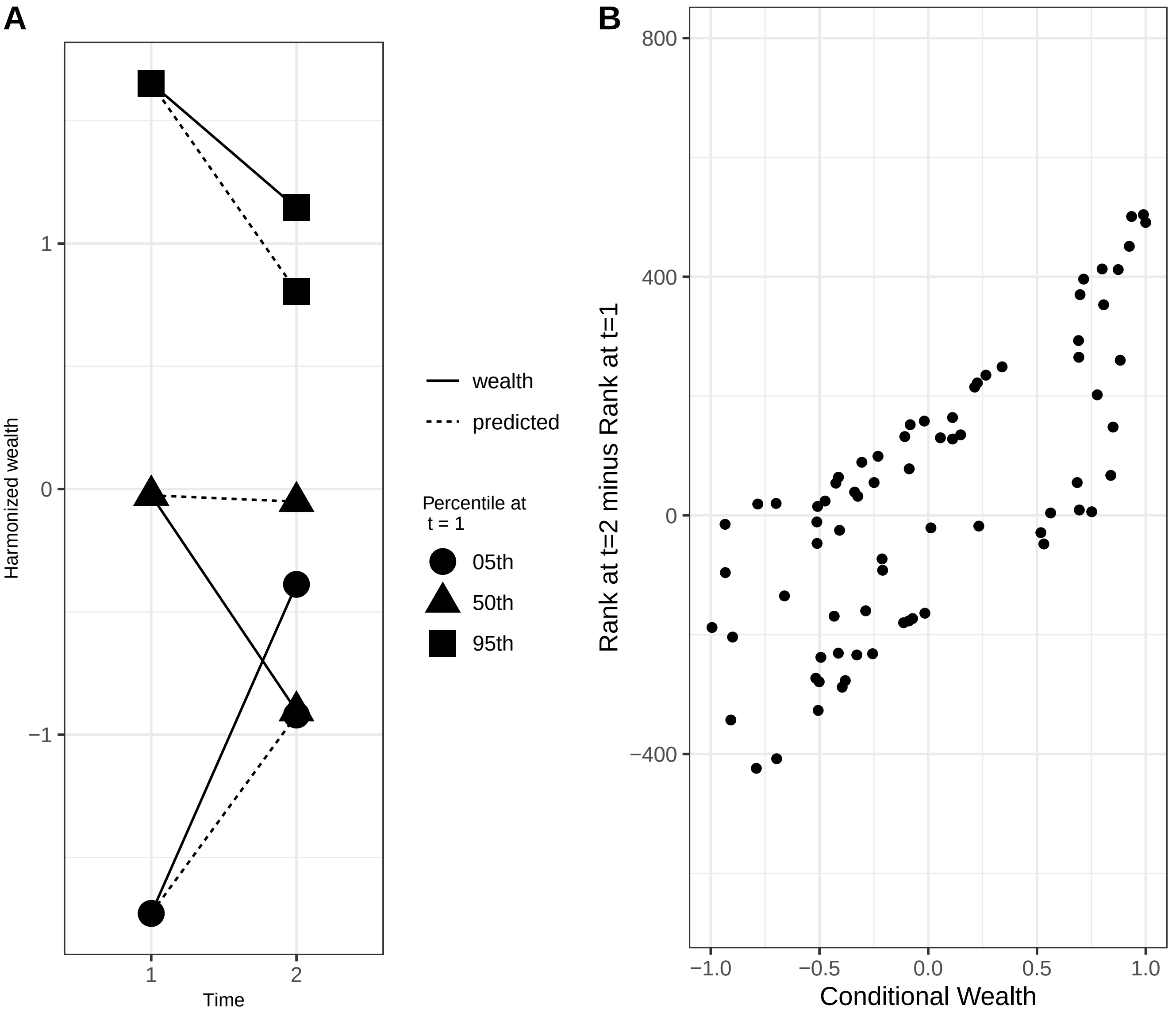

**S2Fig 96:**
**Example with w_1 = N(0,1)**
**w_2 = 0.3 + 0.5*w_1 + N(0,1)**
**Ratio of variance at time 2: time 1 = 1.2**

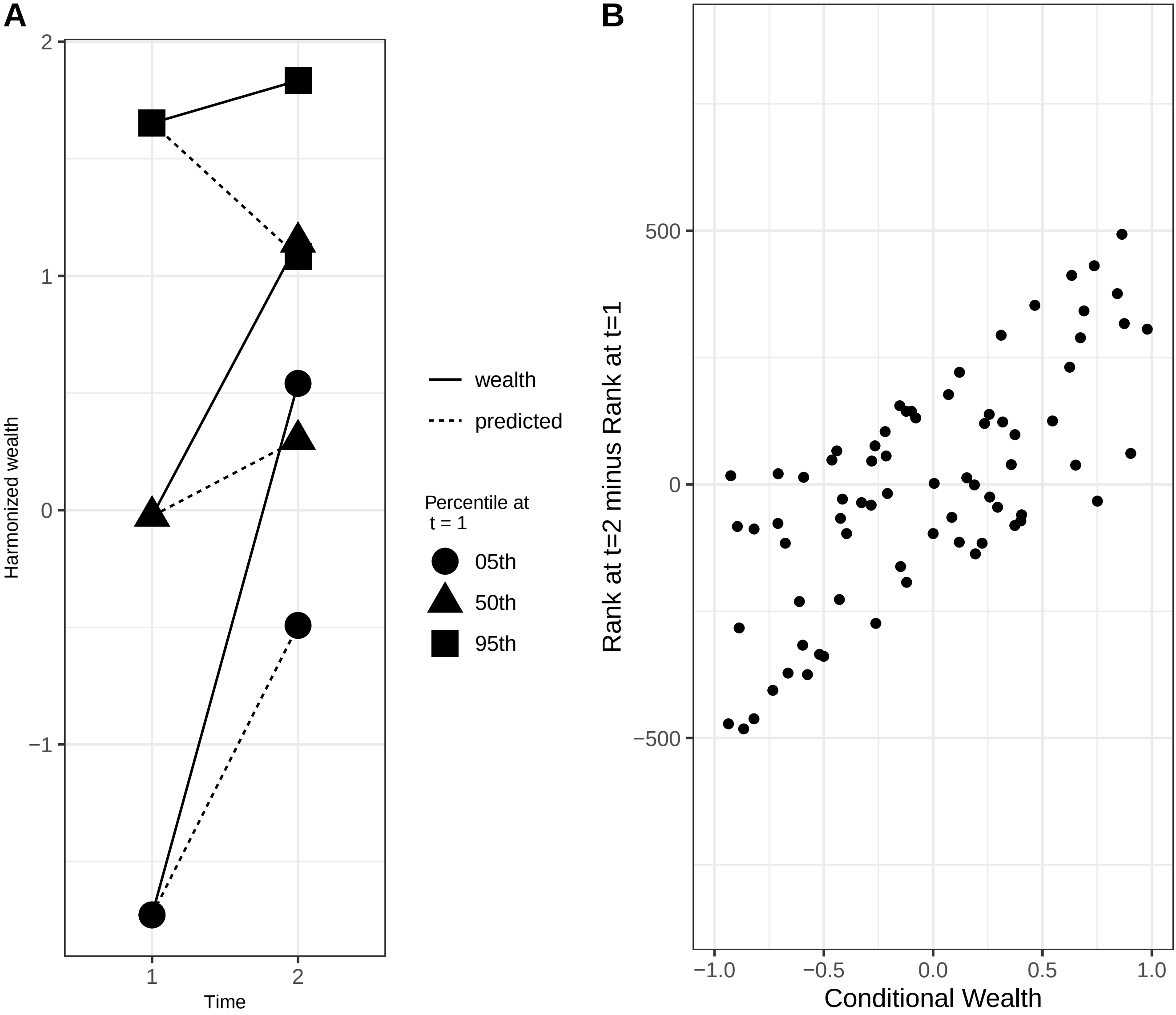

**S2Fig 97:**
**Example with w_1 = N(0,1)**
**w_2 = 0.5 + 0.5*w_1 + N(0,1)**
**Ratio of variance at time 2: time 1 = 1.2**

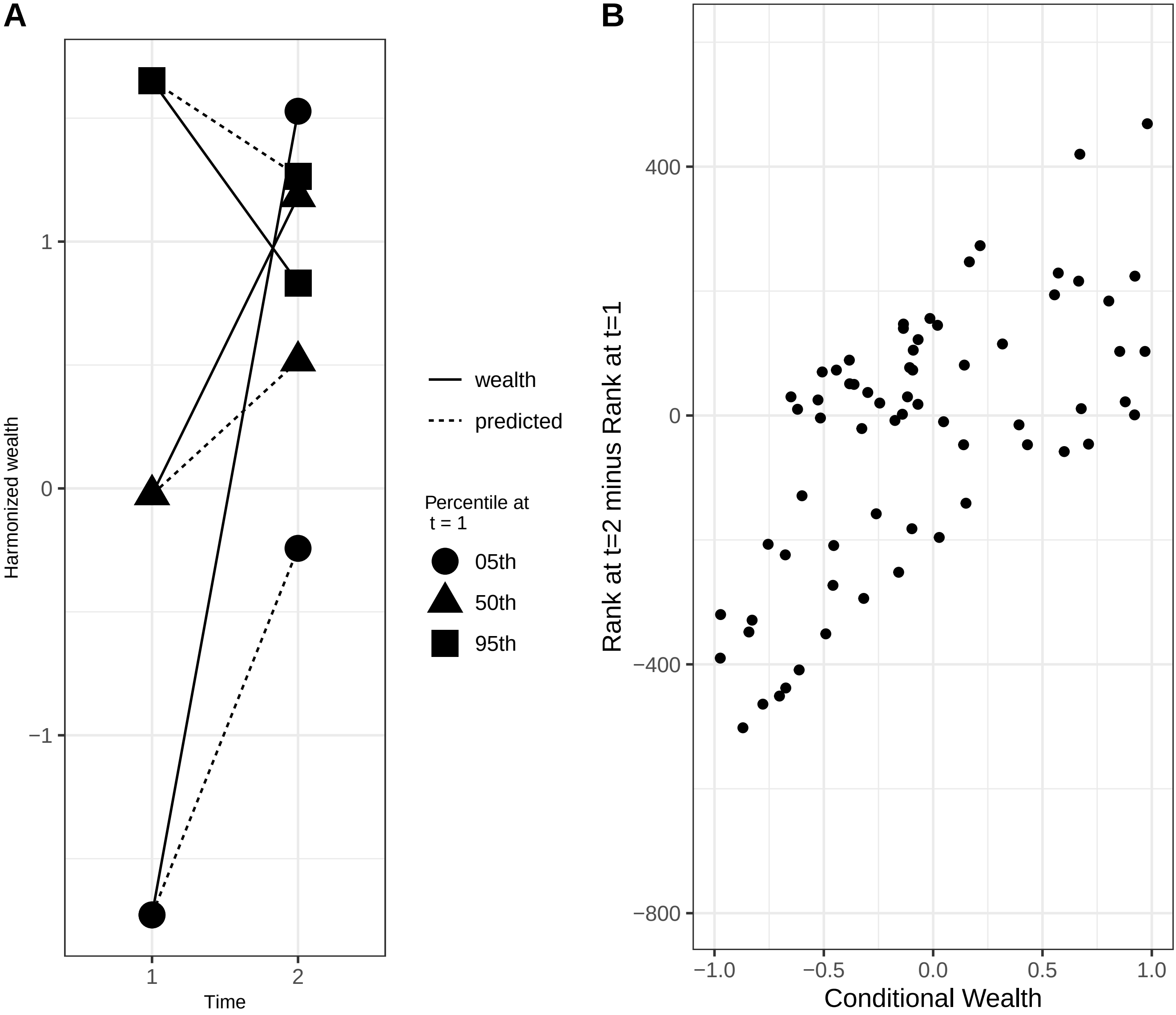

**S2Fig 98:**
**Example with w_1 = N(0,1)**
**w_2 = 1 + 0.5*w_1 + N(0,1)**
**Ratio of variance at time 2: time 1 = 1.2**

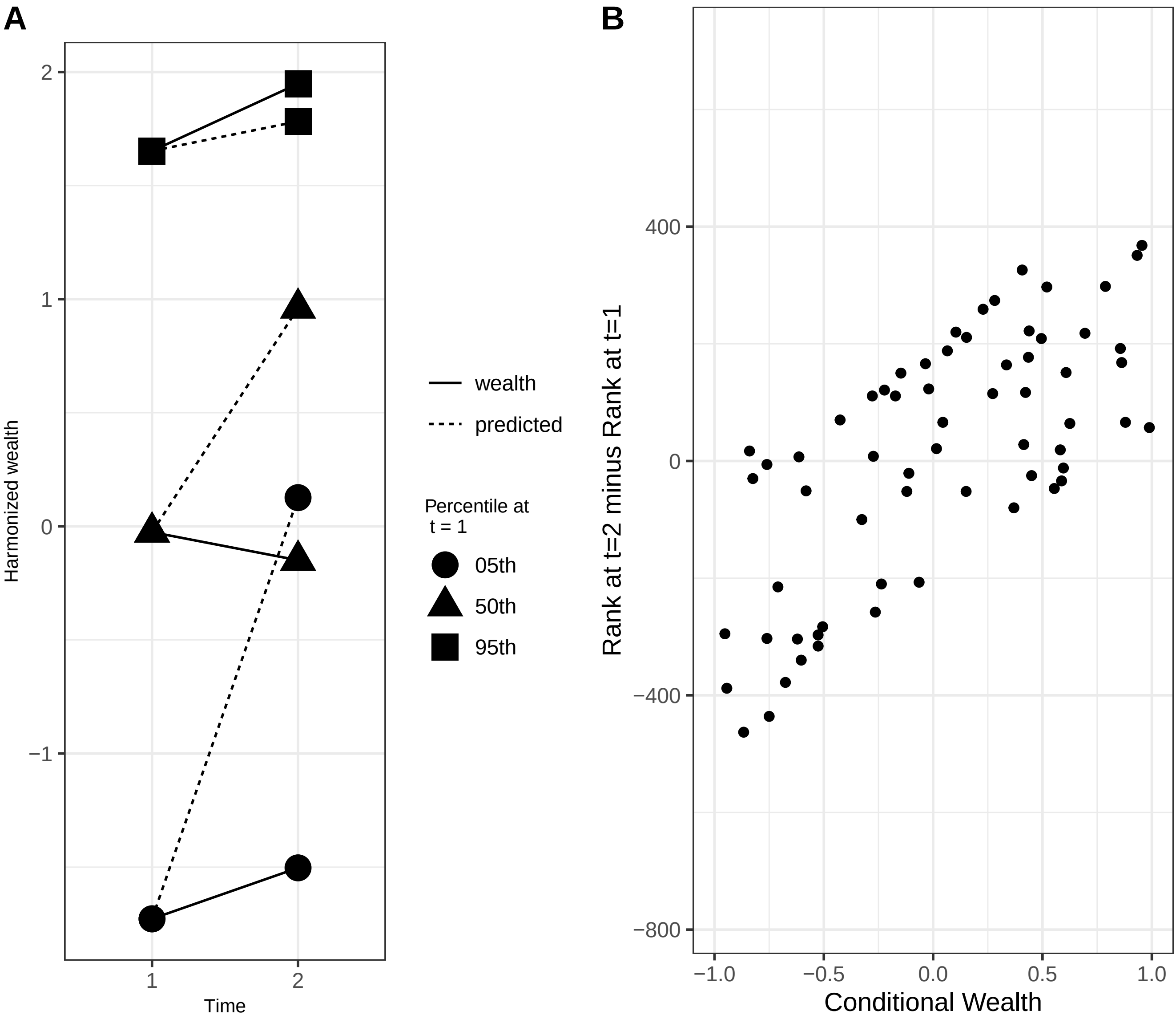

**S2Fig 99:**
**Example with w_1 = N(0,1)**
**w_2 = −1 + 1*w_1 + N(0,1)**
**Ratio of variance at time 2: time 1 = 2**

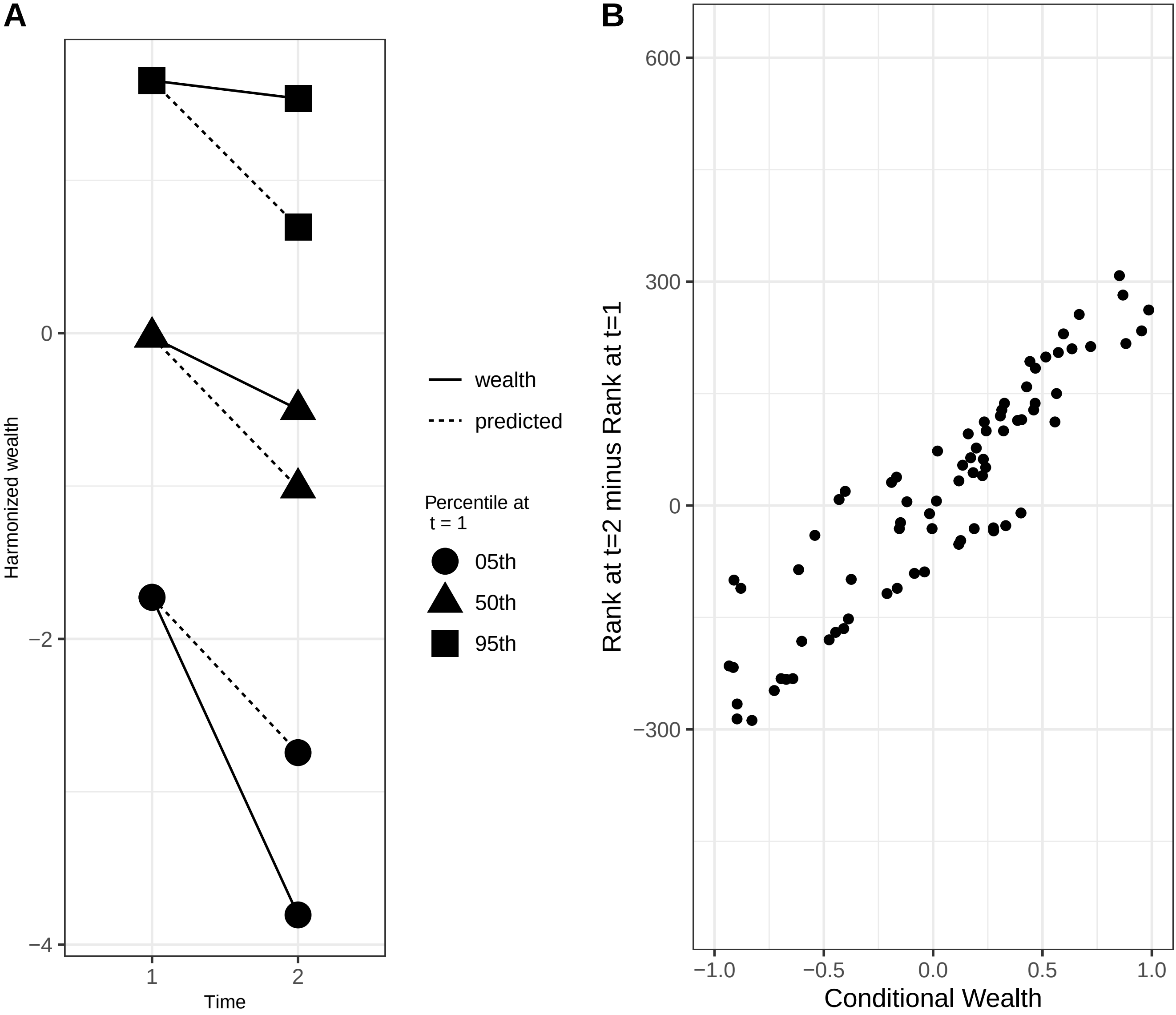

**S2Fig 100:**
**Example with w_1 = N(0,1)**
**w_2 = −0.5 + 1*w_1 + N(0,1)**
**Ratio of variance at time 2: time 1 = 1.9**

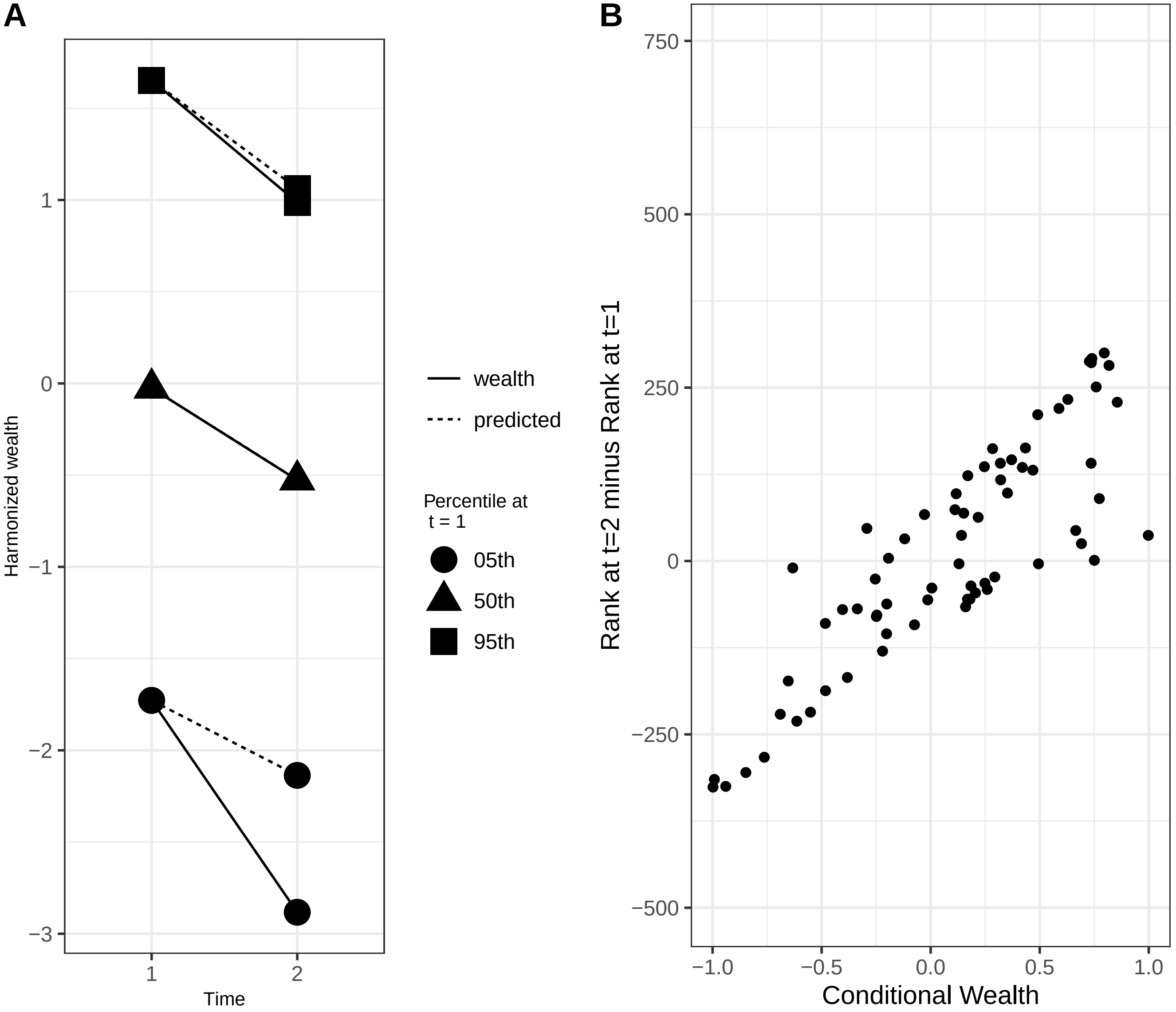

**S2Fig 101:**
**Example with w_1 = N(0,1)**
**w_2 = −0.3 + 1*w_1 + N(0,1)**
**Ratio of variance at time 2: time 1 = 2**

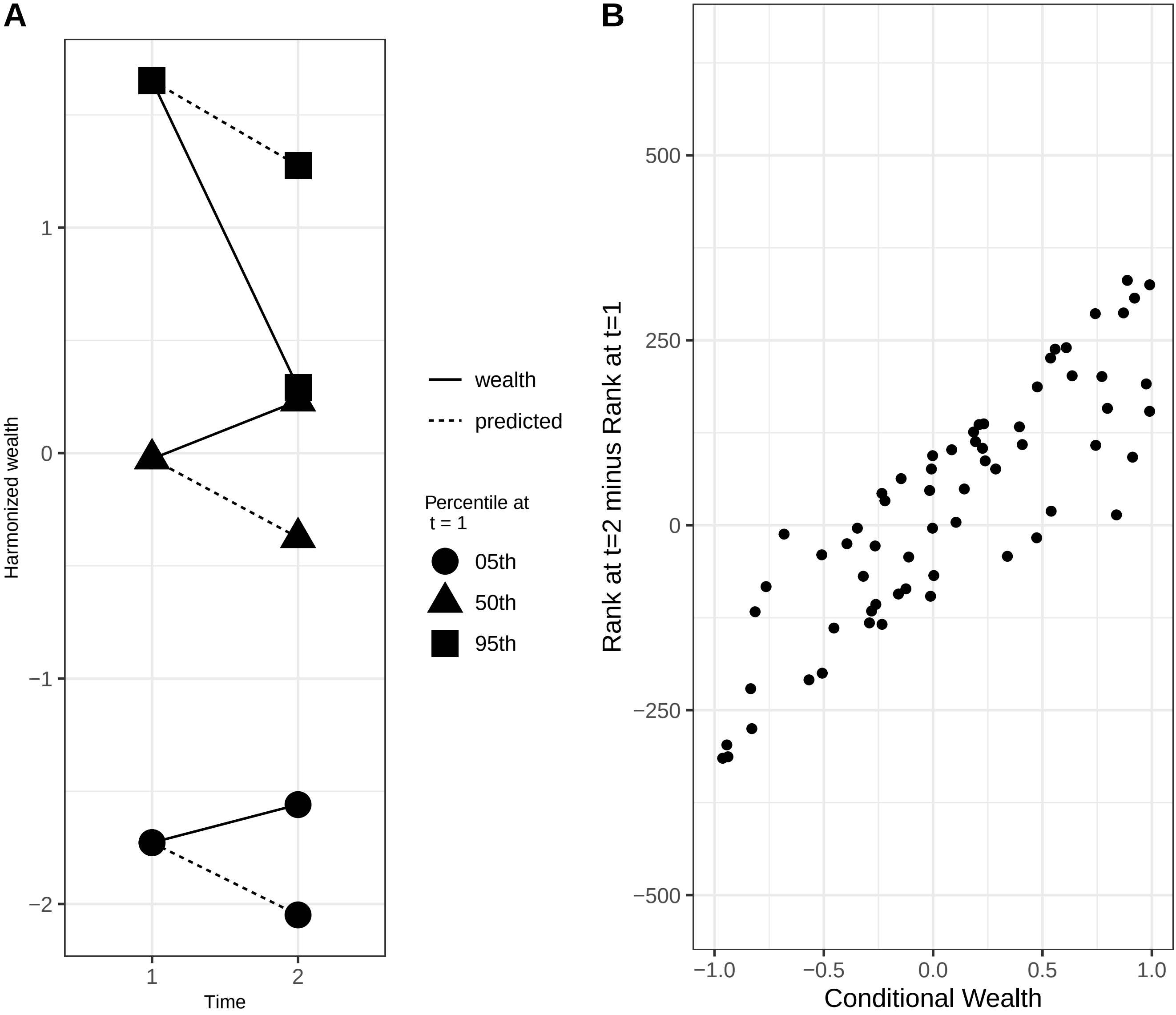

S2Fig 102:
Example with w_1 = N(0,1)
w_2 = 0 + 1*w_1 + N(0,1)
Ratio of variance at time 2: time 1 = 2.2

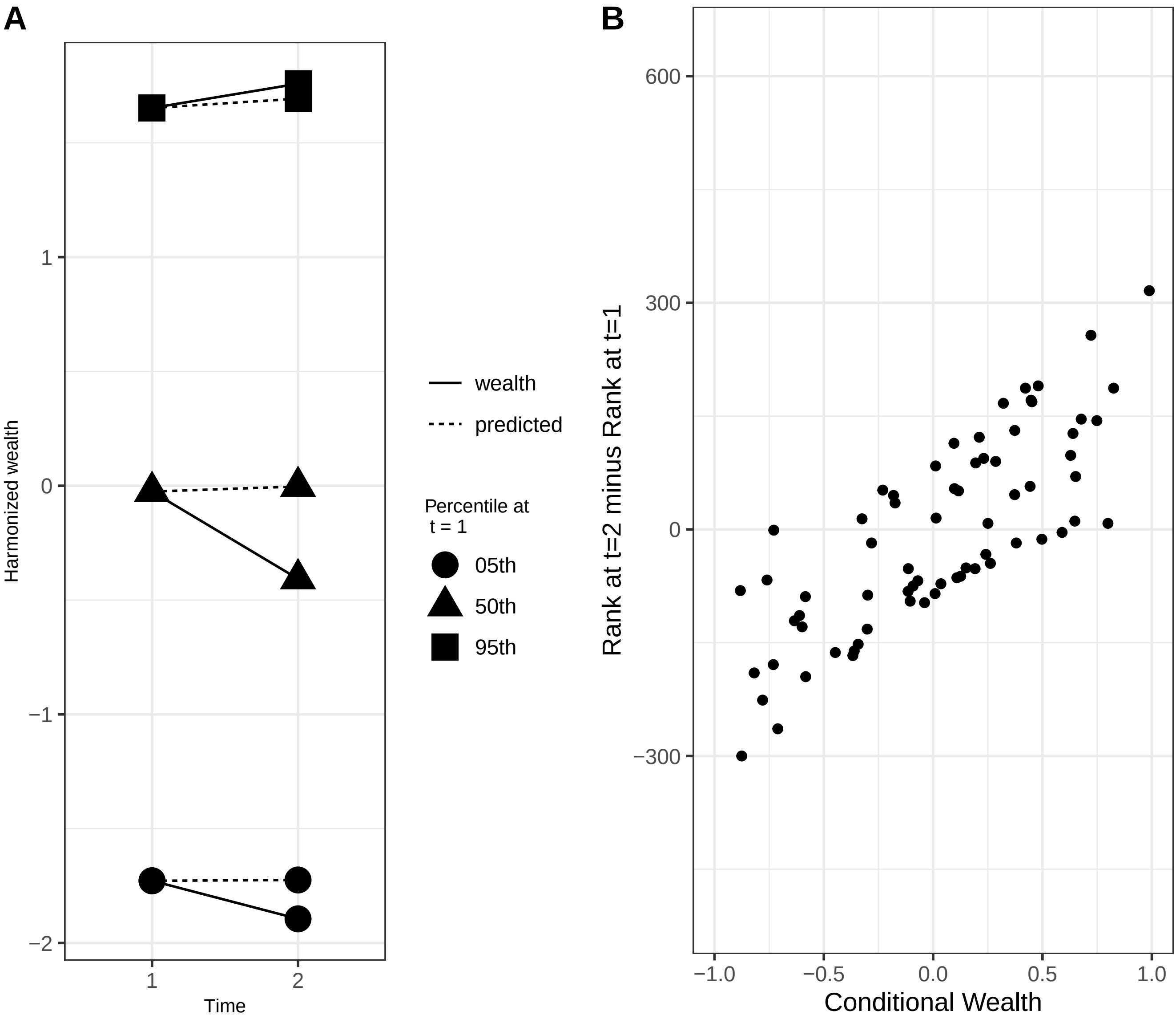

**S2Fig 103:**
**Example with w_1 = N(0,1)**
**w_2 = 0.3 + 1*w_1 + N(0,1)**
**Ratio of variance at time 2: time 1 = 2.1**

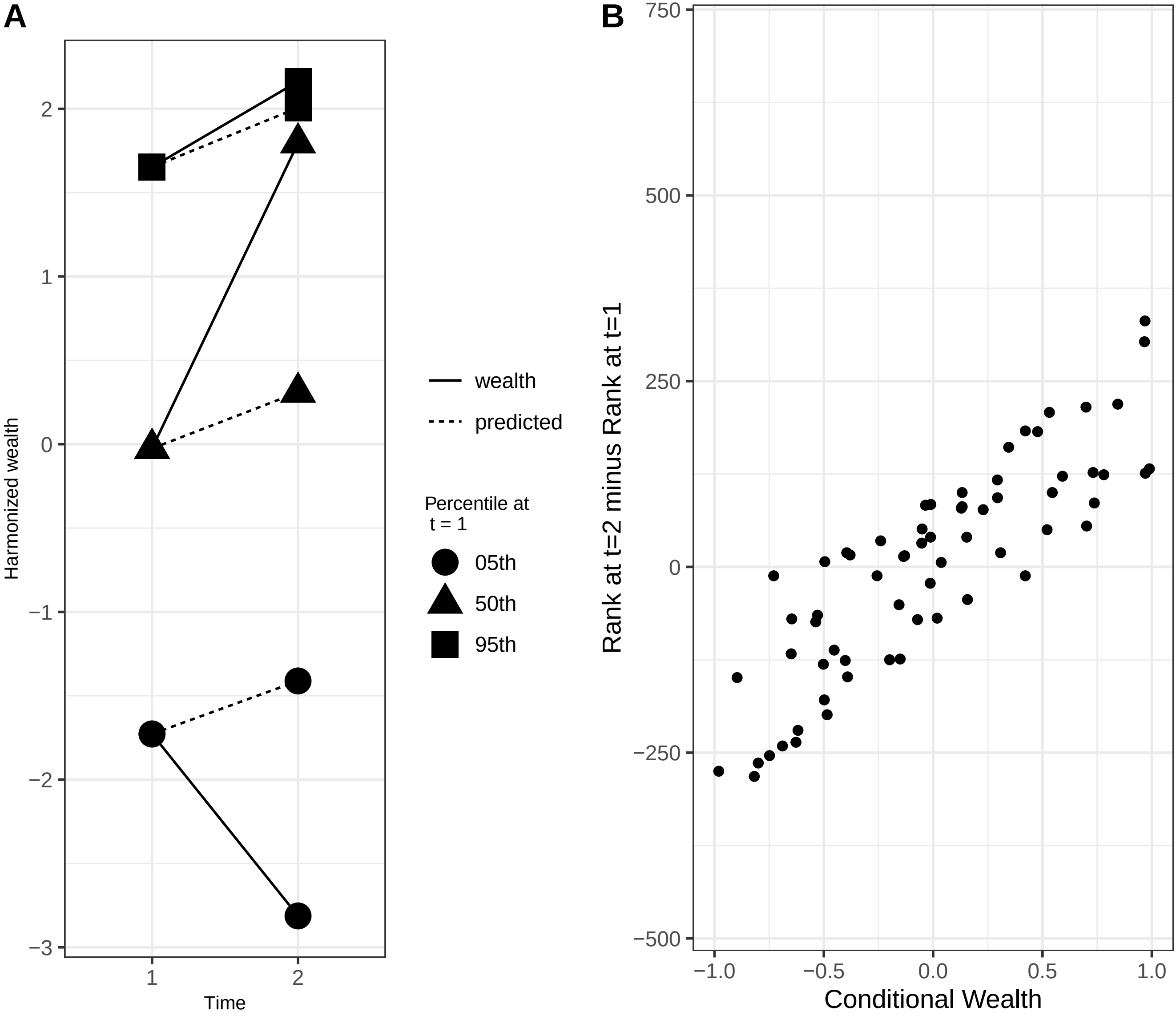

**S2Fig 104:**
**Example with w_1 = N(0,1)**
**w_2 = 0.5 + 1*w_1 + N(0,1)**
**Ratio of variance at time 2: time 1 = 2.2**

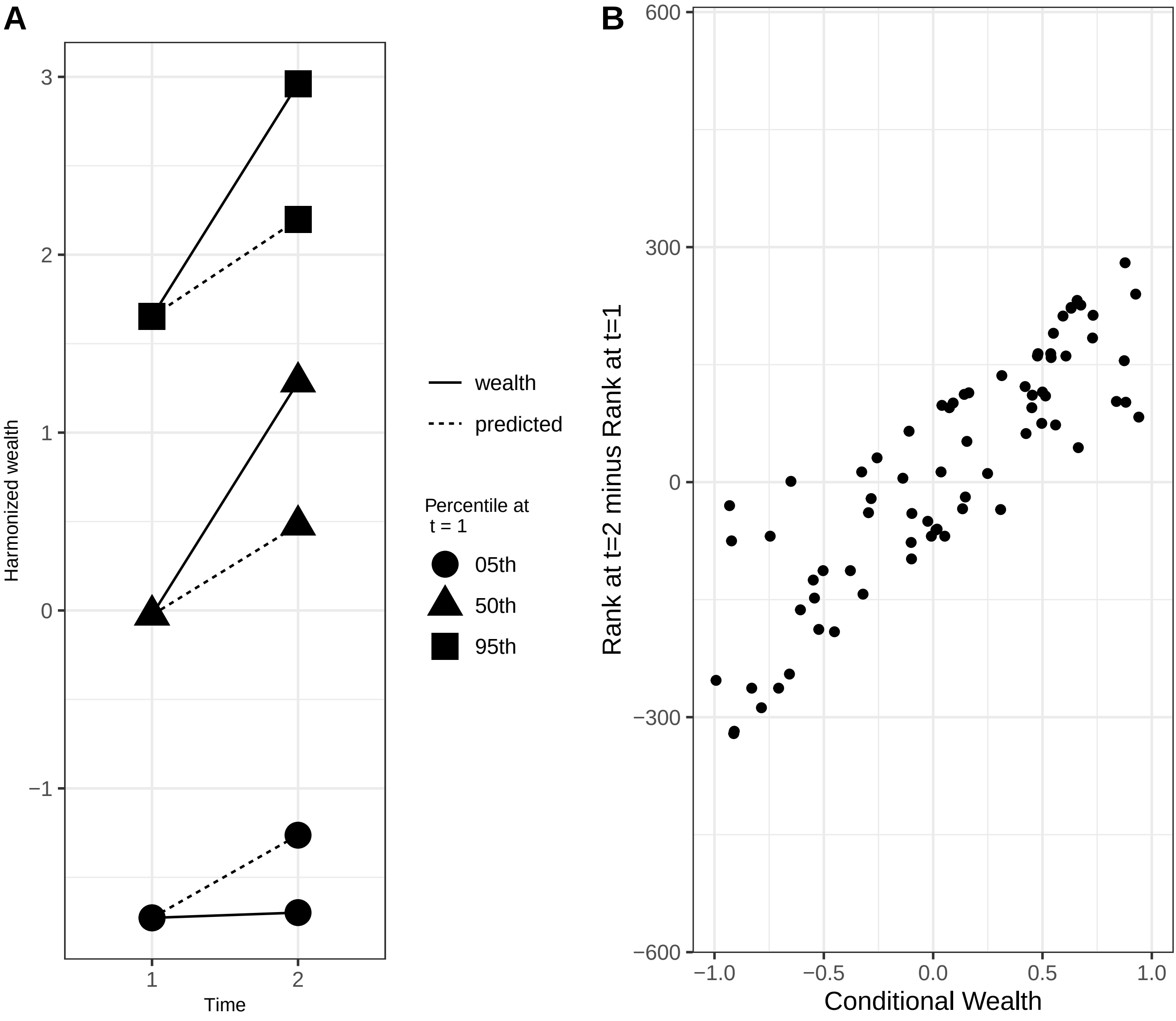

**S2Fig 105:**
**Example with w_1 = N(0,1)**
**w_2 = 1 + 1*w_1 + N(0,1)**
**Ratio of variance at time 2: time 1 = 2.1**

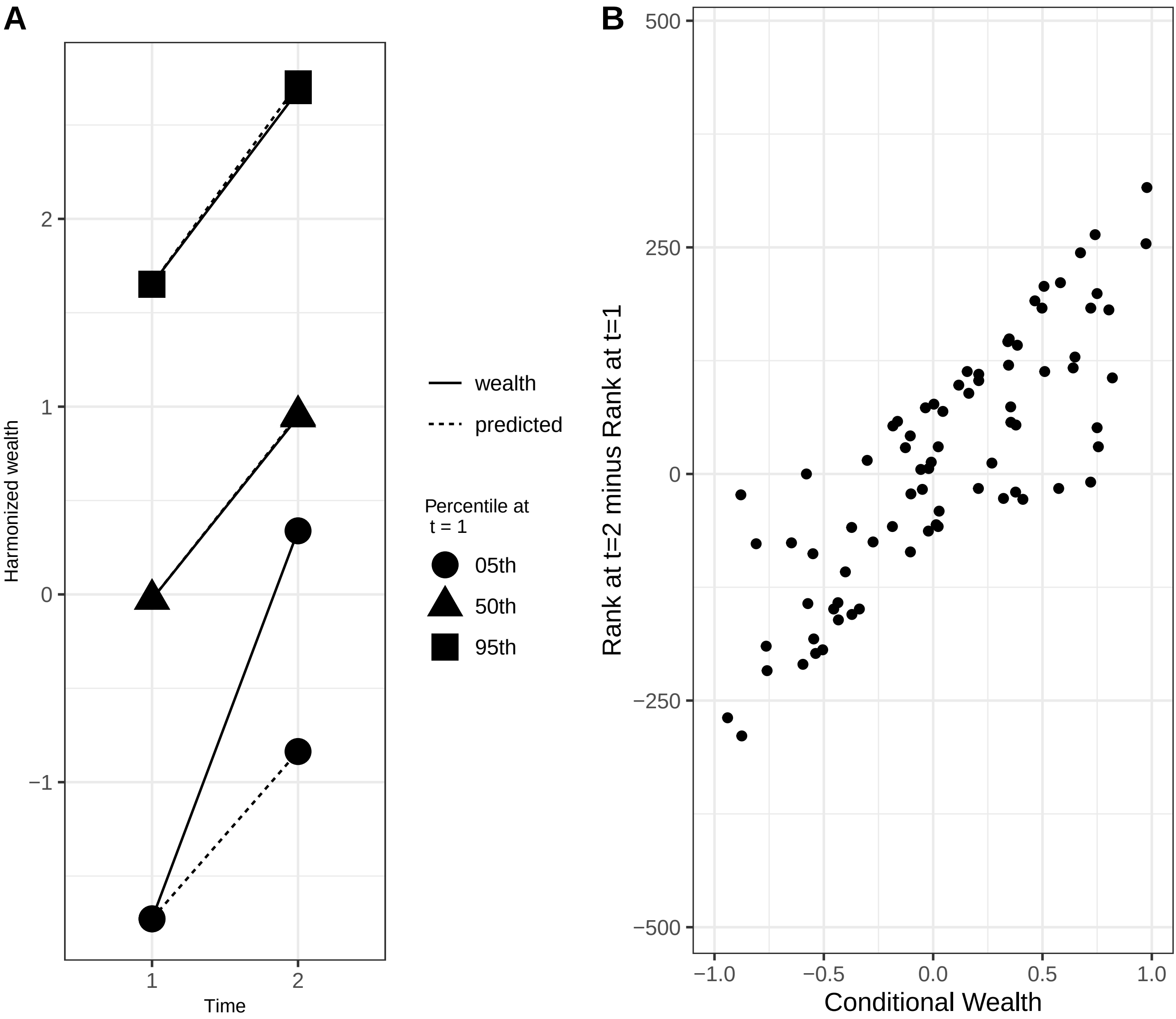

Supplement: Supplementary file 2 — Additional file 2. [file 12874_2022_1757_MOESM2_ESM.pdf]
